# The Prognostic and Molecular Landscape of Autophagy-Related Long Noncoding RNA in Colorectal Cancer

**DOI:** 10.1155/2022/5614915

**Published:** 2022-01-20

**Authors:** YuanLin Sun, XueYuan Cao, YuChen Guo, Bin Liu, Yang Zhang

**Affiliations:** Department of Gastrointestinal Surgery, First Hospital of Jilin University, Changchun, 130021 Jilin, China

## Abstract

Current evidence suggests that autophagy is closely correlated with the pathogenesis and development of malignant tumors. This study is aimed at assessing the potential prognostic significance of autophagy-related long noncoding RNA (ARlncRNA) in colorectal cancer (CRC). 3145 ARlncRNAs were obtained from autophagy-related genes (ARGs) by Pearson correlation analysis, and we established a competing endogenous RNA (ceRNA) network mediated by ARlncRNAs. A novel six-ARlncRNA prognostic signature was constructed based on TCGA samples used as the training group. Kaplan–Meier survival analysis and independent prognosis analysis were performed on the internal (training and test groups) and external validations (GEO datasets) to assess the accuracy and clinical practicability. Moreover, the nomogram combining the two independent prognostic factors (age and ARlncRNA-risk score (ARlncRNA-RS)) intuitively displayed overall survival. Gene set enrichment analysis (GSEA) conducted on the prognostic signature revealed that the gene set of the high-risk group was significantly enriched in the hallmark gene set “hypoxia” and the gene set of the low-risk group was enriched in KEGG pathways, including “peroxisome,” “the citrate cycle (TCA cycle),” and “other glycan degradation.” Assessment of antineoplastic therapy susceptibility and microsatellite instability (MSI) analysis were performed on CRC samples based on the prognostic signature. Moreover, Spearman correlation analysis was conducted on the expression of six ARlncRNAs of the prognostic signature and cancer stem cell (CSC) index as well as the tumor microenvironment (TME). In conclusion, this study established a six-ARlncRNA prognostic signature, which yielded favorable prognostic significance and demonstrated the correlation between ARlncRNAs and CRC progression.

## 1. Introduction

CRC is a common malignant tumor, and its morbidity and mortality show an increasing trend globally over the past 30 years [1]. Colonoscopy [2, 3] is available currently, and such early screening means can effectively prevent the occurrence of CRC, but its hidden onset, long evolution time, and high malignancy grade [4, 5] have frequently led to poor prognosis. At present, early surgical intervention and postoperative chemotherapy [6, 7] remain the major radical treatments for CRC. With the development of molecular mechanism research, the mechanism by which the option of therapeutic regimens is restricted by various gene levels becomes increasingly clear; for instance, fluorouracil treatment is not recommended at the time of DYPB homozygous mutation [8] and cetuximab has poorer therapeutic effect on KRAS mutation patients than on KRAS wild-type patients [9]. Therefore, the importance of individualized treatment has become increasingly prominent. Nonetheless, the CRC prognosis assessment remains the urgent hotspot to be investigated due to its complicated molecular mechanism.

Autophagy is defined as a process in which all intracellular substances are degraded in lysosomes, while the macromolecular components are recycled to maintain the dynamic balance of cellular function [10, 11]. For malignant tumors, autophagy is a double-edged sword, which can not only prevent malignant transformation of normal cells [12] but also promote tumor cell survival to indirectly boost tumor cell growth and metastasis [13]. Recently, with the deepening of research on the regulation of autophagy-targeting individualized treatment at the gene level [14], research on the role of lncRNA in autophagy in the context of tumor has been conducted gradually. lncRNA plays an important role in human physiological processes, including epigenetics [15], cell growth, and apoptosis [16], and cell differentiation [17]. Among them, the effect of lncRNA on autophagy is mainly achieved through directly or indirectly regulating genes to affect autophagic function. Chen et al. discovered that HULC might suppress ARG7 and regulate ITGB1 to promote the genesis of ovarian cancer [18]. Some research also indicates that the overexpressed PTENP1 in hepatocellular carcinoma (HCC) competitively binds with miR-17, miR-19b, and miR-20a to promote the ULK1, ARG7, and p62 autophagic genes to complete the autophagy process and to suppress HCC progression [19].

With the bioinformatic method, this study constructed the coexpression network of lncRNAs and established the prognostic signature based on the six ARlncRNAs to further explore the role of ARlncRNA in prognosis of CRC patients. Meanwhile, the regulatory correlation between ARlncRNA and other factors in CRC progression was further revealed through constructing the ARlncRNA-mediated ceRNA network. Moreover, GSEA hierarchically manifested the biological functions related to the genesis and progression of CRC. Antineoplastic therapy susceptibility analysis centering for the prognostic signature illustrated that ARlncRNA-RS was an ideal prognostic indicator for CRC patients accepting immunotherapy or traditional chemotherapy. Moreover, Spearman correlation analysis conducted for the six-ARlncRNA expression and CSC index as well as six-ARlncRNA expression and TME provided fresh perspectives of molecular regulatory relationships in CRC patients.

## 2. Methods

### 2.1. Data Acquisition

The original profiles of mRNAs, lncRNAs, and clinical data of CRC were obtained from TCGA database, and the Wilcoxon test with “edgeR package” of R software was used for differential analysis of mRNAs and lncRNAs (∣log_2_fold change | (∣logFC∣) > 1, false discovery rate (FDR) < 0.05) to obtain the differentially expressed mRNAs (DEmRNAs) and differentially expressed lncRNAs (DElncRNAs). The ARGs were obtained from the Human Autophagy Database (HADb), and then, ARGs were intersected with the mRNA profiles (all mRNA data were corrected by log_2_ (count + 1)) to obtain the ARGs of CRC. Later, ARGs were conducted by Pearson correlation analysis with lncRNAs of CRC (Cor > 0.4, *P* < 0.001) to obtain the ARlncRNAs in CRC.

### 2.2. The ceRNA Network Mediated by ARlncRNAs

To further explore the regulatory mechanisms of ARlncRNAs with other factors, we constructed the ARlncRNA-mediated ceRNA network. Obtained ARlncRNAs and DElncRNAs were intersected to acquire the target ARlncRNAs. Afterward, miRNA profiles were obtained from the TCGA database and the Wilcoxon test with the “egdeR package” of R software was adopted for differential analysis (∣logFC | >1, FDR < 0.05) to obtain the DEmiRNAs. Furthermore, the miRcode (http://www.mircode.org/) [20] was used to acquire target ARlncRNAs corresponding to DEmiRNAs. Later, the target mRNAs of DEmiRNAs were obtained using the Perl language from the miRDB [21], miRTarBase [22], and TargetScan database [23], which were then intersected with DEmRNAs to acquire the target DEmRNAs of CRC to determine the relationships between DEmiRNAs and DEmRNAs. Moreover, the Cytoscape 3.7.2 [24] was used for visualization to construct the target ARlncRNA-DEmiRNA-DEmRNA network. Meanwhile, we used The Database for Annotation, Visualization and Integrated Discovery (DAVID) to conduct Gene Ontology (GO) functional annotation and KOBAS 3.0 (http://kobas.cbi.pku.edu.cn/) to perform Kyoto Encyclopedia of Genes and Genomes (KEGG) pathway functional enrichment analysis for the downstream DEmRNAs of the ceRNA network to clarify the downstream DEmRNAs of ceRNA network-related biological functions in CRC. The “GO plot package” of R software and Cytoscape 3.7.2 were used to the visualization of the relationships of GO terms and downstream DEmRNAs and the correlations between the KEGG pathways and downstream DEmRNAs.

### 2.3. The Construction of the Prognostic Signature Based on ARlncRNAs

We excluded patients with a survival time of less than or equal to 30 days, since they might die of acute disease such as cardiovascular and cerebrovascular disease rather than CRC. Then, the expression of ARlncRNAs in various clinical samples which were retrieved from TCGA and GEO was integrated with the corresponding patients' survival time and survival status. Meanwhile, the clinical samples retrieved from TCGA were randomly divided into the training group (*n* = 248) and test group (*n* = 248) at a ratio of 1 : 1. And clinical samples retrieved from the GEO were defined as the validation group (*n* = 294). The files of the training group were conducted with univariate Cox regression analysis (*P* < 0.05) and corrected by LASSO regression analysis to obtain the prognosis-associated ARlncRNAs. Later, the prognosis-associated ARlncRNAs went through performed multivariate Cox regression analysis to acquire the ARlncRNAs for the prognostic signature and the ARlncRNA-RS. ARlncRNA-RS was calculated as follows: ARlncRNA − RS = Coef_gene1_ × exp_gene1_ + Coef_gene2_ × exp_gene2_ + , ⋯, +Coef_gene*n*_ × expr_gene*n*_. In addition, it was intuitively observed that ARlncRNAs with HR < 1 were defined as low-risk ARlncRNAs, while those with HR ≥ 1 were defined as the high-risk ARlncRNAs. In addition, patients were classified as the high-risk group and low-risk group according to the median ARlncRNA-RS. Namely, patients with ARlncRNA-RS greater than or equal to the median ARlncRNA-RS were categorized into the high-risk group and patients with ARlncRNA-RS less than the median ARlncRNA-RS were categorized into the low-risk group. Moreover, to assess the prognostic significance for the prognostic signature, we performed Kaplan–Meier survival analysis on both the high- and low-risk groups to judge whether the prognostic signature was statistically significant to the patients' survival. Meanwhile, the results of Kaplan–Meier survival analysis on the training group were examined by the test group and validation group retrieved from GEO datasets (GSE17536 and GSE103479). Moreover, we used independent prognosis analysis to determine whether various clinical features and the prognostic signature could be considered the independent prognostic factors to independently predict patients' prognosis. Moreover, we used the eight-autophagy-mRNA prognostic model [25], four-lncRNA prognostic model [26], and four-mRNA prognostic model [27] to further validate prognostic superiority of the prognostic signature based on ARlncRNAs.

### 2.4. Nomogram Based on the Independent Prognostic Factors

On the basis of the outcome of the independent prognosis analysis, we used the independent prognostic factors to plot the nomogram [28]. The Harrell' concordance index (C-index) and the calibration curve about the patients' long-term survival probability (3- and 5-year probability) examined the accuracy and divergence of the nomogram. The decision curve analysis (DCA) was utilized to explore the clinical benefit with clinical intervention in the patients' corresponding survival.

### 2.5. Clinical Correlation Analysis for the Six-ARlncRNA Prognostic Signature

To figure out the correlation between the six-ARlncRNA prognostic signature and clinical factors, we conducted clinical correlation analysis to evaluate the difference comparison between ARlncRNA-RS of the six-ARlncRNA prognostic signature and clinical factors, namely, age, gender, stage, pathological-T (pT), pathological-N (pN), and pathological-M (pM). The chi-square test was used for comparison of distributional differences for clinical factors in high- and low-risk groups. The Wilcoxon test was used to compare intragroup differences for ARlncRNA-RS of the six-ARlncRNA prognostic signature in clinical factors. *P* < 0.05, *P* < 0.01, and *P* < 0.001 were severally considered statistically different, highly statistically different, and markedly statistically different.

### 2.6. Gene Set Enrichment Analysis for the Prognostic Signature

GSEA was used to detect the biological functional enrichment of gene sets in various tumor samples [29]. In this study, the status and expression of the gene set in various clinical samples of the high- and low-risk groups in the prognostic signature were analyzed with GSEA based on the KEGG and hallmark gene sets to identify the biological pathways and functions in the high- and low-risk groups. NOM *P* < 0.01 and FDR *q* < 0.25 were deemed as the filter criteria.

### 2.7. Antineoplastic Therapy Susceptibility and MSI

The antineoplastic therapy susceptibility analyses containing immune checkpoint inhibitor (ICI) therapy susceptibility analysis and conventional chemotherapy susceptibility analysis based on the six-ARlncRNA prognostic signature was illustrated with Tumor Immune Dysfunction and Exclusion (TIDE) algorithm [30] and pRRophetic package” of R software [31], respectively. TIDE algorithm was utilized to predict the anti-PD-1 and anti-CTLA4 immunotherapeutic response of CRC patients. With the increase of the TIDE score, the probability of immune escape escalated and the immunotherapeutic response worsened. Integrated with the gene expression of TCGA CRC samples and the gene expression of CRC cell lines of the Cancer Genome Project (CGP), the algorithm driven by “pRRophetic package” of R software based on ridge regression analysis [32] was applied on the examination of conventional chemotherapy susceptibility for the high- and low-risk groups based on the six-ARlncRNA prognostic signature. A total of 138 kinds of antineoplastic drugs were presented with the susceptibility differences in the high- and low-risk groups. IC50 (50% inhibitory concentration) was used to assess the conventional chemotherapy susceptibility, and patients with lower IC50 were more sensitive to antineoplastic therapy. MSI statuses (MSI-L, MSI-H, and MSS) of CRC, searched from the Cancer Immunome Atlas (TCIA) (the database providing the results of immune genomic analysis based on the next generation sequence (NGS) data of 20 kinds of carcinomas in TCGA) [33], were employed to compare differences in the high- and low-risk groups.

### 2.8. Further Exploration for the Six ARlncRNAs of the Prognostic Signature

lnCAR (https://lncar.renlab.org/explorer), database-aggregated microarray data of approximately 60000 samples and clinical data of 13000 samples for survival analysis from 10 cancer types based on GEO database [34], was used to compare and validate the expression difference of six ARlncRNAs in CRC and normal tissues. Expression of six ARlncRNAs and the CSC index because of RNA-seq (RNAss) acquired from UCSC Xena database were conducted with Spearman correlation analysis to explore the regulatory correlation existing in ARlncRNA expression and the CSC index. Stromal score and immune score represented the relative contents of stromal components and immune cells and tumor cells in the TME [35]. The ESTIMATE score, the sum of the stromal score and immune score, indirectly signified the infiltration of tumor cells [35]. The “estimate package” of R software was used to calculate the stromal score, immune score, and ESTIMATE score based on TCGA gene expression files. Spearman correlation analysis was performed for the correlation between the expression of six ARlncRNAs and the TME score (stromal score, immune score, and ESTIMATE score). Moreover, to further uncover the innate regulatory relationship between ARlncRNAs and immune components of TME, we conducted the ANOVA for immune subtype data (C1: wound healing, C2: IFN-*γ* dominant, C3: inflammatory, C4: lymphocyte depleted, C5: immunologically quiet, and C6: TGF-*β* dominant) retrieved from UCSC Xena database and six-ARlncRNA expression and Spearman correlation analysis for the relative contents of 22 immunocytes calculated from CIBERSORT deconvolution algorithm and six-ARlncRNA expression.

## 3. Results

### 3.1. Data Acquisition

**Figure 1:**
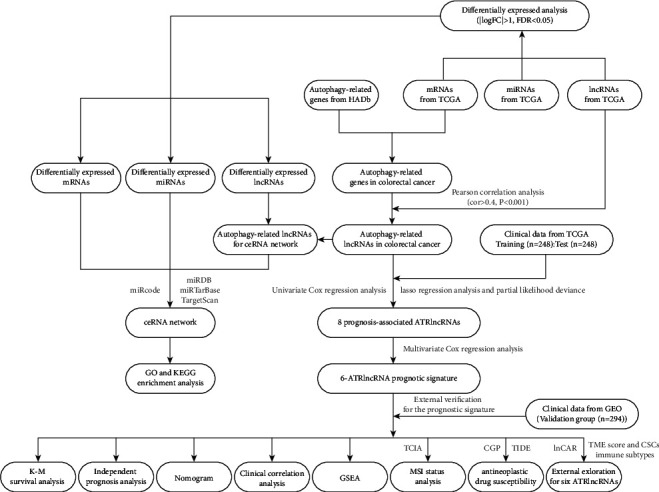
The flow chart of this study.

**Figure 2:**
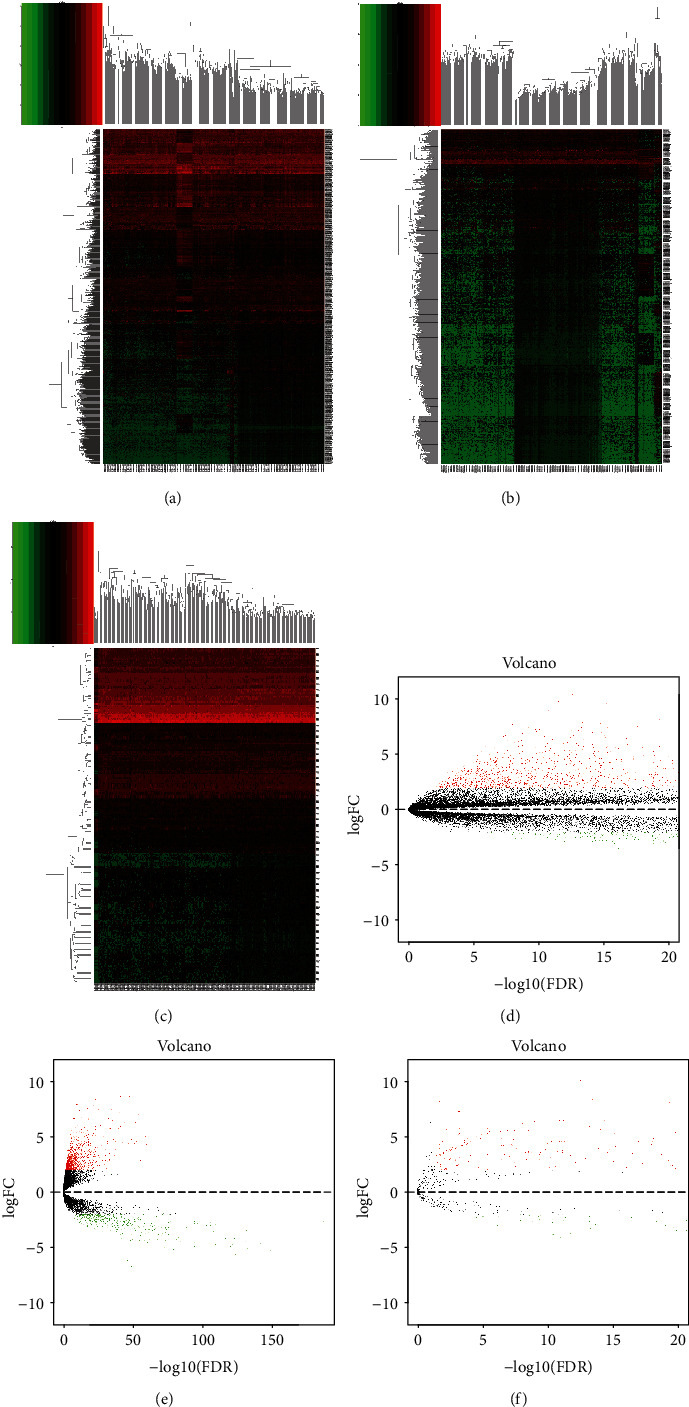
The DEmRNAs and DElncRNAs and DEmiRNAs. (a–c) The heat maps about expression quantities of DEmRNAs and DElncRNAs and DEmiRNAs. (d–f) The volcano plots about expression quantities of DEmRNAs and DElncRNAs and DEmiRNAs.

Figure 1 showed the research procedure about this study.  Table 1 showed the clinical features of CRC patients retrieved from TCGA. In this study, we obtained 2079 DEmRNAs and 1063 DElncRNAs from 562 tumor tissues and 43 normal or paratumor tissues in TCGA database with the “edgeR package” of R software, including 1084 upregulated DEmRNAs and 995 downregulated DEmRNAs (Figures 2(a) and 2(d) and Table S1) and 823 upregulated DElncRNAs and 240 downregulated DElncRNAs (Figures 2(b) and 2(e) and Table S2), respectively. Moreover, 232 ARGs were acquired from HADb (http://www.autophagy.lu/index.html), which were integrated with the mRNAs in CRC to obtain 210 ARGs in CRC. These ARGs in CRC were conducted with Pearson correlation analysis with lncRNA (Cor > 0.4, *P* < 0.001) to finally obtain 3145 ARlncRNAs.

### 3.2. The Construction of the ceRNA Network

**Figure 3:**
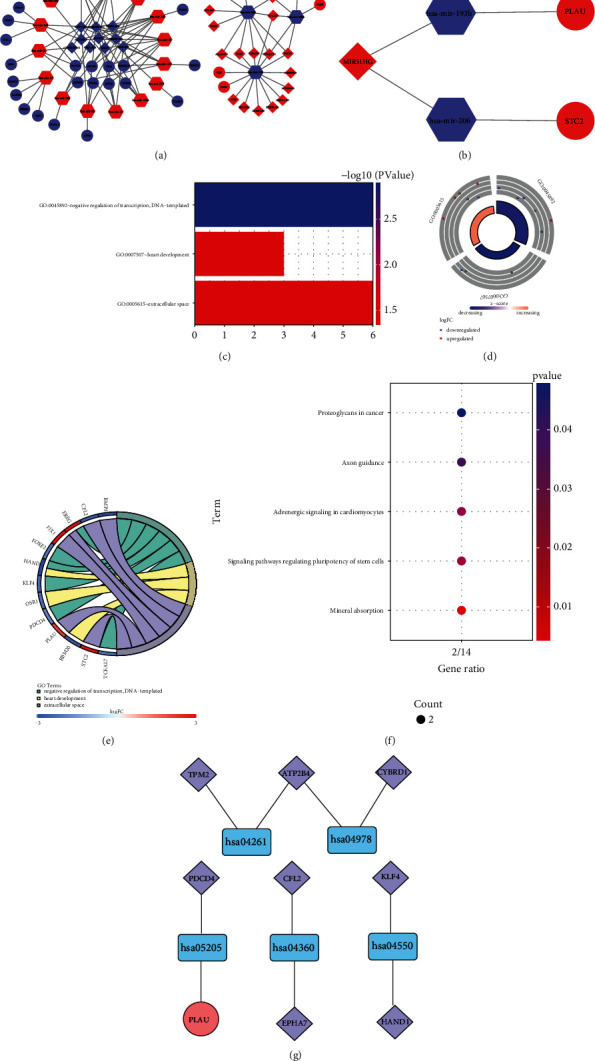
The ceRNA network mediated by ARlncRNAs. (a) The target ARlncRNA-DEmiRNA-DEmRNA ceRNA network. (b) MIR31HG/has-mir-206/STC2 axis and MIR31HG/has-mir-193b/PLAU axis. (c) GO bar plot for the three GO terms enriching the downstream DEmRNAs of the ceRNA network with statistical significance (*P* < 0.05). (d) GO circle for the distribution of the downstream DEmRNAs of the ceRNA network. The red dots represented upregulated downstream DEmRNAs and the blue dots represented downregulated downstream DEmRNAs. (e) GO chord for the distribution of official gene symbols of upregulated and downregulated downstream DEmRNAs. (f) KEGG bar plot representing the five KEGG pathways enriching the downstream DEmRNAs of the ceRNA network with statistical significance (*P* < 0.05). (g) The interaction between KEGG pathways and corresponding enriched downstream DEmRNAs. The blue round rectangles represented KEGG pathways, the red circle represented upregulated downstream DEmRNAs, and the purple rhombuses represented downregulated downstream DEmRNAs.

To better illustrate the regulatory mechanism of ARlncRNAs in CRC, we constructed the ARlncRNA-DEmiRNA-DEmRNA ceRNA network. We acquired 202 upregulated and 76 downregulated DEmiRNAs (Figures 2(c) and 2(f) and Table S3) with “edgeR package” of R software. Then, 3145 ARlncRNAs were intersected with 1063 DElncRNAs to acquire 418 target ARlncRNAs (Figure S1A). Then, 278 DEmiRNAs were integrated with miRcode via Perl language for miRNA target prediction, and 339 ARlncRNA-DEmiRNA relation pairs were obtained, including 51 ARlncRNAs and 32 DEmiRNAs. Afterward, the Perl language was used to identify 1990 DEmiRNA-target mRNA relation pairs and 1407 target mRNAs based on the miRTarBase, miRDB, and TargetScan database. Then, 1407 target mRNAs were intersected with 2079 DEmRNAs to eventually determine 56 target DEmRNAs (Figure S1B) for constructing the ceRNA network. Finally, we obtained 38 DEmiRNA-DEmRNA relation pairs and 81 ARlncRNA-DEmiRNA relation pairs for constructing the ceRNA network according to the regulatory mechanisms of the ceRNA network, which was visualized with the Cytoscape 3.7.2 (Figures 3(a) and 3(b)), including 30 ARlncRNAs, 18 DEmiRNAs, and 29 downstream DEmRNAs (Table 2). Then, with DAVID, we identified that 13 downstream DEmRNAs of 29 downstream DEmRNAs were enriched in three GO terms: GO:0045892—negative regulation of transcription; DNA templated (*P* = 0.001), GO:0007507—heart development (*P* = 0.037); and GO:0005615—extracellular space (*P* = 0.045) (Figure 3(c) and Table 3). The GO circle (Figure 3(d)) and GO chord (Figure 3(e)) visualized the results of the amounts of up- and downregulated downstream DEmRNAs enriched in three GO terms, in which GO:0045892—negative regulation of transcription, DNA-templated enriched most downregulated target DEmRNAs (TCEAL7, FOXF2, HAND1, and PDCD4, KLF4) and GO:0005615—extracellular space enriched the most upregulated target DEmRNAs (PLAU, STC2, FJX1, and EREG). Using KOBAS 3.0 for the KEGG pathway enrichment analysis, we ascertained five KEGG pathways enriching nine downstream DEmRNAs with statistical significance (*P* < 0.05), namely, hsa04978: mineral absorption (*P* = 0.005); hsa04550: signaling pathways regulating pluripotency of stem cells (*P* = 0.025); hsa04261: adrenergic signaling in cardiomyocytes (*P* = 0.027); hsa04360: axon guidance (*P* = 0.038); and hsa05205: proteoglycans in cancer (*P* = 0.048) (Table 3). Figures 3(f) and 3(g) showed the enrichment and interaction of nine downstream DEmRNAs in five KEGG pathways visually.

### 3.3. The Prognostic Signature Based on Autophagy-Related lncRNAs

**Figure 4:**
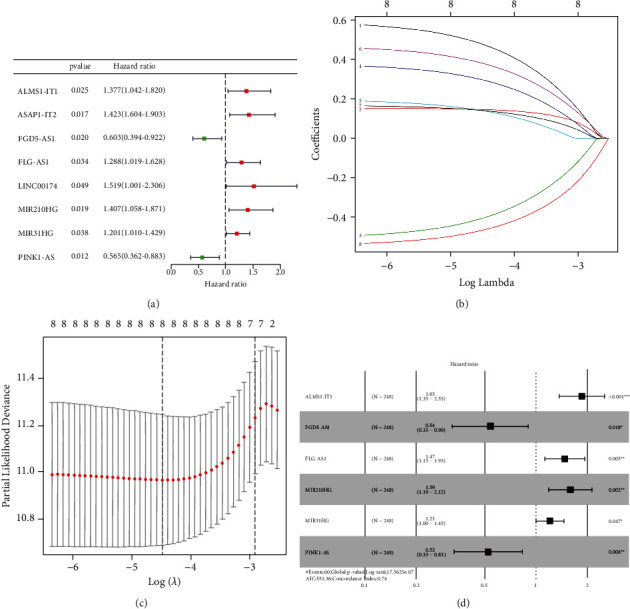
The screening of six ARlncRNAs of the prognostic signature. (a) The forest plot about the eight prognosis-associated ARlncRNAs on the basis of univariate Cox regression analysis. (b, c) The LASSO regression analysis and partial likelihood deviance on the eight prognosis-associated ARlncRNAs. (d) The forest plot about the six ARlncRNAs for the prognostic signature, in which ARlncRNAs with HR ≥ 1 were defined as high-risk ARlncRNAs and ARlncRNAs with HR < 1 were defined as low-risk ARlncRNAs.

**Figure 5:**
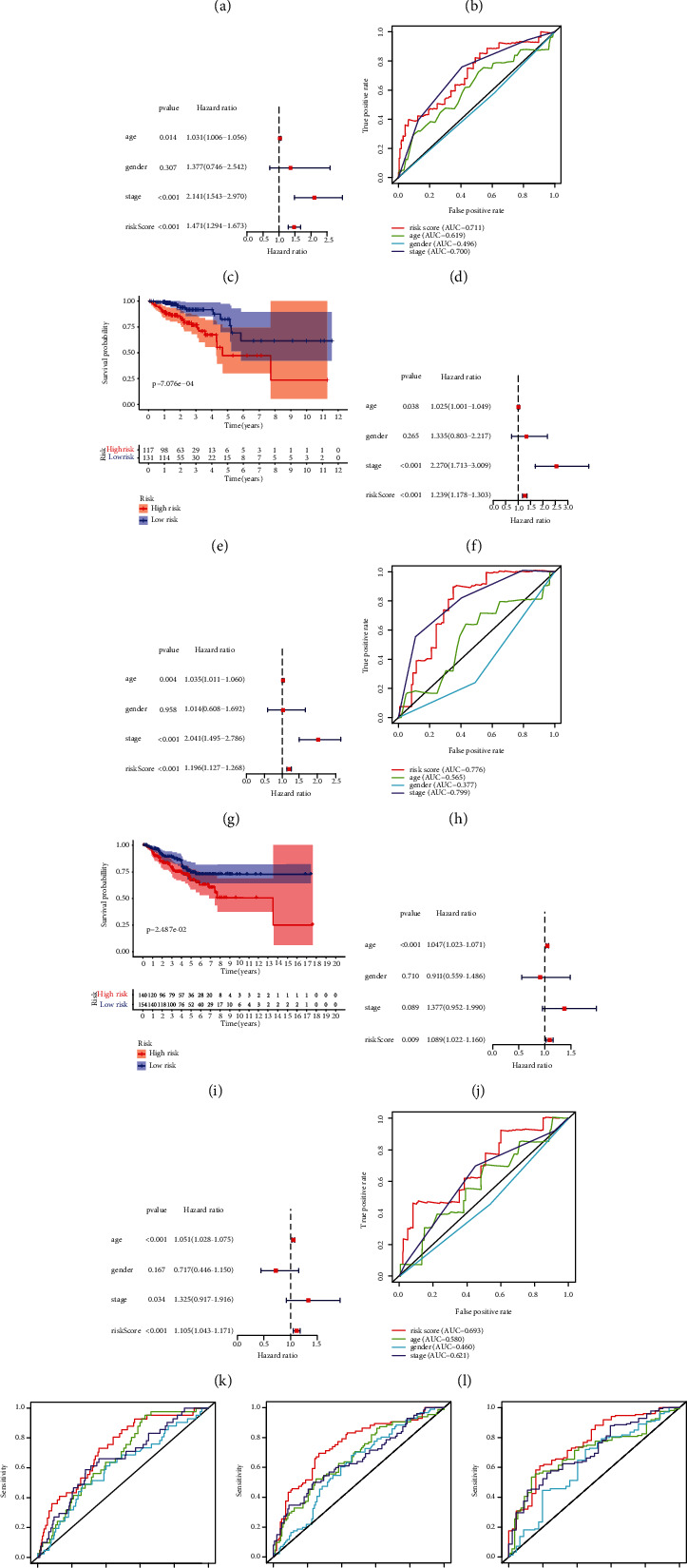
Internal and external verification for the six-ARlncRNA prognostic signature. (a, e, i) The Kaplan-Meier survival curves for the high- and low-risk groups in the training, test, and validation groups. (b, f, j) Univariate independent prognosis analysis for the training, test, and validation groups. (c, g, k) Multivariate independent prognosis analysis for the training, test, and validation groups. (d, h, l) The multi-indicator ROC curves for the training, test, and validation groups. (m–o) The 1-year, 3-year, and 5-year time-dependent ROC curves for the six-ARlncRNA prognostic signature, Xu prognostic model, Yang prognostic model, and Zhang prognostic model, in which red curves represented the six-ARlncRNA prognostic signature, green curves represented the Xu prognostic model, blue curves represented the Yang prognostic model, and purple curves represented the Zhang prognostic model.

Firstly, we used the “caret package” of R software to randomly divide the patients into the training group (*n* = 248) and test group (*n* = 248) at a ratio of 1 : 1 (Table S4). Integrating the clinical samples' survival statistics and the corresponding ARlncRNA expression quantities, we used the “survival package” of R software for univariate cox regression analysis to screen eight prognosis-associated ARlncRNAs based on the training group (*P* < 0.05) (Figure 4(a)). Then, rectification for the eight prognosis-associated ARlncRNAs was accomplished through LASSO regression analysis with “glmnet package” of R software (Figures 4(b) and 4(c)). Subsequently, we conducted the multivariate Cox regression analysis (“survival package” and “survimer package” of R software) on eight prognosis-associated ARlncRNAs with a minimum AIC value (412.23) and the prognostic signature based on six ARlncRNAs (ALMS1-IT1, FGD5-AS1, FLG-AS1, MIR210HG, MIR31HG, and PINK1-AS), including four high-risk ARlncRNAs (ALMS1-IT1, FLG-AS1, MIR210HG, and MIR31HG) and two low-risk ARlncRNAs (FGD5-AS1 and PINK1-AS) (Figure 4(d)), was obtained. The ARlncRNA − RS = 0.473 × exp_ALMS1−IT1_ + (−0.470) × exp_FGD5−AS1_ + 0.352 × exp_FLG−AS1_ + 0.395 × exp_MIR210HG_ + 0.183 × exp_MIR31HG_ + (−0.485) × exp_PINK1−AS_. Patients with the ARlncRNA-RS greater than or equal to the median ARlncRNA-RS (1.067) were categorized into the high-risk group; otherwise, they were categorized into the low-risk group. As shown in Figures 5(a) and 5(e), the Kaplan–Meier survival curves for the training and test group demonstrated that the six-ARlncRNA prognostic signature was statistically correlated with the survival probability in high- and low-risk groups (*P* = 6.662*e* − 06 and *P* = 7.076*e* − 04). Moreover, to determine whether this prognostic signature could independently predict the prognosis of CRC patients, we combined the clinical features (age, gender, and American Joint Committee on Cancer (AJCC) stage) with ARlncRNA-RS of the six-ARlncRNA prognostic signature to perform univariate and multivariate independent prognosis analyses. As shown in Figures 5(b) and 5(c), the age, AJCC-stage, and ARlncRNA-RS in the training group showed significant statistical differences, whose results were consistent with that in the test group (Figures 5(f) and 5(g)). As shown in Figures 5(d) and 5(h), the area under curves (AUC) of multi-indicator receiver operating characteristic (ROC) curves including ARlncRNA-RS, age, gender, and AJCC stage in the training and test group were 0.711, 0.619, 0.496, 0.700, 0.776, 0.565, 0.377, and 0.799, demonstrating the favorable feasibility of the six-ARlncRNA prognostic signature in predicting CRC patients' prognosis. These results suggested that ARlncRNA-RS of the six-ARlncRNA prognostic signature could serve as the independent prognostic factors to predict the prognosis of CRC patients.

### 3.4. External Verification and Prognostic Superiority of the Prognostic Signature

The strength of the prognostic signature in predicting the survival probability of patients was further verified by GEO datasets, GSE17536 and GSE103479 (batch effect has been eliminated with “sva package” of R software) (Table S4). As shown in Figure 5(i), the survival probability between the high- and low-risk groups was proved to be statistically different (*P* = 0.025), which is consistent with the training and test groups. Figures 5(j) and 5(k) showed the results of the univariate and multivariate independent prognosis analyses for the validation group, in which the age and ARlncRNA-RS were identified as the independent prognostic factors. The AUCs of multi-indicator ROC curves including ARlncRNA-RS, age, gender, and AJCC-stage (Figure 5(l)) were 0.693, 0.590, 0.460, and 0.621, respectively, which proved that the prognostic signature verified by the validation group had relatively robust accuracy. In addition, to further demonstrate the prognostic superiority of the six-ARlncRNA prognostic signature, we plotted the time-dependent ROC curves among the six-ARlncRNA prognostic signature and other three prognostic models. As shown in Figures 5(m)–5(o), the 1-year, 3-year, and 5-year AUCs of ROC curves for the six-ARlncRNA prognostic signature (0.711, 0.788, and 0.718) were all higher than those for the XuSig (eight-autophagy-gene prognostic model) (0.671, 0.666, and 0.700), YangSig (four-lncRNA prognostic model) (0.616, 0.612, and 0.667), and ZhangSig (four-mRNA prognostic model) (0.620, 0.683, and 0.715), indicating that the accuracy of the six-ARlncRNA prognostic signature in the prediction of survival was superior to the other three prognostic models.

### 3.5. Establishment and Validation of a Nomogram Based on Independent Prognostic Factors

**Figure 6:**
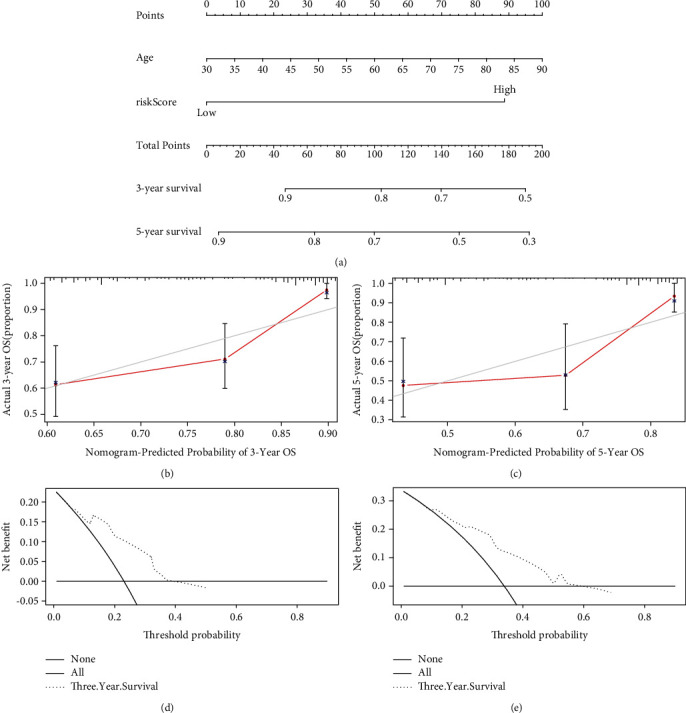
The construction of the nomogram containing two independent prognostic factors. (a) The nomogram based on independent prognostic factors (age and ARlncRNA-RS of the six-ARlncRNA prognostic signature) for the training group. (b, c) The 3- and 5-year calibration curves for the nomogram in the training group. (d, e) The 3- and 5-year DCA curves for the nomogram in the training group.

After performing univariate and multivariate independent prognosis analyses using the training, test, and validation group datasets, we then constructed a nomogram including two independent prognostic factors (age and ARlncRNA-RS of the six-ARlncRNA prognostic signature) based on the training group (Figure 6(a)), while the test group (Figure S2A) and validation group (Figure S2B) were used for verifying the accuracy and feasibility of the nomogram. To be specific, for the currently eligible patients, the corresponding scores of the “age” and “ARlncRNA-RS” were found on the nomogram; then, the corresponding scores were added to obtain the corresponding 3- and 5-year survival probability of patients. The C-indexes of the nomogram based on the training, test, and validation groups were 0.706 (95% CI: 0.637-0.774), 0.710 (95% CI: 0.624-0.796), and 0.641 (95% CI: 0.574-0.708), respectively. The calibration curves of the nomogram predicting 3- and 5-year survival based on the training (Figures 6(b) and 6(c)), test (Figure S2C and S2D), and validation groups (Figure S2G and S2H) showed good agreement. The decision curve analysis (DCA) based on the training (Figures 6(d) and 6(e)), test (Figure S2E and S2F), and validation groups (Figure S2I and S2J) demonstrated that CRC patients with relatively long-time survival (including 3- and 5-year survival) could benefit from clinical intervention.

### 3.6. Clinical Correlation Analysis

**Figure 7:**
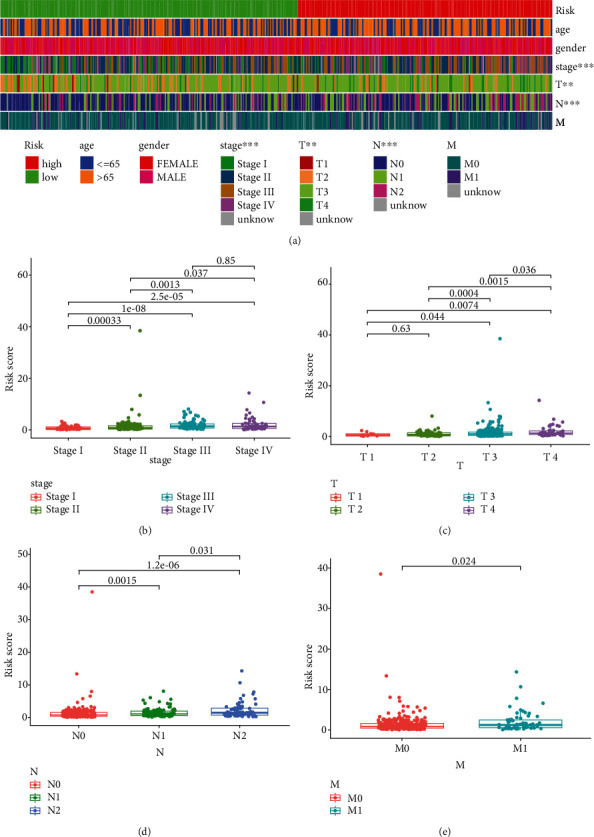
Clinical correlation analysis for the six-ARlncRNA prognostic signature. (a) The heat map showing the distributional differences of clinical factors (age, gender, stage, pT, pN, and pM) in high- and low-risk groups. ^∗^ Represented the distribution of the clinical factor in the high- and low-risk groups with statistical significance (*P* < 0.05). ^∗∗^ Represented the distribution of the clinical factor in the high- and low-risk groups with highly statistical significance (*P* < 0.01). ^∗∗∗^ Represented the distribution of the clinical factor in the high- and low-risk groups with markedly statistical significance (*P* < 0.001). (b–e) The intragroup comparison for the distribution of ARlncRNA-RS among various clinical factors. *P* < 0.05 was considered statistically significant.

On the basis of TCGA CRC patients' data, clinical correlation analysis was performed to elaborately explore the underlying relationship between the six-ARlncRNA prognostic signature and clinical factors (age, gender, stage, pT, pN, and pM). We first investigated which clinical factors were statistically significant between the high- and low-risk groups in the macrolevel. As shown in Figure 7(a), the statistically significant difference in stage and pN was found between the high- and low-risk groups (*P* < 0.001). In addition, there was a highly statistical difference between the high- and low-risk groups for pT (*P* < 0.01). Moreover, we further analyzed whether there were statistical differences between ARlncRNA-RS of the six-ARlncRNA prognostic signature among clinical features. As shown in Figures 7(b)–7(e), statistical differences for ARlncRNA-RS within the stage, pT, pN, and pM were found, specially between stage I and stage III (*P* = 1*e* − 08), pT2 and pT4 (*P* = 0.00015), pN0 and pN2 (*P* = 1.2*e* − 06), and pM0 and pM1 (*P* = 0.024).

### 3.7. Gene Set Enrichment Analysis

**Figure 8:**
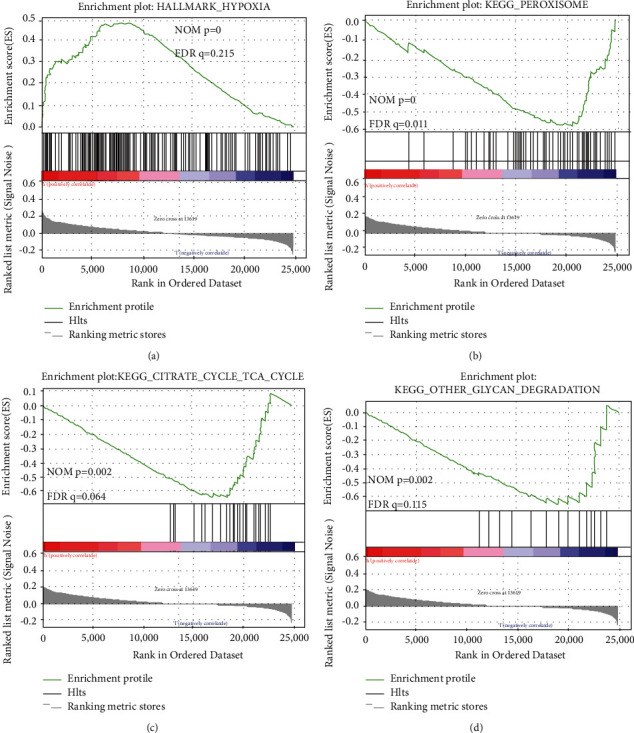
GSEA for the six-ARlncRNA prognostic signature. (a) GSEA based on hallmark gene sets for the high-risk group. (b–d) GSEA based on KEGG database for the low-risk group.

Gene set enrichment analysis (GSEA) was also conducted to further reveal the relationships between gene biological functions and tumor pathogenesis.  Table 4 showed the results of GSEA for the high- and low-risk groups. In this study, it was discovered that the gene set reannotated based on the hallmark gene sets, namely, hypoxia (NOM *P* < 0.01, FDR *q* = 0.215), was mainly enriched in the high-risk group (Figure 8(a)). In addition, there were no gene sets based on the hallmark gene sets enriched in the low-risk group. Further, the metabolism-related pathways based on the KEGG pathways were mainly enriched in the low-risk group, such as peroxisome (NOM *P* < 0.01, FDR *q* = 0.011) (Figure 8(b)), the citrate cycle (TCA cycle) (NOM *P* = 0.002, FDR *q* = 0.064) (Figure 8(c)), and other glycan degradation (NOM *P* = 0.002, FDR *q* = 0.115) (Figure 8(d)). In contrast, the high-risk group was not involved in pathways based on the KEGG pathways.

### 3.8. Overview for the Antineoplastic Therapy Susceptibilities and MSI

**Figure 9:**
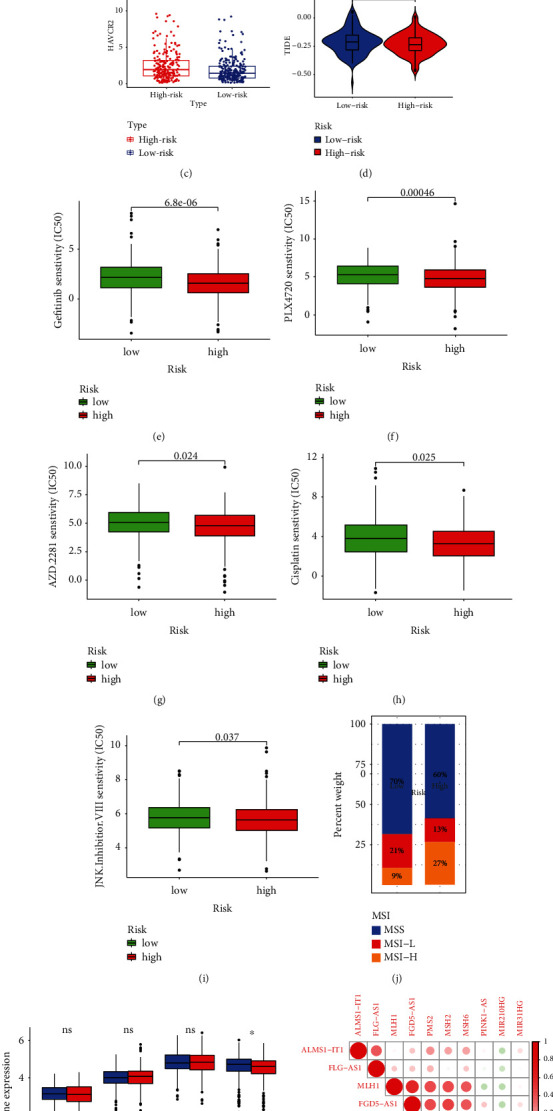
Antineoplastic therapy susceptibility and MSI analysis. (a–c) The boxplots showing the statistical differences of the expression of tumor immune check points (PDCD1, CTLA4, and HAVCR2) in the high- and low-risk groups. (d) The violin plot illustrating the TIDE score of the low-risk group was higher than that in the high-risk group with statistical significance (*P* < 0.05). (e–i) The boxplots showing the statistical differences in the IC50 of gefitinib, PLX4720, AZD.2281, cisplatin, and JNK.inhibitor.VIII in the high- and low-risk groups. (j) The bar plot depicting the distribution differences of CRC patients with different MSI statuses in the high- and low-risk groups. (k) The boxplots showing the statistical differences of the expression of characteristic genes (PMS2, MSH6, MSH2, and MLH1) of the MMR system in the high- and low-risk groups. (l) The correlation heat map illustrating the coexpression correlation among the four characteristic genes (PMS2, MSH6, MSH2, and MLH1) and six ARlncRNAs (ALMS1-IT1, FGD5-AS1, FLG-AS1, MIR210HG, MIR31HG, and PINK1-AS) of the prognostic signature.

The expression differences of tumor immune checkpoints (PDCD1, CTLA4, and HAVCR2) in the high- and low-risk groups were shown in Figures 9(a)–9(c). It was noted that the expression of HAVCR2, PDCD1, and CTLA4 was all higher in the high-risk group than in the low-risk group. TIDE algorithm characterizing anti-PD1 and anti-CTLA4 response demonstrated that CRC patients in the low-risk group were more amenable to developing immune escape capacities (Figure 9(d)). Meanwhile, we analyzed the differences of conventional chemotherapy susceptibility of the CRC patients in the high- and low-risk groups based on the six-ARlncRNA prognostic signature. As shown in Figures 9(e)–9(i), after comprehensive conventional chemotherapy susceptibility analysis for the 138 kinds of chemotherapy drug, it was noted that gefitinib (*P* = 6.8*e* − 06), PLX4720 (*P* = 0.00046), AZD.2281 (*P* = 0.024), cisplatin (*P* =0.025), and JNK.inhibitor.VIII (*P* = 0.037) were more susceptible to the CRC patients in the high-risk group than in the low-risk group. Furthermore, differences of the MSI statuses between the high- and low-risk groups were also observed. As shown in Figure 9(j), the CRC patients with MSS status and MSI-L status took larger proportions in the high-risk group compared with the low-risk group (60% vs 70% and 13% vs 21%) and CRC patients MSI-H status in the high-risk group and low-risk group occupied 27% and 9%, respectively. MLH1 exhibited a higher expression level in the low-risk group compared with the high-risk group with statistical difference (*P* < 0.05) (Figure 9(k)). In addition, the expression of MLH1 was positively correlated with the expression of the low-risk ARlncRNA, FGD5-AS1, and negatively correlated with the expression of the high-risk ARlncRNA, MIR210HG (Figure 9(l)).

### 3.9. Validation for Expression of Six ARlncRNAs

**Figure 10:**
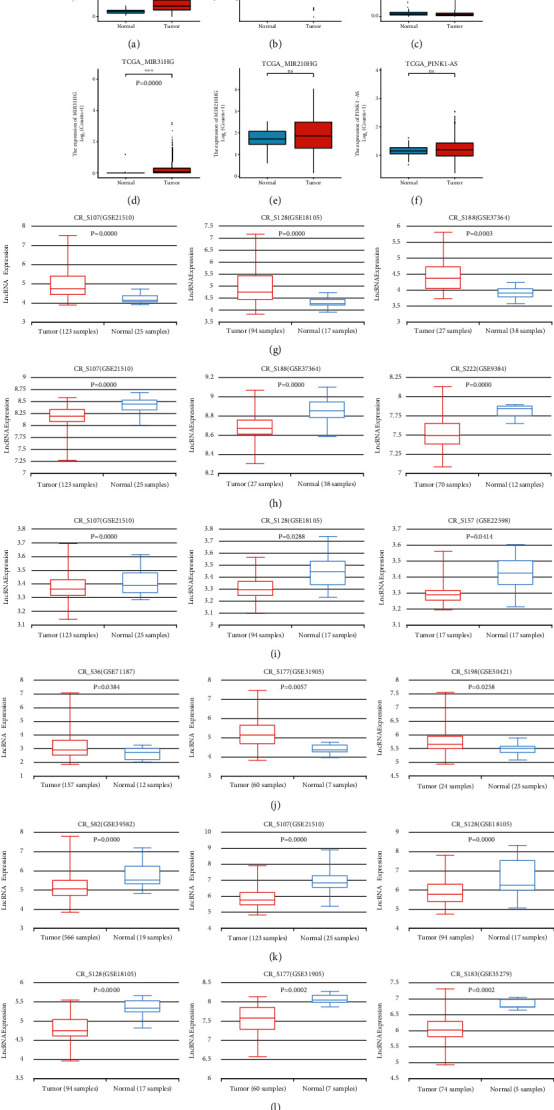
External expression validation for the six ARlncRNAs of the prognostic signature. (a–f) Expression differences of ALMS1-IT1, FGD5-AS1, FLG-AS1, MIR31HG, MIR210HG, and PINK1-AS based on TCGA CRC samples. (g–l) External validation for expression of ALMS1-IT1, FGD5-AS1, FLG-AS1, MIR31HG, MIR210HG, and PINK1-AS based on lnCAR database.

As shown in Figures 10(a)–10(f), the expression differences among ALMS1-IT1, FGD5-AS1, FLG-AS1, and MIR31HG were considered statistically significant (*P* < 0.001) based on expression data retrieved from TCGA. It was noted that the expression of ALMS1-IT1 and MIR31HG in tumor tissues was higher than that in the normal tissues and the expression of FGD5-AS1 and FLG-AS1 in tumor tissues was lower than that in the normal tissues. However, there was no statistical significance between the expression of MIR210HG and PINK1-AS in both tumor and normal tissues based on TCGA. Figures 10(g)–10(l) showed the validation results for the expression of the six ARlncRNAs (ALMS1-IT1, FGD5-AS1, FLG-AS1, MIR31HG, MIR210HG, and PINK1-AS) in the tumor and normal tissues based on lnCAR database. Results of the study revealed that the expression of ALMS1-IT1 and MIR31HG in CR_S107 (GSE21510), CR_S128 (GSE18105), CR_S188 (GSE37364), CR_S36 (GSE71187), CR_S177 (GSE31905), and CR_S198 (GSE50421) was upregulated in the tumor tissues compared with normal tissues with statistical significance (*P* < 0.05) and the expression of FGD5-AS1 and FLG-AS1 in CR_S107 (GSE21510), CR_S188 (GSE37364), CR_S222 (GSE9348), CR_S107 (GSE21510), CR_S128 (GSE18105), and CR_S157 (GSE22598) was downregulated in tumor tissues compared with normal tissues with statistical significance (*P* < 0.05), both of which were consistent with the results based on TCGA. Moreover, lnCAR was used to evidently expound the expression differences between MIR210HG and PINK1-AS in CR_S82 (GSE39582), CR_S107 (GSE21510), CR_S128 (GSE18105), CR_S128 (GSE18105), CR_S177 (GSE31905), and CR_S183 (GSE35279), where the expression of MIR210HG and PINK1-AS was both downregulated in the tumor tissues with statistical significance (*P* < 0.05).

### 3.10. CSC Index and TME Correlation Analysis

**Figure 11:**
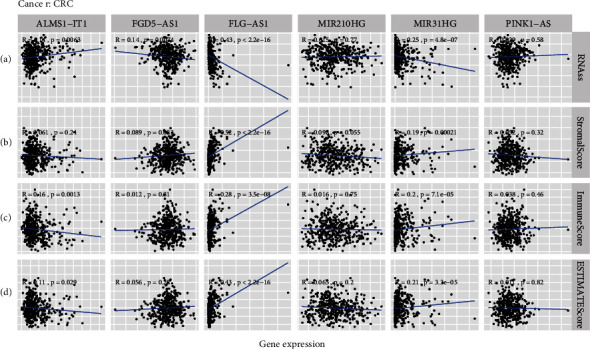
Correlation analysis for expression of six ARlncRNAs and CSCs as well as the TME score. (a–d) The linear correlation between expression of six ARlncRNAs (ALMS1-IT1, FGD5-AS1, FLG-AS1, MIR210HG, MIR31HG, and PINK1-AS) and the CSC index based on RNA-seq as well as the TME score (stromal score, immune score, and ESTIMATE score).

**Figure 12:**
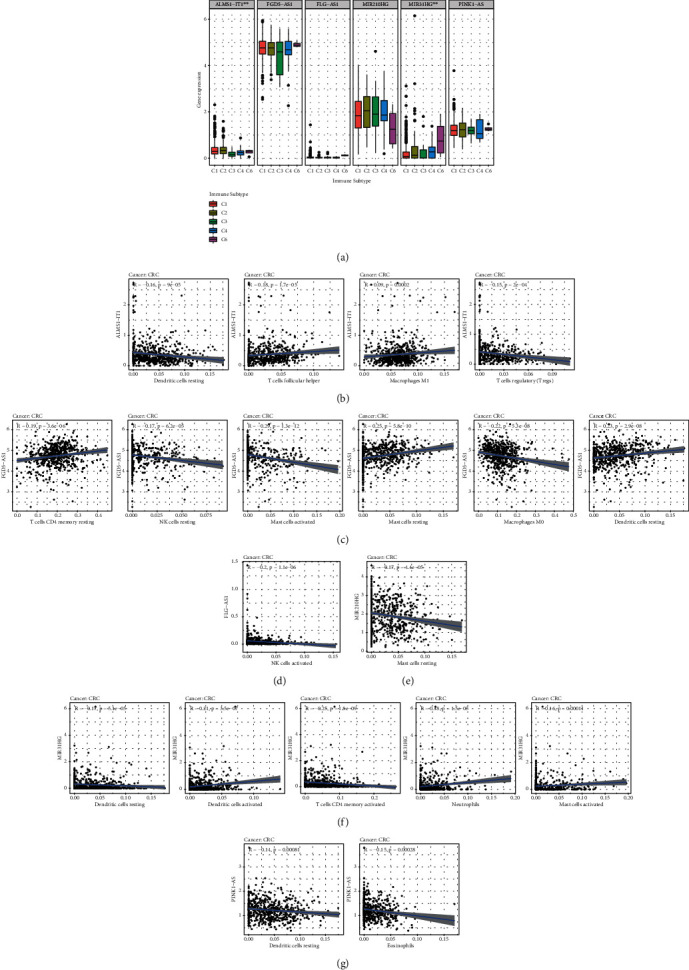
External exploration for expression of six ARlncRNAs and immune components of TME. (a) The statistical differences of the expression of six ARlncRNAs (ALMS1-IT1, FGD5-AS1, FLG-AS1, MIR210HG, MIR31HG, and PINK1-AS) in five immune subtypes (C1, C2, C3, C4, and C6). (b–g) The linear correlation between expression of six ARlncRNAs (ALMS1-IT1, FGD5-AS1, FLG-AS1, MIR210HG, MIR31HG, and PINK1-AS) and relative contents of immunocytes.

CSCs have been proved to be associated with the progression of malignancies and TME was mainly comprised of the stroma microenvironment and immune microenvironment. In this research, the expression of six ARlncRNAs and CSC index as well as the TME score was synthesized to delineate the potential correlation between the ARlncRNAs and CSCs as well as TME in CRC. Figures 11(a)–11(d) manifested the results of the linear correlation between the expression of six ARlncRNAs and the CSC index as well as the TME score (stromal score, immune score, and ESTIMATE score). It was concluded that the expression of ALMS1-IT1 was positively correlated with the CSC index (*R* = 0.14, *P* = 0.0063), indicating that CRC cells with higher expression of ALMS1-IT1 had more distinct stem cell properties and lower degree of cell differentiation. In addition, the expression of FGD5-AS1, FLG-AS1, and MIR31HG was negatively correlated with the CSC index (*R* = −0.14, *P* = 0.0074 and *R* = −0.43, *P* = 2.2*e* − 16 and *R* = −0.25, *P* = 4.8*e* − 07). Moreover, there was no statistical correlation between the expression of MIR210HG and PINK1-AS and the CSC index. For the TME score, higher stromal score, and immune score represented higher relative contents of stromal cells or immunocytes in the tumor microenvironment, and the ESTIMATE score indicated the aggregation of the stromal score and immune score in TME. The expression of FLG-AS1 and MIR31HG was positively correlated with the stromal score (*R* = 0.52, *P* < 2.2*e* − 16 and *R* = 0.19, *P* = 0.00021), immune score (*R* = 0.28, *P* = 3.5*e* − 08 and *R* = 0.2, *P* = 7.1*e* − 05), and ESTIMATE score (*R* = 0.43, *P* < 2.2*e* − 16 and *R* = 0.21, *P* = 3.3*e* − 05). The expression of ALMS1-IT1 was negatively correlated with the immune score (*R* = −0.16, *P* = 0.0013) and ESTIMATE score (*R* = −0.11, *P* = 0.029). In addition, the expression of FGD5-AS1 was positively correlated with the stromal score. However, there was no statistical correlation between the expression of MIR210HG and PINK-AS1 and the stromal score, immune score, or ESTIMATE score. Moreover, we further investigated the correlation between the expression of six ARlncRNAs and the immune environment of CRC to uncover the innate regulatory relationship between ARlncRNA and immune heterogeneity. As shown in Figure 12(a), the expression of ALMS1-IT1 was highest in immune subtype C2 and lowest in the immune subtype C3 with statistical significance (*P* = 0.003). In addition, it was found that the expression of MIR31HG was highest in immune subtype C6 and negligible in the immune subtype C3 with statistical significance (*P* = 0.005). Meanwhile, it was revealed that C5 did not exist in TCGA CRC samples and there were no statistical differences in the expression of FGD5-AS1, FLG-AS1, MIR210HG, and PINK1-AS among C1, C2, C3, C4, and C6. Moreover, results of Spearman correlation analysis for immunocytes obtained from CIBERSORT deconvolution algorithm and six-ARlncRNA expression also showed the relationship between the relative contents of 22 immunocytes and six-ARlncRNA expression, in which “*P* < 0.001” was considered the statistical filtering criteria. The relationships between the expression of ALMS1-IT1, FGD5-AS1, FLG-AS1, MIR210HG, MIR31G, and PINK1-AS with the immunocytes was shown in Figures 12(b)–12(g), respectively.

## 4. Discussion

CRC is a malignant tumor with poor prognosis. Research on the prediction of the prognosis of CRC has always attracted a lot of interest. The currently emerging protein markers, such as CEA and CA199 levels [36, 37] and procalcitonin level [38], could also be used to predict the prognosis for CRC. Moreover, some molecular signatures are also used to predict the CRC prognosis by detecting changes in gene expression of miRNA [39] and TGF-*β* target gene [40] or by gene mutations such as KRAS and BRAF mutations [41]. However, research on using lncRNAs to predict CRC prognosis is on the rise. This study is aimed at exploring the prognostic value of ARlncRNAs on CRC. First, we combined the ARlncRNA expression profiles obtained through coexpression of ARGs with the survival status and survival time of patients retrieved from TCGA, based on which TCGA CRC patients were randomly divided into the training and test groups. Later, the training group was conducted using univariate Cox regression analysis to preliminarily obtain the prognosis-associated ARlncRNAs. Finally, multivariate Cox regression analysis was performed on the prognosis-associated ARlncRNAs after LASSO regression analysis to obtain the six-ARlncRNA (ALMS1-IT1, FGD5-AS1, FLG-AS1, MIR210HG, MIR31HG, and PINK1-AS) prognostic signature. Gao et al. found that downregulation of mir-153-3p or upregulation of CITED2 could reverse the suppressive effects of FGD5-AS1 on the tumor progression and 5-FU chemoresistance [42]. In addition, Li et al. revealed that MIR210HG accelerated the development of breast cancer via the downregulation of the miR-1226-3p/MUC1-C axis [43]. A previous study has illustrated that the high-level expression of MIR31HG was associated with poor prognosis of CRC patients [44], which was indirectly verified by risk stratification in the prognostic signature. The research on the role of ALMS1-IT1, FLG-AS1, and PINK1-AS in the genesis and development of CRC is still in exploration. Moreover, we combined this prognostic signature with the patient clinical feature (age) to further synthetically predict the patients' overall survival with a nomogram. Meanwhile, the biological function of genes in the high- and low-risk groups was hierarchically analyzed based on the six-ARlncRNA prognostic signature with GSEA. The results of GSEA indicated that hypoxia was mostly active in the patients in the high-risk group through a hallmark gene set. Meanwhile, it was found that the metabolism-related pathways, such as peroxisome, the citrate cycle (TCA cycle), and other glycan degradation, were mainly enriched in the CRC patients in the low-risk group. Hypoxia, whose biological functions were mainly mediated by hypoxia-inducible factor-1 (HIF-1) and hypoxia-inducible factor-2 (HIF-2) and their subgroup [45], has expounded the mechanisms in the regulation of tumor vascularization, invasion, and metastasis [46]. For CRC patients, Yu et al. illustrated that the expression level of SIRT1 decreased under hypoxia conditions. This could increase the acetylation of NF-*κ*B, which activated the downstream targets MMP-2/−9 and mediated CRC migration as well as invasion [46]. According to Garzon et al., colon tumor cells with knockdown of HIF-1*α* produced smaller and less hypoxic tumors, as well as increased the functional vascular perfusion system and reduced angiogenic factors [47]. To date, it has been revealed that peroxisome was related to malignant transformation of prostate cancer [48] and progression of hepatic carcinoma [49]. The TCA cycle, the main source of biosynthesis in CRC cells, has demonstrated the central role in the metabolism of CRC cells [50]. Moreover, recent studies have shown that the deficiency of autophagy could exhaust the metabolites of the TCA cycle under the activation of Ras, which was related to the poor prognosis of malignancies [51]. In conclusion, we systemically explored the potential biological processes in CRC genesis and development through stratification for patients in the high- and low-risk groups based on the prognostic signature, which would provide possible evidence for the autophagy-targeted therapy.

Besides, we also constructed the ARlncRNA-DEmiRNA-DEmRNA ceRNA network. At first, Salmena et al. proposed the ceRNA hypothesis, in other words, ceRNA bound with miRNA through the response element to affect the miRNA-induced gene silencing [52]. An increasing number of studies have indicated that lncRNA as a ceRNA played an important regulatory role in the progression of various malignant tumors, including CRC [53]. Notably, we revealed that, the MIR31HG, which was a crucial ARlncRNA in the prognostic signature, mediated two main ceRNA networks, namely, MIR31HG/hsa-mir−193b/PLAU and MIR31HG/hsa-mir−206/STC2. In the ceRNA network MIR31HG/hsa-mir−193b/PLAU, we found that the expression of MIR31HG and PLAU was upregulated, whereas the expression of hsa-mir-193b was downregulated. In the ceRNA network MIR31HG/hsa-mir−206/STC2, the expression trends of MIR31HG and hsa-mir-206 and STC2 were found to be constant with those in the ceRNA network MIR31HG/hsa-mir−193b/PLAU. In the ceRNA network MIR31HG/hsa-mir−193b/PLAU and MIR31HG/hsa-mir−206/STC2, it was hypothesized that the upregulated MIR31HG competitively combined with mir-193b and mir-206, respectively, and inhibited their activation, thus stimulating PLAU and STC2 expression levels to promote the proliferation of CRC cells. In addition, GO and KEGG functional enrichment analysis was also performed on the downstream DEmRNAs to explore potential biological functions. The GO terms, negative regulation of transcription, DNA-templated, heart development, and extracellular space, were deemed statistically significant, in which the negative regulation of transcription, DNA templated, and extracellular space enriched most downstream DEmRNAs. Further, a total of five KEGG pathways, namely, hsa04978: mineral absorption; hsa04550: signaling pathways regulating pluripotency of stem cells; hsa04261: adrenergic signaling in cardiomyocytes; hsa04360: axon guidance; and hsa05205: proteoglycans in cancer, enriched nine downstream DEmRNAs, in which we noticed that ATP2B4 activated both in KEGG pathways, hsa04978: mineral absorption and hsa04261: adrenergic signaling in cardiomyocytes. Zhao et al. have discovered that the downregulated transcription of MDM2 was influenced by oroxylin A to inhibit the degradation of p53 [54]. It was evident that the regulating linkage of the ceRNA network including the downstream DEmRNAs might suggest a clue referring to the correlation between the level of transcription and autophagy. Previous studies have also conducted in-depth exploration on the secretion or transportation of proteins related to the process of autophagy [55, 56] in the extracellular space. It was obvious that the studies for the ceRNA network mediated by ARlncRNAs developed prospects of biological functions in the process of autophagy. Moreover, the absorption of Ca^2+^ was a critical part of the mineral absorption. ATP2B4, as a gene participating the transportation of Ca^2+^, has been confirmed that its overexpression could inhibit the progress and migration of melanoma cells with BRAF mutation [57]. Hence, our studies have presented theoretical significance for the KEGG pathways involved in the downstream DEmRNAs of the ceRNA network in the process of autophagy in CRC.

In this research, we systematically explored the antineoplastic therapy susceptibilities from the aspects of ICI therapy and conventional chemotherapy hierarchically. In the ICI therapy analysis, we noticed that the expression of PDCD1, CTLA4, and HAVCR2 was higher in the high-risk group. Recently, the studies of ICI therapy targeting PD-1 (PDCD1), CTLA-4 (CTLA4), and TIM-3 (HAVCR2) were blooming. The immunotherapies aiming at PD-1 and CTLA-4 have been applied for CRC [56], prostate cancer [57], lung cancer [58], gastric cancer [59], etc. TIM-3 (HAVCR2), which inhibited tumor immunity with depletion of T cells, was a negative regulation immune check point. The ICI therapy for the HAVCR2 has encouraged efficacy in treating advanced non-small cell lung cancer [60], hepatocellular carcinoma [61], etc. TIDE algorithm also revealed that CRC patients in the high-risk group were more sensitive to anti-PD-1 and anti-CTLA4 ICI therapy. Meanwhile, CRC patients with MSI-H statuses were more distributed in the high-risk group. Hu et al. discovered that neoadjuvant toripalimab (the anti-PD-1 monoclonal antibody) could be a potential therapeutic option for CRC patients with the MSI-H status [58]. Therefore, it could be speculated that CRC patients with the MSI-H status in the high-risk group might be more sensitive to the anti-PD-1 ICI therapy. In examining conventional chemotherapy susceptibility based on the prognostic signature, Gefitinib, PLX4720, AZD.2281, cisplatin, and JNK.inhibitor.VIII were all more efficient for CRC patients in the high-risk group compared to CRC patients in the low-risk group. The synergy of gefitinib (inhibitor of EGFR-TK) and inhibition of menin has proven the effect of obstruction of CRC progression [59]. PLX4720 has identified an inhibitor of BRAFV600E kinase to prevent CRC progression [60]. AZD.2281 was initially found hypersensitive on BRCA1-deficient breast and hepatocellular carcinoma cell lines [61]. Furthermore, the latest phase II study suggested that microsatellite-stable CRC cells could not respond to a single application of AZD.2281. Meanwhile, it has been reported that the combination of AZD.2281 and radiation therapy would promote the fatality of CRC cells [62]. Previous studies on the cisplatin have reported that the first generation of platinum anticancer drug was previously widely used in CRC, ovarian cancer, and head and neck cancers, and now, it was more adopted in the intraperitoneal hyperthermic perfusion chemotherapy [63, 64]. Recent studies have suggested that cisplatin presented temperature-dependent efficacy (>41°C) on the apoptosis of CRC cells when using intraperitoneal hyperthermic perfusion chemotherapy [63]. Generally, recent studies on the roles of JNK.inhibitor.VIII in CRC were rare. Obviously, the examination for antineoplastic therapy sensitivity in the high- and low-risk groups provided the propitious indications for applying single or multiple antineoplastic therapies in the fields of individualized treatment.

Cancer stem cells (CSCs) are a cluster of cells that can self-renew and increase heterogeneous tumor cells [65, 66]. Increasing evidence suggested that CSCs were affected by autophagy in the self-renew ability [67, 68]. In this study, we performed Spearman correlation analysis for the CSC index based on RNA-seq and expression of six ARlncRNAs of the prognostic signature to investigate the characteristics of tumor dedifferentiation associated with ARlncRNAs. The expression of ALMS1-IT1 demonstrated its positive correlation with the CSC index with statistical significance, which indicated that high expression of ALMS1-IT1 exhibited the connection with the invasion and chemotherapy resistance of CRC. In contrast with ALMS1-IT1, the expression of FGD5-AS1, FLG-AS1, and MIR31HG was negatively correlated with the CSC index. This might suggest that CRC patients with low expression of FGD5-AS1, FLG-AS1, and MIR31HG were related to the increase of sensitivity of the chemotherapy and weaker proliferation of tumor cells. Moreover, we further explored the correlation between six-ARlncRNA expression and TME. Expression of FLG-AS1 and MIR31HG showed positive relations with the stromal score, immune score, and ESTIMATE score, which proposed that expression of FLG-AS1 and MIR31HG might be involved in the tumor development from the levels of stromal components and immunocytes. In addition, the expression of ALMS1-IT1 was just negatively correlated with the immune score and the ESTIMATE score might indicate that high expression of ALMS1-IT1 inhibits immunoreaction to promote the progression of CRC cells. In the examination of immune subtypes, it was observed that C5 (immunologically quiet) was missing in CRC. Thorsson et al. identified characteristics of tumor immunity, based on which tumors were grouped into six immune subtypes. And C2 (IFN-*γ* dominant) possessed the highest polarities of M1/M2 macrophages, and C6 (TGF-*β* dominant), the smallest immune subtype among six immune subtypes, displayed a significant TGF-*β*-like signature [69]. Referring to the results that the expression of ALMS1-IT1 presented the highest level in C2 (IFN-*γ* dominant) with a statistical difference and the expression of ALMS1-IT1 was positively correlated with the relative contents of M1 macrophages, we could conclude that ALMS1-IT1 affected positively (regulating) the polarities of M1 macrophages of C2 (IFN-*γ* dominant). This also expanded the research scope about the relationships between IFN-*γ* and progression of CRC. The expression of MIR31HG was also in positive regulation with the relative contents of dendritic cells activated. Dendritic cell was an essential kind of antigen-presenting cell, and Hanks et al. have elaborated that TGF-*β* upregulated IDO in dendritic cells to mediate immune escape in tumor models [70]. Therefore, it could be hypothesized that MIR31HG might be involved in the regulation of dendritic cells by TGF-*β*.

In conclusion, this study determined a novel prognostic signature based on six ARlncRNAs (ALMS1-IT1, FGD5-AS1, FLG-AS1, MIR210HG, MIR31HG, and PINK1-AS), which annunciated an encouraging predictive value. The ARlncRNA-mediated ceRNA network further revealed the potential regulatory relationship among various molecules in CRC. GSEA was hierarchically conducted with the hallmark gene set and KEGG pathways to explore specific biological functions for CRC patients in the high- and low-risk groups, which might be of certain guiding significance to individualized treatment for CRC patients in different risk stratification. Furthermore, the antineoplastic therapy susceptibility analysis based on the six-ARlncRNA prognostic signature uncovered a novel perspective for individualized antineoplastic immunotherapy.

## Figures and Tables

**Table 1:** Clinical statistics of TCGA CRC patients.

Characteristics	Variables	Amounts (percentage)
Age	≤65	236 (43.54%)
	>65	306 (56.46%)
Gender	Female	253 (46.68%)
	Male	289 (53.32%)
AJCC-T staging	T1	14 (2.58%)
	T2	95 (17.53%)
	T3	370 (68.27%)
	T4	62 (11.44%)
	Tis	1 (0.18%)
AJCC-N staging	N0	319 (58.86%)
	N1	129 (23.80%)
	N2	93 (17.16%)
	Nx	1 (0.18%)
AJCC-M staging	M0	404 (74.54%)
	M1	75 (13.84%)
	MX	55 (10.15%)
	NA	8 (1.47%)
AJCC stage	Stage I	94 (17.34%)
	Stage II	208 (38.38%)
	Stage III	149 (27.49%)
	Stage IV	76 (14.02%)
	NA	15 (2.77%)

**(a):** 

lncRNA	miRNA	lncRNA	miRNA
AC009093.1	hsa-mir-150	LATS2-AS1	hsa-mir-150
AC020704.1	hsa-mir-150	C15orf54	hsa-mir-150, hsa-mir-206
ST7-OT4	hsa-mir-150	HECW1-IT1	hsa-mir-150
SPATA13-AS1	hsa-mir-193b	AL020995.1	hsa-mir-192
PCA3	hsa-mir-150, hsa-mir-206	RMRP	hsa-mir-206
KCNQ1OT1	hsa-mir-150, hsa-mir-193b, hsa-mir-206	ST7-AS2	hsa-mir-206
FRMD6-AS2	hsa-mir-143, hsa-mir-182, hsa-mir-338	CHL1-AS2	hsa-mir-183
AL139147.1	hsa-mir-150, hsa-mir-206	USP12-AS1	hsa-mir-206
ADAMTS9-AS1	hsa-mir-143, hsa-mir-21, hsa-mir-31	MIR31HG	hsa-mir-193b, hsa-mir-206
MALAT1	hsa-mir-150, hsa-mir-193b, hsa-mir-206	BTBD9-AS1	hsa-mir-150
SFTA1P	hsa-mir-143, hsa-mir-182, hsa-mir-424	PVT1	hsa-mir-150
SMCR5	hsa-mir-150, hsa-mir-193b, hsa-mir-206	RBMS3-AS3	hsa-mir-182
LIFR-AS1	hsa-mir-106a, hsa-mir-182, has-mir-31, hsa-mir-32, hsa-mir-372	JAZF1-AS1	hsa-mir-106a, hsa-mir-143, hsa-mir-17, hsa-mir-21, hsa-mir-32, hsa-mir-372, hsa-mir-98
AC110491.1	hsa-mir-141, hsa-mir-143, hsa-mir-429, hsa-mir-182, hsa-mir-338, hsa-mir-98	ADAMTS9-AS2	hsa-mir-182, hsa-mir-106a, hsa-mir-141, hsa-mir-143, hsa-mir-183, hsa-mir-223
DIRC3	hsa-mir-183, hsa-mir-31, hsa-mir-338, hsa-mir-424, hsa-mir-429, hsa-mir-98		hsa-mir-31, hsa-mir-32, hsa-mir-338, hsa-mir-372, hsa-mir-98

**(b):** 

miRNA	mRNA	miRNA	mRNA
hsa-mir-106a	CADM2, CFL2	hsa-mir-206	STC2
hsa-mir-21	ATP2B4, ELAVL4, OSR1	hsa-mir-223	TCEAL7
hsa-mir-141	CHL1, EDIL3, ELAVL4, EPHA7	hsa-mir-143	BMP3
hsa-mir-150	EREG, FJX1	hsa-mir-17	CYBRD1, HAND1, RBM20, UGP2
hsa-mir-182	FOXF2, PHLPP2, TMEM100	hsa-mir-183	NOVA1
hsa-mir-193b	PLAU	hsa-mir-31	NPTX1
hsa-mir-32	GRIK3, PDCD4	hsa-mir-338	PBLD
hsa-mir-372	EPB41L3, TMEM100, ATP2B4	hsa-mir-98	CADM2, CFL2, NPTX1
hsa-mir-424	CFL2, PDCD4, PHLPP2, TPM2	hsa-mir-429	KLF4

**(a):** 

GO functional annotations			
GO terms	Count	Genes	*P* value
GO:0045892—negative regulation of transcription, DNA-templated	6	TCEAL7, FOXF2, HAND1, PDCD4, KLF4, EREG	0.001
GO:0007507—heart development	3	OSR1, HAND1, RBM20	0.037
GO:0005615—extracellular space	6	BMP3, PLAU, CFL2, STC2, FJX1, EREG	0.045

**(b):** 

KEGG pathway enrichment analysis			
KEGG terms	Count	Genes	*P* value
hsa04978: mineral absorption	2	ATP2B4, CYBRD1	0.005
hsa04550: signaling pathways regulating pluripotency of stem cells	2	KLF4, HAND1	0.025
hsa04261: adrenergic signaling in cardiomyocytes	2	ATP2B4, TPM2	0.027
hsa04360: axon guidance	2	CFL2, EPHA7	0.038
hsa05205: proteoglycans in cancer	2	PLAU, PDCD4	0.048

**Table 4:** GSEA for the high- and low-risk groups.

Names	Size	ES	NES	NOM *P*	FDR *q*
High-risk group					
Hypoxia	197	0.48	1.90	<0.01	0.215
Low-risk group					
Peroxisome	77	−0.58	−2.24	<0.01	0.011
The citrate cycle (TCA cycle)	29	−0.64	−1.97	0.002	0.064
Other glycan degradation	40	−0.66	−1.87	0.002	0.115

## Data Availability

The research data used to support the findings of this study are available from the corresponding author upon request.

## Abbreviations

ARlncRNA: Autophagy-related lncRNA
ARGs: Autophagy-related genes
CRC: Colorectal cancer
DEmRNA: Differentially expressed mRNA
DElncRNA: Differentially expressed lncRNA
DEmiRNA: Differentially expressed miRNA
HADb: The human autophagy database
TCGA: The Cancer Genome Atlas Project
GEO: Gene Expression Omnibus
GO: Gene Ontology
KEGG: Kyoto Encyclopedia of Genes and Genomes
ROC: Receiver operating characteristic
AUC: Area under the curve
FDR: False discovery rate
GSEA: Gene set enrichment analysis
∣logFC∣: ∣logfold change∣
DCA: Decision curve analysis
MSI: Microsatellite instability
ICI: Immune checkpoint inhibitor
TME: Tumor microenvironment
CSC: Cancer stem cell.

## Supplementary Materials

Supplementary Materials Figure S1: Venn diagrams about the target DElncRNAs and target DEmRNAs. Figure S2: the nomogram verified by the test and validation group. Table S1: the summary table of DEmRNA. Table S2: the summary table of DElncRNA. Table S3: the summary table of DEmiRNA. Table S4: grouping statistics for the training, test, and validation groups.
