# Supplementary material for: The Prognostic and Molecular Landscape of Autophagy-Related Long Noncoding RNA in Colorectal Cancer
Source: Biomed Res Int. 2022 Jan 20;2022:5614915. doi: 10.1155/2022/5614915 (PMC8794669; doi:10.1155/2022/5614915)
Supplement: Supplementary Materials — Figure S1: Venn diagrams about the target DElncRNAs and target DEmRNAs. Figure S2: the nomogram verified by the test and validation group. Table S1: the summary table of DEmRNA. Table S2: the summary table of DElncRNA. Table S3: the summary table of DEmiRNA. Table S4: grouping statistics for the training, test, and validation groups. [file 5614915.f1.docx]

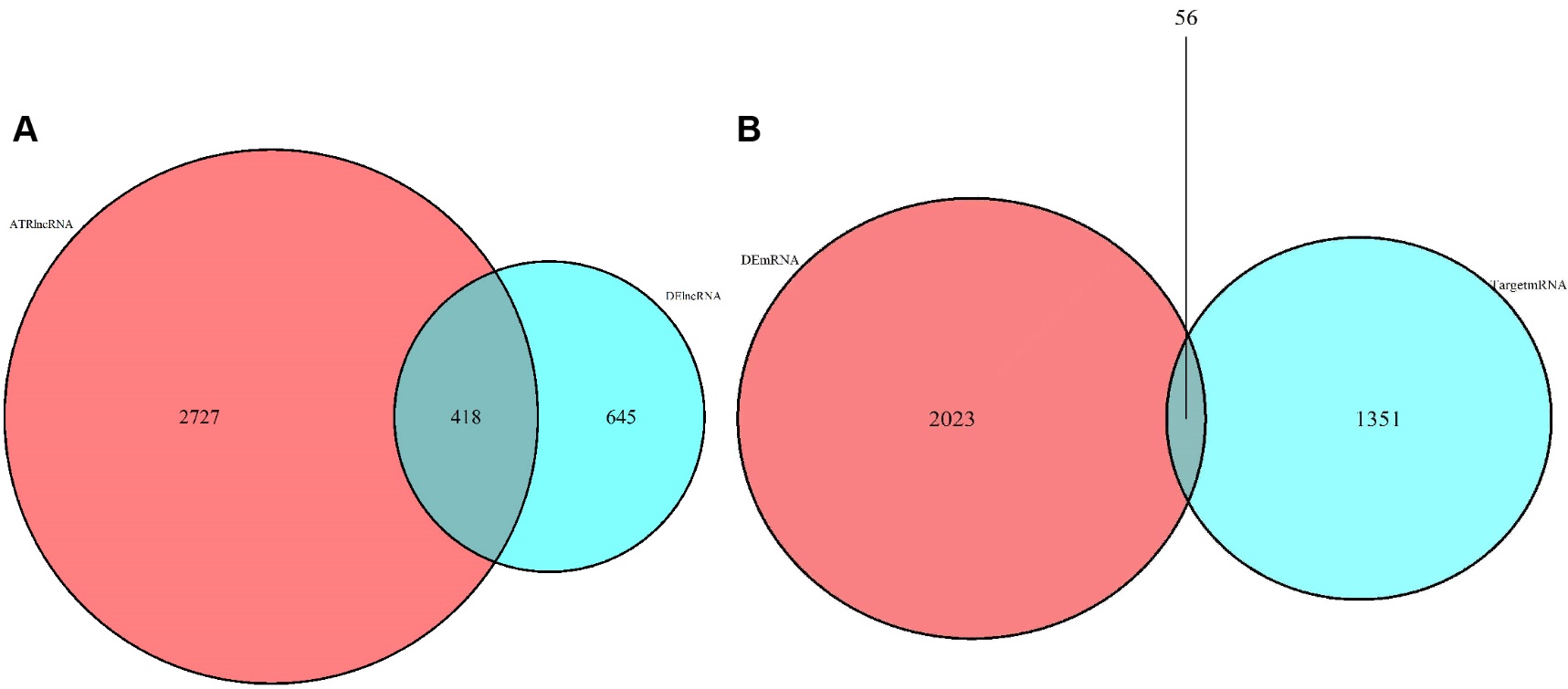


**Figure S1: Venn diagrams anout the target DElncRNAs and target DEmRNAs.** (A): The acquisition of target DElncRNAs. (B): The acquisition of target DEmRNAs.


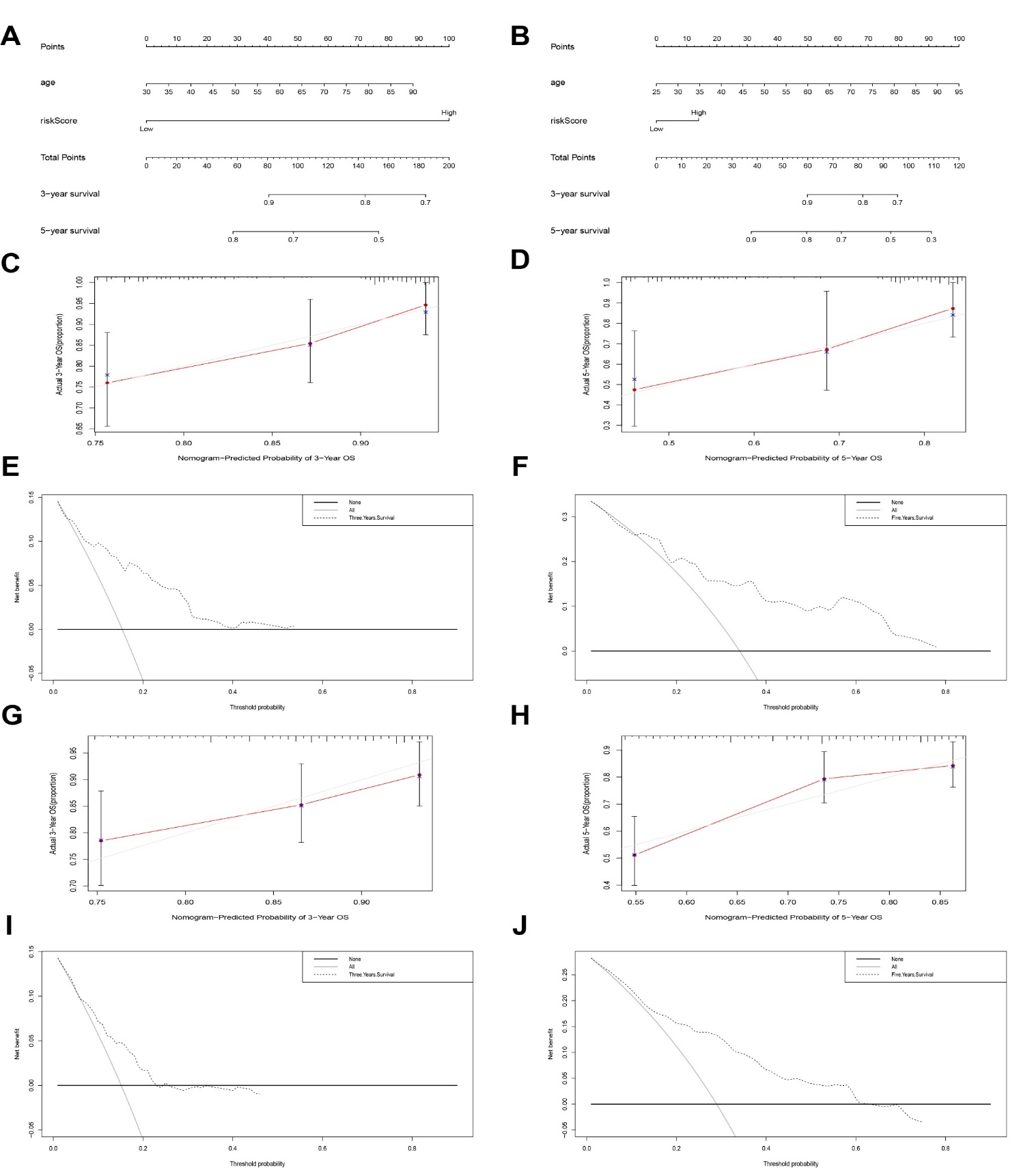
**Figure S2: The nomogram verified by test and validation group**

**(A) and (B):** The nomogram based on independent prognostic factors (Age and ARlncRNA-RS) for the test group and validation group. **(C) and (G):** The 3-year calibration curves for the nomogram in the test group. **(D) and (H):** The 5-year calibration curves for the nomogram in the validation group. **(E) and (I):** The 3-year DCA curves for the nomogram in the test group. **(F) and (J):** The 5-year DCA curves for the nomogram in the validation group.

| **Table S1: The summary table of DEmRNA** | | | | |
| --- | --- | --- | --- | --- |
| **mRNA** | **logFC** | **logCPM** | **P-Value** | **FDR** |
| CDH3 | 5.896806 | 6.642198 | 5.02E-152 | 2.45E-149 |
| ETV4 | 5.386355 | 6.914017 | 8.06E-129 | 2.08E-126 |
| KRT80 | 6.726042 | 5.402403 | 5.12E-124 | 1.19E-121 |
| ESM1 | 5.774344 | 2.906355 | 5.42E-122 | 1.19E-119 |
| MTHFD1L | 2.409154 | 5.713454 | 1.78E-118 | 3.55E-116 |
| AJUBA | 3.005437 | 4.689354 | 1.55E-113 | 2.73E-111 |
| CLDN1 | 4.822122 | 6.632324 | 1.37E-96 | 1.49E-94 |
| NFE2L3 | 2.654052 | 7.107417 | 1.41E-94 | 1.44E-92 |
| FOXQ1 | 6.315455 | 5.486989 | 1.12E-87 | 9.51E-86 |
| CEMIP | 5.245588 | 7.232156 | 7.43E-86 | 5.99E-84 |
| WNT2 | 5.425403 | 3.239267 | 4.87E-83 | 3.54E-81 |
| LRP8 | 3.014605 | 4.636504 | 1.58E-82 | 1.14E-80 |
| TRIP13 | 2.339485 | 4.85844 | 6.39E-81 | 4.41E-79 |
| GRIN2D | 4.903539 | 5.382865 | 7.19E-81 | 4.94E-79 |
| ENC1 | 2.055555 | 7.449748 | 8.93E-81 | 6.09E-79 |
| TRIB3 | 3.887181 | 6.459062 | 5.30E-76 | 3.09E-74 |
| C6orf223 | 4.896214 | 5.326939 | 2.46E-75 | 1.40E-73 |
| SLCO4A1 | 3.476904 | 6.306657 | 3.65E-71 | 1.83E-69 |
| SIM2 | 4.502788 | 4.030199 | 1.27E-70 | 6.33E-69 |
| EPHX4 | 4.513258 | 2.978059 | 1.88E-70 | 9.22E-69 |
| GTF2IRD1 | 2.209347 | 6.274051 | 1.01E-68 | 4.70E-67 |
| IQANK1 | 2.886064 | 5.242907 | 2.18E-67 | 9.62E-66 |
| KIAA1549 | 2.32258 | 4.87591 | 3.73E-67 | 1.63E-65 |
| SPTBN2 | 3.100581 | 5.377799 | 3.43E-66 | 1.43E-64 |
| PPM1H | 2.300043 | 5.612196 | 1.43E-65 | 5.87E-64 |
| DDN | 4.659202 | 1.85448 | 3.30E-63 | 1.27E-61 |
| FUT1 | 2.859799 | 3.254136 | 1.72E-62 | 6.50E-61 |
| OTX1 | 5.66828 | 1.670687 | 4.74E-61 | 1.70E-59 |
| ORC6 | 2.030221 | 4.077399 | 5.36E-61 | 1.91E-59 |
| TEAD4 | 2.317634 | 4.965878 | 5.37E-61 | 1.91E-59 |
| CPNE7 | 5.733498 | 5.07024 | 7.75E-61 | 2.73E-59 |
| BLACAT1 | 5.355354 | 2.585416 | 7.75E-61 | 2.73E-59 |
| MDFI | 3.635056 | 3.96874 | 1.32E-60 | 4.59E-59 |
| PLEKHN1 | 3.447067 | 2.219535 | 2.24E-59 | 7.56E-58 |
| PDX1 | 4.767917 | 4.301579 | 2.95E-59 | 9.87E-58 |
| AUNIP | 2.256072 | 2.561814 | 7.82E-59 | 2.59E-57 |
| NEBL | 2.3772 | 6.650745 | 2.17E-58 | 7.12E-57 |
| CBX2 | 3.647594 | 4.081817 | 4.19E-57 | 1.31E-55 |
| INHBA | 5.372686 | 5.409375 | 6.51E-57 | 2.02E-55 |
| KIAA1257 | 3.453193 | 2.289158 | 7.94E-57 | 2.45E-55 |
| CGREF1 | 3.527702 | 4.836098 | 1.06E-56 | 3.25E-55 |
| LARGE2 | 4.497393 | 5.472394 | 4.43E-56 | 1.32E-54 |
| SLC7A5 | 3.061737 | 7.721593 | 4.47E-56 | 1.34E-54 |
| SPATA12 | 3.409531 | 0.262607 | 1.01E-55 | 2.98E-54 |
| ZC3HAV1L | 2.168317 | 3.065669 | 2.02E-55 | 5.90E-54 |
| PRSS22 | 3.936843 | 4.716433 | 2.11E-55 | 6.16E-54 |
| EPOP | 3.798948 | 4.07563 | 2.72E-55 | 7.87E-54 |
| SALL4 | 4.83784 | 2.007996 | 6.31E-54 | 1.72E-52 |
| SH3TC2 | 3.384736 | 3.574055 | 9.48E-54 | 2.57E-52 |
| ANLN | 2.141717 | 5.912269 | 1.73E-53 | 4.62E-52 |
| HILPDA | 2.394895 | 5.085906 | 4.49E-53 | 1.20E-51 |
| XRCC2 | 2.160012 | 3.279695 | 1.96E-52 | 5.06E-51 |
| SLC6A6 | 2.808043 | 6.980939 | 5.05E-52 | 1.29E-50 |
| SKA3 | 2.134836 | 4.77719 | 2.12E-51 | 5.35E-50 |
| TMEM132A | 2.55497 | 5.443469 | 4.61E-50 | 1.12E-48 |
| COL11A1 | 6.520142 | 5.422305 | 6.90E-50 | 1.66E-48 |
| CDC25B | 2.218051 | 7.445371 | 7.53E-50 | 1.80E-48 |
| FJX1 | 2.893912 | 2.433165 | 8.20E-50 | 1.96E-48 |
| RHPN1 | 3.351929 | 4.928654 | 1.11E-49 | 2.63E-48 |
| MCIDAS | 3.957037 | 0.252882 | 1.46E-49 | 3.45E-48 |
| UBE2C | 2.185023 | 6.527424 | 2.10E-49 | 4.95E-48 |
| EVA1A | 3.767232 | 3.264012 | 4.10E-49 | 9.57E-48 |
| VWA2 | 3.94154 | 4.636569 | 4.45E-49 | 1.04E-47 |
| APLN | 3.190763 | 3.34416 | 9.79E-47 | 2.07E-45 |
| CST1 | 8.446928 | 4.897225 | 1.10E-46 | 2.32E-45 |
| TGFBI | 3.236035 | 9.939867 | 3.03E-46 | 6.30E-45 |
| COL10A1 | 7.830557 | 4.749814 | 1.58E-45 | 3.21E-44 |
| TRIM29 | 4.273523 | 6.567342 | 2.31E-45 | 4.67E-44 |
| TNFRSF12A | 2.32348 | 5.884159 | 3.32E-45 | 6.65E-44 |
| CBY2 | 7.087658 | -0.11136 | 3.74E-45 | 7.45E-44 |
| CCNO | 3.010381 | 3.429978 | 1.22E-44 | 2.40E-43 |
| TESC | 4.5857 | 5.880523 | 2.17E-44 | 4.23E-43 |
| IGFL4 | 5.590463 | 1.321838 | 4.02E-44 | 7.79E-43 |
| SNTB1 | 2.248701 | 6.035682 | 5.12E-44 | 9.90E-43 |
| FAM166C | 3.39535 | 2.460548 | 1.12E-43 | 2.13E-42 |
| KLK6 | 9.518106 | 4.874579 | 1.26E-43 | 2.41E-42 |
| ATP11A | 2.166114 | 6.633469 | 1.34E-43 | 2.55E-42 |
| EGFL6 | 3.511925 | 1.954767 | 1.67E-43 | 3.18E-42 |
| CNTD2 | 4.84912 | 2.508849 | 1.68E-43 | 3.19E-42 |
| PSAT1 | 2.737527 | 5.979112 | 3.61E-43 | 6.77E-42 |
| SMOX | 2.33489 | 4.729824 | 3.90E-43 | 7.30E-42 |
| MMP7 | 7.098882 | 6.116352 | 6.10E-43 | 1.13E-41 |
| ARNTL2 | 2.397152 | 4.843307 | 7.92E-43 | 1.47E-41 |
| MSX2 | 4.754459 | 3.904732 | 8.55E-43 | 1.58E-41 |
| LEMD1 | 6.023073 | 2.069695 | 1.10E-42 | 2.03E-41 |
| PRR7 | 3.650419 | 3.647425 | 1.20E-42 | 2.20E-41 |
| ACAN | 3.130615 | 3.371253 | 5.63E-42 | 1.00E-40 |
| ANKRD13B | 2.401273 | 4.246262 | 9.93E-42 | 1.75E-40 |
| MMP11 | 4.370696 | 6.946575 | 1.00E-41 | 1.76E-40 |
| PERM1 | 3.075981 | 0.751884 | 5.49E-41 | 9.37E-40 |
| LRRC8E | 2.435177 | 0.256707 | 6.04E-41 | 1.03E-39 |
| MAPK15 | 4.344037 | 2.863066 | 6.46E-41 | 1.10E-39 |
| NKD2 | 4.354747 | 4.77384 | 9.34E-41 | 1.58E-39 |
| ATG9B | 3.494762 | 2.627579 | 1.05E-40 | 1.78E-39 |
| ADAMTS12 | 3.411037 | 4.519212 | 2.27E-40 | 3.81E-39 |
| SMKR1 | 4.06202 | 0.978203 | 2.40E-40 | 4.03E-39 |
| SAPCD2 | 2.095852 | 6.317574 | 2.62E-40 | 4.39E-39 |
| GABRD | 2.886035 | 0.692637 | 6.49E-40 | 1.07E-38 |
| STRA6 | 5.744405 | 3.790281 | 9.38E-40 | 1.53E-38 |
| DBNDD1 | 2.471889 | 4.687235 | 1.60E-39 | 2.59E-38 |
| POU5F1B | 4.619871 | 3.122637 | 2.20E-39 | 3.54E-38 |
| FXYD5 | 2.107286 | 6.957992 | 3.98E-39 | 6.33E-38 |
| FBXO41 | 2.037391 | 4.513219 | 5.40E-39 | 8.53E-38 |
| CTHRC1 | 3.787624 | 4.99052 | 6.97E-39 | 1.09E-37 |
| WDR66 | 2.66342 | 1.687818 | 2.10E-38 | 3.22E-37 |
| C2CD4A | 4.264836 | 4.745116 | 2.20E-38 | 3.36E-37 |
| GRHL3 | 3.879899 | 2.526081 | 2.77E-38 | 4.22E-37 |
| KRT23 | 7.351488 | 6.238429 | 4.68E-38 | 7.05E-37 |
| KLC3 | 5.966579 | 0.291032 | 6.09E-38 | 9.13E-37 |
| STPG4 | 4.869458 | 0.50823 | 7.05E-38 | 1.05E-36 |
| TNS4 | 3.381494 | 7.126939 | 7.72E-38 | 1.15E-36 |
| BTBD16 | 3.576515 | 0.438731 | 8.65E-38 | 1.28E-36 |
| CELSR3 | 2.720357 | 4.635796 | 9.33E-38 | 1.38E-36 |
| UCN2 | 4.551108 | -0.55781 | 1.09E-37 | 1.61E-36 |
| HAGHL | 3.558853 | 3.625961 | 1.23E-37 | 1.81E-36 |
| KLHL35 | 3.904207 | 2.867554 | 1.37E-37 | 2.00E-36 |
| SFTA2 | 7.322948 | 1.731188 | 1.39E-37 | 2.04E-36 |
| MACC1 | 2.273372 | 6.186839 | 1.47E-37 | 2.15E-36 |
| CELSR1 | 2.349164 | 5.492406 | 1.79E-37 | 2.61E-36 |
| ULBP1 | 3.949543 | 0.01683 | 2.02E-37 | 2.93E-36 |
| PABPC1L | 3.06641 | 4.882885 | 2.22E-37 | 3.23E-36 |
| ASCL2 | 4.004905 | 6.967883 | 3.92E-37 | 5.64E-36 |
| CST2 | 6.074087 | 0.86226 | 4.96E-37 | 7.10E-36 |
| SCD | 2.310274 | 9.074708 | 5.04E-37 | 7.21E-36 |
| WNT3 | 2.419979 | 1.933092 | 1.35E-36 | 1.88E-35 |
| SLC4A11 | 4.118423 | 3.240707 | 1.65E-36 | 2.30E-35 |
| PRDM12 | 2.839785 | -1.40746 | 2.89E-36 | 3.99E-35 |
| STRIP2 | 2.496653 | 3.012711 | 3.17E-36 | 4.36E-35 |
| MEX3A | 2.879188 | 4.080365 | 4.25E-36 | 5.81E-35 |
| CCDC78 | 3.611625 | 1.952345 | 6.62E-36 | 8.94E-35 |
| GDF15 | 3.062631 | 7.642787 | 6.67E-36 | 9.01E-35 |
| RNF183 | 3.255349 | 2.446609 | 7.17E-36 | 9.65E-35 |
| OXTR | 2.815501 | 1.304414 | 8.80E-36 | 1.18E-34 |
| RECQL4 | 2.269645 | 5.617101 | 1.32E-35 | 1.76E-34 |
| GLS2 | 2.568151 | 0.29079 | 2.02E-35 | 2.65E-34 |
| GAD1 | 5.395717 | 1.938572 | 3.27E-35 | 4.25E-34 |
| TLX1 | 4.043004 | 2.036753 | 3.52E-35 | 4.58E-34 |
| PRR36 | 4.06298 | 3.235889 | 3.75E-35 | 4.85E-34 |
| RNF43 | 2.2904 | 8.17844 | 4.07E-35 | 5.26E-34 |
| GRHL1 | 2.327795 | 2.690696 | 4.54E-35 | 5.85E-34 |
| NOTUM | 9.15167 | 6.380503 | 9.29E-35 | 1.18E-33 |
| FAM227A | 2.414906 | 1.366183 | 1.06E-34 | 1.34E-33 |
| GRIN2B | 3.486826 | 2.986011 | 1.06E-34 | 1.34E-33 |
| STC2 | 3.676087 | 4.896541 | 2.81E-34 | 3.49E-33 |
| PHLDA1 | 2.509399 | 7.348446 | 5.15E-34 | 6.30E-33 |
| MYBL2 | 2.146614 | 6.933756 | 5.23E-34 | 6.39E-33 |
| COL7A1 | 3.175315 | 5.313508 | 1.56E-33 | 1.87E-32 |
| FEZF1 | 8.24267 | 1.375909 | 2.79E-33 | 3.30E-32 |
| MSX1 | 3.399518 | 2.979371 | 3.29E-33 | 3.89E-32 |
| IRX5 | 4.534552 | 0.458381 | 3.53E-33 | 4.17E-32 |
| NPFFR1 | 3.419854 | 2.067583 | 1.08E-32 | 1.24E-31 |
| ULBP3 | 2.04428 | 0.742703 | 1.57E-32 | 1.81E-31 |
| VSNL1 | 2.778192 | 4.98876 | 2.20E-32 | 2.51E-31 |
| RNFT2 | 2.005814 | 2.201961 | 3.41E-32 | 3.84E-31 |
| TG | 4.287732 | 3.442139 | 5.52E-32 | 6.19E-31 |
| STK31 | 3.799847 | 1.872642 | 5.67E-32 | 6.36E-31 |
| RGS16 | 2.164929 | 4.321636 | 5.85E-32 | 6.55E-31 |
| BRCA2 | 2.08425 | 4.214958 | 5.97E-32 | 6.67E-31 |
| ULBP2 | 4.27603 | 1.190265 | 8.03E-32 | 8.95E-31 |
| SULT2B1 | 2.98918 | 4.80193 | 1.38E-31 | 1.52E-30 |
| CLDN2 | 5.41181 | 7.37342 | 1.45E-31 | 1.59E-30 |
| HOMER1 | 2.043239 | 3.273619 | 1.46E-31 | 1.60E-30 |
| DMBX1 | 5.985545 | -0.04124 | 2.01E-31 | 2.19E-30 |
| AZGP1 | 3.119783 | 6.270756 | 2.13E-31 | 2.32E-30 |
| KLK8 | 8.437143 | 1.519165 | 2.76E-31 | 2.98E-30 |
| SHISA2 | 3.092968 | 2.053325 | 8.32E-31 | 8.78E-30 |
| ADAM12 | 4.14808 | 4.608404 | 9.89E-31 | 1.04E-29 |
| GJB4 | 3.98541 | 2.159904 | 1.08E-30 | 1.13E-29 |
| SERPINA4 | 6.476594 | 0.605637 | 1.20E-30 | 1.25E-29 |
| GDPD5 | 2.757312 | 5.31427 | 1.48E-30 | 1.55E-29 |
| ASCL5 | 4.11758 | 0.836134 | 1.76E-30 | 1.83E-29 |
| CCDC150 | 2.098829 | 1.63955 | 1.96E-30 | 2.02E-29 |
| TFR2 | 2.808299 | 2.399617 | 5.17E-30 | 5.25E-29 |
| SLC7A11 | 2.716161 | 4.752086 | 6.41E-30 | 6.49E-29 |
| PLEKHG4 | 2.027742 | 5.502176 | 6.82E-30 | 6.89E-29 |
| ZNF695 | 2.17274 | 0.94393 | 6.95E-30 | 7.01E-29 |
| KLK7 | 8.321878 | 2.800665 | 8.96E-30 | 9.00E-29 |
| FANCB | 2.016458 | 1.347798 | 1.02E-29 | 1.02E-28 |
| IL11 | 4.457776 | 2.307111 | 1.40E-29 | 1.40E-28 |
| MMP3 | 4.775019 | 5.637583 | 1.46E-29 | 1.45E-28 |
| CST4 | 6.350881 | -0.78255 | 1.69E-29 | 1.68E-28 |
| SP5 | 3.722727 | 3.932485 | 1.79E-29 | 1.78E-28 |
| LMTK3 | 2.112802 | 3.029173 | 3.03E-29 | 2.97E-28 |
| MLXIPL | 2.841015 | 5.508632 | 3.72E-29 | 3.63E-28 |
| CA9 | 5.551164 | 5.658977 | 5.64E-29 | 5.47E-28 |
| PMAIP1 | 2.060063 | 3.890421 | 5.81E-29 | 5.64E-28 |
| KLK10 | 5.066419 | 5.736009 | 9.34E-29 | 8.95E-28 |
| CORIN | 3.354002 | 1.359661 | 9.43E-29 | 9.03E-28 |
| LY6G6E | 5.424318 | -1.37131 | 1.39E-28 | 1.32E-27 |
| DSG4 | 2.476643 | 0.319017 | 1.66E-28 | 1.57E-27 |
| NKPD1 | 3.239545 | -0.10198 | 2.14E-28 | 2.00E-27 |
| TEX45 | 2.392097 | 2.124799 | 2.25E-28 | 2.11E-27 |
| COMP | 5.979372 | 4.479064 | 3.16E-28 | 2.93E-27 |
| CXCL3 | 2.863097 | 5.630776 | 4.10E-28 | 3.79E-27 |
| KIF26B | 3.218031 | 3.932359 | 5.25E-28 | 4.82E-27 |
| S100A2 | 4.594198 | 3.748327 | 6.37E-28 | 5.83E-27 |
| PALD1 | 2.001426 | 5.627897 | 6.99E-28 | 6.39E-27 |
| LZTS3 | 2.208681 | 5.425077 | 7.19E-28 | 6.56E-27 |
| RAET1L | 3.889217 | 0.607189 | 7.20E-28 | 6.57E-27 |
| CYSRT1 | 2.628454 | 0.580289 | 1.17E-27 | 1.06E-26 |
| PGGHG | 3.126443 | 6.452669 | 1.62E-27 | 1.45E-26 |
| IBSP | 6.417533 | 0.435458 | 1.65E-27 | 1.48E-26 |
| CARD14 | 2.245095 | 2.321086 | 1.86E-27 | 1.66E-26 |
| ATP6V1C2 | 2.382597 | 3.224732 | 3.29E-27 | 2.91E-26 |
| SHH | 2.126524 | 4.839091 | 3.54E-27 | 3.12E-26 |
| CCN4 | 2.632581 | 3.094941 | 3.81E-27 | 3.36E-26 |
| ATP6V1FNB | 2.226699 | 2.330519 | 3.83E-27 | 3.38E-26 |
| SRPX2 | 3.028084 | 5.033815 | 4.17E-27 | 3.66E-26 |
| APELA | 3.15464 | -1.30388 | 4.67E-27 | 4.09E-26 |
| LRRC36 | 3.288653 | 1.932405 | 4.84E-27 | 4.23E-26 |
| SLC22A3 | 2.025471 | 4.552246 | 6.42E-27 | 5.57E-26 |
| MMP1 | 4.452143 | 6.907599 | 8.12E-27 | 7.02E-26 |
| TNNI3 | 4.749986 | 0.844549 | 9.77E-27 | 8.42E-26 |
| WNT7B | 5.976756 | 1.074195 | 1.01E-26 | 8.66E-26 |
| PPEF1 | 2.92391 | -0.98441 | 1.19E-26 | 1.02E-25 |
| SOX14 | 7.274212 | 0.881635 | 1.25E-26 | 1.07E-25 |
| NKD1 | 4.170653 | 6.678226 | 1.38E-26 | 1.18E-25 |
| ASPHD1 | 2.3798 | 3.258984 | 1.48E-26 | 1.26E-25 |
| PLAU | 2.056327 | 6.196302 | 1.63E-26 | 1.38E-25 |
| LY6G6F | 5.210559 | -1.62919 | 1.67E-26 | 1.41E-25 |
| AXIN2 | 2.478911 | 7.311365 | 1.68E-26 | 1.42E-25 |
| TACSTD2 | 4.804611 | 5.418237 | 1.74E-26 | 1.48E-25 |
| XKRX | 2.469806 | 2.81943 | 2.42E-26 | 2.04E-25 |
| GPR3 | 2.317976 | 0.796673 | 3.50E-26 | 2.93E-25 |
| ANOS1 | 3.003417 | 3.213449 | 5.63E-26 | 4.65E-25 |
| CFAP45 | 2.166309 | 0.127872 | 7.29E-26 | 5.99E-25 |
| PCSK9 | 2.745277 | 6.212044 | 8.45E-26 | 6.91E-25 |
| SPTBN5 | 3.117205 | 3.017888 | 9.03E-26 | 7.38E-25 |
| KRT75 | 6.928701 | 0.953633 | 1.14E-25 | 9.26E-25 |
| C5orf46 | 5.866031 | -0.60193 | 1.15E-25 | 9.35E-25 |
| DNAH2 | 2.893093 | 2.545816 | 1.16E-25 | 9.42E-25 |
| TCN1 | 7.207169 | 4.699515 | 1.32E-25 | 1.07E-24 |
| SHROOM4 | 2.004999 | 4.800831 | 1.35E-25 | 1.09E-24 |
| DKK4 | 8.418061 | 2.670061 | 1.50E-25 | 1.21E-24 |
| ALKAL1 | 3.552276 | 0.947093 | 1.94E-25 | 1.55E-24 |
| CXCL1 | 2.80416 | 6.410274 | 2.52E-25 | 2.02E-24 |
| C20orf144 | 2.194546 | -1.03451 | 2.54E-25 | 2.03E-24 |
| PKP1 | 3.990566 | 3.751583 | 2.70E-25 | 2.16E-24 |
| MROH6 | 2.297608 | 5.193448 | 2.89E-25 | 2.30E-24 |
| LEF1 | 2.133373 | 3.975943 | 2.98E-25 | 2.37E-24 |
| C8orf74 | 6.080865 | -1.64313 | 3.23E-25 | 2.56E-24 |
| SLCO1B3 | 6.396556 | 2.11723 | 3.26E-25 | 2.58E-24 |
| AMELX | 4.982711 | -1.26161 | 3.87E-25 | 3.05E-24 |
| MFAP2 | 2.360629 | 4.32758 | 3.92E-25 | 3.09E-24 |
| TCF24 | 4.083667 | -1.65314 | 4.20E-25 | 3.30E-24 |
| TMPRSS13 | 3.30468 | 3.531749 | 6.43E-25 | 4.99E-24 |
| FGFRL1 | 2.41465 | 6.649679 | 6.69E-25 | 5.19E-24 |
| MYEOV | 3.018934 | 5.072085 | 7.84E-25 | 6.05E-24 |
| AKR1C4 | 5.602388 | 0.567039 | 8.76E-25 | 6.75E-24 |
| KRT17 | 5.167842 | 5.070945 | 1.31E-24 | 1.01E-23 |
| GBX2 | 5.510066 | -1.25387 | 1.73E-24 | 1.31E-23 |
| TDGF1 | 3.077629 | 4.866331 | 1.82E-24 | 1.39E-23 |
| FAP | 3.213609 | 3.775278 | 1.93E-24 | 1.46E-23 |
| TMEM74B | 2.954732 | 2.97092 | 2.22E-24 | 1.68E-23 |
| ARID3A | 2.185517 | 5.698285 | 2.74E-24 | 2.06E-23 |
| DHRS2 | 4.605834 | 2.379467 | 2.77E-24 | 2.08E-23 |
| CEL | 5.945769 | 5.34167 | 2.84E-24 | 2.13E-23 |
| DLX4 | 2.395475 | -0.08954 | 3.13E-24 | 2.34E-23 |
| NOX4 | 3.066748 | 1.45735 | 3.19E-24 | 2.38E-23 |
| KCNH8 | 2.562585 | 2.457395 | 3.40E-24 | 2.54E-23 |
| UCN | 2.272637 | -0.04563 | 3.51E-24 | 2.61E-23 |
| SMCO2 | 2.560334 | -1.72514 | 3.58E-24 | 2.66E-23 |
| IL23A | 2.882461 | 1.770611 | 3.88E-24 | 2.89E-23 |
| CLEC5A | 3.52094 | 1.786964 | 6.22E-24 | 4.58E-23 |
| CLDN16 | 2.953419 | 0.052908 | 1.08E-23 | 7.84E-23 |
| SLC17A9 | 2.416139 | 5.857234 | 1.34E-23 | 9.71E-23 |
| ACBD7 | 2.124116 | 0.253734 | 1.48E-23 | 1.07E-22 |
| MMP13 | 5.449558 | 1.216464 | 1.68E-23 | 1.21E-22 |
| TNFSF9 | 3.44013 | 3.332257 | 1.68E-23 | 1.21E-22 |
| ACSL6 | 4.341076 | 4.651025 | 2.53E-23 | 1.81E-22 |
| S100P | 2.399441 | 7.980729 | 3.22E-23 | 2.29E-22 |
| CACNG8 | 3.541858 | 0.280151 | 3.64E-23 | 2.58E-22 |
| BGN | 2.514451 | 8.068706 | 3.87E-23 | 2.74E-22 |
| DNASE1L2 | 2.496088 | 0.372192 | 4.08E-23 | 2.89E-22 |
| SPOCD1 | 2.229997 | 1.59198 | 4.77E-23 | 3.37E-22 |
| ZIC5 | 8.093734 | 1.615209 | 5.13E-23 | 3.62E-22 |
| GOLGA7B | 2.457953 | 3.283984 | 5.21E-23 | 3.67E-22 |
| ASB9 | 2.179653 | 3.17505 | 5.81E-23 | 4.07E-22 |
| GABRE | 2.342354 | 5.202126 | 7.15E-23 | 4.98E-22 |
| OLR1 | 3.714205 | 2.641496 | 9.52E-23 | 6.58E-22 |
| TMEM249 | 3.892946 | -0.27647 | 1.16E-22 | 7.97E-22 |
| ZIC2 | 6.126415 | 2.868832 | 1.46E-22 | 9.97E-22 |
| TMPRSS3 | 2.977377 | 3.989253 | 1.74E-22 | 1.18E-21 |
| UNC5CL | 2.010849 | 4.107392 | 1.78E-22 | 1.21E-21 |
| PAH | 5.515616 | 2.030691 | 2.36E-22 | 1.59E-21 |
| NANOS3 | 3.828083 | 1.094 | 2.39E-22 | 1.61E-21 |
| DUSP27 | 7.706683 | 5.641041 | 2.51E-22 | 1.68E-21 |
| NXPH4 | 5.643252 | 3.064638 | 2.62E-22 | 1.76E-21 |
| SERPINB5 | 3.516401 | 6.18981 | 2.67E-22 | 1.79E-21 |
| WT1 | 4.81436 | 1.201103 | 3.33E-22 | 2.22E-21 |
| ERFE | 3.182571 | 1.426386 | 3.39E-22 | 2.26E-21 |
| RELL2 | 2.071051 | 2.006108 | 4.13E-22 | 2.73E-21 |
| GSDMC | 2.962705 | -1.02485 | 5.48E-22 | 3.61E-21 |
| PTP4A3 | 2.334967 | 6.149628 | 6.50E-22 | 4.26E-21 |
| COL1A1 | 2.641152 | 11.39971 | 6.79E-22 | 4.45E-21 |
| C6orf15 | 7.449671 | 1.734762 | 8.18E-22 | 5.33E-21 |
| LGR5 | 2.868819 | 6.366169 | 8.44E-22 | 5.50E-21 |
| EPYC | 6.997238 | 1.018628 | 1.38E-21 | 8.89E-21 |
| ADAMTS2 | 2.350338 | 5.629641 | 1.44E-21 | 9.29E-21 |
| SLAMF9 | 3.628827 | -0.72948 | 1.53E-21 | 9.88E-21 |
| SIX1 | 3.507737 | -0.01513 | 1.56E-21 | 1.00E-20 |
| TFAP2A | 3.344495 | 2.832775 | 1.73E-21 | 1.11E-20 |
| NEK5 | 2.189555 | 1.82363 | 1.87E-21 | 1.20E-20 |
| TMEM270 | 2.513234 | -1.3554 | 1.97E-21 | 1.26E-20 |
| DUSP4 | 3.298701 | 5.715854 | 2.25E-21 | 1.44E-20 |
| SLC22A11 | 4.576594 | 1.647346 | 2.55E-21 | 1.62E-20 |
| RFX8 | 2.758037 | -1.60441 | 2.75E-21 | 1.75E-20 |
| CHI3L1 | 2.791849 | 5.020368 | 2.83E-21 | 1.79E-20 |
| PTK7 | 2.053033 | 6.700734 | 6.24E-21 | 3.87E-20 |
| SLC35D3 | 4.358394 | 2.827482 | 9.05E-21 | 5.55E-20 |
| LY6G6F-LY6G6D | 5.814301 | 3.36484 | 9.96E-21 | 6.10E-20 |
| AMH | 4.131607 | 2.034709 | 1.03E-20 | 6.28E-20 |
| RIPPLY3 | 3.672839 | 0.547875 | 1.27E-20 | 7.75E-20 |
| FNDC11 | 2.557504 | 0.398453 | 1.33E-20 | 8.08E-20 |
| LPO | 4.688537 | -0.08888 | 1.47E-20 | 8.95E-20 |
| PAX9 | 2.99247 | 1.326098 | 1.67E-20 | 1.01E-19 |
| KRT6B | 5.456671 | 4.094591 | 2.50E-20 | 1.50E-19 |
| STC1 | 2.004062 | 3.975052 | 2.54E-20 | 1.52E-19 |
| GJC3 | 2.581053 | -0.48314 | 3.48E-20 | 2.08E-19 |
| EN2 | 6.103206 | 1.078371 | 3.57E-20 | 2.13E-19 |
| IGFL1 | 8.159895 | 0.297147 | 3.61E-20 | 2.15E-19 |
| HS6ST2 | 3.533872 | 4.083057 | 4.63E-20 | 2.75E-19 |
| SPP1 | 3.941019 | 7.370634 | 4.64E-20 | 2.75E-19 |
| DIO2 | 2.050875 | 3.540134 | 4.83E-20 | 2.86E-19 |
| SPDYC | 3.615592 | -0.25547 | 5.23E-20 | 3.09E-19 |
| KRTAP5-4 | 3.629205 | -2.39759 | 5.64E-20 | 3.32E-19 |
| PGC | 5.026833 | 1.336939 | 5.83E-20 | 3.43E-19 |
| HOXB8 | 2.790003 | 5.227173 | 5.86E-20 | 3.45E-19 |
| DUSP9 | 4.384454 | -0.02588 | 7.60E-20 | 4.44E-19 |
| TBX15 | 2.860464 | -0.07888 | 7.72E-20 | 4.50E-19 |
| RP1 | 5.178037 | -1.62968 | 7.82E-20 | 4.56E-19 |
| SLC11A1 | 2.38612 | 3.350112 | 8.12E-20 | 4.73E-19 |
| DNAH5 | 2.401792 | 0.853559 | 8.34E-20 | 4.85E-19 |
| ZNF280A | 4.807972 | -2.00495 | 1.14E-19 | 6.59E-19 |
| ASGR1 | 2.589212 | 2.329293 | 1.25E-19 | 7.18E-19 |
| SAPCD1 | 2.110283 | -0.08115 | 1.32E-19 | 7.59E-19 |
| TMEM211 | 3.805785 | 2.067084 | 1.33E-19 | 7.61E-19 |
| GLYATL1 | 3.117635 | 1.328004 | 1.38E-19 | 7.90E-19 |
| SLC13A3 | 4.223326 | 3.256687 | 1.76E-19 | 1.00E-18 |
| BMP7 | 3.282405 | 5.449456 | 1.91E-19 | 1.09E-18 |
| ADAMTS6 | 2.149833 | 1.455951 | 2.15E-19 | 1.22E-18 |
| ALDH3B2 | 3.62161 | 1.424671 | 2.19E-19 | 1.24E-18 |
| DACH1 | 2.167601 | 5.241764 | 2.23E-19 | 1.27E-18 |
| TP73 | 2.258602 | 2.418308 | 2.24E-19 | 1.27E-18 |
| LY6G6C | 3.111399 | 0.591405 | 2.55E-19 | 1.44E-18 |
| CPA4 | 3.70121 | -0.98698 | 3.21E-19 | 1.81E-18 |
| PIWIL1 | 4.953983 | 3.030833 | 3.51E-19 | 1.97E-18 |
| HTR1D | 2.823054 | 3.270761 | 3.99E-19 | 2.23E-18 |
| TAS2R38 | 2.760268 | -1.34565 | 4.26E-19 | 2.38E-18 |
| LRRN4 | 2.988072 | -1.1039 | 4.42E-19 | 2.46E-18 |
| CLDN14 | 3.104067 | 0.684241 | 4.83E-19 | 2.68E-18 |
| CDKN2A | 2.976185 | 3.372373 | 6.21E-19 | 3.42E-18 |
| LY6G6D | 5.569499 | 3.373756 | 6.51E-19 | 3.58E-18 |
| MSLNL | 5.515107 | -1.30516 | 7.11E-19 | 3.90E-18 |
| CXCL2 | 2.274071 | 4.789406 | 7.14E-19 | 3.93E-18 |
| SH2D5 | 3.139807 | -0.90008 | 7.62E-19 | 4.18E-18 |
| CCDC198 | 6.228697 | -0.76511 | 7.82E-19 | 4.28E-18 |
| ELF5 | 7.529553 | 1.752916 | 1.12E-18 | 6.08E-18 |
| CBARP | 2.432315 | 0.743384 | 1.21E-18 | 6.55E-18 |
| SP6 | 2.000909 | 4.828986 | 1.24E-18 | 6.69E-18 |
| SULT1C2 | 2.788169 | 3.801057 | 1.30E-18 | 7.00E-18 |
| KRTAP5-1 | 2.087824 | 0.211342 | 1.45E-18 | 7.81E-18 |
| KRT7 | 3.717169 | 3.837547 | 1.82E-18 | 9.77E-18 |
| CSF2 | 3.458661 | -0.33024 | 2.20E-18 | 1.17E-17 |
| NXPH1 | 4.536592 | -2.4896 | 2.97E-18 | 1.57E-17 |
| WDR97 | 2.514623 | 1.278965 | 4.38E-18 | 2.29E-17 |
| PAEP | 8.970793 | 2.84292 | 5.08E-18 | 2.65E-17 |
| AGT | 2.033343 | 4.885571 | 5.44E-18 | 2.83E-17 |
| CCDC148 | 2.193765 | -0.86943 | 6.61E-18 | 3.43E-17 |
| PRSS33 | 4.68045 | 3.26288 | 7.67E-18 | 3.96E-17 |
| KREMEN2 | 2.222927 | 1.37606 | 7.72E-18 | 3.98E-17 |
| UNC93A | 3.981811 | 2.282778 | 8.43E-18 | 4.33E-17 |
| TH | 3.280322 | 0.49978 | 8.48E-18 | 4.36E-17 |
| PPBP | 8.866776 | 5.837925 | 8.97E-18 | 4.60E-17 |
| ONECUT2 | 2.656268 | 2.531106 | 9.14E-18 | 4.68E-17 |
| PNPLA3 | 3.62619 | 0.72435 | 1.12E-17 | 5.72E-17 |
| HABP2 | 6.341753 | 1.286096 | 1.49E-17 | 7.55E-17 |
| KRT16 | 5.846847 | 2.105984 | 1.79E-17 | 8.98E-17 |
| SIX4 | 2.749253 | 0.948192 | 1.91E-17 | 9.58E-17 |
| TM4SF19 | 2.775598 | -1.53109 | 2.32E-17 | 1.16E-16 |
| TMPRSS5 | 3.287074 | 1.125576 | 2.50E-17 | 1.25E-16 |
| FOSL1 | 2.290921 | 4.137418 | 2.69E-17 | 1.34E-16 |
| MATN3 | 2.647931 | 1.686717 | 2.79E-17 | 1.39E-16 |
| EREG | 3.24634 | 5.907026 | 2.90E-17 | 1.44E-16 |
| LCN12 | 2.157019 | 2.265056 | 4.01E-17 | 1.97E-16 |
| FAM131C | 2.53115 | -0.68104 | 4.37E-17 | 2.15E-16 |
| CXCL8 | 3.61798 | 7.10338 | 4.69E-17 | 2.30E-16 |
| FOLR1 | 4.36267 | 2.717875 | 4.71E-17 | 2.31E-16 |
| CAMKV | 4.442925 | 0.630785 | 5.37E-17 | 2.63E-16 |
| DOK7 | 2.195295 | 1.850806 | 5.99E-17 | 2.92E-16 |
| SSTR5 | 4.237302 | 0.994111 | 7.10E-17 | 3.44E-16 |
| TRIM72 | 5.464682 | 2.397478 | 7.33E-17 | 3.55E-16 |
| RDH16 | 2.199677 | -0.28791 | 7.48E-17 | 3.62E-16 |
| PLPP4 | 2.837154 | 1.471007 | 8.51E-17 | 4.10E-16 |
| WFDC10A | 3.556454 | -2.57348 | 9.54E-17 | 4.59E-16 |
| GJB5 | 4.485423 | 2.207468 | 1.01E-16 | 4.85E-16 |
| GOLGA6L2 | 5.537238 | -2.28226 | 1.03E-16 | 4.92E-16 |
| SRMS | 2.129725 | 2.791926 | 1.18E-16 | 5.62E-16 |
| C9orf50 | 2.287536 | -1.35254 | 1.30E-16 | 6.18E-16 |
| EDAR | 2.877982 | 4.270318 | 1.31E-16 | 6.24E-16 |
| MYADML2 | 3.008814 | 1.369887 | 1.46E-16 | 6.91E-16 |
| TSPEAR | 3.164472 | 0.94203 | 1.51E-16 | 7.15E-16 |
| NMU | 2.907364 | 2.46474 | 1.71E-16 | 8.05E-16 |
| ERICH2 | 2.436148 | -0.02176 | 1.71E-16 | 8.05E-16 |
| DSC3 | 4.267463 | 3.884268 | 1.74E-16 | 8.21E-16 |
| DMRTA2 | 5.199842 | 0.916256 | 1.88E-16 | 8.84E-16 |
| HOGA1 | 2.748317 | -0.32656 | 1.97E-16 | 9.26E-16 |
| CCN6 | 4.243317 | 1.437588 | 2.42E-16 | 1.13E-15 |
| GSDMA | 2.32568 | 1.946301 | 2.42E-16 | 1.13E-15 |
| KISS1 | 2.730685 | -0.01278 | 2.53E-16 | 1.18E-15 |
| NEB | 2.768184 | 2.013861 | 2.87E-16 | 1.33E-15 |
| TNNT1 | 4.224708 | 2.19484 | 3.15E-16 | 1.46E-15 |
| CITED1 | 2.773471 | 0.834126 | 3.16E-16 | 1.47E-15 |
| PRSS56 | 9.436423 | 2.43045 | 4.34E-16 | 2.00E-15 |
| PRKCG | 3.640505 | 1.39216 | 4.41E-16 | 2.03E-15 |
| MMP8 | 5.612647 | 0.352035 | 4.44E-16 | 2.04E-15 |
| OBP2B | 6.465927 | -0.51491 | 4.55E-16 | 2.09E-15 |
| CHST4 | 5.079369 | 1.833275 | 5.12E-16 | 2.34E-15 |
| TMEM40 | 4.031662 | -1.40222 | 6.02E-16 | 2.75E-15 |
| SPRR2D | 7.40871 | 0.695558 | 6.08E-16 | 2.77E-15 |
| SLC26A9 | 6.596117 | 1.509098 | 6.36E-16 | 2.89E-15 |
| CSTL1 | 3.122175 | -2.03066 | 6.73E-16 | 3.06E-15 |
| COL9A3 | 3.112711 | 4.908472 | 7.48E-16 | 3.39E-15 |
| MAGEB17 | 5.496404 | 1.596993 | 7.63E-16 | 3.45E-15 |
| SPRR1A | 7.7584 | 0.486369 | 8.86E-16 | 3.99E-15 |
| CLPSL2 | 3.805297 | -3.2321 | 1.10E-15 | 4.91E-15 |
| CYP4X1 | 2.947689 | 3.548548 | 1.11E-15 | 4.96E-15 |
| DSG1 | 2.430506 | -0.65164 | 1.31E-15 | 5.84E-15 |
| MUC6 | 6.867988 | 4.600973 | 1.40E-15 | 6.23E-15 |
| GRK1 | 3.501837 | -0.87388 | 1.44E-15 | 6.42E-15 |
| CYP2W1 | 4.161095 | 6.034092 | 1.44E-15 | 6.43E-15 |
| PALM3 | 2.911233 | 2.062968 | 1.56E-15 | 6.91E-15 |
| CILP2 | 2.825662 | 1.085255 | 1.66E-15 | 7.34E-15 |
| CXCL5 | 4.552279 | 5.262792 | 1.80E-15 | 7.95E-15 |
| H1-4 | 6.373404 | 2.992811 | 2.07E-15 | 9.10E-15 |
| CXCL17 | 5.187067 | 0.810924 | 2.17E-15 | 9.54E-15 |
| WDR72 | 3.020385 | 4.122459 | 2.19E-15 | 9.65E-15 |
| ALOXE3 | 2.990609 | -1.45232 | 2.52E-15 | 1.11E-14 |
| PRSS41 | 3.913595 | -1.20325 | 2.68E-15 | 1.17E-14 |
| AL662899.3 | 3.350406 | -2.79927 | 3.12E-15 | 1.36E-14 |
| SEC14L4 | 4.531908 | -0.42923 | 3.25E-15 | 1.42E-14 |
| EVX1 | 3.415315 | 2.902174 | 3.34E-15 | 1.45E-14 |
| IGFL2 | 3.616662 | 1.697517 | 3.53E-15 | 1.54E-14 |
| DMRT3 | 4.135504 | -1.62558 | 3.56E-15 | 1.55E-14 |
| SFRP4 | 3.086064 | 5.443775 | 3.59E-15 | 1.56E-14 |
| GJA3 | 3.290562 | -0.32821 | 3.61E-15 | 1.57E-14 |
| FGF20 | 5.86663 | 0.021648 | 4.15E-15 | 1.80E-14 |
| MYBPHL | 5.542701 | -0.40166 | 4.24E-15 | 1.83E-14 |
| AKAP4 | 4.91633 | -1.96669 | 4.25E-15 | 1.83E-14 |
| DUSP15 | 2.402933 | 1.684733 | 4.94E-15 | 2.13E-14 |
| BAAT | 4.725423 | 0.404813 | 4.96E-15 | 2.13E-14 |
| NT5DC4 | 2.303047 | -0.77151 | 4.97E-15 | 2.14E-14 |
| KRT83 | 4.071447 | -1.30134 | 5.31E-15 | 2.28E-14 |
| DSG3 | 4.248921 | 3.815729 | 5.32E-15 | 2.29E-14 |
| CCR8 | 2.114453 | -0.96195 | 5.44E-15 | 2.34E-14 |
| ARHGAP40 | 4.827741 | -1.04694 | 5.53E-15 | 2.37E-14 |
| PADI3 | 5.151117 | 2.130882 | 6.08E-15 | 2.60E-14 |
| H1-5 | 6.860732 | 2.954878 | 6.38E-15 | 2.72E-14 |
| TREM1 | 2.588184 | 2.274768 | 6.57E-15 | 2.80E-14 |
| NDUFA4L2 | 2.335584 | 4.483373 | 7.32E-15 | 3.11E-14 |
| LAIR2 | 2.616739 | -0.4221 | 8.15E-15 | 3.46E-14 |
| ONECUT3 | 6.678756 | 2.101626 | 8.28E-15 | 3.52E-14 |
| MYOM3 | 2.03133 | 4.656448 | 1.06E-14 | 4.48E-14 |
| SP8 | 4.548679 | 0.223339 | 1.07E-14 | 4.50E-14 |
| NPC1L1 | 3.810157 | 2.59715 | 1.12E-14 | 4.72E-14 |
| WNT8B | 2.341048 | -1.73663 | 1.19E-14 | 4.98E-14 |
| MAT1A | 3.099082 | 2.015148 | 1.29E-14 | 5.40E-14 |
| SERPIND1 | 3.682393 | 0.744259 | 1.35E-14 | 5.67E-14 |
| BRSK2 | 2.710544 | 2.766393 | 1.37E-14 | 5.73E-14 |
| RAD51AP2 | 2.103227 | -1.33677 | 1.39E-14 | 5.82E-14 |
| NAT16 | 3.397813 | -1.64755 | 1.40E-14 | 5.87E-14 |
| C1orf105 | 2.966645 | -0.94435 | 1.55E-14 | 6.45E-14 |
| SLC38A5 | 2.050558 | 6.516078 | 1.80E-14 | 7.47E-14 |
| CCDC187 | 2.758098 | -0.03117 | 1.92E-14 | 7.96E-14 |
| MIOX | 3.405574 | -1.01128 | 1.92E-14 | 7.97E-14 |
| VGLL1 | 6.266557 | -0.95714 | 2.01E-14 | 8.33E-14 |
| F2 | 3.50052 | -0.50222 | 2.04E-14 | 8.43E-14 |
| KCNH4 | 2.058859 | -0.18012 | 2.15E-14 | 8.89E-14 |
| RASSF10 | 2.386539 | 2.946236 | 2.21E-14 | 9.11E-14 |
| CADPS | 2.145966 | 4.483234 | 2.43E-14 | 1.00E-13 |
| PRR9 | 7.802801 | 0.36047 | 2.62E-14 | 1.08E-13 |
| TRIM71 | 5.598239 | -0.06252 | 2.63E-14 | 1.08E-13 |
| KLK11 | 3.226351 | 3.571213 | 3.14E-14 | 1.28E-13 |
| IFITM5 | 4.099903 | -2.51164 | 3.27E-14 | 1.33E-13 |
| NEUROG2 | 5.451989 | -1.02415 | 3.44E-14 | 1.40E-13 |
| VENTX | 3.609868 | 2.289653 | 3.52E-14 | 1.43E-13 |
| CLDN9 | 2.514427 | 1.152208 | 3.59E-14 | 1.46E-13 |
| HCAR1 | 3.691187 | 1.371603 | 3.72E-14 | 1.51E-13 |
| AQP5 | 5.678627 | 2.610747 | 3.91E-14 | 1.58E-13 |
| IGF2BP3 | 4.142982 | 2.283966 | 4.78E-14 | 1.92E-13 |
| SPRR1B | 7.365759 | 0.921257 | 5.18E-14 | 2.07E-13 |
| WFDC10B | 2.325411 | -1.43675 | 5.49E-14 | 2.20E-13 |
| NACA2 | 2.586304 | 0.042855 | 5.57E-14 | 2.23E-13 |
| IRX3 | 3.255551 | 0.532897 | 5.64E-14 | 2.25E-13 |
| SPRR2A | 7.017657 | 0.645002 | 5.85E-14 | 2.33E-13 |
| CLDN18 | 7.029718 | 4.712269 | 6.47E-14 | 2.57E-13 |
| NPW | 2.87998 | 0.987428 | 6.54E-14 | 2.60E-13 |
| ERP27 | 2.671427 | 2.936029 | 7.29E-14 | 2.89E-13 |
| IL1A | 2.579718 | 1.566462 | 7.74E-14 | 3.06E-13 |
| DCSTAMP | 3.054139 | -0.93852 | 7.76E-14 | 3.07E-13 |
| F7 | 3.702617 | 1.386825 | 8.50E-14 | 3.36E-13 |
| TNNT2 | 2.312915 | 0.340109 | 9.07E-14 | 3.57E-13 |
| AFP | 4.65237 | -0.61548 | 9.21E-14 | 3.63E-13 |
| MSLN | 2.895272 | 6.346734 | 9.27E-14 | 3.65E-13 |
| NPSR1 | 5.670191 | 2.631795 | 9.38E-14 | 3.69E-13 |
| BEST3 | 4.257911 | -0.46855 | 9.57E-14 | 3.76E-13 |
| IGF2BP1 | 5.004876 | 2.573259 | 9.93E-14 | 3.90E-13 |
| H2AC12 | 5.898133 | 0.441067 | 1.03E-13 | 4.04E-13 |
| MMP20 | 3.686245 | -1.85701 | 1.04E-13 | 4.06E-13 |
| CBLIF | 3.562901 | 1.178519 | 1.05E-13 | 4.11E-13 |
| ERVMER34-1 | 2.608476 | 1.840726 | 1.12E-13 | 4.37E-13 |
| LRRC9 | 2.552821 | -1.7624 | 1.14E-13 | 4.44E-13 |
| H3C12 | 5.248547 | 0.247861 | 1.18E-13 | 4.61E-13 |
| RHBG | 3.015644 | -2.24726 | 1.18E-13 | 4.62E-13 |
| H4C5 | 4.915561 | 2.129818 | 1.23E-13 | 4.81E-13 |
| ABCA12 | 3.947653 | 1.259369 | 1.38E-13 | 5.38E-13 |
| H4C3 | 6.681286 | 2.011449 | 1.40E-13 | 5.44E-13 |
| THBS2 | 2.281082 | 7.205131 | 1.44E-13 | 5.60E-13 |
| MAGEA3 | 10.32599 | 2.628543 | 1.44E-13 | 5.61E-13 |
| SLC28A3 | 2.434451 | 3.113571 | 1.51E-13 | 5.87E-13 |
| APCDD1 | 2.486211 | 6.711568 | 1.60E-13 | 6.17E-13 |
| CKMT2 | 3.494775 | 4.131485 | 1.61E-13 | 6.21E-13 |
| C2CD4B | 2.438004 | 3.11587 | 1.70E-13 | 6.56E-13 |
| IZUMO1 | 2.301557 | -1.95607 | 1.72E-13 | 6.62E-13 |
| H4C2 | 6.510691 | 0.737045 | 1.77E-13 | 6.81E-13 |
| KRT31 | 7.165757 | -0.99025 | 1.86E-13 | 7.14E-13 |
| DLX6 | 4.002212 | 0.198425 | 1.89E-13 | 7.26E-13 |
| H2BU1 | 2.38587 | -0.28419 | 1.97E-13 | 7.58E-13 |
| SLCO1B3-SLCO1B7 | 4.210985 | -3.08666 | 2.16E-13 | 8.27E-13 |
| SLCO1B1 | 4.567549 | -2.12317 | 2.46E-13 | 9.38E-13 |
| RNF224 | 2.157271 | -0.83821 | 2.60E-13 | 9.91E-13 |
| ISM2 | 3.91234 | 1.737866 | 2.69E-13 | 1.03E-12 |
| MCEMP1 | 2.824608 | 0.634516 | 3.07E-13 | 1.16E-12 |
| GZMB | 2.175662 | 3.887503 | 3.40E-13 | 1.29E-12 |
| PLA2G3 | 4.369384 | 1.392934 | 3.46E-13 | 1.31E-12 |
| KRT6A | 5.64342 | 3.532174 | 3.70E-13 | 1.40E-12 |
| IGF2 | 5.249785 | 10.33667 | 3.71E-13 | 1.40E-12 |
| HCRT | 2.872359 | -2.54288 | 3.72E-13 | 1.40E-12 |
| BIRC7 | 2.482435 | 0.030734 | 3.75E-13 | 1.41E-12 |
| C20orf204 | 2.73663 | 1.070608 | 3.77E-13 | 1.42E-12 |
| USP26 | 4.056776 | -2.99103 | 4.07E-13 | 1.53E-12 |
| ORM2 | 3.005489 | -1.27171 | 4.12E-13 | 1.55E-12 |
| UPK2 | 4.290123 | -0.64495 | 4.37E-13 | 1.64E-12 |
| PRDM13 | 4.055035 | -0.9155 | 4.56E-13 | 1.71E-12 |
| H4C4 | 5.966903 | 1.598716 | 4.82E-13 | 1.81E-12 |
| CST6 | 4.732935 | 1.467831 | 5.17E-13 | 1.93E-12 |
| FXYD4 | 2.74966 | -0.66577 | 5.35E-13 | 1.99E-12 |
| LY6D | 5.322911 | 1.110478 | 6.31E-13 | 2.34E-12 |
| CALCA | 5.305943 | 1.742767 | 6.52E-13 | 2.42E-12 |
| KRT6C | 5.457132 | -1.3027 | 6.62E-13 | 2.45E-12 |
| KCNK9 | 2.830273 | -0.04731 | 6.99E-13 | 2.59E-12 |
| PRSS51 | 3.021574 | -0.05141 | 7.84E-13 | 2.89E-12 |
| VGF | 2.850992 | 2.596358 | 8.00E-13 | 2.95E-12 |
| BHLHA9 | 4.88202 | -1.93138 | 8.10E-13 | 2.98E-12 |
| IL17C | 3.244447 | -0.89124 | 8.30E-13 | 3.05E-12 |
| PRSS1 | 4.519228 | 0.848422 | 8.89E-13 | 3.27E-12 |
| TTC16 | 2.069716 | -0.97445 | 9.61E-13 | 3.52E-12 |
| PSORS1C2 | 2.911762 | -0.89343 | 9.69E-13 | 3.55E-12 |
| RHCG | 2.630955 | 1.361259 | 1.00E-12 | 3.67E-12 |
| DNAH12 | 2.098794 | -1.24303 | 1.05E-12 | 3.84E-12 |
| KRTAP3-1 | 5.149873 | -2.22312 | 1.12E-12 | 4.09E-12 |
| KISS1R | 3.072141 | -1.14996 | 1.21E-12 | 4.39E-12 |
| MPP4 | 2.077433 | -2.69438 | 1.28E-12 | 4.65E-12 |
| MMP10 | 2.445023 | 2.113447 | 1.29E-12 | 4.68E-12 |
| PADI1 | 2.495011 | 1.673593 | 1.48E-12 | 5.37E-12 |
| SYT8 | 2.196608 | 1.286984 | 1.55E-12 | 5.59E-12 |
| INHBB | 2.168832 | 3.257086 | 1.91E-12 | 6.87E-12 |
| MAGEA6 | 9.682092 | 2.351697 | 1.97E-12 | 7.09E-12 |
| DIRC1 | 3.216255 | -2.90647 | 2.16E-12 | 7.74E-12 |
| MYCN | 2.428939 | 2.101312 | 2.31E-12 | 8.25E-12 |
| CXCL6 | 2.355606 | 1.746127 | 2.45E-12 | 8.75E-12 |
| H2BC18 | 3.211311 | 1.257766 | 2.49E-12 | 8.89E-12 |
| GPR143 | 2.202615 | 3.110991 | 2.59E-12 | 9.23E-12 |
| RASL10B | 2.255709 | 1.693012 | 2.60E-12 | 9.25E-12 |
| OSM | 2.226021 | 2.746396 | 2.63E-12 | 9.36E-12 |
| RNF182 | 3.52305 | 0.789832 | 2.67E-12 | 9.48E-12 |
| WNT11 | 2.651905 | 4.668616 | 2.85E-12 | 1.01E-11 |
| SOX1 | 4.604003 | 1.080615 | 2.85E-12 | 1.01E-11 |
| SERPINB7 | 4.77645 | 0.543123 | 3.62E-12 | 1.28E-11 |
| H2AC4 | 5.861059 | -0.14488 | 3.87E-12 | 1.36E-11 |
| RP1L1 | 2.300114 | -1.30892 | 4.65E-12 | 1.63E-11 |
| DLL3 | 2.662311 | -0.55254 | 5.52E-12 | 1.92E-11 |
| CXCL11 | 2.512499 | 4.12596 | 5.62E-12 | 1.95E-11 |
| PTF1A | 6.078209 | -1.1376 | 5.71E-12 | 1.98E-11 |
| H2BC17 | 4.844527 | 0.732622 | 5.72E-12 | 1.99E-11 |
| UPK1A | 3.844983 | -0.97709 | 5.94E-12 | 2.06E-11 |
| CLIC3 | 2.052524 | 3.018904 | 6.09E-12 | 2.11E-11 |
| H2AC16 | 4.978119 | -0.24851 | 6.39E-12 | 2.21E-11 |
| AC004805.1 | 2.355915 | -2.72608 | 6.44E-12 | 2.23E-11 |
| POU4F1 | 2.723254 | -0.82505 | 6.78E-12 | 2.34E-11 |
| TEX19 | 3.024966 | -2.03865 | 6.92E-12 | 2.39E-11 |
| SLCO1A2 | 4.056806 | -1.02863 | 7.12E-12 | 2.45E-11 |
| METTL11B | 2.873298 | -3.11575 | 7.30E-12 | 2.51E-11 |
| ORM1 | 6.254211 | 1.839591 | 7.35E-12 | 2.52E-11 |
| PGLYRP3 | 4.350684 | -2.29844 | 8.72E-12 | 2.98E-11 |
| ITGBL1 | 2.386121 | 2.436115 | 8.73E-12 | 2.98E-11 |
| POU3F2 | 2.915967 | -2.15214 | 9.06E-12 | 3.09E-11 |
| KLRG2 | 3.74587 | 0.327912 | 9.42E-12 | 3.21E-11 |
| CALB1 | 5.315392 | 3.731677 | 9.90E-12 | 3.36E-11 |
| MUC16 | 4.424343 | 1.22204 | 1.00E-11 | 3.41E-11 |
| KRT37 | 4.554822 | -2.85468 | 1.02E-11 | 3.47E-11 |
| CACNG4 | 3.550569 | 2.327416 | 1.08E-11 | 3.66E-11 |
| CST5 | 4.279791 | -2.27697 | 1.10E-11 | 3.73E-11 |
| PLAC1 | 2.653584 | -0.67015 | 1.14E-11 | 3.84E-11 |
| H2BC10 | 6.724654 | 0.576775 | 1.25E-11 | 4.21E-11 |
| GAST | 3.616789 | -2.7131 | 1.29E-11 | 4.35E-11 |
| MAGEA12 | 8.939977 | 1.562318 | 1.30E-11 | 4.36E-11 |
| B4GALNT4 | 2.685374 | 3.576148 | 1.30E-11 | 4.36E-11 |
| KRTAP3-2 | 3.778837 | -3.05532 | 1.35E-11 | 4.55E-11 |
| KRT32 | 4.684289 | -2.65849 | 1.41E-11 | 4.72E-11 |
| LYPD3 | 2.022576 | 2.350911 | 1.55E-11 | 5.17E-11 |
| ATP6V0A4 | 3.785978 | -1.4769 | 1.58E-11 | 5.29E-11 |
| C9orf57 | 2.978752 | -3.1244 | 1.65E-11 | 5.50E-11 |
| SPAG17 | 2.742694 | -1.40809 | 1.68E-11 | 5.62E-11 |
| IVL | 6.661733 | -1.40377 | 1.86E-11 | 6.20E-11 |
| KLK12 | 3.148995 | 2.58073 | 2.15E-11 | 7.12E-11 |
| GNG4 | 2.626734 | 5.468867 | 2.15E-11 | 7.13E-11 |
| SERPINB2 | 4.25066 | 0.490528 | 2.18E-11 | 7.22E-11 |
| GNGT1 | 3.206226 | -1.13948 | 2.19E-11 | 7.25E-11 |
| CCL26 | 2.225706 | 0.047192 | 2.22E-11 | 7.35E-11 |
| SPZ1 | 3.123744 | -3.41323 | 2.23E-11 | 7.37E-11 |
| DKK1 | 3.778993 | 1.607787 | 2.29E-11 | 7.55E-11 |
| AC010422.3 | 2.60561 | 1.774999 | 2.41E-11 | 7.97E-11 |
| FGF3 | 5.458885 | -1.7453 | 2.46E-11 | 8.09E-11 |
| HES7 | 3.056304 | -2.00075 | 2.47E-11 | 8.13E-11 |
| H3C11 | 5.608519 | -0.24234 | 2.53E-11 | 8.32E-11 |
| GABRP | 3.314343 | 3.239726 | 2.56E-11 | 8.44E-11 |
| RIPPLY1 | 2.209303 | -2.19091 | 2.65E-11 | 8.72E-11 |
| KIAA1549L | 2.547713 | 2.350482 | 2.69E-11 | 8.83E-11 |
| BEAN1 | 2.130464 | 0.499713 | 2.73E-11 | 8.96E-11 |
| H2AC14 | 5.176659 | 0.541452 | 2.78E-11 | 9.12E-11 |
| ZNF750 | 2.926574 | -0.11777 | 2.99E-11 | 9.80E-11 |
| MYH4 | 2.957363 | -1.07553 | 3.23E-11 | 1.05E-10 |
| DRD2 | 3.220281 | 2.246693 | 3.32E-11 | 1.08E-10 |
| LHX5 | 3.503442 | -2.24815 | 3.56E-11 | 1.16E-10 |
| CREG2 | 2.473044 | 0.460699 | 4.27E-11 | 1.38E-10 |
| FOXI3 | 5.130298 | -2.01799 | 4.38E-11 | 1.41E-10 |
| DMRT1 | 5.069238 | -1.87391 | 4.65E-11 | 1.50E-10 |
| TCP11 | 4.656145 | 0.410353 | 4.93E-11 | 1.58E-10 |
| S100A7 | 5.094259 | -1.06772 | 5.06E-11 | 1.63E-10 |
| FGF8 | 2.74576 | -2.56645 | 5.18E-11 | 1.66E-10 |
| ATP6V1B1 | 2.136558 | -0.65715 | 6.29E-11 | 2.01E-10 |
| SPRR3 | 6.591023 | 1.113911 | 6.70E-11 | 2.14E-10 |
| EPHA8 | 5.04761 | -1.05716 | 6.78E-11 | 2.16E-10 |
| MUC5AC | 4.079729 | 5.833996 | 7.11E-11 | 2.26E-10 |
| AKR1C8P | 3.387319 | -2.48487 | 7.42E-11 | 2.35E-10 |
| NKX2-1 | 7.148071 | 0.943401 | 7.95E-11 | 2.51E-10 |
| KCNJ15 | 2.212184 | 1.101042 | 8.97E-11 | 2.82E-10 |
| KRT35 | 4.419459 | -2.77817 | 9.80E-11 | 3.08E-10 |
| H2BC13 | 3.676925 | 0.472285 | 1.07E-10 | 3.36E-10 |
| DMP1 | 3.598801 | -3.27648 | 1.11E-10 | 3.47E-10 |
| MAGEA11 | 6.153795 | 0.891702 | 1.32E-10 | 4.11E-10 |
| RNF225 | 3.276596 | -3.34765 | 1.34E-10 | 4.18E-10 |
| MUCL3 | 4.244211 | -0.68121 | 1.41E-10 | 4.38E-10 |
| SIGLEC15 | 2.15955 | 1.40299 | 1.44E-10 | 4.47E-10 |
| PNMA5 | 7.125932 | 2.328791 | 1.50E-10 | 4.63E-10 |
| CSAG1 | 6.945071 | 0.609202 | 1.51E-10 | 4.68E-10 |
| H2AC21 | 5.503189 | -0.35454 | 1.53E-10 | 4.72E-10 |
| LCN2 | 2.146199 | 9.424634 | 1.54E-10 | 4.76E-10 |
| IFNE | 3.770049 | -2.4089 | 1.56E-10 | 4.83E-10 |
| CYP4F8 | 2.994122 | -0.83594 | 1.60E-10 | 4.94E-10 |
| LRP2 | 3.562418 | -1.65739 | 1.64E-10 | 5.05E-10 |
| CGB5 | 4.728212 | -2.81725 | 1.65E-10 | 5.08E-10 |
| IGFL3 | 3.49189 | -1.53119 | 1.65E-10 | 5.09E-10 |
| SIX2 | 2.97847 | 0.738596 | 1.74E-10 | 5.37E-10 |
| SAA4 | 3.298537 | -2.40629 | 1.82E-10 | 5.59E-10 |
| TBX20 | 3.181102 | -0.96935 | 2.19E-10 | 6.71E-10 |
| FBXO2 | 2.367694 | 3.366279 | 2.28E-10 | 6.98E-10 |
| SLC6A20 | 2.135632 | 4.935496 | 2.35E-10 | 7.18E-10 |
| FGB | 3.809645 | 0.444453 | 2.59E-10 | 7.90E-10 |
| LCE1E | 4.416904 | -3.1119 | 2.61E-10 | 7.95E-10 |
| HEPHL1 | 2.881313 | 1.173014 | 2.75E-10 | 8.38E-10 |
| DPEP1 | 2.385069 | 8.155332 | 3.08E-10 | 9.32E-10 |
| BHMG1 | 2.801132 | -3.02622 | 3.30E-10 | 9.98E-10 |
| AQP9 | 2.329492 | 2.710957 | 3.36E-10 | 1.02E-09 |
| FOXD1 | 2.947601 | 1.351105 | 3.54E-10 | 1.07E-09 |
| IL36RN | 3.454741 | -2.04925 | 3.54E-10 | 1.07E-09 |
| MYBPC3 | 2.104734 | -0.92601 | 3.55E-10 | 1.07E-09 |
| IZUMO2 | 3.200851 | -0.88888 | 3.59E-10 | 1.08E-09 |
| ERVV-2 | 4.248661 | -2.86991 | 3.66E-10 | 1.10E-09 |
| SPRR2E | 7.377126 | -0.0719 | 3.71E-10 | 1.12E-09 |
| LYZL4 | 2.847503 | -3.47945 | 3.71E-10 | 1.12E-09 |
| FGF19 | 3.716424 | 1.937791 | 3.90E-10 | 1.18E-09 |
| A2ML1 | 4.416805 | 0.498856 | 4.17E-10 | 1.25E-09 |
| ANKRD18B | 3.243728 | -1.4487 | 4.33E-10 | 1.30E-09 |
| IGFBP1 | 3.378529 | -0.3547 | 4.47E-10 | 1.34E-09 |
| CA5A | 2.049196 | -2.71835 | 4.78E-10 | 1.43E-09 |
| TBX4 | 3.578871 | 0.09468 | 5.44E-10 | 1.62E-09 |
| ANKRD33 | 2.719601 | -2.55193 | 5.46E-10 | 1.63E-09 |
| GRM8 | 2.115861 | 3.989878 | 5.48E-10 | 1.63E-09 |
| NPIPA3 | 2.083437 | -1.52004 | 5.59E-10 | 1.66E-09 |
| CD200R1L | 4.48713 | -1.96114 | 5.70E-10 | 1.69E-09 |
| EYA1 | 3.056137 | 1.375064 | 5.92E-10 | 1.76E-09 |
| VTCN1 | 3.456051 | -0.79834 | 5.93E-10 | 1.76E-09 |
| GRPR | 2.788883 | -0.00534 | 5.95E-10 | 1.76E-09 |
| AQP2 | 3.016292 | -2.863 | 6.01E-10 | 1.78E-09 |
| LGALS7B | 2.642592 | -2.16852 | 6.13E-10 | 1.82E-09 |
| GSC | 2.02702 | -2.39871 | 6.30E-10 | 1.86E-09 |
| MUC21 | 4.138119 | -2.88418 | 6.56E-10 | 1.94E-09 |
| CLLU1-AS1 | 2.826098 | -1.68682 | 6.86E-10 | 2.03E-09 |
| FZD10 | 3.190952 | 3.008997 | 7.64E-10 | 2.25E-09 |
| KLK5 | 6.268953 | -0.14816 | 7.87E-10 | 2.31E-09 |
| SLC38A3 | 2.787695 | 0.437195 | 8.04E-10 | 2.36E-09 |
| H1-3 | 4.418527 | 1.716511 | 8.71E-10 | 2.55E-09 |
| SLC6A14 | 2.351747 | 4.104556 | 9.55E-10 | 2.78E-09 |
| CALML3 | 2.800529 | 0.171814 | 9.99E-10 | 2.91E-09 |
| H2BC6 | 3.791926 | -0.24607 | 1.00E-09 | 2.92E-09 |
| SP9 | 5.232664 | -1.58101 | 1.04E-09 | 3.02E-09 |
| SERPINB4 | 5.742526 | -1.44317 | 1.07E-09 | 3.12E-09 |
| SPACA3 | 2.653524 | 0.185954 | 1.09E-09 | 3.16E-09 |
| H4C13 | 6.836806 | -0.38474 | 1.15E-09 | 3.32E-09 |
| SAA2 | 2.555181 | 1.961555 | 1.15E-09 | 3.34E-09 |
| AC005324.3 | 2.313422 | -2.37233 | 1.21E-09 | 3.51E-09 |
| KRT38 | 4.083891 | -3.06634 | 1.35E-09 | 3.89E-09 |
| PRSS2 | 4.886818 | 5.657506 | 1.37E-09 | 3.95E-09 |
| MYO18B | 2.524498 | -1.09443 | 1.38E-09 | 3.96E-09 |
| MAGEC1 | 6.446506 | -1.59024 | 1.40E-09 | 4.03E-09 |
| CHRND | 4.491114 | -2.20414 | 1.45E-09 | 4.16E-09 |
| H2BC7 | 3.615427 | 0.666281 | 1.59E-09 | 4.54E-09 |
| DLX3 | 3.13102 | 1.077981 | 1.60E-09 | 4.57E-09 |
| F5 | 2.331614 | 3.149646 | 1.63E-09 | 4.67E-09 |
| AHSG | 4.175515 | 0.009162 | 1.66E-09 | 4.75E-09 |
| CHRNA6 | 2.403153 | -2.49591 | 1.71E-09 | 4.90E-09 |
| KCNV2 | 2.166006 | -2.41397 | 1.84E-09 | 5.23E-09 |
| MEGF11 | 2.25839 | 0.541513 | 1.88E-09 | 5.35E-09 |
| ARMC3 | 2.971645 | -1.61717 | 1.97E-09 | 5.59E-09 |
| FGF21 | 3.024539 | -3.58374 | 2.02E-09 | 5.74E-09 |
| SMTNL2 | 2.064168 | 1.018204 | 2.19E-09 | 6.18E-09 |
| SOHLH1 | 5.631539 | -1.2376 | 2.37E-09 | 6.67E-09 |
| SERPINA7 | 3.356591 | 0.084918 | 2.82E-09 | 7.89E-09 |
| C10orf113 | 4.635298 | -2.44082 | 2.82E-09 | 7.89E-09 |
| GSX1 | 3.646025 | -3.40867 | 2.94E-09 | 8.21E-09 |
| EGR4 | 2.170542 | -1.28311 | 2.98E-09 | 8.31E-09 |
| HOXC12 | 5.486697 | -1.05673 | 3.10E-09 | 8.64E-09 |
| H2AC17 | 3.239134 | 0.371325 | 3.28E-09 | 9.12E-09 |
| UROC1 | 2.561336 | -2.90704 | 3.43E-09 | 9.53E-09 |
| HBE1 | 6.170699 | -0.29517 | 3.49E-09 | 9.70E-09 |
| H2AC20 | 2.547508 | 1.327258 | 3.53E-09 | 9.79E-09 |
| MT4 | 6.279236 | -0.98629 | 3.59E-09 | 9.96E-09 |
| GPR87 | 5.36579 | -1.472 | 3.59E-09 | 9.96E-09 |
| POU5F2 | 4.970251 | -0.76546 | 3.73E-09 | 1.03E-08 |
| SEMG2 | 5.077974 | -1.99523 | 3.89E-09 | 1.08E-08 |
| SMIM36 | 2.430748 | -3.56458 | 3.96E-09 | 1.10E-08 |
| INHA | 2.919368 | -1.46146 | 3.98E-09 | 1.10E-08 |
| SIX3 | 3.607503 | -1.39723 | 4.01E-09 | 1.11E-08 |
| SERPINB3 | 5.679102 | -0.87792 | 4.13E-09 | 1.14E-08 |
| KRT4 | 3.499317 | -1.23035 | 4.65E-09 | 1.28E-08 |
| UNC5A | 2.301658 | 0.193766 | 4.79E-09 | 1.32E-08 |
| KRT34 | 3.620698 | -3.22371 | 4.92E-09 | 1.35E-08 |
| HBQ1 | 3.084535 | -1.99955 | 4.99E-09 | 1.37E-08 |
| MAGEA10 | 6.142196 | -1.13101 | 5.00E-09 | 1.37E-08 |
| HS3ST4 | 4.616648 | -0.18502 | 5.25E-09 | 1.43E-08 |
| SBSN | 2.835176 | -1.35355 | 5.38E-09 | 1.47E-08 |
| CPN1 | 3.275986 | -0.19598 | 5.43E-09 | 1.48E-08 |
| IL17A | 2.390492 | -1.16903 | 5.44E-09 | 1.48E-08 |
| H4C1 | 4.359461 | -1.1154 | 5.61E-09 | 1.53E-08 |
| ZNF556 | 3.188029 | -0.29461 | 5.93E-09 | 1.61E-08 |
| CHRNG | 2.140212 | -2.00965 | 6.06E-09 | 1.65E-08 |
| RGS20 | 2.145065 | -1.6787 | 6.58E-09 | 1.78E-08 |
| MUC19 | 3.512097 | -2.77142 | 6.76E-09 | 1.83E-08 |
| MAGEA1 | 7.625678 | 0.052214 | 6.77E-09 | 1.83E-08 |
| CYP26A1 | 2.427917 | -1.16737 | 7.48E-09 | 2.02E-08 |
| NPTX2 | 2.610141 | 4.685085 | 9.26E-09 | 2.48E-08 |
| CLDN6 | 3.178583 | -0.97881 | 9.34E-09 | 2.50E-08 |
| ANXA10 | 4.441515 | 1.271543 | 9.40E-09 | 2.52E-08 |
| RGR | 3.43449 | -1.19416 | 9.59E-09 | 2.56E-08 |
| HOXC13 | 4.799231 | -1.74731 | 9.60E-09 | 2.57E-08 |
| MS4A15 | 2.359609 | -0.56888 | 1.03E-08 | 2.75E-08 |
| NMUR2 | 4.143503 | 0.105508 | 1.09E-08 | 2.90E-08 |
| RNF113B | 4.02335 | -2.96553 | 1.13E-08 | 3.01E-08 |
| VNN3 | 2.06165 | -0.81798 | 1.16E-08 | 3.09E-08 |
| FAM9A | 3.799575 | -3.32041 | 1.17E-08 | 3.09E-08 |
| TLX3 | 4.129768 | -2.2575 | 1.27E-08 | 3.35E-08 |
| KRT5 | 4.340121 | 3.592555 | 1.30E-08 | 3.44E-08 |
| C5orf38 | 2.960004 | 0.905128 | 1.32E-08 | 3.49E-08 |
| C19orf81 | 2.692602 | -2.3687 | 1.38E-08 | 3.63E-08 |
| TFF2 | 3.31205 | 4.031366 | 1.38E-08 | 3.63E-08 |
| ACTL8 | 2.735639 | 1.719323 | 1.38E-08 | 3.64E-08 |
| TRIM54 | 2.751401 | 1.767734 | 1.43E-08 | 3.76E-08 |
| DLX5 | 2.622825 | -0.3106 | 1.53E-08 | 4.01E-08 |
| COLEC10 | 2.416798 | -0.92941 | 1.57E-08 | 4.12E-08 |
| GRP | 2.033961 | 0.492691 | 1.59E-08 | 4.16E-08 |
| TTC29 | 5.350889 | -0.28957 | 1.69E-08 | 4.42E-08 |
| GCM1 | 2.398675 | -2.31587 | 1.73E-08 | 4.51E-08 |
| GATA4 | 4.626524 | 1.089111 | 1.84E-08 | 4.80E-08 |
| HCAR3 | 2.671209 | 0.1505 | 1.93E-08 | 5.02E-08 |
| FGF23 | 3.886531 | -2.22286 | 2.03E-08 | 5.29E-08 |
| TBX5 | 4.016586 | -2.16174 | 2.10E-08 | 5.45E-08 |
| TRIM7 | 2.014591 | 3.018088 | 2.17E-08 | 5.63E-08 |
| OBP2A | 2.789129 | -2.49783 | 2.17E-08 | 5.63E-08 |
| ANKRD1 | 2.618083 | -1.92516 | 2.22E-08 | 5.74E-08 |
| PLK5 | 2.236776 | -1.56426 | 2.28E-08 | 5.90E-08 |
| PRAME | 2.639435 | 1.755032 | 2.35E-08 | 6.07E-08 |
| FEZF2 | 3.557041 | -3.18479 | 2.43E-08 | 6.27E-08 |
| KAAG1 | 2.193137 | -2.86131 | 2.49E-08 | 6.41E-08 |
| SYNGR4 | 2.460615 | -0.16656 | 2.64E-08 | 6.80E-08 |
| MAGEA4 | 6.165213 | -0.4303 | 2.79E-08 | 7.18E-08 |
| H2AC7 | 2.863169 | 1.306853 | 2.90E-08 | 7.45E-08 |
| TMPRSS11E | 4.696529 | 0.167397 | 2.93E-08 | 7.51E-08 |
| HOXC11 | 3.727219 | 0.787922 | 2.95E-08 | 7.56E-08 |
| PAX7 | 5.479015 | -0.65975 | 3.07E-08 | 7.87E-08 |
| H3C13 | 2.763583 | -1.79311 | 3.24E-08 | 8.30E-08 |
| GBP7 | 2.176559 | -2.24195 | 3.27E-08 | 8.36E-08 |
| LHX1 | 3.480456 | -2.88803 | 3.49E-08 | 8.91E-08 |
| HCAR2 | 2.311821 | 1.449105 | 3.65E-08 | 9.31E-08 |
| FOXL2NB | 3.535393 | -2.72193 | 3.70E-08 | 9.44E-08 |
| CDC20B | 3.143079 | -2.54512 | 3.75E-08 | 9.54E-08 |
| PGLYRP4 | 2.280961 | -1.93512 | 3.89E-08 | 9.89E-08 |
| GJB6 | 2.70749 | -1.33326 | 3.96E-08 | 1.01E-07 |
| REN | 2.432913 | 1.410443 | 4.14E-08 | 1.05E-07 |
| ODAM | 2.204661 | 2.058858 | 4.33E-08 | 1.10E-07 |
| PRSS48 | 4.396326 | -0.66836 | 4.38E-08 | 1.11E-07 |
| H2BC3 | 6.556892 | -0.04191 | 4.76E-08 | 1.20E-07 |
| LHX3 | 3.913885 | -2.65194 | 4.83E-08 | 1.22E-07 |
| SAGE1 | 4.565759 | -2.64534 | 4.85E-08 | 1.22E-07 |
| H3C7 | 3.75731 | 0.666025 | 4.95E-08 | 1.25E-07 |
| PAX2 | 2.755195 | -2.152 | 4.99E-08 | 1.26E-07 |
| ADAMTS20 | 4.33059 | -2.72996 | 5.05E-08 | 1.27E-07 |
| EPGN | 2.723589 | -2.8184 | 5.07E-08 | 1.28E-07 |
| SERPINA10 | 2.516475 | 1.185851 | 5.44E-08 | 1.37E-07 |
| IL36G | 2.220546 | -2.01912 | 5.60E-08 | 1.40E-07 |
| MUC15 | 4.59303 | -1.64074 | 5.79E-08 | 1.45E-07 |
| BTN1A1 | 2.084242 | -0.17896 | 5.89E-08 | 1.47E-07 |
| MAGEC2 | 6.647359 | -0.82516 | 5.92E-08 | 1.48E-07 |
| SMC1B | 2.005951 | -0.78282 | 6.01E-08 | 1.50E-07 |
| KRT74 | 3.249328 | -3.31323 | 6.24E-08 | 1.56E-07 |
| ACTBL2 | 2.9339 | -2.63761 | 6.24E-08 | 1.56E-07 |
| POU6F2 | 3.425349 | 1.26301 | 6.31E-08 | 1.57E-07 |
| USP41 | 2.0268 | -2.14642 | 6.64E-08 | 1.65E-07 |
| AIRE | 2.83465 | -0.16899 | 6.65E-08 | 1.66E-07 |
| SLC22A12 | 2.878358 | -3.42607 | 6.68E-08 | 1.66E-07 |
| GFY | 3.395571 | -2.9696 | 7.05E-08 | 1.75E-07 |
| UGT2B4 | 4.453997 | -0.5574 | 7.19E-08 | 1.79E-07 |
| KCNA10 | 2.496568 | -3.52935 | 7.25E-08 | 1.80E-07 |
| C20orf85 | 3.750452 | -2.52463 | 7.37E-08 | 1.83E-07 |
| FOXL2 | 3.346223 | -2.45617 | 7.43E-08 | 1.84E-07 |
| CYP4Z1 | 2.402194 | -2.49471 | 7.47E-08 | 1.85E-07 |
| OR1J4 | 2.636952 | -3.49289 | 7.56E-08 | 1.87E-07 |
| ITIH6 | 2.477526 | -1.86146 | 7.65E-08 | 1.90E-07 |
| GPRC6A | 3.231329 | -1.89811 | 7.82E-08 | 1.94E-07 |
| HCN1 | 2.283159 | 0.255732 | 8.05E-08 | 1.99E-07 |
| NKX2-5 | 3.61654 | -2.54786 | 8.13E-08 | 2.01E-07 |
| H2BC14 | 4.431985 | -0.36995 | 8.22E-08 | 2.03E-07 |
| FAM83A | 2.148153 | 1.421828 | 8.41E-08 | 2.08E-07 |
| IRX2 | 2.777091 | 2.701953 | 8.65E-08 | 2.13E-07 |
| H3C4 | 2.136696 | 1.126795 | 8.70E-08 | 2.14E-07 |
| CGB3 | 4.57922 | -2.97371 | 8.78E-08 | 2.16E-07 |
| MAP7D2 | 2.274235 | 3.53378 | 8.93E-08 | 2.20E-07 |
| HTR2C | 5.035706 | -2.19799 | 9.01E-08 | 2.22E-07 |
| ANKUB1 | 3.077103 | -2.43119 | 9.29E-08 | 2.28E-07 |
| INSL4 | 5.151511 | -1.42668 | 9.35E-08 | 2.30E-07 |
| DDX53 | 5.36347 | -2.17319 | 9.83E-08 | 2.41E-07 |
| SLC22A31 | 2.77751 | 0.999006 | 1.02E-07 | 2.50E-07 |
| LCN1 | 2.305846 | -3.13401 | 1.05E-07 | 2.58E-07 |
| XAGE2 | 5.06786 | -1.96841 | 1.06E-07 | 2.58E-07 |
| MYH6 | 2.92183 | -3.37328 | 1.06E-07 | 2.59E-07 |
| SHISAL2B | 2.206117 | -3.08765 | 1.08E-07 | 2.63E-07 |
| NR5A1 | 2.890931 | -2.63004 | 1.10E-07 | 2.68E-07 |
| STRIT1 | 4.368332 | -1.88257 | 1.16E-07 | 2.81E-07 |
| OR5M11 | 2.266525 | -3.5856 | 1.21E-07 | 2.93E-07 |
| CLEC2A | 4.734913 | -2.85712 | 1.30E-07 | 3.15E-07 |
| TYR | 3.922059 | -2.72233 | 1.31E-07 | 3.16E-07 |
| ANGPTL3 | 2.914954 | -1.3781 | 1.34E-07 | 3.25E-07 |
| SHOC1 | 2.810293 | -0.26777 | 1.36E-07 | 3.29E-07 |
| WNT3A | 2.296339 | -2.42964 | 1.39E-07 | 3.35E-07 |
| CSAG3 | 4.493534 | -2.34632 | 1.43E-07 | 3.45E-07 |
| SLC6A3 | 2.216468 | -2.39372 | 1.53E-07 | 3.69E-07 |
| MUCL1 | 3.274373 | -0.72239 | 1.67E-07 | 4.01E-07 |
| PBOV1 | 4.181787 | -1.81306 | 1.70E-07 | 4.08E-07 |
| ASB18 | 2.827176 | -3.56643 | 1.74E-07 | 4.15E-07 |
| MAS1 | 2.50762 | -3.5075 | 1.78E-07 | 4.25E-07 |
| TCHH | 2.11036 | -0.00017 | 1.78E-07 | 4.26E-07 |
| CALHM3 | 2.449106 | -1.98602 | 1.84E-07 | 4.38E-07 |
| RPTN | 4.247216 | -2.85402 | 1.84E-07 | 4.38E-07 |
| KRT78 | 4.061168 | -2.20783 | 1.84E-07 | 4.40E-07 |
| FBXO47 | 3.688762 | -2.84113 | 1.89E-07 | 4.50E-07 |
| HTN1 | 4.374783 | -2.99691 | 2.24E-07 | 5.31E-07 |
| MROH9 | 3.629064 | -3.19829 | 2.50E-07 | 5.88E-07 |
| TAS2R30 | 4.365478 | -1.44894 | 2.61E-07 | 6.14E-07 |
| SEMG1 | 3.089896 | 0.571523 | 2.71E-07 | 6.38E-07 |
| OTP | 2.271565 | -3.5712 | 2.78E-07 | 6.52E-07 |
| NKX2-8 | 3.853054 | -2.87944 | 2.82E-07 | 6.61E-07 |
| LCN15 | 3.699846 | 5.95067 | 2.86E-07 | 6.70E-07 |
| CGB7 | 2.26626 | -3.11406 | 2.90E-07 | 6.80E-07 |
| C1QTNF8 | 2.54614 | -2.939 | 2.98E-07 | 6.99E-07 |
| PRB1 | 4.010179 | -2.99573 | 3.06E-07 | 7.17E-07 |
| H3C1 | 3.21292 | -0.61162 | 3.07E-07 | 7.18E-07 |
| CT83 | 5.610809 | -1.39729 | 3.08E-07 | 7.19E-07 |
| FGA | 2.372793 | 0.035525 | 3.16E-07 | 7.37E-07 |
| CAPN14 | 2.004572 | -0.73009 | 3.28E-07 | 7.65E-07 |
| KCNT1 | 2.013035 | 0.088053 | 3.33E-07 | 7.76E-07 |
| OTOG | 2.245771 | -2.4559 | 3.36E-07 | 7.84E-07 |
| COX7B2 | 4.576085 | -2.8245 | 3.37E-07 | 7.85E-07 |
| RAMACL | 2.161814 | -2.14197 | 3.44E-07 | 8.00E-07 |
| CGB8 | 3.53136 | -3.54614 | 3.65E-07 | 8.49E-07 |
| WIF1 | 3.528039 | 2.311982 | 3.72E-07 | 8.63E-07 |
| NXF3 | 2.814729 | 1.459509 | 3.78E-07 | 8.76E-07 |
| CFAP47 | 2.542518 | -2.46915 | 3.97E-07 | 9.20E-07 |
| ERVV-1 | 2.560699 | -2.9801 | 4.10E-07 | 9.48E-07 |
| FIBCD1 | 2.024728 | 4.074249 | 4.45E-07 | 1.03E-06 |
| IGFN1 | 2.051849 | 0.783014 | 4.78E-07 | 1.10E-06 |
| CA6 | 3.338111 | -1.78758 | 4.94E-07 | 1.13E-06 |
| ASB4 | 2.177001 | -0.41114 | 5.01E-07 | 1.15E-06 |
| PNMT | 2.257241 | -0.36696 | 5.08E-07 | 1.17E-06 |
| SPINT4 | 4.13118 | -3.06144 | 5.25E-07 | 1.20E-06 |
| LIN28A | 3.638156 | -2.31695 | 5.32E-07 | 1.22E-06 |
| HPN | 2.394917 | 2.317204 | 5.32E-07 | 1.22E-06 |
| SLC13A5 | 2.13008 | -1.09411 | 5.52E-07 | 1.26E-06 |
| PLA2G2F | 2.683693 | 0.652792 | 5.72E-07 | 1.31E-06 |
| OTX2 | 3.481851 | -3.52312 | 6.12E-07 | 1.40E-06 |
| KRT40 | 3.127501 | 2.384224 | 6.25E-07 | 1.42E-06 |
| KCTD16 | 2.078963 | -0.06527 | 6.89E-07 | 1.56E-06 |
| KRTAP1-1 | 3.048998 | -3.56248 | 7.37E-07 | 1.67E-06 |
| DCAF8L2 | 3.152435 | -3.59594 | 7.68E-07 | 1.74E-06 |
| PCSK1 | 2.54404 | 4.901185 | 7.88E-07 | 1.78E-06 |
| DAW1 | 2.467238 | -2.69534 | 8.18E-07 | 1.84E-06 |
| VAX1 | 3.979199 | -1.52177 | 8.30E-07 | 1.87E-06 |
| KRT13 | 2.624607 | -0.28585 | 8.64E-07 | 1.94E-06 |
| PAGE1 | 4.952005 | -1.92656 | 8.86E-07 | 1.99E-06 |
| PSAPL1 | 3.50969 | -1.67054 | 9.20E-07 | 2.06E-06 |
| IL17F | 2.16626 | -2.46231 | 9.45E-07 | 2.12E-06 |
| SPATA21 | 2.184035 | -2.82819 | 1.01E-06 | 2.25E-06 |
| WNT7A | 2.841026 | -1.67355 | 1.01E-06 | 2.25E-06 |
| CALCB | 4.251246 | 2.456263 | 1.07E-06 | 2.38E-06 |
| CD300LD | 2.264539 | -3.01345 | 1.07E-06 | 2.39E-06 |
| PRB2 | 2.778138 | -2.94552 | 1.09E-06 | 2.43E-06 |
| MSMB | 3.97947 | -1.17513 | 1.10E-06 | 2.44E-06 |
| GJB7 | 2.923258 | -1.10967 | 1.20E-06 | 2.66E-06 |
| TBR1 | 3.187721 | -3.02133 | 1.24E-06 | 2.74E-06 |
| DCDC1 | 2.016377 | -2.45311 | 1.29E-06 | 2.87E-06 |
| EN1 | 2.763472 | -2.29596 | 1.30E-06 | 2.89E-06 |
| CTAG2 | 5.665955 | -0.8846 | 1.39E-06 | 3.07E-06 |
| ZAN | 2.163172 | -3.35004 | 1.40E-06 | 3.10E-06 |
| SLURP1 | 2.913161 | -3.47074 | 1.48E-06 | 3.25E-06 |
| RSPO4 | 2.69446 | -0.42931 | 1.49E-06 | 3.29E-06 |
| CALML5 | 3.994411 | -2.36477 | 1.57E-06 | 3.45E-06 |
| CLPSL1 | 3.451397 | -3.39714 | 1.70E-06 | 3.73E-06 |
| KRT3 | 2.679219 | -3.48796 | 1.76E-06 | 3.86E-06 |
| NTF4 | 2.492684 | -2.87071 | 1.78E-06 | 3.89E-06 |
| ACP7 | 2.672315 | -2.50375 | 1.91E-06 | 4.16E-06 |
| IRX4 | 3.139954 | -3.28449 | 1.93E-06 | 4.22E-06 |
| PAX3 | 3.17178 | -2.47647 | 1.96E-06 | 4.28E-06 |
| FAM25A | 3.085664 | -3.34703 | 2.06E-06 | 4.48E-06 |
| MAGEA9B | 4.33339 | -2.8291 | 2.08E-06 | 4.53E-06 |
| MAGEB2 | 5.216458 | -0.50511 | 2.10E-06 | 4.56E-06 |
| SOX21 | 3.289406 | -2.51574 | 2.11E-06 | 4.58E-06 |
| CRCT1 | 4.413762 | -2.37985 | 2.15E-06 | 4.67E-06 |
| TKTL1 | 3.525738 | 0.769988 | 2.18E-06 | 4.74E-06 |
| FOXG1 | 3.565964 | -1.34659 | 2.25E-06 | 4.88E-06 |
| TAS2R3 | 2.99469 | -2.49513 | 2.31E-06 | 4.99E-06 |
| S100G | 3.06232 | -3.25056 | 2.39E-06 | 5.16E-06 |
| DSPP | 4.133664 | -2.83172 | 2.39E-06 | 5.16E-06 |
| KRT84 | 3.561656 | -2.15597 | 2.42E-06 | 5.22E-06 |
| UNCX | 3.734806 | -3.14084 | 2.51E-06 | 5.42E-06 |
| NKX6-1 | 2.960408 | -2.68333 | 2.81E-06 | 6.03E-06 |
| OR1J1 | 2.504351 | -3.51628 | 2.94E-06 | 6.31E-06 |
| PSG11 | 3.617529 | -3.46324 | 3.18E-06 | 6.80E-06 |
| FAM81B | 3.483674 | -2.22101 | 3.22E-06 | 6.87E-06 |
| AC109583.1 | 2.178514 | -2.12184 | 3.33E-06 | 7.10E-06 |
| SFTA3 | 4.467957 | -2.07283 | 3.42E-06 | 7.28E-06 |
| BBOX1 | 2.47205 | -1.32879 | 3.70E-06 | 7.85E-06 |
| ZNF716 | 3.810878 | -2.95009 | 3.98E-06 | 8.43E-06 |
| CDH7 | 2.196469 | -1.45974 | 4.04E-06 | 8.55E-06 |
| BPIFB2 | 3.561768 | -2.02359 | 4.09E-06 | 8.65E-06 |
| REG4 | 2.373087 | 9.243571 | 4.32E-06 | 9.10E-06 |
| TMPRSS11D | 3.358815 | -2.57062 | 4.34E-06 | 9.15E-06 |
| BANF2 | 2.099509 | -3.54017 | 4.74E-06 | 9.95E-06 |
| ALX1 | 2.993754 | -2.19153 | 4.75E-06 | 9.97E-06 |
| TMEM213 | 2.445689 | -1.77675 | 5.09E-06 | 1.07E-05 |
| TAS2R13 | 3.457868 | -2.20952 | 5.19E-06 | 1.09E-05 |
| FGL1 | 3.221861 | -1.04449 | 5.39E-06 | 1.13E-05 |
| LIN28B | 4.804875 | -1.4088 | 5.45E-06 | 1.14E-05 |
| MAGEB6 | 3.121413 | -3.56837 | 5.48E-06 | 1.15E-05 |
| INSYN2B | 2.018716 | -1.9602 | 5.63E-06 | 1.18E-05 |
| REG3A | 3.188919 | 7.658554 | 5.82E-06 | 1.22E-05 |
| CT45A1 | 4.69524 | -2.66568 | 5.95E-06 | 1.24E-05 |
| SSX1 | 3.242727 | -3.57462 | 6.37E-06 | 1.32E-05 |
| CT45A10 | 4.184945 | -2.95978 | 6.42E-06 | 1.33E-05 |
| PSG6 | 3.763921 | -3.38935 | 6.46E-06 | 1.34E-05 |
| DCAF4L2 | 4.223012 | -2.89373 | 6.91E-06 | 1.43E-05 |
| CNPY1 | 2.546148 | -3.31903 | 7.23E-06 | 1.49E-05 |
| GREB1L | 2.005493 | 0.435635 | 7.23E-06 | 1.49E-05 |
| CASP14 | 2.814686 | -3.60596 | 7.27E-06 | 1.50E-05 |
| FRMPD2 | 2.155013 | -2.82779 | 8.24E-06 | 1.69E-05 |
| CCDC83 | 2.507395 | -3.12835 | 8.61E-06 | 1.76E-05 |
| KRTAP3-3 | 2.373002 | -3.10493 | 9.55E-06 | 1.94E-05 |
| BRDT | 2.274273 | -3.40768 | 1.01E-05 | 2.05E-05 |
| SALL3 | 3.848254 | -2.57648 | 1.04E-05 | 2.11E-05 |
| OR51B5 | 2.889603 | -2.7779 | 1.15E-05 | 2.33E-05 |
| CSAG2 | 3.59812 | -2.9891 | 1.17E-05 | 2.37E-05 |
| PAGE2 | 3.285192 | -3.16456 | 1.34E-05 | 2.69E-05 |
| KCNE1B | 3.357092 | -1.78654 | 1.54E-05 | 3.08E-05 |
| TREML4 | 2.376154 | -2.50996 | 1.72E-05 | 3.40E-05 |
| SERPINB13 | 4.420738 | -2.26932 | 1.75E-05 | 3.47E-05 |
| CDH16 | 2.163109 | 1.326303 | 1.86E-05 | 3.67E-05 |
| SPRR2G | 3.739154 | -3.36116 | 1.89E-05 | 3.73E-05 |
| PPP4R3C | 3.75522 | -3.35462 | 1.98E-05 | 3.90E-05 |
| GPR52 | 3.080732 | -2.46792 | 2.27E-05 | 4.44E-05 |
| EPPIN | 2.701635 | -2.8161 | 2.27E-05 | 4.45E-05 |
| ADAM2 | 2.679435 | -3.32967 | 2.31E-05 | 4.50E-05 |
| NKX6-3 | 3.438812 | 0.037078 | 2.31E-05 | 4.51E-05 |
| CRP | 2.916127 | -2.94854 | 2.32E-05 | 4.52E-05 |
| SULT1C3 | 2.139691 | -0.46614 | 2.38E-05 | 4.65E-05 |
| BOLL | 2.450668 | -3.01399 | 2.64E-05 | 5.14E-05 |
| FLG2 | 3.213197 | -3.13013 | 2.67E-05 | 5.19E-05 |
| ANXA8 | 2.851904 | -1.23851 | 2.70E-05 | 5.25E-05 |
| PSG7 | 3.509521 | -3.40602 | 3.30E-05 | 6.34E-05 |
| TGM6 | 3.362728 | -2.55759 | 3.30E-05 | 6.35E-05 |
| PYDC1 | 2.27228 | -2.80487 | 3.36E-05 | 6.46E-05 |
| RGS21 | 2.710737 | -3.53318 | 3.53E-05 | 6.76E-05 |
| PSG1 | 3.69933 | -3.1424 | 3.72E-05 | 7.11E-05 |
| HBZ | 3.989977 | -2.52935 | 3.78E-05 | 7.22E-05 |
| CLDN10 | 2.448948 | -0.06176 | 4.04E-05 | 7.70E-05 |
| MRLN | 2.561761 | -0.78641 | 4.14E-05 | 7.86E-05 |
| REG3G | 3.476444 | -0.59444 | 4.17E-05 | 7.93E-05 |
| TMPRSS11A | 3.037161 | -3.43792 | 4.21E-05 | 8.01E-05 |
| TUBA3C | 3.139785 | -3.27469 | 4.83E-05 | 9.13E-05 |
| ADAM20 | 2.064592 | -2.3139 | 4.88E-05 | 9.21E-05 |
| BPIFB1 | 3.088438 | -1.64005 | 4.89E-05 | 9.24E-05 |
| LIPF | 3.178984 | -3.07512 | 4.95E-05 | 9.35E-05 |
| ZNF479 | 3.39416 | -3.37889 | 5.25E-05 | 9.89E-05 |
| NAA11 | 2.670532 | -3.22243 | 5.28E-05 | 9.94E-05 |
| ALPP | 2.097702 | -0.23027 | 5.33E-05 | 0.000100298 |
| INS | 3.060394 | -3.51307 | 5.38E-05 | 0.000101156 |
| IL36B | 2.647133 | -2.27679 | 5.51E-05 | 0.000103435 |
| SLC38A8 | 2.205727 | -2.86463 | 5.65E-05 | 0.000105927 |
| PLET1 | 2.590026 | -3.42178 | 6.07E-05 | 0.000113663 |
| IFNK | 3.816618 | -2.16929 | 6.18E-05 | 0.000115534 |
| DAZ1 | 3.52882 | -3.50073 | 6.20E-05 | 0.00011595 |
| LCE3D | 3.175258 | -3.61081 | 6.54E-05 | 0.000122078 |
| SPINK7 | 2.015397 | -3.19426 | 6.65E-05 | 0.000123863 |
| MUC7 | 2.856876 | -2.912 | 7.08E-05 | 0.000131628 |
| CCDC190 | 2.642798 | -1.77849 | 7.08E-05 | 0.000131669 |
| APOA2 | 2.759707 | 0.488941 | 8.41E-05 | 0.000155125 |
| OR1N2 | 3.359597 | -3.3235 | 9.20E-05 | 0.000169151 |
| KRTAP4-6 | 2.979852 | -3.6198 | 9.33E-05 | 0.000171351 |
| SAA2-SAA4 | 2.085771 | -0.49665 | 9.65E-05 | 0.000176991 |
| TMCO2 | 2.162424 | -3.48065 | 9.85E-05 | 0.000180438 |
| AC011473.4 | 2.622568 | -3.69459 | 0.000106396 | 0.000193902 |
| CRNN | 2.919129 | -3.70757 | 0.000113735 | 0.000206862 |
| MAGEB1 | 3.062903 | -3.65655 | 0.000115222 | 0.000209414 |
| LGALS7 | 2.051162 | -3.58636 | 0.000118708 | 0.000215438 |
| AMBN | 2.787322 | -2.90131 | 0.000119566 | 0.000216906 |
| NPVF | 3.31022 | -3.54585 | 0.000119841 | 0.000217382 |
| TAAR1 | 2.717856 | -3.57273 | 0.00012502 | 0.000226193 |
| BPIFA1 | 3.720757 | -2.22415 | 0.000126755 | 0.00022912 |
| CAPSL | 2.179885 | -3.25668 | 0.000132095 | 0.000238357 |
| PRLHR | 2.449125 | -2.85327 | 0.000154912 | 0.00027762 |
| OR2T10 | 2.884083 | -3.42433 | 0.00015717 | 0.000281552 |
| PRSS55 | 2.210446 | -3.49351 | 0.000158527 | 0.000283866 |
| SBK2 | 2.646665 | -3.49891 | 0.000165422 | 0.000295551 |
| GABRR1 | 2.102251 | -0.93896 | 0.000180801 | 0.000321655 |
| FBXO40 | 3.378502 | -1.20437 | 0.000184892 | 0.000328501 |
| H2AP | 2.441341 | -2.67899 | 0.000195648 | 0.000346947 |
| KRT14 | 3.091577 | 2.055019 | 0.000205236 | 0.000363034 |
| SFTPB | 2.135319 | 0.492257 | 0.000214931 | 0.000379382 |
| WFDC8 | 2.333489 | -3.50605 | 0.000220787 | 0.000388937 |
| TAS2R46 | 2.316904 | -2.89205 | 0.000231697 | 0.000406974 |
| GPR22 | 2.279109 | -0.69821 | 0.000254143 | 0.000444757 |
| CTCFL | 2.139885 | -1.86939 | 0.000289003 | 0.000502413 |
| TAS2R45 | 2.158576 | -2.73827 | 0.000308768 | 0.000534554 |
| TAS2R50 | 2.332738 | -2.69687 | 0.000367602 | 0.000631251 |
| SPRR2F | 3.417316 | -1.37665 | 0.000381343 | 0.000653577 |
| PSG9 | 2.694682 | -3.28095 | 0.000389621 | 0.000667174 |
| GABRA3 | 2.127319 | 0.065186 | 0.000432063 | 0.00073534 |
| PRB4 | 2.609016 | -3.3991 | 0.000467601 | 0.000792753 |
| H1-1 | 2.881123 | -3.4735 | 0.000475313 | 0.000805052 |
| VCX | 2.160132 | -3.62853 | 0.000486712 | 0.000823725 |
| CER1 | 2.236033 | -2.42579 | 0.000526634 | 0.000887786 |
| SCRT2 | 2.063053 | -3.56279 | 0.000561605 | 0.000944025 |
| OR10A2 | 2.229062 | -3.50894 | 0.00057176 | 0.000960361 |
| NEUROD4 | 2.865729 | -3.54944 | 0.000580972 | 0.000975367 |
| TRPC5 | 2.157751 | -3.13878 | 0.000614411 | 0.001027387 |
| TSPYL6 | 2.351005 | -2.68496 | 0.000920873 | 0.00150913 |
| PNLIP | 3.303484 | -2.56116 | 0.000945085 | 0.001546935 |
| DLK1 | 2.566774 | -1.68888 | 0.001094257 | 0.001778522 |
| GPR50 | 2.363967 | -3.63391 | 0.001280281 | 0.002062381 |
| H4C6 | 2.45873 | -2.05551 | 0.002251009 | 0.003519716 |
| KCNC2 | 2.244392 | -1.42113 | 0.002452946 | 0.003818492 |
| MLN | 2.058428 | -2.4464 | 0.003301111 | 0.00505471 |
| GALP | 2.274122 | -3.66017 | 0.003351376 | 0.005127212 |
| CPA1 | 2.036099 | -2.18172 | 0.003728889 | 0.005667783 |
| DEFB126 | 2.027098 | -3.51702 | 0.003940362 | 0.005969094 |
| EZHIP | 2.08856 | -2.92392 | 0.007243722 | 0.01063606 |
| BEST4 | -5.87294 | 3.013125 | 0 | 0 |
| CUBN | -5.05342 | 1.482111 | 1.41E-282 | 1.24E-278 |
| CLEC3B | -4.2101 | 2.51047 | 7.19E-249 | 4.21E-245 |
| SLC51A | -4.24862 | 3.218713 | 1.34E-247 | 5.87E-244 |
| CPO | -6.22734 | -0.27455 | 8.69E-244 | 3.05E-240 |
| SLC25A34 | -4.03107 | 1.390415 | 5.23E-238 | 1.53E-234 |
| ABCG2 | -4.82946 | 3.207466 | 3.90E-219 | 9.80E-216 |
| PHLPP2 | -2.62539 | 4.699858 | 5.01E-205 | 1.10E-201 |
| TEX11 | -4.13493 | -0.23196 | 6.54E-203 | 1.28E-199 |
| CA7 | -5.6649 | 3.40799 | 6.48E-196 | 1.14E-192 |
| UGP2 | -2.12533 | 6.927201 | 6.53E-192 | 1.04E-188 |
| MGAM | -5.12665 | 2.089418 | 5.76E-188 | 8.44E-185 |
| MS4A10 | -7.42454 | -0.07961 | 1.92E-185 | 2.60E-182 |
| USP2 | -3.99143 | 2.480506 | 2.82E-185 | 3.55E-182 |
| APOA1 | -7.43115 | 3.603699 | 2.84E-184 | 3.33E-181 |
| OTOP2 | -8.21108 | 2.986885 | 3.71E-184 | 4.08E-181 |
| PRKG2 | -4.00344 | 1.066119 | 6.60E-184 | 6.83E-181 |
| CA2 | -4.81044 | 7.268832 | 2.52E-182 | 2.21E-179 |
| DAO | -5.6644 | -1.08077 | 8.36E-182 | 7.00E-179 |
| MEP1B | -5.63907 | 2.131762 | 5.55E-172 | 4.44E-169 |
| AQP8 | -7.02759 | 6.642307 | 2.21E-161 | 1.62E-158 |
| SPIB | -4.69379 | 2.773742 | 1.11E-156 | 7.80E-154 |
| GLP2R | -3.9133 | 0.873972 | 1.03E-155 | 6.97E-153 |
| PDCD4 | -2.04815 | 6.799957 | 1.22E-155 | 7.94E-153 |
| C2orf88 | -3.59865 | 4.080445 | 1.35E-154 | 8.50E-152 |
| GCNT2 | -3.77902 | 1.845755 | 1.69E-153 | 9.92E-151 |
| IL6R | -2.79636 | 4.175224 | 2.56E-153 | 1.45E-150 |
| SULT1A2 | -3.84208 | 1.590462 | 2.72E-153 | 1.49E-150 |
| SCARA5 | -4.74528 | 3.445899 | 3.40E-153 | 1.81E-150 |
| SLC51B | -4.1097 | 3.372706 | 4.14E-153 | 2.14E-150 |
| PKIB | -3.9418 | 4.288805 | 1.22E-152 | 6.14E-150 |
| CDKN2B | -3.28025 | 4.687649 | 6.16E-151 | 2.93E-148 |
| ABCB11 | -5.05505 | -0.6065 | 5.73E-147 | 2.65E-144 |
| DHRS7C | -4.9597 | -3.19162 | 3.90E-145 | 1.72E-142 |
| ASPA | -3.91884 | 0.065805 | 8.67E-143 | 3.63E-140 |
| TAT | -3.83419 | -1.29763 | 1.27E-142 | 5.18E-140 |
| CLDN23 | -2.98917 | 4.677466 | 4.20E-142 | 1.68E-139 |
| PLP1 | -5.40244 | 1.593616 | 3.03E-141 | 1.18E-138 |
| FAM107A | -3.51745 | 2.266182 | 3.25E-140 | 1.24E-137 |
| GUCA2A | -5.17497 | 5.900932 | 1.61E-139 | 6.03E-137 |
| RBFOX3 | -4.87339 | 1.223187 | 1.15E-137 | 4.14E-135 |
| NAALADL1 | -3.38487 | 2.987569 | 4.63E-137 | 1.63E-134 |
| BMP3 | -5.28473 | 2.743084 | 5.06E-137 | 1.74E-134 |
| LYVE1 | -4.13279 | 2.726837 | 8.52E-137 | 2.88E-134 |
| SCGN | -4.9618 | 0.94221 | 1.02E-136 | 3.39E-134 |
| PDK4 | -3.35449 | 4.934629 | 4.22E-134 | 1.35E-131 |
| TNXB | -4.00972 | 3.997155 | 2.85E-133 | 8.96E-131 |
| CSRP1 | -2.26348 | 8.223789 | 1.48E-132 | 4.58E-130 |
| ADAMDEC1 | -3.62029 | 5.468113 | 7.05E-132 | 2.14E-129 |
| PTGS1 | -2.91526 | 4.597373 | 1.31E-131 | 3.84E-129 |
| SMPDL3A | -2.62701 | 4.745937 | 2.25E-131 | 6.48E-129 |
| VEGFD | -4.40011 | -0.74171 | 2.92E-131 | 8.29E-129 |
| GFRA2 | -3.66582 | 0.416182 | 6.01E-131 | 1.68E-128 |
| PYGM | -3.89439 | 1.181353 | 3.05E-130 | 8.39E-128 |
| SLC30A10 | -5.7105 | 1.126538 | 1.53E-129 | 4.15E-127 |
| DPP6 | -4.98506 | 0.473838 | 3.59E-129 | 9.58E-127 |
| TRPM6 | -4.18715 | 4.038517 | 5.56E-129 | 1.46E-126 |
| SLC26A2 | -4.16408 | 7.953435 | 1.17E-128 | 2.99E-126 |
| CA1 | -6.62423 | 6.512352 | 1.66E-128 | 4.18E-126 |
| CCDC69 | -2.89856 | 4.755899 | 5.20E-127 | 1.29E-124 |
| GUCA2B | -6.19098 | 3.41508 | 4.55E-125 | 1.11E-122 |
| MAMDC2 | -4.68111 | 1.738529 | 7.69E-125 | 1.85E-122 |
| CMTM5 | -4.64868 | -2.44024 | 8.02E-125 | 1.91E-122 |
| SGK1 | -2.78931 | 5.856227 | 1.11E-124 | 2.59E-122 |
| ABCA8 | -4.47278 | 2.274605 | 3.45E-123 | 7.87E-121 |
| ENTPD5 | -2.57828 | 6.304583 | 6.79E-123 | 1.53E-120 |
| TMEM100 | -3.87416 | 0.717777 | 2.60E-122 | 5.78E-120 |
| AHCYL2 | -2.71457 | 6.901118 | 6.45E-122 | 1.40E-119 |
| SLC4A4 | -4.5909 | 5.08421 | 1.75E-120 | 3.76E-118 |
| ENPP6 | -3.44036 | -0.97524 | 3.03E-120 | 6.42E-118 |
| RXRG | -3.9584 | -1.52538 | 4.19E-120 | 8.78E-118 |
| PLCD1 | -2.26281 | 4.568602 | 5.85E-119 | 1.20E-116 |
| POPDC2 | -3.43918 | 2.02041 | 7.39E-119 | 1.49E-116 |
| TMEM37 | -3.06184 | 4.624417 | 4.35E-118 | 8.60E-116 |
| TP53INP2 | -2.56898 | 5.877501 | 4.59E-118 | 8.97E-116 |
| TMEM220 | -3.02894 | 2.115006 | 2.34E-117 | 4.52E-115 |
| ZNF575 | -2.09923 | 2.106052 | 7.42E-117 | 1.42E-114 |
| TMEM72 | -4.29882 | 1.647763 | 7.56E-117 | 1.43E-114 |
| TMEM253 | -2.99108 | 0.92749 | 6.74E-116 | 1.26E-113 |
| NPTX1 | -4.94905 | 1.820954 | 1.24E-114 | 2.29E-112 |
| TMIGD1 | -6.24738 | 3.475334 | 1.66E-114 | 3.03E-112 |
| RERGL | -5.13799 | -0.8416 | 3.58E-114 | 6.49E-112 |
| TMCC3 | -2.44508 | 4.966836 | 8.29E-114 | 1.49E-111 |
| UGT1A8 | -4.37556 | 1.026147 | 1.32E-113 | 2.35E-111 |
| G6PC | -6.30631 | -1.13432 | 2.15E-113 | 3.74E-111 |
| CD177 | -5.48084 | 6.05605 | 3.57E-113 | 6.15E-111 |
| PLAC9 | -2.57928 | 1.305696 | 2.01E-112 | 3.43E-110 |
| NAAA | -2.09475 | 5.281328 | 2.11E-112 | 3.57E-110 |
| ATP1A2 | -4.89503 | 2.407166 | 9.01E-112 | 1.51E-109 |
| LRRN2 | -3.65512 | 2.711061 | 1.09E-111 | 1.80E-109 |
| VSTM2A | -5.16435 | -0.44069 | 6.65E-111 | 1.08E-108 |
| FHL1 | -3.30147 | 5.368357 | 7.35E-111 | 1.19E-108 |
| MYOM1 | -3.25019 | 1.784566 | 9.63E-111 | 1.54E-108 |
| GRIK3 | -3.58542 | 0.475617 | 2.51E-110 | 3.95E-108 |
| MPZ | -2.82477 | 0.827422 | 4.59E-110 | 7.15E-108 |
| ALPI | -4.35768 | 2.643271 | 6.56E-110 | 1.01E-107 |
| SLC17A8 | -5.27038 | -1.74666 | 4.31E-109 | 6.54E-107 |
| TMEM35A | -4.0193 | 1.473248 | 4.32E-109 | 6.54E-107 |
| DPT | -4.00298 | 3.993857 | 7.54E-109 | 1.13E-106 |
| SEMA6D | -3.08745 | 3.47158 | 1.91E-108 | 2.85E-106 |
| MT1M | -4.37213 | 3.007605 | 5.32E-108 | 7.86E-106 |
| CYP3A4 | -5.10245 | 1.961674 | 6.47E-108 | 9.48E-106 |
| PLCE1 | -2.06712 | 5.042072 | 1.65E-107 | 2.37E-105 |
| HPGDS | -3.1786 | 0.285912 | 2.19E-107 | 3.13E-105 |
| CD163L1 | -3.02387 | 3.176602 | 7.04E-107 | 9.90E-105 |
| LDB3 | -4.03793 | 1.666003 | 1.32E-106 | 1.84E-104 |
| FAM180B | -4.45854 | -2.64076 | 2.58E-106 | 3.54E-104 |
| ADHFE1 | -2.84273 | -0.23396 | 3.96E-106 | 5.39E-104 |
| CADM3 | -4.60492 | 1.563391 | 5.83E-106 | 7.89E-104 |
| PYY | -5.7278 | 3.123707 | 6.45E-106 | 8.66E-104 |
| APOB | -6.75606 | 3.977465 | 8.00E-106 | 1.07E-103 |
| HSPB8 | -3.64853 | 4.000164 | 1.54E-104 | 2.03E-102 |
| METTL7A | -2.51848 | 5.887744 | 1.80E-104 | 2.37E-102 |
| MYLK | -3.33258 | 7.393511 | 3.02E-103 | 3.90E-101 |
| TMEM236 | -3.6584 | 4.073015 | 1.22E-102 | 1.56E-100 |
| CREB3L3 | -4.41506 | 1.926145 | 2.10E-102 | 2.68E-100 |
| NIBAN1 | -3.09621 | 4.488989 | 2.40E-102 | 3.03E-100 |
| GSTM5 | -2.9704 | 0.777067 | 9.16E-102 | 1.14E-99 |
| SYNM | -4.29226 | 5.798397 | 1.82E-101 | 2.25E-99 |
| MYH11 | -4.47364 | 9.295416 | 4.63E-101 | 5.61E-99 |
| CNN1 | -4.15452 | 6.730503 | 1.10E-100 | 1.33E-98 |
| ENPEP | -3.04583 | 3.062235 | 1.18E-100 | 1.41E-98 |
| MYL9 | -3.21929 | 8.19446 | 7.29E-100 | 8.66E-98 |
| GSTA2 | -4.91149 | -1.22778 | 9.91E-100 | 1.17E-97 |
| MAL | -3.57174 | -0.03055 | 2.42E-99 | 2.84E-97 |
| AMPD1 | -4.05932 | -0.35012 | 3.15E-99 | 3.67E-97 |
| MT1F | -2.99275 | 3.838325 | 1.86E-98 | 2.12E-96 |
| SH2D7 | -3.76316 | 0.001302 | 2.60E-98 | 2.94E-96 |
| NKX2-3 | -2.82813 | 2.311419 | 5.36E-98 | 6.04E-96 |
| PDE2A | -2.68036 | 2.276192 | 4.73E-97 | 5.24E-95 |
| LGI1 | -5.09266 | -1.6964 | 5.83E-97 | 6.41E-95 |
| GNAO1 | -3.51627 | 2.695762 | 1.04E-96 | 1.14E-94 |
| PSD | -3.28789 | 3.225018 | 1.38E-96 | 1.49E-94 |
| LIFR | -3.26315 | 2.393128 | 1.59E-96 | 1.71E-94 |
| C1orf115 | -2.4258 | 5.248308 | 3.10E-96 | 3.30E-94 |
| NR3C2 | -2.70337 | 4.888546 | 4.07E-96 | 4.31E-94 |
| PRIMA1 | -4.63897 | 1.364456 | 1.10E-95 | 1.15E-93 |
| ACTG2 | -4.15858 | 8.117568 | 2.22E-95 | 2.31E-93 |
| CEACAM7 | -4.31622 | 8.601375 | 3.07E-95 | 3.18E-93 |
| CAVIN2 | -3.05552 | 3.426983 | 6.16E-95 | 6.34E-93 |
| NAP1L2 | -3.62728 | -0.44461 | 2.39E-94 | 2.42E-92 |
| SLC10A2 | -7.75993 | 0.542633 | 1.60E-93 | 1.61E-91 |
| FAM151A | -2.37706 | 0.881785 | 2.52E-93 | 2.52E-91 |
| TRANK1 | -2.1367 | 5.339649 | 2.98E-93 | 2.96E-91 |
| KLF4 | -2.50876 | 6.729214 | 5.24E-93 | 5.17E-91 |
| SCN7A | -4.86071 | 1.748458 | 1.09E-92 | 1.06E-90 |
| UGT1A10 | -3.70709 | 2.954243 | 1.31E-92 | 1.28E-90 |
| APOA4 | -8.62294 | 2.723024 | 3.81E-92 | 3.67E-90 |
| APOC3 | -7.65774 | 0.920945 | 1.07E-91 | 1.01E-89 |
| RBPMS2 | -3.41396 | 2.11826 | 1.37E-91 | 1.30E-89 |
| GPAT3 | -2.51309 | 3.380782 | 2.23E-91 | 2.10E-89 |
| RDH5 | -2.2776 | 1.36337 | 3.72E-91 | 3.48E-89 |
| STMN4 | -4.33682 | -2.12272 | 6.67E-91 | 6.21E-89 |
| HSD3B2 | -5.83575 | 1.219197 | 8.69E-91 | 8.00E-89 |
| SELENOP | -3.20911 | 6.846843 | 1.61E-90 | 1.47E-88 |
| RNF152 | -2.80843 | 3.286999 | 7.63E-90 | 6.95E-88 |
| HPSE2 | -3.30929 | 0.078271 | 1.18E-89 | 1.06E-87 |
| MT1H | -3.97049 | 2.858274 | 1.85E-89 | 1.66E-87 |
| FLNC | -3.41418 | 6.343942 | 2.42E-89 | 2.15E-87 |
| NEGR1 | -2.97103 | 2.522816 | 3.00E-89 | 2.66E-87 |
| TACR2 | -3.80229 | 2.889705 | 1.72E-88 | 1.50E-86 |
| SLC26A3 | -4.61813 | 8.81525 | 2.88E-88 | 2.50E-86 |
| BCHE | -4.20982 | 1.00405 | 3.21E-88 | 2.77E-86 |
| MT1G | -3.45937 | 5.838739 | 3.76E-88 | 3.22E-86 |
| LMOD1 | -3.55484 | 5.42413 | 5.01E-88 | 4.28E-86 |
| ADH1C | -4.15274 | 5.78637 | 1.29E-87 | 1.09E-85 |
| SLC5A11 | -3.62039 | -0.82455 | 2.15E-87 | 1.81E-85 |
| CFL2 | -2.53673 | 3.91575 | 2.58E-87 | 2.16E-85 |
| SST | -5.15754 | 0.98034 | 3.26E-87 | 2.72E-85 |
| PADI2 | -3.29017 | 6.615008 | 3.69E-87 | 3.06E-85 |
| CLCA4 | -5.4408 | 6.852891 | 4.83E-87 | 3.99E-85 |
| SLC17A7 | -2.94735 | -0.95433 | 4.87E-87 | 4.00E-85 |
| TSPAN7 | -2.78078 | 4.420219 | 5.80E-86 | 4.73E-84 |
| MS4A12 | -5.33359 | 5.341287 | 6.45E-86 | 5.23E-84 |
| SGCG | -4.08364 | -2.45411 | 7.92E-86 | 6.36E-84 |
| SLC6A19 | -5.44768 | 3.199446 | 8.29E-86 | 6.62E-84 |
| ECRG4 | -4.18144 | -0.19347 | 1.54E-85 | 1.22E-83 |
| DHRS11 | -2.30019 | 5.677241 | 1.61E-85 | 1.27E-83 |
| ELANE | -3.55489 | -2.4195 | 2.33E-85 | 1.84E-83 |
| OTOP3 | -6.00916 | -1.55684 | 3.12E-85 | 2.45E-83 |
| PTN | -2.66921 | 1.809403 | 5.66E-85 | 4.42E-83 |
| SCN9A | -3.43851 | 1.602326 | 6.24E-85 | 4.85E-83 |
| ABI3BP | -3.13501 | 3.348123 | 2.15E-84 | 1.65E-82 |
| PIRT | -4.14483 | -0.33424 | 2.37E-84 | 1.81E-82 |
| FXYD1 | -3.22401 | -0.89462 | 2.61E-84 | 1.99E-82 |
| SVIL | -2.0109 | 6.492172 | 4.37E-84 | 3.31E-82 |
| NCAM1 | -2.97825 | 2.575218 | 6.31E-84 | 4.76E-82 |
| GREM2 | -3.83009 | 2.888915 | 7.43E-84 | 5.59E-82 |
| LDHD | -2.98961 | 4.089792 | 1.01E-83 | 7.52E-82 |
| TMEM151B | -3.29553 | -1.65678 | 2.79E-83 | 2.07E-81 |
| SLC22A18AS | -2.35827 | 3.260594 | 3.53E-83 | 2.61E-81 |
| HSD17B2 | -3.46303 | 4.81079 | 4.00E-83 | 2.94E-81 |
| CHP2 | -4.00584 | 5.615887 | 4.13E-83 | 3.02E-81 |
| PDZD4 | -3.1897 | 0.990916 | 4.60E-83 | 3.35E-81 |
| FBXL22 | -2.36973 | 1.140602 | 6.75E-83 | 4.86E-81 |
| NTN1 | -2.75342 | 2.891662 | 2.34E-82 | 1.68E-80 |
| NR5A2 | -2.31185 | 3.860784 | 2.41E-82 | 1.71E-80 |
| ST6GALNAC6 | -2.5623 | 5.742364 | 2.61E-82 | 1.85E-80 |
| CA4 | -4.95161 | 5.624248 | 3.31E-82 | 2.34E-80 |
| SLC17A4 | -3.33614 | 3.07145 | 5.57E-82 | 3.92E-80 |
| ANK2 | -3.10897 | 2.775951 | 1.18E-81 | 8.28E-80 |
| SLC2A4 | -2.69649 | 2.404485 | 1.94E-81 | 1.35E-79 |
| PRPH | -3.77527 | 0.327191 | 4.44E-81 | 3.08E-79 |
| FAM135B | -4.15336 | -1.27598 | 7.29E-81 | 4.99E-79 |
| CNGB1 | -3.56009 | -2.21198 | 9.02E-81 | 6.13E-79 |
| GNG7 | -2.62423 | 1.985154 | 2.15E-80 | 1.46E-78 |
| SYNPO2 | -3.73111 | 6.398517 | 3.71E-80 | 2.49E-78 |
| ACADS | -2.15458 | 6.114608 | 5.08E-80 | 3.40E-78 |
| SCN11A | -3.46611 | -1.22257 | 5.79E-80 | 3.86E-78 |
| CEACAM1 | -2.30973 | 8.214827 | 6.91E-80 | 4.59E-78 |
| ASB2 | -2.95208 | 2.691637 | 7.67E-80 | 5.07E-78 |
| CHAD | -2.93784 | 0.81136 | 7.71E-80 | 5.08E-78 |
| PBLD | -2.33258 | 4.798966 | 9.16E-80 | 6.01E-78 |
| HAND2 | -3.93375 | 2.071097 | 1.07E-79 | 6.97E-78 |
| SCNN1B | -4.04734 | 4.073921 | 1.13E-79 | 7.34E-78 |
| SLC9A3 | -3.8243 | 5.760677 | 1.66E-79 | 1.08E-77 |
| PDZRN4 | -4.08761 | 1.026735 | 3.79E-79 | 2.44E-77 |
| CPM | -2.52862 | 4.480937 | 3.80E-79 | 2.44E-77 |
| GDPD2 | -3.27411 | 2.143795 | 6.28E-79 | 3.97E-77 |
| WSCD1 | -3.06537 | 3.315353 | 6.73E-79 | 4.24E-77 |
| NSG2 | -4.05559 | -0.21488 | 1.10E-78 | 6.89E-77 |
| PDE6A | -3.4721 | 0.939339 | 1.55E-78 | 9.72E-77 |
| NRXN1 | -4.61553 | 0.212722 | 2.32E-78 | 1.45E-76 |
| TPH1 | -3.61186 | 0.989592 | 2.52E-78 | 1.57E-76 |
| SHISAL1 | -3.15789 | 1.881797 | 3.73E-78 | 2.30E-76 |
| ADCYAP1R1 | -4.16789 | -0.45109 | 4.22E-78 | 2.60E-76 |
| AFF3 | -3.03435 | 1.018176 | 6.52E-78 | 3.99E-76 |
| HLF | -2.6088 | 1.689533 | 7.97E-78 | 4.86E-76 |
| TM6SF2 | -3.18253 | 0.616835 | 9.07E-78 | 5.52E-76 |
| CXCL12 | -2.6415 | 5.025355 | 9.43E-78 | 5.72E-76 |
| SECTM1 | -2.96683 | 5.301046 | 9.47E-78 | 5.72E-76 |
| PPP1R12B | -2.63564 | 6.323475 | 1.85E-77 | 1.11E-75 |
| PDE9A | -2.59569 | 4.916357 | 2.79E-77 | 1.68E-75 |
| CCL23 | -3.20185 | -0.9337 | 3.13E-77 | 1.87E-75 |
| MT1X | -2.40089 | 4.595517 | 5.22E-77 | 3.11E-75 |
| ATP6V1G2 | -2.41365 | -1.04057 | 6.58E-77 | 3.91E-75 |
| PGM5 | -3.44216 | 4.430788 | 9.20E-77 | 5.45E-75 |
| TRIM40 | -3.0855 | 1.657429 | 2.56E-76 | 1.51E-74 |
| MAB21L2 | -3.27884 | 3.914015 | 2.71E-76 | 1.59E-74 |
| KCNMB1 | -3.26052 | 3.276778 | 2.86E-76 | 1.68E-74 |
| SLC28A1 | -4.2276 | -1.57023 | 5.56E-76 | 3.22E-74 |
| PCSK5 | -2.47281 | 4.094144 | 6.39E-76 | 3.70E-74 |
| DNAJB5 | -2.49248 | 2.741834 | 7.66E-76 | 4.42E-74 |
| PHOX2B | -4.60052 | -1.55618 | 8.95E-76 | 5.15E-74 |
| TENT5B | -3.00198 | 1.260523 | 2.15E-75 | 1.23E-73 |
| EMILIN3 | -2.64539 | -1.05126 | 3.45E-75 | 1.96E-73 |
| ARL14 | -2.40812 | 3.457559 | 9.84E-75 | 5.55E-73 |
| UNC5C | -2.33062 | 1.854775 | 1.18E-74 | 6.62E-73 |
| TMEFF2 | -4.04163 | -2.16788 | 1.53E-74 | 8.56E-73 |
| ELAVL4 | -3.25651 | -0.66755 | 1.68E-74 | 9.36E-73 |
| GSTA1 | -4.2076 | 1.845648 | 2.18E-74 | 1.20E-72 |
| SFRP1 | -4.14219 | 2.86972 | 2.59E-74 | 1.42E-72 |
| PI16 | -4.35453 | 1.684884 | 2.85E-74 | 1.56E-72 |
| P2RY1 | -2.50868 | 3.442384 | 4.37E-74 | 2.38E-72 |
| ACKR2 | -2.50239 | 0.3045 | 5.64E-74 | 3.06E-72 |
| SRPX | -2.7155 | 2.831814 | 8.20E-74 | 4.44E-72 |
| SMYD1 | -4.59533 | 0.046978 | 2.57E-73 | 1.39E-71 |
| P2RY14 | -2.67294 | 1.661995 | 2.77E-73 | 1.49E-71 |
| BVES | -2.76973 | 1.511871 | 2.97E-73 | 1.59E-71 |
| HHLA2 | -2.78752 | 5.543204 | 3.05E-73 | 1.63E-71 |
| NEFM | -3.65585 | -1.53027 | 4.38E-73 | 2.33E-71 |
| KHDRBS2 | -4.15973 | -2.6679 | 5.37E-73 | 2.84E-71 |
| ADH1B | -4.43185 | 4.015128 | 5.71E-73 | 3.01E-71 |
| LRRC19 | -2.81669 | 4.952448 | 1.57E-72 | 8.20E-71 |
| CPEB1 | -3.22154 | -0.63463 | 1.94E-72 | 1.01E-70 |
| ADAMTSL3 | -3.20665 | 1.892146 | 2.09E-72 | 1.09E-70 |
| SCN2B | -3.70705 | -0.58511 | 2.16E-72 | 1.12E-70 |
| METTL24 | -3.302 | -0.87307 | 2.49E-72 | 1.29E-70 |
| RNF150 | -3.21253 | 2.350526 | 3.04E-72 | 1.57E-70 |
| EDN3 | -3.28685 | 3.558656 | 9.06E-72 | 4.66E-70 |
| PLAAT2 | -3.24575 | 1.165241 | 9.22E-72 | 4.73E-70 |
| JAM2 | -2.49025 | 2.675916 | 9.58E-72 | 4.90E-70 |
| NGB | -4.82522 | -2.07412 | 1.06E-71 | 5.41E-70 |
| PTPRH | -2.08635 | 5.80173 | 1.53E-71 | 7.76E-70 |
| C16orf89 | -3.52612 | 0.691238 | 2.51E-71 | 1.26E-69 |
| TMEM82 | -3.29081 | 1.714699 | 7.00E-71 | 3.51E-69 |
| WDR78 | -2.31773 | 2.029491 | 9.12E-71 | 4.54E-69 |
| KCNN3 | -2.32186 | 2.195171 | 1.55E-70 | 7.69E-69 |
| TPM2 | -2.6157 | 7.555862 | 1.62E-70 | 8.02E-69 |
| SLC23A1 | -2.94462 | 1.874906 | 1.78E-70 | 8.77E-69 |
| PKHD1L1 | -3.5003 | -0.62039 | 2.30E-70 | 1.13E-68 |
| FGL2 | -2.63245 | 4.769863 | 3.02E-70 | 1.47E-68 |
| KCNA5 | -2.60629 | -0.77589 | 3.22E-70 | 1.57E-68 |
| RSPO2 | -3.88594 | 0.35284 | 5.21E-70 | 2.52E-68 |
| CCBE1 | -3.70666 | 1.181 | 5.38E-70 | 2.60E-68 |
| SLC13A1 | -5.78314 | -1.65419 | 1.04E-69 | 4.98E-68 |
| BTNL8 | -3.29152 | 4.069195 | 1.42E-69 | 6.82E-68 |
| PLAC8 | -3.07239 | 6.076567 | 2.49E-69 | 1.19E-67 |
| CHAT | -4.53108 | -2.87613 | 2.74E-69 | 1.30E-67 |
| PAPSS2 | -2.02771 | 6.749373 | 6.21E-69 | 2.94E-67 |
| PCSK2 | -4.76476 | -0.32385 | 6.29E-69 | 2.97E-67 |
| ATP2B4 | -2.15994 | 6.225755 | 6.82E-69 | 3.21E-67 |
| P2RX2 | -3.77691 | -2.22816 | 7.24E-69 | 3.41E-67 |
| HPGD | -2.83898 | 5.54838 | 1.01E-68 | 4.70E-67 |
| PCP4L1 | -3.87779 | -0.26018 | 1.03E-68 | 4.77E-67 |
| SORCS1 | -3.74131 | 0.296759 | 1.52E-68 | 7.00E-67 |
| HSD11B2 | -2.3178 | 7.374607 | 1.78E-68 | 8.19E-67 |
| KCNE2 | -2.77552 | -2.27996 | 2.03E-68 | 9.34E-67 |
| LILRB5 | -2.6948 | 2.098601 | 2.64E-68 | 1.21E-66 |
| SLCO4C1 | -3.55245 | -0.13373 | 3.11E-68 | 1.42E-66 |
| PKNOX2 | -2.7006 | -0.31938 | 4.93E-68 | 2.25E-66 |
| C7 | -3.75907 | 4.182209 | 5.22E-68 | 2.36E-66 |
| ITM2C | -2.24959 | 9.330608 | 6.77E-68 | 3.06E-66 |
| CAND2 | -2.22759 | 0.692926 | 7.10E-68 | 3.20E-66 |
| C8orf88 | -3.0389 | -0.54162 | 1.19E-67 | 5.36E-66 |
| GFRA1 | -3.11397 | 2.225514 | 1.34E-67 | 6.00E-66 |
| NPY2R | -4.56312 | -2.05646 | 1.50E-67 | 6.70E-66 |
| CTNND2 | -3.43155 | -1.30862 | 1.84E-67 | 8.18E-66 |
| CHGA | -4.54925 | 5.12232 | 1.86E-67 | 8.24E-66 |
| SCUBE2 | -2.63383 | 2.500996 | 2.00E-67 | 8.83E-66 |
| KRTAP13-2 | -5.51404 | -1.43123 | 2.76E-67 | 1.21E-65 |
| TCF21 | -2.28527 | 2.329452 | 4.71E-67 | 2.05E-65 |
| CEACAM20 | -4.5771 | -1.62153 | 5.77E-67 | 2.51E-65 |
| ADCY5 | -2.91397 | 2.860629 | 9.57E-67 | 4.13E-65 |
| JCHAIN | -3.82996 | 9.3282 | 9.94E-67 | 4.26E-65 |
| ANPEP | -3.86721 | 8.023041 | 1.14E-66 | 4.88E-65 |
| TRPV3 | -2.92386 | 1.1598 | 1.35E-66 | 5.73E-65 |
| TAGLN | -2.91647 | 8.350886 | 1.37E-66 | 5.79E-65 |
| TMEM171 | -2.30668 | 4.336377 | 1.52E-66 | 6.41E-65 |
| TUBB4A | -3.34585 | -0.29953 | 1.63E-66 | 6.84E-65 |
| CNTN4 | -2.49301 | 1.624774 | 4.44E-66 | 1.85E-64 |
| FRMPD4 | -4.39864 | -2.0009 | 5.30E-66 | 2.20E-64 |
| SLC2A2 | -5.19079 | -1.01351 | 7.61E-66 | 3.14E-64 |
| JPH2 | -3.07871 | 3.00328 | 9.63E-66 | 3.96E-64 |
| ARHGAP44 | -2.25394 | 4.579261 | 9.72E-66 | 3.99E-64 |
| TNFRSF13B | -3.34991 | -0.38556 | 1.72E-65 | 7.00E-64 |
| LVRN | -3.22515 | -1.4135 | 2.90E-65 | 1.18E-63 |
| KCNMA1 | -3.0744 | 4.403791 | 3.81E-65 | 1.54E-63 |
| PLN | -3.41668 | 3.593619 | 7.71E-65 | 3.10E-63 |
| STMN2 | -3.36229 | 2.162438 | 8.48E-65 | 3.41E-63 |
| EML1 | -2.25725 | 3.355743 | 9.33E-65 | 3.74E-63 |
| XKR4 | -3.99019 | -1.0174 | 1.52E-64 | 6.07E-63 |
| CNR1 | -3.69485 | 0.398174 | 1.58E-64 | 6.30E-63 |
| CNTN2 | -3.79757 | -0.69489 | 2.29E-64 | 9.11E-63 |
| C15orf48 | -2.45119 | 7.261704 | 2.66E-64 | 1.05E-62 |
| SULT1A1 | -2.16929 | 4.649265 | 4.39E-64 | 1.74E-62 |
| PLCL2 | -2.41727 | 3.155393 | 9.63E-64 | 3.79E-62 |
| TNS1 | -2.74044 | 6.986565 | 1.44E-63 | 5.67E-62 |
| TLR3 | -2.06632 | 3.59106 | 1.94E-63 | 7.59E-62 |
| INSL5 | -5.76663 | 1.760894 | 2.10E-63 | 8.20E-62 |
| HSPB6 | -3.50304 | 4.018533 | 2.47E-63 | 9.58E-62 |
| MYOCD | -2.96631 | 2.754652 | 3.27E-63 | 1.26E-61 |
| CAP2 | -2.20723 | 2.497944 | 3.45E-63 | 1.33E-61 |
| PTGDR2 | -2.56627 | 1.273911 | 5.24E-63 | 2.01E-61 |
| LEXM | -2.57273 | -1.66517 | 6.06E-63 | 2.32E-61 |
| GRIK5 | -2.90393 | 0.676635 | 7.55E-63 | 2.88E-61 |
| LMO3 | -3.50017 | 2.302009 | 8.15E-63 | 3.10E-61 |
| MUSK | -3.54339 | -1.46614 | 1.48E-62 | 5.60E-61 |
| CBLN2 | -2.92216 | -0.87193 | 1.52E-62 | 5.76E-61 |
| MALL | -2.31473 | 3.84529 | 1.81E-62 | 6.80E-61 |
| SCIN | -2.69897 | 4.459439 | 1.88E-62 | 7.08E-61 |
| ARL4D | -2.27889 | 1.683141 | 1.90E-62 | 7.11E-61 |
| SSTR2 | -2.3788 | 0.571892 | 2.08E-62 | 7.78E-61 |
| EPB41L3 | -2.66891 | 4.502826 | 2.90E-62 | 1.07E-60 |
| CLIC5 | -2.10522 | 6.066819 | 2.98E-62 | 1.10E-60 |
| GPT | -2.93721 | 4.139707 | 3.04E-62 | 1.12E-60 |
| ARHGEF25 | -2.36487 | 2.988532 | 3.45E-62 | 1.27E-60 |
| MOBP | -2.94538 | -2.1483 | 3.59E-62 | 1.32E-60 |
| HMGCLL1 | -2.88184 | -2.16932 | 4.68E-62 | 1.71E-60 |
| SDCBP2 | -2.3571 | 6.085263 | 5.83E-62 | 2.13E-60 |
| LPAR1 | -2.14486 | 3.455734 | 5.93E-62 | 2.16E-60 |
| SLCO2A1 | -2.16225 | 4.951997 | 1.48E-61 | 5.36E-60 |
| SCNN1G | -4.31247 | 0.638818 | 3.02E-61 | 1.09E-59 |
| ANGPTL1 | -3.84292 | 1.904093 | 3.73E-61 | 1.35E-59 |
| LYPD8 | -3.66906 | 6.009614 | 3.81E-61 | 1.37E-59 |
| KRT24 | -5.8251 | -1.01485 | 5.16E-61 | 1.84E-59 |
| PPY | -4.28388 | -2.86419 | 5.80E-61 | 2.06E-59 |
| MYOT | -3.62815 | -1.02154 | 9.48E-61 | 3.33E-59 |
| TMOD1 | -2.83481 | 1.195368 | 9.84E-61 | 3.46E-59 |
| CTNNA3 | -3.70581 | -0.76683 | 1.16E-60 | 4.06E-59 |
| CLEC9A | -2.79649 | -1.68323 | 1.17E-60 | 4.09E-59 |
| CADM2 | -4.18532 | -0.95828 | 1.32E-60 | 4.59E-59 |
| SLC9A9 | -2.17325 | 1.908885 | 1.36E-60 | 4.73E-59 |
| GPM6A | -4.29593 | 1.19095 | 1.40E-60 | 4.84E-59 |
| ADGRB3 | -3.48837 | -1.04795 | 1.49E-60 | 5.13E-59 |
| NRG2 | -2.90353 | -1.53482 | 2.06E-60 | 7.12E-59 |
| AOC3 | -2.7302 | 5.030312 | 2.08E-60 | 7.17E-59 |
| FXYD6 | -2.58638 | 3.795472 | 2.99E-60 | 1.03E-58 |
| CDHR5 | -2.40997 | 7.400146 | 5.34E-60 | 1.83E-58 |
| KCNB1 | -3.71281 | -0.03553 | 6.95E-60 | 2.38E-58 |
| HS3ST6 | -4.22074 | -2.30523 | 1.20E-59 | 4.09E-58 |
| GPM6B | -2.46 | 2.213884 | 1.32E-59 | 4.50E-58 |
| CCN5 | -3.72155 | 1.043604 | 1.62E-59 | 5.50E-58 |
| TSPAN1 | -2.30071 | 8.727988 | 2.06E-59 | 6.96E-58 |
| PIANP | -2.45977 | -0.30602 | 2.82E-59 | 9.47E-58 |
| FILIP1 | -2.56561 | 1.901072 | 3.79E-59 | 1.27E-57 |
| CASQ2 | -4.22735 | 2.069814 | 4.31E-59 | 1.44E-57 |
| FMO5 | -2.08877 | 4.376209 | 4.57E-59 | 1.52E-57 |
| MAB21L1 | -3.31531 | -1.9791 | 6.58E-59 | 2.18E-57 |
| MAPK4 | -3.76144 | -0.22847 | 6.89E-59 | 2.28E-57 |
| FGFBP2 | -2.93071 | -1.498 | 1.54E-58 | 5.09E-57 |
| NR1H4 | -3.93329 | 1.291363 | 1.93E-58 | 6.34E-57 |
| PNCK | -3.3754 | 1.540973 | 3.19E-58 | 1.04E-56 |
| ELAVL3 | -3.42187 | -2.52625 | 3.26E-58 | 1.06E-56 |
| CLEC10A | -2.7818 | 2.014317 | 3.59E-58 | 1.17E-56 |
| EDIL3 | -2.08522 | 4.789994 | 6.11E-58 | 1.98E-56 |
| RGS9 | -2.20616 | 0.342092 | 7.53E-58 | 2.43E-56 |
| MRGPRF | -2.56736 | 3.657047 | 8.13E-58 | 2.62E-56 |
| RUNDC3B | -2.461 | 1.174781 | 8.37E-58 | 2.69E-56 |
| NOVA1 | -3.09288 | 0.287335 | 1.07E-57 | 3.45E-56 |
| STOX2 | -2.49293 | 0.300507 | 1.20E-57 | 3.85E-56 |
| CDH19 | -4.11056 | 0.277779 | 1.26E-57 | 4.04E-56 |
| SULT1B1 | -2.68794 | 6.311052 | 1.37E-57 | 4.36E-56 |
| REEP2 | -2.45846 | 1.051008 | 2.29E-57 | 7.25E-56 |
| FOXF2 | -2.20045 | 2.875444 | 2.32E-57 | 7.31E-56 |
| GNG3 | -2.25757 | -1.25229 | 2.73E-57 | 8.59E-56 |
| DACT3 | -2.48679 | 2.479718 | 2.96E-57 | 9.32E-56 |
| ARHGAP20 | -2.62411 | 0.378548 | 3.37E-57 | 1.06E-55 |
| B3GNT7 | -3.18298 | 5.887952 | 3.84E-57 | 1.20E-55 |
| PPP1R14A | -2.46581 | 3.036284 | 5.01E-57 | 1.56E-55 |
| RNF112 | -2.37365 | -0.27986 | 5.98E-57 | 1.86E-55 |
| P2RY4 | -3.45761 | -1.99001 | 7.20E-57 | 2.23E-55 |
| ADTRP | -3.18457 | 4.565679 | 7.28E-57 | 2.25E-55 |
| DAND5 | -2.93727 | -1.80926 | 7.94E-57 | 2.45E-55 |
| TCEAL6 | -3.8535 | -3.23175 | 1.09E-56 | 3.34E-55 |
| DISP2 | -2.28775 | 2.7429 | 1.26E-56 | 3.87E-55 |
| C11orf86 | -3.71129 | 2.479784 | 1.46E-56 | 4.45E-55 |
| BEND5 | -2.43872 | -0.38285 | 1.60E-56 | 4.88E-55 |
| AVPR1B | -3.60709 | -3.32649 | 1.74E-56 | 5.30E-55 |
| DMRTA1 | -3.39976 | -0.92483 | 1.91E-56 | 5.80E-55 |
| PHOX2A | -3.59087 | -2.41062 | 2.04E-56 | 6.19E-55 |
| KRT20 | -2.73784 | 8.670282 | 2.64E-56 | 7.98E-55 |
| CA12 | -2.30822 | 7.267536 | 2.77E-56 | 8.33E-55 |
| S100B | -2.37972 | 1.791824 | 3.64E-56 | 1.09E-54 |
| CWH43 | -3.95033 | 2.612383 | 3.97E-56 | 1.19E-54 |
| MORN5 | -4.67081 | -0.84541 | 6.85E-56 | 2.04E-54 |
| HAPLN1 | -2.38953 | 1.722926 | 7.77E-56 | 2.31E-54 |
| SLC5A12 | -3.81218 | 0.167106 | 8.98E-56 | 2.66E-54 |
| DNASE1L3 | -3.29052 | 2.33144 | 9.05E-56 | 2.68E-54 |
| CRHBP | -2.59641 | -2.105 | 9.91E-56 | 2.93E-54 |
| CCL14 | -2.23681 | -0.05516 | 1.05E-55 | 3.10E-54 |
| TNFRSF17 | -3.25765 | 1.27169 | 1.11E-55 | 3.27E-54 |
| EDN2 | -2.85438 | 0.691621 | 1.15E-55 | 3.37E-54 |
| LYNX1 | -2.60165 | 1.92899 | 1.25E-55 | 3.67E-54 |
| ZG16 | -4.52605 | 7.292271 | 2.24E-55 | 6.52E-54 |
| GLDN | -2.52267 | 1.392015 | 2.48E-55 | 7.20E-54 |
| OGN | -3.87024 | 2.658966 | 3.14E-55 | 9.03E-54 |
| FBXO32 | -2.06822 | 5.629394 | 3.40E-55 | 9.78E-54 |
| ATP1B2 | -2.38935 | 0.160681 | 5.26E-55 | 1.51E-53 |
| P2RX1 | -2.2661 | 1.38927 | 9.53E-55 | 2.72E-53 |
| SUGCT | -2.1151 | 1.731166 | 1.55E-54 | 4.38E-53 |
| AGTR1 | -3.66774 | 0.212084 | 1.72E-54 | 4.85E-53 |
| CCDC68 | -2.10161 | 3.954542 | 1.74E-54 | 4.90E-53 |
| LRMP | -2.50179 | 2.196422 | 1.85E-54 | 5.21E-53 |
| B4GALNT2 | -4.308 | 4.555071 | 2.54E-54 | 7.10E-53 |
| MYOC | -4.78445 | -0.24037 | 2.61E-54 | 7.29E-53 |
| CDH10 | -3.81625 | -3.21911 | 2.72E-54 | 7.57E-53 |
| SLC5A7 | -4.07697 | -1.86988 | 3.85E-54 | 1.06E-52 |
| SEMA6A | -2.36004 | 5.000583 | 3.90E-54 | 1.07E-52 |
| CRACD | -2.09826 | 3.877232 | 4.15E-54 | 1.14E-52 |
| ASPG | -3.04601 | 1.403319 | 4.27E-54 | 1.17E-52 |
| PEG3 | -2.62858 | 0.975225 | 4.55E-54 | 1.25E-52 |
| SPEG | -2.94469 | 3.235577 | 4.55E-54 | 1.25E-52 |
| ZNF536 | -2.9174 | -0.57962 | 5.00E-54 | 1.37E-52 |
| ANGPTL5 | -3.25324 | -3.0645 | 6.48E-54 | 1.77E-52 |
| CLDN8 | -5.2658 | 2.52906 | 7.31E-54 | 1.99E-52 |
| PRKCB | -2.38848 | 2.959727 | 1.28E-53 | 3.45E-52 |
| VIP | -3.48098 | 3.13247 | 1.61E-53 | 4.32E-52 |
| BTNL3 | -2.75159 | 4.238724 | 1.72E-53 | 4.61E-52 |
| DHRS9 | -3.34514 | 4.808519 | 3.65E-53 | 9.73E-52 |
| CPED1 | -2.30902 | 3.448337 | 4.78E-53 | 1.27E-51 |
| EPHA6 | -3.93296 | -1.78004 | 6.11E-53 | 1.62E-51 |
| DCLK1 | -2.81143 | 1.444027 | 6.13E-53 | 1.62E-51 |
| EPHA7 | -3.4983 | 1.956068 | 6.56E-53 | 1.73E-51 |
| PDE7B | -2.1418 | 0.812016 | 9.70E-53 | 2.55E-51 |
| GDPD3 | -2.19706 | 3.667984 | 1.00E-52 | 2.63E-51 |
| CMA1 | -3.82713 | -1.07725 | 1.31E-52 | 3.43E-51 |
| GPR15 | -3.5892 | 0.899965 | 1.33E-52 | 3.46E-51 |
| LAMA1 | -2.46787 | 2.188931 | 1.33E-52 | 3.47E-51 |
| FRRS1L | -3.40539 | -1.24626 | 1.42E-52 | 3.68E-51 |
| TAGLN3 | -3.57708 | -1.05945 | 1.74E-52 | 4.50E-51 |
| IGSF10 | -2.87006 | 0.840116 | 3.49E-52 | 8.99E-51 |
| AKR1B10 | -3.13399 | 4.729982 | 4.32E-52 | 1.11E-50 |
| SLIT3 | -2.49585 | 4.239039 | 4.39E-52 | 1.12E-50 |
| MAGEE2 | -3.22689 | -3.36018 | 4.47E-52 | 1.14E-50 |
| C1QTNF9 | -2.67841 | -2.21586 | 6.98E-52 | 1.78E-50 |
| SLC16A12 | -2.69549 | -2.3119 | 1.15E-51 | 2.93E-50 |
| SCN4B | -2.15762 | 1.323556 | 1.36E-51 | 3.44E-50 |
| TUBAL3 | -2.55934 | 3.222099 | 1.47E-51 | 3.71E-50 |
| BCAS1 | -2.51191 | 5.772727 | 1.52E-51 | 3.84E-50 |
| RYR3 | -2.2205 | 0.612129 | 1.67E-51 | 4.21E-50 |
| MAS1L | -3.80309 | -3.40681 | 2.27E-51 | 5.72E-50 |
| SLC7A9 | -4.00852 | 1.260505 | 2.58E-51 | 6.48E-50 |
| DPF3 | -2.32986 | 1.157071 | 2.71E-51 | 6.80E-50 |
| NACAD | -2.1456 | 0.835082 | 3.81E-51 | 9.51E-50 |
| ALDOB | -3.49756 | 6.189356 | 4.31E-51 | 1.07E-49 |
| CASR | -3.36645 | -2.75387 | 4.47E-51 | 1.11E-49 |
| MOGAT2 | -2.85401 | 4.856537 | 4.51E-51 | 1.12E-49 |
| MMP28 | -2.60798 | 3.586069 | 6.09E-51 | 1.51E-49 |
| UGT2B15 | -3.28142 | 2.748618 | 6.80E-51 | 1.68E-49 |
| GDNF | -2.22402 | 0.371281 | 7.07E-51 | 1.74E-49 |
| LIMS2 | -2.36245 | 4.742462 | 8.14E-51 | 2.01E-49 |
| JPH4 | -2.56085 | -0.90557 | 8.42E-51 | 2.07E-49 |
| PCK1 | -2.86857 | 6.195611 | 1.71E-50 | 4.18E-49 |
| UGT2B17 | -4.17119 | 6.213081 | 1.91E-50 | 4.67E-49 |
| CRYAB | -2.41674 | 3.846174 | 3.05E-50 | 7.42E-49 |
| B3GALT1 | -3.30529 | 1.510905 | 4.12E-50 | 9.99E-49 |
| CES2 | -2.07684 | 8.010425 | 4.89E-50 | 1.18E-48 |
| CPNE8 | -2.08883 | 2.818876 | 6.43E-50 | 1.55E-48 |
| VAT1L | -2.07545 | 1.481195 | 6.77E-50 | 1.63E-48 |
| ADGRL3 | -2.45272 | 1.422476 | 1.04E-49 | 2.48E-48 |
| MT2A | -2.24931 | 6.317261 | 1.11E-49 | 2.64E-48 |
| FABP2 | -3.15467 | 3.598549 | 1.12E-49 | 2.66E-48 |
| PLA2G2C | -3.15673 | -2.88337 | 1.28E-49 | 3.03E-48 |
| GAP43 | -3.17489 | 0.30103 | 3.31E-49 | 7.74E-48 |
| CNTFR | -3.80573 | 1.625587 | 5.02E-49 | 1.17E-47 |
| ADAMTS1 | -2.04134 | 4.796694 | 5.35E-49 | 1.24E-47 |
| KCNIP4 | -2.08001 | 0.989895 | 5.39E-49 | 1.25E-47 |
| CYP4B1 | -2.875 | -0.81978 | 8.27E-49 | 1.91E-47 |
| ITM2A | -2.16099 | 3.112442 | 9.90E-49 | 2.28E-47 |
| ADAMTSL1 | -2.13826 | 2.328859 | 1.01E-48 | 2.31E-47 |
| BEST2 | -3.72252 | 2.503407 | 1.02E-48 | 2.35E-47 |
| MSRB3 | -2.38464 | 4.623591 | 1.26E-48 | 2.87E-47 |
| MMRN1 | -3.08861 | 2.375403 | 1.28E-48 | 2.91E-47 |
| CA14 | -2.0557 | -0.27135 | 1.82E-48 | 4.13E-47 |
| PMP2 | -3.95294 | -1.97791 | 2.01E-48 | 4.55E-47 |
| NLGN1 | -3.55439 | -1.13571 | 2.22E-48 | 5.01E-47 |
| ABCA9 | -2.79189 | 0.536103 | 2.30E-48 | 5.19E-47 |
| BMX | -2.39275 | 1.06054 | 2.80E-48 | 6.31E-47 |
| GPX3 | -2.32485 | 5.336535 | 2.83E-48 | 6.36E-47 |
| ANKS1B | -2.91556 | -1.15789 | 2.87E-48 | 6.44E-47 |
| P2RY12 | -2.79521 | -1.13307 | 2.97E-48 | 6.66E-47 |
| SORBS1 | -2.158 | 6.08175 | 3.08E-48 | 6.90E-47 |
| TMEM59L | -2.94862 | -0.39499 | 3.28E-48 | 7.33E-47 |
| TINCR | -2.94765 | 0.744684 | 4.05E-48 | 9.00E-47 |
| PRKACB | -2.01747 | 5.840973 | 4.22E-48 | 9.38E-47 |
| MPP2 | -2.04683 | 1.054344 | 6.23E-48 | 1.38E-46 |
| CD209 | -2.3673 | 3.03616 | 6.25E-48 | 1.38E-46 |
| MASP1 | -3.27367 | 2.553188 | 7.08E-48 | 1.56E-46 |
| AKAP6 | -2.14053 | 1.885039 | 9.74E-48 | 2.14E-46 |
| SPARCL1 | -2.23043 | 6.481386 | 1.12E-47 | 2.45E-46 |
| PPP1R1A | -3.36155 | 0.556389 | 1.12E-47 | 2.45E-46 |
| MPEG1 | -2.0956 | 4.886786 | 1.13E-47 | 2.47E-46 |
| CHODL | -2.87995 | -0.50068 | 1.13E-47 | 2.48E-46 |
| TENT5C | -2.00683 | 4.988711 | 1.16E-47 | 2.54E-46 |
| ENHO | -2.53396 | -0.39367 | 1.32E-47 | 2.87E-46 |
| HMCN2 | -2.4068 | 4.267126 | 1.37E-47 | 2.97E-46 |
| APOBEC3A | -2.71467 | 0.248783 | 1.81E-47 | 3.93E-46 |
| DUSP26 | -2.74916 | -1.09803 | 2.26E-47 | 4.89E-46 |
| ANGPTL7 | -3.95883 | -0.7915 | 2.72E-47 | 5.85E-46 |
| CYBRD1 | -2.10883 | 5.698657 | 4.55E-47 | 9.75E-46 |
| GALNT16 | -2.67234 | 0.709588 | 4.61E-47 | 9.87E-46 |
| TMIGD3 | -2.07892 | 1.542768 | 5.03E-47 | 1.08E-45 |
| SETBP1 | -2.1105 | 2.67547 | 5.44E-47 | 1.16E-45 |
| NEXMIF | -3.31944 | -0.90616 | 6.52E-47 | 1.39E-45 |
| DES | -3.73139 | 8.384144 | 6.93E-47 | 1.47E-45 |
| SALL2 | -2.23014 | 1.332635 | 9.70E-47 | 2.05E-45 |
| KIF5A | -2.58815 | 0.950712 | 1.31E-46 | 2.76E-45 |
| CCDC152 | -2.39761 | 0.846633 | 1.89E-46 | 3.95E-45 |
| ANO5 | -3.10731 | 1.26201 | 1.93E-46 | 4.03E-45 |
| CTSG | -3.32439 | 0.633203 | 1.97E-46 | 4.10E-45 |
| CEND1 | -2.31257 | -1.20979 | 4.35E-46 | 9.00E-45 |
| XDH | -2.35085 | 5.066192 | 5.37E-46 | 1.11E-44 |
| SLC52A1 | -2.69279 | 0.757646 | 5.59E-46 | 1.15E-44 |
| HTR7 | -2.40976 | -1.39886 | 5.80E-46 | 1.19E-44 |
| TTLL6 | -2.27221 | 1.476581 | 7.56E-46 | 1.55E-44 |
| FLNA | -2.14284 | 9.871819 | 8.01E-46 | 1.64E-44 |
| KLB | -2.1644 | 0.016227 | 1.70E-45 | 3.43E-44 |
| LRAT | -3.33326 | -1.48619 | 2.32E-45 | 4.68E-44 |
| FEV | -3.82298 | -1.13423 | 2.43E-45 | 4.90E-44 |
| MMP27 | -3.79372 | -3.32059 | 2.90E-45 | 5.82E-44 |
| CYP2D6 | -2.33519 | 1.365954 | 3.86E-45 | 7.69E-44 |
| NECAB1 | -2.7063 | 0.194056 | 5.57E-45 | 1.11E-43 |
| HRCT1 | -2.40664 | 3.1869 | 8.58E-45 | 1.69E-43 |
| FCRL4 | -3.10185 | -1.929 | 1.19E-44 | 2.34E-43 |
| TCEAL7 | -2.14455 | 0.736362 | 1.34E-44 | 2.63E-43 |
| ITIH5 | -2.01428 | 4.599096 | 1.44E-44 | 2.83E-43 |
| SPINK2 | -3.03261 | -1.32729 | 1.67E-44 | 3.27E-43 |
| ZBTB16 | -3.32763 | 0.30718 | 3.85E-44 | 7.46E-43 |
| SIGLEC11 | -2.3034 | -1.40453 | 4.19E-44 | 8.11E-43 |
| SLC16A9 | -2.34691 | 3.856413 | 5.95E-44 | 1.15E-42 |
| SEC14L5 | -2.45967 | -0.42277 | 6.75E-44 | 1.30E-42 |
| F13A1 | -2.65957 | 4.353574 | 7.94E-44 | 1.53E-42 |
| STAB2 | -2.15175 | 0.065143 | 8.32E-44 | 1.60E-42 |
| LONRF2 | -3.50732 | 0.47607 | 1.03E-43 | 1.97E-42 |
| MFSD4A | -2.16666 | 4.003364 | 2.94E-43 | 5.53E-42 |
| LGALS9C | -2.87679 | 2.295917 | 3.25E-43 | 6.10E-42 |
| EYA2 | -2.88754 | 2.456587 | 4.72E-43 | 8.80E-42 |
| RBM20 | -2.59237 | -0.40286 | 5.34E-43 | 9.93E-42 |
| THRB | -2.38678 | 3.23661 | 8.39E-43 | 1.56E-41 |
| FOLR2 | -2.37338 | 2.979553 | 8.79E-43 | 1.62E-41 |
| GLRA4 | -2.7837 | -0.94258 | 1.28E-42 | 2.34E-41 |
| CES3 | -2.20432 | 5.714828 | 1.45E-42 | 2.65E-41 |
| CHL1 | -2.50795 | 2.107165 | 1.48E-42 | 2.69E-41 |
| ATCAY | -3.1676 | -1.61025 | 1.56E-42 | 2.83E-41 |
| GALR1 | -3.56765 | -2.64814 | 1.85E-42 | 3.36E-41 |
| RGMA | -2.42914 | 3.308899 | 2.27E-42 | 4.12E-41 |
| CYS1 | -2.14431 | 1.491921 | 2.32E-42 | 4.20E-41 |
| CD160 | -2.22403 | -0.45348 | 3.06E-42 | 5.51E-41 |
| BAALC | -2.31277 | -0.40256 | 3.27E-42 | 5.87E-41 |
| TMEM255A | -2.30723 | 0.218085 | 3.27E-42 | 5.87E-41 |
| PGR | -2.54075 | 0.513049 | 4.89E-42 | 8.71E-41 |
| CLU | -2.36755 | 6.353364 | 5.18E-42 | 9.21E-41 |
| ATP6V0D2 | -2.19764 | 0.656072 | 5.83E-42 | 1.03E-40 |
| GFRA3 | -2.83512 | 0.414341 | 1.25E-41 | 2.19E-40 |
| MADCAM1 | -2.39545 | 0.883187 | 1.33E-41 | 2.33E-40 |
| MAP6 | -2.16016 | 0.8661 | 1.56E-41 | 2.74E-40 |
| C1QTNF7 | -2.6398 | 0.90514 | 1.59E-41 | 2.77E-40 |
| TMEM130 | -2.51922 | -0.18036 | 1.79E-41 | 3.11E-40 |
| SLC35F1 | -2.11447 | -0.34675 | 2.58E-41 | 4.47E-40 |
| FAM163B | -2.94685 | -1.31236 | 3.35E-41 | 5.77E-40 |
| RAB9B | -2.15588 | 0.469542 | 3.60E-41 | 6.21E-40 |
| IQCN | -2.06902 | 1.459578 | 3.95E-41 | 6.79E-40 |
| NPAS4 | -2.70008 | -2.48013 | 4.02E-41 | 6.91E-40 |
| FGFR2 | -2.25572 | 4.357085 | 4.26E-41 | 7.32E-40 |
| LRRC3B | -3.14749 | -3.34945 | 4.81E-41 | 8.25E-40 |
| FOXD3 | -3.28712 | -1.99766 | 5.15E-41 | 8.83E-40 |
| NUGGC | -2.50183 | 0.24348 | 5.47E-41 | 9.37E-40 |
| ASB5 | -4.2909 | -0.966 | 1.35E-40 | 2.28E-39 |
| ASTN1 | -3.46428 | -1.53369 | 2.27E-40 | 3.81E-39 |
| ATRNL1 | -2.95709 | -0.1099 | 2.66E-40 | 4.46E-39 |
| MFAP4 | -2.23152 | 5.717025 | 2.76E-40 | 4.62E-39 |
| IRF4 | -2.41933 | 2.75732 | 2.78E-40 | 4.64E-39 |
| STUM | -3.18241 | 1.990897 | 3.50E-40 | 5.83E-39 |
| SMIM28 | -3.63203 | -2.97323 | 3.57E-40 | 5.93E-39 |
| PDLIM3 | -2.14292 | 4.831718 | 4.37E-40 | 7.25E-39 |
| TCEAL5 | -3.33528 | -2.58868 | 7.10E-40 | 1.17E-38 |
| SCUBE1 | -2.06908 | 1.995879 | 8.36E-40 | 1.37E-38 |
| SEMA3G | -2.00692 | 2.82206 | 1.14E-39 | 1.85E-38 |
| RIC3 | -2.83585 | -0.81113 | 1.81E-39 | 2.92E-38 |
| SLC7A14 | -3.66693 | -1.29626 | 2.41E-39 | 3.87E-38 |
| CASQ1 | -2.4468 | -1.74075 | 2.94E-39 | 4.70E-38 |
| CCL28 | -2.15598 | 5.115224 | 3.29E-39 | 5.24E-38 |
| ADRA1A | -2.77447 | -2.53339 | 4.89E-39 | 7.74E-38 |
| ATP13A4 | -2.57464 | 0.468004 | 5.73E-39 | 9.03E-38 |
| UGT2A3 | -3.22888 | 4.416904 | 6.24E-39 | 9.80E-38 |
| GRIA4 | -2.59801 | -1.14972 | 7.17E-39 | 1.12E-37 |
| TCEAL2 | -3.72037 | -0.0374 | 7.70E-39 | 1.20E-37 |
| VIT | -2.65339 | -0.72707 | 7.73E-39 | 1.21E-37 |
| CD36 | -2.43537 | 3.610203 | 9.16E-39 | 1.43E-37 |
| RASGEF1C | -3.00541 | -1.96723 | 9.56E-39 | 1.49E-37 |
| NOS1 | -3.31537 | 0.13944 | 1.17E-38 | 1.81E-37 |
| CCL15-CCL14 | -2.11028 | -0.27617 | 1.28E-38 | 1.98E-37 |
| SEMA3E | -3.56114 | -0.11319 | 1.33E-38 | 2.06E-37 |
| IGSF11 | -3.02095 | -1.77561 | 1.37E-38 | 2.12E-37 |
| NPY | -3.22856 | -2.03167 | 1.55E-38 | 2.40E-37 |
| C14orf132 | -2.0585 | 2.733878 | 1.74E-38 | 2.67E-37 |
| PDZK1 | -2.41163 | 0.686683 | 2.11E-38 | 3.24E-37 |
| FCER1A | -2.47936 | -0.10997 | 2.44E-38 | 3.73E-37 |
| FCRLA | -2.8136 | 0.43179 | 3.23E-38 | 4.91E-37 |
| CASP5 | -2.13445 | 2.967952 | 3.34E-38 | 5.07E-37 |
| CLCNKB | -2.79719 | -2.27996 | 3.45E-38 | 5.23E-37 |
| KCNK3 | -2.92033 | 0.456317 | 5.10E-38 | 7.68E-37 |
| CLEC4G | -2.98836 | -1.30057 | 6.08E-38 | 9.11E-37 |
| SIGLEC8 | -2.33152 | 0.019938 | 7.69E-38 | 1.15E-36 |
| SLC9A2 | -2.1724 | 4.780214 | 8.02E-38 | 1.19E-36 |
| FAM163A | -2.13216 | -0.90772 | 8.62E-38 | 1.28E-36 |
| PPP1R3C | -2.25405 | 1.565796 | 8.78E-38 | 1.30E-36 |
| B3GALT5 | -2.67139 | 5.437294 | 9.27E-38 | 1.37E-36 |
| FABP1 | -2.79505 | 9.182657 | 1.09E-37 | 1.60E-36 |
| DMD | -2.1303 | 4.002745 | 1.34E-37 | 1.96E-36 |
| GCSAML | -2.42936 | -1.43527 | 1.36E-37 | 2.00E-36 |
| HTR3E | -3.36996 | -1.45935 | 1.38E-37 | 2.02E-36 |
| ISX | -2.37715 | 4.811563 | 2.20E-37 | 3.20E-36 |
| TRIM9 | -2.38325 | 0.407742 | 2.28E-37 | 3.31E-36 |
| RERG | -2.20895 | 2.066305 | 2.71E-37 | 3.92E-36 |
| SLITRK3 | -3.61594 | -1.15994 | 2.73E-37 | 3.96E-36 |
| CHRDL1 | -3.0661 | 3.671768 | 2.76E-37 | 3.99E-36 |
| CPXM2 | -2.39203 | 3.667754 | 2.77E-37 | 4.00E-36 |
| MEP1A | -2.41143 | 7.204206 | 3.13E-37 | 4.51E-36 |
| SLC8A2 | -2.85949 | 0.33156 | 5.20E-37 | 7.43E-36 |
| RELN | -2.66681 | 1.05141 | 5.47E-37 | 7.81E-36 |
| BMP5 | -2.60703 | 1.469797 | 6.04E-37 | 8.61E-36 |
| ACSM5 | -2.6136 | -1.47822 | 6.34E-37 | 9.00E-36 |
| PCOLCE2 | -3.06843 | 1.194782 | 8.07E-37 | 1.14E-35 |
| CCDC158 | -2.01854 | -1.51716 | 1.23E-36 | 1.72E-35 |
| CORO2B | -2.202 | 0.694346 | 1.27E-36 | 1.78E-35 |
| BEND4 | -2.56636 | -1.62494 | 1.59E-36 | 2.22E-35 |
| GNG8 | -2.45137 | -2.61613 | 2.00E-36 | 2.78E-35 |
| CNTNAP3 | -2.59829 | -0.97441 | 2.10E-36 | 2.92E-35 |
| TREH | -2.40522 | 1.384646 | 2.45E-36 | 3.40E-35 |
| ADCY2 | -2.10922 | 0.688722 | 2.69E-36 | 3.73E-35 |
| PLA2G5 | -2.34986 | 0.322093 | 3.01E-36 | 4.15E-35 |
| RND2 | -2.1819 | -0.35107 | 3.44E-36 | 4.72E-35 |
| SFRP5 | -3.61027 | -0.07313 | 4.75E-36 | 6.47E-35 |
| ZNF835 | -2.08406 | -1.19359 | 5.08E-36 | 6.90E-35 |
| CD79B | -2.16155 | 1.910484 | 5.14E-36 | 6.97E-35 |
| UNC5D | -2.81796 | -1.93845 | 5.52E-36 | 7.48E-35 |
| HBB | -2.59619 | 4.553813 | 7.30E-36 | 9.82E-35 |
| PROKR1 | -2.76362 | -3.05875 | 7.37E-36 | 9.91E-35 |
| SCG2 | -2.34363 | 2.15263 | 1.26E-35 | 1.68E-34 |
| LGALS2 | -2.5277 | 3.397207 | 1.35E-35 | 1.80E-34 |
| LRCH2 | -2.13693 | 0.766489 | 1.39E-35 | 1.84E-34 |
| GCNT4 | -2.16457 | 1.140701 | 1.75E-35 | 2.32E-34 |
| KRT9 | -3.01918 | -2.8627 | 2.00E-35 | 2.64E-34 |
| PCDH10 | -2.9639 | -1.69423 | 2.20E-35 | 2.89E-34 |
| FMN2 | -3.25162 | 0.077509 | 2.86E-35 | 3.73E-34 |
| KY | -2.42586 | -1.81799 | 3.65E-35 | 4.74E-34 |
| AARD | -2.35941 | -1.84395 | 8.20E-35 | 1.05E-33 |
| RHEX | -2.27399 | -0.22835 | 1.24E-34 | 1.57E-33 |
| SI | -3.27573 | 4.579164 | 1.37E-34 | 1.73E-33 |
| HTR4 | -2.86161 | 0.286283 | 1.50E-34 | 1.88E-33 |
| STAP1 | -2.37097 | -0.62715 | 1.51E-34 | 1.90E-33 |
| HSPB7 | -2.67257 | 3.070438 | 1.68E-34 | 2.11E-33 |
| SCN3A | -2.1528 | -0.25779 | 1.70E-34 | 2.13E-33 |
| KCNA1 | -3.52768 | -2.24197 | 1.76E-34 | 2.20E-33 |
| LY9 | -2.12433 | 0.931633 | 1.77E-34 | 2.22E-33 |
| COL21A1 | -2.0915 | 0.448851 | 2.52E-34 | 3.13E-33 |
| POU3F4 | -3.98058 | -3.34758 | 2.99E-34 | 3.70E-33 |
| SPHKAP | -3.45335 | -3.34231 | 3.06E-34 | 3.78E-33 |
| EPHA5 | -2.80559 | -2.55118 | 3.43E-34 | 4.23E-33 |
| NXPE4 | -2.9338 | 5.357842 | 4.38E-34 | 5.39E-33 |
| SLC4A10 | -3.04201 | 0.213202 | 5.11E-34 | 6.26E-33 |
| GPR119 | -3.45998 | -3.56946 | 6.05E-34 | 7.36E-33 |
| ADAM33 | -2.18089 | 2.042567 | 6.61E-34 | 8.03E-33 |
| SYT10 | -3.33109 | -2.68501 | 7.18E-34 | 8.71E-33 |
| GHR | -2.2131 | 1.960643 | 8.76E-34 | 1.06E-32 |
| NRSN1 | -3.13572 | -2.50352 | 1.58E-33 | 1.89E-32 |
| NEU4 | -2.25688 | 3.773062 | 1.71E-33 | 2.04E-32 |
| MT1E | -2.37651 | 5.69958 | 1.82E-33 | 2.17E-32 |
| CDHR2 | -2.23762 | 5.991971 | 2.01E-33 | 2.40E-32 |
| VSIG2 | -2.82826 | 5.284502 | 2.10E-33 | 2.50E-32 |
| MAOB | -2.43534 | 3.46966 | 2.68E-33 | 3.18E-32 |
| RASD2 | -2.03792 | 3.502869 | 3.15E-33 | 3.72E-32 |
| HAND1 | -4.02165 | 1.115518 | 4.25E-33 | 5.00E-32 |
| LGI4 | -2.0868 | 2.450991 | 5.42E-33 | 6.34E-32 |
| ZNF229 | -2.03899 | -0.73919 | 5.58E-33 | 6.52E-32 |
| CD79A | -2.47835 | 3.588519 | 1.12E-32 | 1.29E-31 |
| GPER1 | -2.10578 | 1.84326 | 1.25E-32 | 1.44E-31 |
| CYP2C18 | -2.24073 | 1.870128 | 1.71E-32 | 1.96E-31 |
| PTPRZ1 | -3.05829 | -0.2922 | 2.01E-32 | 2.30E-31 |
| CD22 | -2.46783 | 1.986874 | 2.10E-32 | 2.40E-31 |
| FAM189A2 | -2.194 | 0.477339 | 2.48E-32 | 2.82E-31 |
| SNAP91 | -3.01125 | -1.46281 | 2.52E-32 | 2.87E-31 |
| NAP1L3 | -2.03445 | 0.419563 | 2.67E-32 | 3.03E-31 |
| EFHC2 | -2.35018 | -0.60617 | 2.91E-32 | 3.30E-31 |
| ABCB5 | -3.28494 | -1.72386 | 3.14E-32 | 3.54E-31 |
| TRHDE | -2.32179 | 1.33335 | 3.30E-32 | 3.72E-31 |
| PRELP | -2.58944 | 4.518245 | 3.47E-32 | 3.91E-31 |
| NEXN | -2.09803 | 3.562035 | 5.24E-32 | 5.89E-31 |
| AADACL2 | -3.78908 | -3.02689 | 5.72E-32 | 6.41E-31 |
| NWD2 | -2.53651 | -0.90791 | 6.08E-32 | 6.79E-31 |
| CNTN3 | -2.63489 | 1.014753 | 7.25E-32 | 8.09E-31 |
| PPP2R2B | -2.03584 | -0.0912 | 8.26E-32 | 9.19E-31 |
| CCR2 | -2.12516 | 1.414498 | 1.02E-31 | 1.13E-30 |
| RIMS4 | -3.33303 | -0.93585 | 1.19E-31 | 1.31E-30 |
| MYPN | -2.48387 | -0.80942 | 1.34E-31 | 1.48E-30 |
| SIGLEC6 | -2.27407 | -1.25073 | 1.44E-31 | 1.58E-30 |
| ROPN1 | -3.12861 | -2.94359 | 1.56E-31 | 1.71E-30 |
| ZBTB7C | -2.14936 | 4.753124 | 3.09E-31 | 3.33E-30 |
| NKAPL | -2.0448 | -2.22341 | 3.89E-31 | 4.17E-30 |
| TLR10 | -2.25299 | 0.137617 | 4.83E-31 | 5.17E-30 |
| CILP | -2.83637 | 2.98697 | 6.07E-31 | 6.46E-30 |
| HIF3A | -2.25382 | 1.647957 | 8.25E-31 | 8.72E-30 |
| SLC27A6 | -2.79778 | -1.92106 | 8.33E-31 | 8.79E-30 |
| SGCA | -2.40511 | 1.106815 | 8.85E-31 | 9.33E-30 |
| NLGN4X | -2.01414 | 0.71246 | 8.94E-31 | 9.42E-30 |
| MFAP5 | -2.46328 | 3.354987 | 1.00E-30 | 1.05E-29 |
| TLL1 | -2.33588 | 0.021486 | 1.01E-30 | 1.06E-29 |
| CFD | -2.32174 | 5.096268 | 1.28E-30 | 1.33E-29 |
| AC008878.3 | -2.9175 | -3.33801 | 1.44E-30 | 1.50E-29 |
| CELF4 | -2.50631 | -1.55807 | 1.51E-30 | 1.57E-29 |
| PRDM6 | -2.03829 | 1.030195 | 1.56E-30 | 1.62E-29 |
| VPREB3 | -2.35143 | -0.13055 | 1.69E-30 | 1.75E-29 |
| BHMT2 | -2.13867 | 0.07871 | 1.75E-30 | 1.81E-29 |
| HOXD1 | -2.29706 | -0.39906 | 2.02E-30 | 2.09E-29 |
| CLDN11 | -2.13971 | 0.97009 | 2.04E-30 | 2.10E-29 |
| ABCA6 | -2.29236 | 0.849406 | 2.16E-30 | 2.23E-29 |
| ADAMTS8 | -2.08456 | 1.20997 | 2.18E-30 | 2.25E-29 |
| ENTPD8 | -2.32033 | 4.776 | 2.96E-30 | 3.04E-29 |
| PTCHD1 | -2.92759 | 0.194317 | 5.25E-30 | 5.34E-29 |
| FCER2 | -2.94686 | -0.08517 | 5.80E-30 | 5.88E-29 |
| GPR88 | -2.4012 | -2.26541 | 5.86E-30 | 5.93E-29 |
| TMEM74 | -2.16885 | -2.24405 | 7.06E-30 | 7.11E-29 |
| AGXT2 | -3.2418 | -3.16209 | 9.32E-30 | 9.34E-29 |
| DPEP3 | -2.48066 | -2.58226 | 1.25E-29 | 1.25E-28 |
| CNTN1 | -2.71425 | 0.935636 | 1.28E-29 | 1.28E-28 |
| TRPC7 | -2.26715 | -2.37913 | 1.29E-29 | 1.29E-28 |
| CCL8 | -2.41816 | 1.276196 | 1.32E-29 | 1.32E-28 |
| PTH1R | -2.02699 | -0.50601 | 1.97E-29 | 1.95E-28 |
| SELENBP1 | -2.00449 | 8.675346 | 2.01E-29 | 1.99E-28 |
| SCGB2A1 | -2.65293 | -0.13087 | 2.25E-29 | 2.22E-28 |
| LCN6 | -2.72142 | -2.46296 | 2.37E-29 | 2.33E-28 |
| CALY | -2.65446 | -1.493 | 3.20E-29 | 3.14E-28 |
| KIAA0408 | -2.91989 | -3.08089 | 5.42E-29 | 5.27E-28 |
| LCN10 | -2.6669 | -2.84657 | 6.64E-29 | 6.42E-28 |
| GALNT15 | -2.07544 | 1.75334 | 6.84E-29 | 6.61E-28 |
| AADAC | -2.94539 | 0.365811 | 7.70E-29 | 7.42E-28 |
| KCNA3 | -2.18738 | -0.22296 | 8.27E-29 | 7.96E-28 |
| ENPP7 | -3.07236 | -2.22701 | 8.67E-29 | 8.35E-28 |
| SPOCK3 | -3.3392 | -1.93729 | 1.13E-28 | 1.08E-27 |
| ARPP21 | -3.11168 | -3.035 | 1.41E-28 | 1.34E-27 |
| TMEM155 | -2.05304 | -2.34992 | 1.62E-28 | 1.53E-27 |
| C10orf105 | -2.27539 | -3.22882 | 2.01E-28 | 1.88E-27 |
| SYT4 | -3.19302 | -1.1734 | 2.39E-28 | 2.24E-27 |
| LUZP2 | -2.29028 | -0.13132 | 2.44E-28 | 2.28E-27 |
| CHRM2 | -3.58323 | 1.298549 | 2.64E-28 | 2.47E-27 |
| CLEC4M | -3.08193 | -3.35262 | 2.76E-28 | 2.57E-27 |
| GCNT3 | -2.12353 | 7.055727 | 2.79E-28 | 2.60E-27 |
| PHF24 | -2.28351 | -1.98075 | 5.76E-28 | 5.28E-27 |
| NBEA | -2.21102 | 1.864729 | 6.19E-28 | 5.68E-27 |
| COL4A6 | -2.18198 | 0.54542 | 7.54E-28 | 6.87E-27 |
| NCR2 | -2.61853 | -3.46313 | 1.19E-27 | 1.07E-26 |
| CCL13 | -2.51646 | 1.718284 | 1.26E-27 | 1.14E-26 |
| MAPT | -2.2478 | 1.321482 | 1.41E-27 | 1.27E-26 |
| SLC17A1 | -2.41 | -2.42168 | 1.46E-27 | 1.31E-26 |
| CXCR5 | -2.65359 | -2.1281 | 1.46E-27 | 1.31E-26 |
| PCDH11Y | -3.44663 | -3.57295 | 1.98E-27 | 1.77E-26 |
| SERPINA9 | -3.66072 | -1.66353 | 2.15E-27 | 1.92E-26 |
| SSTR3 | -2.22523 | -1.8734 | 2.74E-27 | 2.43E-26 |
| CNR2 | -2.26714 | -1.13753 | 3.20E-27 | 2.83E-26 |
| SLC15A1 | -2.75435 | 2.777509 | 3.76E-27 | 3.31E-26 |
| RPH3A | -2.37246 | -2.32431 | 3.90E-27 | 3.43E-26 |
| MGAT4C | -3.06941 | -2.33178 | 4.16E-27 | 3.66E-26 |
| HHIP | -2.02411 | 2.203262 | 4.17E-27 | 3.66E-26 |
| VXN | -2.32232 | -2.19184 | 5.25E-27 | 4.58E-26 |
| FGF10 | -2.1379 | -0.18457 | 5.63E-27 | 4.91E-26 |
| ALKAL2 | -2.03946 | -0.30663 | 5.90E-27 | 5.13E-26 |
| MB | -2.23054 | 2.043397 | 5.93E-27 | 5.16E-26 |
| BLK | -2.44302 | 0.37315 | 8.19E-27 | 7.08E-26 |
| PENK | -3.16318 | -0.23338 | 8.28E-27 | 7.16E-26 |
| SH2D6 | -2.32688 | 0.359877 | 8.90E-27 | 7.68E-26 |
| CNKSR2 | -2.12884 | -1.04984 | 9.12E-27 | 7.86E-26 |
| OSR1 | -2.25346 | 0.815641 | 1.39E-26 | 1.19E-25 |
| WDR17 | -2.50671 | -1.04941 | 1.56E-26 | 1.33E-25 |
| ST8SIA3 | -3.14842 | -2.42234 | 1.60E-26 | 1.36E-25 |
| FCRL2 | -2.3216 | 0.15538 | 2.35E-26 | 1.98E-25 |
| WSCD2 | -2.63968 | -0.61458 | 2.40E-26 | 2.02E-25 |
| ACKR1 | -2.24655 | 3.099605 | 3.06E-26 | 2.56E-25 |
| LRFN5 | -2.06079 | -1.0227 | 3.21E-26 | 2.69E-25 |
| SNAP25 | -2.59743 | -0.03746 | 3.63E-26 | 3.03E-25 |
| COL19A1 | -2.67984 | -2.27134 | 3.82E-26 | 3.18E-25 |
| DNER | -2.51418 | -0.05023 | 4.02E-26 | 3.35E-25 |
| COL6A5 | -2.71066 | -1.46843 | 4.77E-26 | 3.96E-25 |
| CUX2 | -2.38228 | -2.47239 | 5.93E-26 | 4.89E-25 |
| MS4A2 | -2.08878 | 0.663676 | 6.73E-26 | 5.53E-25 |
| CHST5 | -2.45345 | 2.570175 | 1.11E-25 | 8.99E-25 |
| ENAM | -2.59007 | -1.35142 | 1.16E-25 | 9.42E-25 |
| PLD5 | -2.9125 | -3.03611 | 1.18E-25 | 9.59E-25 |
| UGT1A1 | -2.90453 | -0.21198 | 1.34E-25 | 1.08E-24 |
| BEX1 | -2.635 | -0.83564 | 1.47E-25 | 1.18E-24 |
| RBM24 | -2.00424 | 0.619708 | 1.48E-25 | 1.20E-24 |
| SYNDIG1L | -2.28886 | -2.61081 | 1.82E-25 | 1.46E-24 |
| BRINP3 | -3.42593 | 1.085207 | 2.39E-25 | 1.92E-24 |
| SLITRK2 | -2.58806 | -2.28828 | 2.51E-25 | 2.01E-24 |
| PAPPA2 | -2.42732 | -0.41276 | 2.83E-25 | 2.26E-24 |
| GSG1L | -2.65999 | -2.73888 | 2.89E-25 | 2.30E-24 |
| INA | -2.63686 | -0.29683 | 2.98E-25 | 2.37E-24 |
| KRT222 | -2.61375 | -2.80651 | 3.17E-25 | 2.52E-24 |
| OLFM3 | -3.00447 | -2.12029 | 3.63E-25 | 2.87E-24 |
| SHISA3 | -2.25941 | 0.930961 | 4.07E-25 | 3.21E-24 |
| AC136428.1 | -2.60204 | -1.09393 | 4.87E-25 | 3.80E-24 |
| ENTPD3 | -2.15211 | 0.52111 | 5.94E-25 | 4.63E-24 |
| TMEM179 | -2.67323 | -1.10448 | 6.32E-25 | 4.91E-24 |
| FOXP2 | -2.2013 | 2.660779 | 6.82E-25 | 5.28E-24 |
| ASB11 | -2.6654 | -3.49411 | 9.38E-25 | 7.22E-24 |
| CR2 | -2.71902 | 2.551576 | 9.81E-25 | 7.54E-24 |
| SCRG1 | -2.81603 | -0.21067 | 1.29E-24 | 9.88E-24 |
| PNOC | -2.10883 | -0.51228 | 1.88E-24 | 1.43E-23 |
| ZMAT4 | -2.7601 | -2.78278 | 2.44E-24 | 1.84E-23 |
| MS4A8 | -2.12238 | 3.350672 | 2.73E-24 | 2.06E-23 |
| CHRNA3 | -2.05353 | 1.450749 | 2.96E-24 | 2.22E-23 |
| DSCAML1 | -2.31526 | -0.11154 | 2.97E-24 | 2.22E-23 |
| NXPE1 | -2.2719 | 4.443139 | 4.14E-24 | 3.07E-23 |
| CLVS2 | -2.86557 | -2.35226 | 4.96E-24 | 3.67E-23 |
| UCHL1 | -2.01595 | 2.579301 | 6.14E-24 | 4.52E-23 |
| GPIHBP1 | -2.06851 | -0.50045 | 1.37E-23 | 9.91E-23 |
| CIDEC | -2.23641 | 1.948545 | 1.80E-23 | 1.30E-22 |
| HMGCS2 | -2.31723 | 8.044316 | 2.57E-23 | 1.83E-22 |
| SYT9 | -2.53505 | -3.05811 | 2.78E-23 | 1.98E-22 |
| MS4A1 | -2.59368 | 1.974412 | 3.46E-23 | 2.46E-22 |
| RPL10L | -2.09178 | -1.27212 | 5.54E-23 | 3.89E-22 |
| GRIN2A | -2.49388 | -1.50492 | 5.80E-23 | 4.07E-22 |
| CNGA3 | -2.83138 | -0.13878 | 5.84E-23 | 4.09E-22 |
| MZB1 | -2.04003 | 4.129914 | 7.21E-23 | 5.02E-22 |
| VWC2 | -2.21303 | -1.72849 | 7.37E-23 | 5.13E-22 |
| CLEC17A | -2.31286 | -1.35823 | 7.73E-23 | 5.37E-22 |
| AC009163.3 | -2.44279 | -2.04654 | 9.16E-23 | 6.34E-22 |
| SNTG2 | -2.30178 | -2.59792 | 1.42E-22 | 9.72E-22 |
| TCL1A | -2.98976 | 0.343844 | 1.74E-22 | 1.18E-21 |
| PLIN4 | -2.82603 | 3.938805 | 1.89E-22 | 1.28E-21 |
| ASXL3 | -2.06957 | 0.586724 | 2.46E-22 | 1.65E-21 |
| CCL21 | -2.10062 | 4.25783 | 2.69E-22 | 1.80E-21 |
| SLC30A8 | -2.51311 | -2.91171 | 4.22E-22 | 2.79E-21 |
| HEPACAM2 | -2.49112 | 4.578285 | 6.10E-22 | 4.01E-21 |
| HBA2 | -2.02007 | 2.334414 | 6.20E-22 | 4.07E-21 |
| CHRM4 | -2.08558 | -1.36544 | 1.60E-21 | 1.03E-20 |
| GCG | -3.66029 | 3.180049 | 2.01E-21 | 1.29E-20 |
| EVX2 | -2.66308 | -2.31336 | 2.20E-21 | 1.40E-20 |
| RALYL | -2.94674 | -3.10757 | 2.32E-21 | 1.48E-20 |
| GABRG2 | -3.05305 | -2.29496 | 2.63E-21 | 1.67E-20 |
| LY6H | -2.00563 | -0.62366 | 2.91E-21 | 1.84E-20 |
| AICDA | -2.87569 | -2.35972 | 2.95E-21 | 1.87E-20 |
| NEFL | -2.75796 | 0.654245 | 3.32E-21 | 2.09E-20 |
| NLRP7 | -2.07121 | -1.73388 | 3.68E-21 | 2.31E-20 |
| ABCC8 | -2.11864 | -1.04291 | 4.67E-21 | 2.91E-20 |
| DCAF12L2 | -2.47324 | -3.16866 | 5.82E-21 | 3.61E-20 |
| SPX | -2.07865 | -2.88308 | 6.34E-21 | 3.93E-20 |
| KIF1A | -2.59779 | 1.272242 | 6.46E-21 | 4.00E-20 |
| CCL19 | -2.27883 | 2.196113 | 6.89E-21 | 4.25E-20 |
| MYT1L | -2.77106 | -2.84126 | 7.51E-21 | 4.62E-20 |
| KCNG3 | -2.14511 | -1.36311 | 7.73E-21 | 4.76E-20 |
| ZDHHC22 | -2.91144 | -2.73749 | 9.77E-21 | 5.98E-20 |
| HTR3A | -2.42326 | -1.05326 | 9.83E-21 | 6.02E-20 |
| KRT1 | -2.3865 | -1.39724 | 1.19E-20 | 7.23E-20 |
| DEFB1 | -2.04675 | 1.938253 | 1.60E-20 | 9.72E-20 |
| NTNG1 | -2.64069 | -1.07183 | 4.87E-20 | 2.88E-19 |
| ADRB3 | -2.46555 | -2.47733 | 5.90E-20 | 3.47E-19 |
| RSPO1 | -2.06112 | -1.4077 | 6.52E-20 | 3.83E-19 |
| RAB3C | -2.06767 | -0.03691 | 6.69E-20 | 3.92E-19 |
| FCGBP | -2.36371 | 10.02651 | 2.00E-19 | 1.13E-18 |
| GFI1B | -2.15448 | -0.89535 | 2.15E-19 | 1.22E-18 |
| REP15 | -2.2066 | 0.978232 | 2.53E-19 | 1.43E-18 |
| ZNF676 | -2.25611 | -2.83412 | 3.44E-19 | 1.93E-18 |
| ABCG5 | -2.07387 | 0.155067 | 3.98E-19 | 2.23E-18 |
| FER1L6 | -2.37005 | 2.869762 | 4.32E-19 | 2.41E-18 |
| SORCS3 | -2.65228 | -2.78988 | 5.82E-19 | 3.22E-18 |
| PLCXD3 | -2.63405 | -0.90094 | 7.96E-19 | 4.36E-18 |
| KIR2DL4 | -2.03455 | -0.88606 | 8.34E-19 | 4.56E-18 |
| RGS13 | -2.18354 | -0.1947 | 1.15E-18 | 6.22E-18 |
| LGALS9B | -2.03945 | 1.881752 | 1.75E-18 | 9.37E-18 |
| DIRAS2 | -2.19768 | -1.96153 | 1.94E-18 | 1.04E-17 |
| ADH1A | -2.35605 | -3.00462 | 2.43E-18 | 1.30E-17 |
| GRIA1 | -2.09596 | -2.14707 | 2.72E-18 | 1.45E-17 |
| SLC18A3 | -2.8164 | -2.51234 | 2.87E-18 | 1.52E-17 |
| KCNJ13 | -3.52332 | 0.061935 | 3.29E-18 | 1.74E-17 |
| FDCSP | -2.61104 | 1.227968 | 4.31E-18 | 2.26E-17 |
| CA10 | -2.28597 | -2.18015 | 4.34E-18 | 2.28E-17 |
| NPY1R | -2.0911 | 1.095228 | 6.10E-18 | 3.17E-17 |
| TBPL2 | -2.06346 | -3.39355 | 8.16E-18 | 4.20E-17 |
| TACR1 | -2.09656 | -0.57621 | 9.78E-18 | 5.00E-17 |
| TMEM196 | -2.57871 | -3.5997 | 1.39E-17 | 7.02E-17 |
| UGT1A9 | -2.15896 | -3.17334 | 1.69E-17 | 8.53E-17 |
| TNNT3 | -2.22091 | -2.30678 | 1.98E-17 | 9.90E-17 |
| CP | -2.10095 | 2.265092 | 2.22E-17 | 1.11E-16 |
| ZNF728 | -2.31444 | -3.43351 | 2.28E-17 | 1.14E-16 |
| C14orf180 | -3.02459 | -2.21777 | 2.48E-17 | 1.24E-16 |
| MRGPRE | -2.26453 | -3.38614 | 3.26E-17 | 1.61E-16 |
| FSIP2 | -2.03562 | 2.682941 | 6.07E-17 | 2.96E-16 |
| TPSG1 | -2.03299 | 3.025437 | 7.46E-17 | 3.62E-16 |
| CD300LG | -2.69863 | -1.41178 | 9.26E-17 | 4.45E-16 |
| SLC14A2 | -2.53991 | -0.36707 | 9.89E-17 | 4.75E-16 |
| ABCG8 | -2.22648 | -0.33435 | 1.47E-16 | 6.96E-16 |
| SULT2A1 | -2.81309 | 0.432188 | 2.09E-16 | 9.80E-16 |
| NRAP | -2.1702 | -0.91185 | 2.94E-16 | 1.37E-15 |
| ITPRID1 | -2.09426 | -1.21392 | 3.10E-16 | 1.44E-15 |
| HRK | -2.33069 | -1.62436 | 4.25E-16 | 1.96E-15 |
| FAM181B | -2.09203 | -1.54413 | 5.11E-16 | 2.34E-15 |
| NTRK3 | -2.00167 | 0.041207 | 5.30E-16 | 2.43E-15 |
| RPRM | -2.06965 | -0.61403 | 6.83E-16 | 3.10E-15 |
| IGLL5 | -2.00173 | 5.781484 | 6.84E-16 | 3.10E-15 |
| GNG13 | -2.19367 | -0.97079 | 7.14E-16 | 3.24E-15 |
| XPNPEP2 | -2.08057 | 4.434718 | 9.88E-16 | 4.44E-15 |
| CELA3B | -2.44049 | -2.23375 | 1.00E-15 | 4.50E-15 |
| IQCM | -2.13986 | -2.06718 | 1.13E-15 | 5.04E-15 |
| AMER2 | -2.48855 | -3.28449 | 2.50E-15 | 1.10E-14 |
| TAFA4 | -2.50313 | -3.12735 | 2.96E-15 | 1.29E-14 |
| KCNS2 | -2.00896 | -2.32483 | 3.58E-15 | 1.56E-14 |
| SLC13A2 | -2.16644 | 3.633703 | 4.13E-15 | 1.79E-14 |
| OMD | -2.24176 | 0.483444 | 4.69E-15 | 2.02E-14 |
| UNC80 | -2.09351 | -2.24213 | 5.12E-15 | 2.20E-14 |
| HEPACAM | -2.41267 | -3.0908 | 5.84E-15 | 2.50E-14 |
| ATP2B3 | -2.45205 | -2.38197 | 7.43E-15 | 3.16E-14 |
| STON1-GTF2A1L | -2.15002 | -2.96842 | 8.31E-15 | 3.52E-14 |
| FCRL1 | -2.17336 | -0.60602 | 1.01E-14 | 4.26E-14 |
| SCG3 | -2.21277 | 0.068179 | 1.41E-14 | 5.91E-14 |
| GPR12 | -2.51135 | -3.38696 | 1.61E-14 | 6.70E-14 |
| KCNK2 | -2.00619 | -1.41786 | 2.02E-14 | 8.36E-14 |
| ACTL6B | -2.266 | -3.07538 | 2.03E-14 | 8.39E-14 |
| KCTD8 | -2.46295 | -2.88439 | 3.25E-14 | 1.32E-13 |
| CLCA1 | -2.58264 | 8.712319 | 4.40E-14 | 1.78E-13 |
| HOXD12 | -2.16665 | -2.20954 | 4.68E-14 | 1.88E-13 |
| THBS4 | -2.05865 | 3.667464 | 5.64E-14 | 2.25E-13 |
| GATA5 | -2.46146 | -1.0879 | 6.17E-14 | 2.45E-13 |
| HHATL | -2.13011 | -3.60528 | 6.33E-14 | 2.52E-13 |
| SNCB | -2.01063 | -2.9294 | 6.68E-14 | 2.65E-13 |
| SVOPL | -2.26174 | -0.61673 | 8.81E-14 | 3.47E-13 |
| MPPED1 | -2.12688 | -3.47385 | 8.84E-14 | 3.49E-13 |
| ADGRG4 | -2.87414 | -3.27781 | 1.74E-13 | 6.69E-13 |
| KCNJ16 | -2.00499 | -1.79402 | 2.47E-13 | 9.43E-13 |
| PCDH11X | -2.24039 | -3.55542 | 2.96E-13 | 1.12E-12 |
| KLHL34 | -2.02235 | -0.34778 | 5.54E-13 | 2.06E-12 |
| DRD5 | -2.65856 | -1.27562 | 7.12E-13 | 2.63E-12 |
| LIX1 | -2.12402 | -2.28671 | 7.17E-13 | 2.65E-12 |
| CARTPT | -2.38806 | -0.91949 | 1.14E-12 | 4.16E-12 |
| SMPX | -2.26883 | 0.0884 | 1.24E-12 | 4.50E-12 |
| UGT1A3 | -2.15373 | -3.19805 | 1.67E-12 | 6.00E-12 |
| NUPR2 | -2.26472 | -2.83542 | 1.09E-11 | 3.70E-11 |
| PIK3C2G | -2.33472 | -2.91513 | 1.10E-11 | 3.73E-11 |
| CHST9 | -2.42045 | -2.0845 | 2.12E-11 | 7.02E-11 |
| ITLN1 | -2.18529 | 6.672735 | 2.96E-11 | 9.72E-11 |
| AQP4 | -2.17829 | -2.97551 | 5.54E-11 | 1.78E-10 |
| HTR3C | -2.39912 | -2.0257 | 6.19E-11 | 1.98E-10 |
| CPB1 | -2.40851 | -1.24728 | 6.86E-11 | 2.18E-10 |
| DPYSL5 | -2.04458 | -1.44713 | 1.33E-10 | 4.14E-10 |
| TMPRSS15 | -2.56999 | -2.64891 | 5.39E-10 | 1.61E-09 |
| HSPB3 | -2.12688 | -0.52414 | 7.23E-10 | 2.13E-09 |
| GPR26 | -2.09083 | -3.46041 | 9.07E-10 | 2.65E-09 |
| IRX6 | -2.07496 | -3.08224 | 1.65E-09 | 4.72E-09 |
| GLYATL3 | -2.21628 | -3.56117 | 1.91E-09 | 5.42E-09 |
| NAT8 | -2.05935 | -0.66753 | 2.68E-09 | 7.50E-09 |
| MTRNR2L1 | -2.2534 | 3.42629 | 2.76E-09 | 7.73E-09 |
| SERTM1 | -2.16749 | -3.24567 | 7.74E-09 | 2.08E-08 |
| CCKAR | -2.13487 | -3.18424 | 2.84E-08 | 7.30E-08 |
| FUT9 | -2.3765 | -2.87219 | 5.49E-08 | 1.38E-07 |

| **Table S2:** **The summary table of DElncRNA** | | | | |
| --- | --- | --- | --- | --- |
| **lncRNA** | **logFC** | **logCPM** | **PValue** | **FDR** |
| AC016027.1 | -2.62414 | 7.956381 | 2.83E-169 | 2.05E-165 |
| CDKN2B-AS1 | -5.10395 | 9.257131 | 2.37E-140 | 8.58E-137 |
| LINC01645 | -4.53228 | 4.655391 | 8.96E-138 | 2.17E-134 |
| AC005358.1 | -4.40934 | 4.483629 | 3.26E-131 | 5.91E-128 |
| AC087379.1 | -4.97556 | 7.077173 | 4.71E-130 | 6.84E-127 |
| AC010442.1 | -3.39082 | 12.71046 | 2.47E-128 | 2.98E-125 |
| AC007182.1 | -4.62925 | 6.083001 | 8.77E-120 | 9.08E-117 |
| HAND2-AS1 | -4.71504 | 8.104153 | 9.24E-115 | 8.38E-112 |
| AC073283.2 | -4.07783 | 4.465976 | 1.81E-110 | 1.46E-107 |
| LINC00682 | -4.5871 | 3.435517 | 3.07E-109 | 2.22E-106 |
| AC036108.3 | -3.3815 | 6.327514 | 5.66E-104 | 3.73E-101 |
| MBNL1-AS1 | -2.88034 | 9.031636 | 6.58E-103 | 3.98E-100 |
| TMEM220-AS1 | -2.85712 | 6.516038 | 1.35E-100 | 7.54E-98 |
| AC020978.4 | -2.93743 | 5.695832 | 6.87E-100 | 3.56E-97 |
| EPCAM-DT | -2.67046 | 7.334588 | 1.73E-94 | 8.34E-92 |
| LINC00974 | -4.82844 | 4.761242 | 1.94E-92 | 8.79E-90 |
| LINC00092 | -2.75272 | 5.043803 | 7.10E-91 | 3.03E-88 |
| LINC02000 | -3.83452 | 3.918125 | 3.65E-89 | 1.47E-86 |
| AL357054.4 | -2.71428 | 6.805877 | 1.67E-88 | 6.38E-86 |
| MIR497HG | -2.53108 | 5.810121 | 1.88E-88 | 6.80E-86 |
| CA3-AS1 | -3.22 | 5.856012 | 6.22E-86 | 2.15E-83 |
| LINC00507 | -4.31991 | 3.699146 | 3.36E-83 | 1.11E-80 |
| AC015908.3 | -3.12143 | 4.919536 | 4.33E-82 | 1.37E-79 |
| LINC02268 | -3.94727 | 3.394417 | 3.25E-81 | 9.81E-79 |
| AC020779.2 | -3.14881 | 4.141843 | 1.72E-78 | 4.99E-76 |
| PDE9A-AS1 | -3.60507 | 4.902369 | 3.82E-76 | 1.06E-73 |
| AC092652.1 | -4.20903 | 3.455182 | 1.53E-75 | 4.12E-73 |
| AL353801.1 | -3.19946 | 3.63032 | 2.07E-74 | 5.37E-72 |
| PGM5-AS1 | -4.92997 | 5.443266 | 6.17E-74 | 1.49E-71 |
| AC021491.4 | -2.61676 | 4.958616 | 1.15E-73 | 2.69E-71 |
| PVT1 | 2.635642 | 10.69924 | 2.27E-73 | 5.13E-71 |
| LINC02490 | -4.86719 | 4.495907 | 1.56E-72 | 3.43E-70 |
| AC092723.1 | -3.52718 | 5.540197 | 2.10E-71 | 4.47E-69 |
| EDIL3-DT | -2.60985 | 4.921132 | 7.06E-71 | 1.46E-68 |
| AC103740.1 | -2.60644 | 6.735145 | 1.26E-69 | 2.54E-67 |
| ADAMTS9-AS1 | -3.74197 | 6.549179 | 2.11E-69 | 4.14E-67 |
| AP000866.2 | -2.21991 | 6.86953 | 4.84E-69 | 9.24E-67 |
| AL121790.2 | -3.85437 | 5.459256 | 4.25E-68 | 7.51E-66 |
| MEF2C-AS1 | -3.00127 | 4.768666 | 2.89E-67 | 4.98E-65 |
| BVES-AS1 | -3.46334 | 4.015863 | 6.21E-67 | 1.05E-64 |
| LINC02023 | -4.13971 | 5.380563 | 1.74E-66 | 2.87E-64 |
| AL132642.1 | -3.2658 | 4.071835 | 2.54E-65 | 4.00E-63 |
| ADAMTS9-AS2 | -2.98995 | 5.417563 | 3.77E-65 | 5.82E-63 |
| AC068189.1 | -3.07175 | 3.969021 | 1.53E-64 | 2.26E-62 |
| UBXN10-AS1 | -2.98024 | 6.033999 | 5.81E-64 | 8.43E-62 |
| LINC00891 | -3.25973 | 3.567073 | 1.11E-63 | 1.58E-61 |
| AF001548.1 | -3.60751 | 4.640178 | 8.30E-63 | 1.16E-60 |
| AP001107.5 | -3.1127 | 5.485954 | 1.04E-62 | 1.42E-60 |
| CASC18 | -3.02344 | 4.226492 | 2.08E-62 | 2.80E-60 |
| LINC02038 | -2.10007 | 7.395776 | 1.06E-61 | 1.40E-59 |
| MIR3150BHG | -2.97604 | 5.416207 | 1.65E-61 | 2.14E-59 |
| AC004947.1 | -3.31508 | 4.260795 | 1.96E-60 | 2.49E-58 |
| AC087379.2 | -3.66621 | 3.328359 | 2.85E-60 | 3.50E-58 |
| AP001554.1 | -3.38461 | 5.318587 | 4.03E-60 | 4.79E-58 |
| B3GALT5-AS1 | -4.11731 | 7.51841 | 5.91E-60 | 6.91E-58 |
| MIR1-1HG-AS1 | -3.52568 | 5.930836 | 1.50E-59 | 1.72E-57 |
| AC004707.2 | -2.76706 | 4.451247 | 1.82E-58 | 2.06E-56 |
| MAFG-DT | 2.960539 | 9.475187 | 4.14E-58 | 4.62E-56 |
| AC012085.2 | -4.33117 | 5.772599 | 4.14E-57 | 4.55E-55 |
| AC073050.1 | -3.31374 | 3.83024 | 5.61E-57 | 5.98E-55 |
| LINC01082 | -2.71931 | 6.587808 | 8.16E-57 | 8.57E-55 |
| CRNDE | 4.644295 | 9.006414 | 1.79E-56 | 1.85E-54 |
| AP000944.1 | -2.74658 | 3.522285 | 6.80E-56 | 6.94E-54 |
| AC009102.2 | -3.53264 | 3.891638 | 3.88E-55 | 3.90E-53 |
| AC110995.1 | -2.94587 | 4.382329 | 5.14E-55 | 5.10E-53 |
| LINC02163 | 7.08454 | 6.253479 | 7.84E-55 | 7.68E-53 |
| CASC19 | 4.522766 | 7.19662 | 1.28E-54 | 1.23E-52 |
| AL158071.1 | -3.183 | 4.06268 | 1.30E-54 | 1.24E-52 |
| SEMA6A-AS2 | -2.74366 | 3.616769 | 1.48E-54 | 1.39E-52 |
| AC104407.1 | -4.28131 | 3.670541 | 4.30E-54 | 3.99E-52 |
| AC079313.2 | -3.7536 | 4.032862 | 7.89E-54 | 7.15E-52 |
| LINC02577 | 6.327387 | 7.703866 | 3.37E-53 | 3.02E-51 |
| AC130371.2 | -2.23021 | 5.557683 | 3.89E-53 | 3.44E-51 |
| AC135586.2 | -2.47599 | 3.473516 | 4.20E-52 | 3.67E-50 |
| AC135012.3 | -2.50602 | 5.917507 | 4.46E-52 | 3.85E-50 |
| TARID | -2.37788 | 5.210875 | 5.68E-52 | 4.84E-50 |
| LINC01505 | -2.9895 | 3.284014 | 6.10E-52 | 5.14E-50 |
| LINC02185 | -2.85854 | 3.551091 | 7.18E-52 | 5.98E-50 |
| CHL1-AS2 | -3.04533 | 3.522304 | 1.84E-51 | 1.51E-49 |
| AC016888.1 | -2.27361 | 7.761659 | 1.94E-51 | 1.58E-49 |
| IL6R-AS1 | -2.33893 | 3.960674 | 2.22E-51 | 1.79E-49 |
| AC115619.1 | -6.54826 | 3.616916 | 4.84E-51 | 3.82E-49 |
| JAZF1-AS1 | -2.86857 | 3.557927 | 7.62E-51 | 5.94E-49 |
| LINC01634 | -3.19522 | 3.400953 | 8.40E-51 | 6.48E-49 |
| ELFN1-AS1 | 4.876138 | 10.03358 | 9.56E-51 | 7.29E-49 |
| LINC02568 | -3.06715 | 7.47229 | 1.29E-50 | 9.78E-49 |
| AC144831.1 | -2.18425 | 6.6424 | 3.87E-50 | 2.89E-48 |
| AL356489.2 | -3.32418 | 5.01263 | 3.95E-50 | 2.92E-48 |
| LINC02418 | 8.837695 | 11.07083 | 9.80E-50 | 7.18E-48 |
| AC007128.1 | 4.507468 | 6.824538 | 1.21E-49 | 8.73E-48 |
| AC010776.2 | -4.14301 | 3.804572 | 1.22E-49 | 8.73E-48 |
| AC024651.1 | -4.26364 | 3.415142 | 1.38E-49 | 9.80E-48 |
| LINC01055 | -2.89139 | 4.121208 | 1.40E-49 | 9.83E-48 |
| LINC01537 | -2.51376 | 4.312123 | 2.30E-49 | 1.60E-47 |
| AL391807.1 | -2.93362 | 3.612915 | 2.75E-49 | 1.90E-47 |
| AC110491.1 | -4.45874 | 3.642257 | 4.84E-49 | 3.31E-47 |
| AL138995.1 | -2.24994 | 4.489331 | 1.94E-48 | 1.32E-46 |
| AL162424.1 | -2.3224 | 3.754139 | 2.52E-48 | 1.69E-46 |
| AC004147.4 | -3.70569 | 3.200125 | 6.72E-48 | 4.47E-46 |
| AC009133.3 | -2.76686 | 4.405523 | 1.11E-47 | 7.29E-46 |
| LINC01954 | -3.14398 | 4.240836 | 1.91E-47 | 1.25E-45 |
| AL513217.1 | -3.26515 | 3.631662 | 7.20E-47 | 4.58E-45 |
| FENDRR | -2.09409 | 10.65384 | 7.74E-47 | 4.88E-45 |
| LINC02033 | -2.40479 | 4.004485 | 1.23E-46 | 7.64E-45 |
| AL121974.1 | -5.94668 | 3.917592 | 2.38E-46 | 1.46E-44 |
| LINC01013 | -2.58113 | 4.337582 | 4.03E-46 | 2.46E-44 |
| AF165147.1 | -2.44322 | 4.984973 | 1.98E-45 | 1.19E-43 |
| AC093607.1 | -3.3363 | 3.662296 | 6.38E-45 | 3.79E-43 |
| AL445426.1 | -2.63646 | 3.642534 | 8.05E-45 | 4.74E-43 |
| CLCA4-AS1 | -2.43328 | 4.133799 | 8.62E-45 | 5.04E-43 |
| AL109615.3 | 4.544801 | 7.960241 | 8.76E-45 | 5.08E-43 |
| LINC01624 | -2.52162 | 3.887143 | 9.13E-45 | 5.26E-43 |
| RBMS3-AS3 | -2.7914 | 3.476853 | 1.19E-44 | 6.82E-43 |
| MRGPRF-AS1 | -2.65816 | 3.827672 | 2.02E-43 | 1.12E-41 |
| AC040168.1 | -2.65182 | 3.998309 | 2.06E-43 | 1.13E-41 |
| LINC00582 | -2.67094 | 4.261007 | 2.20E-43 | 1.20E-41 |
| LINC01475 | -2.55457 | 4.494523 | 2.27E-43 | 1.23E-41 |
| LINC00659 | 5.973774 | 7.452869 | 5.32E-43 | 2.82E-41 |
| LIFR-AS1 | -2.39168 | 4.568981 | 9.90E-43 | 5.16E-41 |
| LINC02829 | -3.34309 | 3.883815 | 1.09E-42 | 5.63E-41 |
| AC011306.1 | -3.11683 | 3.469717 | 1.36E-42 | 6.99E-41 |
| FEZF1-AS1 | 8.715625 | 9.094692 | 1.63E-42 | 8.31E-41 |
| AC022034.1 | -2.5377 | 6.893132 | 1.69E-42 | 8.50E-41 |
| AC007099.1 | 7.278153 | 6.69521 | 1.76E-42 | 8.81E-41 |
| AC005674.1 | -2.29165 | 3.913539 | 3.75E-42 | 1.86E-40 |
| AC104024.1 | -2.84139 | 3.844433 | 9.97E-42 | 4.88E-40 |
| AC123023.1 | 5.852137 | 6.812839 | 1.44E-41 | 6.98E-40 |
| AP002358.1 | -3.08734 | 3.61899 | 2.56E-41 | 1.23E-39 |
| LINC00955 | -3.70199 | 4.857676 | 2.56E-41 | 1.23E-39 |
| AL365361.1 | -2.55671 | 7.380405 | 2.96E-41 | 1.41E-39 |
| LINC01140 | -2.13094 | 5.678507 | 5.22E-41 | 2.47E-39 |
| AL596223.1 | -2.38917 | 5.643826 | 6.56E-41 | 3.09E-39 |
| LINC02441 | -2.57931 | 8.067271 | 1.23E-40 | 5.73E-39 |
| AC005089.1 | 3.944011 | 6.565616 | 1.87E-40 | 8.62E-39 |
| AC123912.4 | -3.57973 | 4.667739 | 2.61E-40 | 1.19E-38 |
| FGF14-AS2 | -2.22437 | 5.623256 | 3.20E-40 | 1.45E-38 |
| LINC02292 | -2.27246 | 4.365792 | 7.95E-40 | 3.56E-38 |
| DIRC3 | -2.39494 | 4.795478 | 8.38E-40 | 3.73E-38 |
| AL022318.1 | -2.90876 | 3.951221 | 1.79E-39 | 7.93E-38 |
| AC007128.2 | 4.653053 | 5.176401 | 2.03E-39 | 8.89E-38 |
| AC002398.2 | -3.48043 | 3.598164 | 3.35E-39 | 1.46E-37 |
| PLUT | 4.909484 | 5.932563 | 4.22E-39 | 1.82E-37 |
| NKAIN3-IT1 | -3.27062 | 5.208019 | 5.46E-39 | 2.33E-37 |
| FAM222A-AS1 | 3.460625 | 7.029933 | 8.21E-39 | 3.48E-37 |
| OSTN-AS1 | -2.96126 | 3.504201 | 9.45E-39 | 3.98E-37 |
| ARHGAP15-AS1 | -3.67294 | 3.348611 | 1.13E-38 | 4.75E-37 |
| AL034346.1 | -2.20663 | 4.311896 | 4.76E-38 | 1.97E-36 |
| FRMD6-AS2 | -3.40657 | 3.41675 | 2.10E-37 | 8.61E-36 |
| DDN-AS1 | 3.020468 | 7.124451 | 2.90E-37 | 1.18E-35 |
| LINC02057 | -2.69857 | 5.228644 | 4.47E-37 | 1.81E-35 |
| LINC00460 | 6.57951 | 7.820211 | 6.18E-37 | 2.49E-35 |
| AC055717.2 | 7.477731 | 6.623165 | 6.77E-37 | 2.71E-35 |
| AC005180.2 | -2.9988 | 4.821537 | 7.26E-37 | 2.89E-35 |
| AC093627.6 | -2.88503 | 4.389888 | 9.95E-37 | 3.94E-35 |
| PRR7-AS1 | 2.297878 | 6.728208 | 1.12E-36 | 4.41E-35 |
| LINC01975 | -2.62494 | 3.772804 | 3.07E-36 | 1.19E-34 |
| AL590708.1 | 2.902886 | 6.689838 | 3.87E-36 | 1.48E-34 |
| LINC01811 | 5.798629 | 6.124962 | 4.76E-36 | 1.82E-34 |
| AC007384.1 | -2.13335 | 5.688805 | 4.99E-36 | 1.89E-34 |
| AC015908.2 | -2.2727 | 5.149759 | 5.63E-36 | 2.12E-34 |
| LINC00461 | -2.74781 | 3.983677 | 7.68E-36 | 2.87E-34 |
| SPRY4-AS1 | 2.84713 | 5.597977 | 9.43E-36 | 3.51E-34 |
| LINC01352 | -2.18052 | 3.725319 | 9.74E-36 | 3.60E-34 |
| LINC01977 | 3.599064 | 6.408672 | 1.36E-35 | 5.02E-34 |
| LINC01605 | 3.065926 | 8.238154 | 2.10E-35 | 7.68E-34 |
| AC005180.1 | -3.04122 | 4.448166 | 3.71E-35 | 1.35E-33 |
| AC126178.1 | -2.83483 | 4.256294 | 5.98E-35 | 2.17E-33 |
| LINC00858 | 5.844816 | 7.517107 | 1.05E-34 | 3.78E-33 |
| AC092834.1 | -3.3815 | 4.592481 | 1.66E-34 | 5.88E-33 |
| AL391845.2 | 4.538844 | 5.4751 | 3.17E-34 | 1.10E-32 |
| AP000802.1 | -2.53516 | 3.160579 | 3.75E-34 | 1.30E-32 |
| FOXP4-AS1 | 2.237251 | 8.151609 | 4.03E-34 | 1.39E-32 |
| VAC14-AS1 | 2.96092 | 6.238723 | 5.50E-34 | 1.88E-32 |
| GAS1RR | -2.70484 | 4.615678 | 5.86E-34 | 1.99E-32 |
| LINC01752 | -2.35942 | 7.266078 | 6.01E-34 | 2.03E-32 |
| FIRRE | 5.453136 | 7.591233 | 6.14E-34 | 2.06E-32 |
| AL356299.3 | 2.58626 | 5.655112 | 8.00E-34 | 2.66E-32 |
| LINC01705 | 6.704993 | 6.30535 | 8.64E-34 | 2.85E-32 |
| AL355607.1 | -2.99323 | 3.510199 | 8.90E-34 | 2.92E-32 |
| LINC02613 | -2.41034 | 4.244345 | 1.09E-33 | 3.55E-32 |
| AC015922.2 | -2.00484 | 6.953237 | 1.76E-33 | 5.68E-32 |
| AC133644.1 | -2.3306 | 4.637367 | 2.49E-33 | 8.03E-32 |
| BBOX1-AS1 | 4.653826 | 8.304216 | 3.63E-33 | 1.16E-31 |
| VPS9D1-AS1 | 2.443294 | 10.5965 | 4.64E-33 | 1.48E-31 |
| HAGLR | -2.25409 | 10.63639 | 5.27E-33 | 1.68E-31 |
| AC011365.2 | -2.37842 | 5.176032 | 1.22E-32 | 3.84E-31 |
| AC116025.2 | 3.893865 | 4.675725 | 1.81E-32 | 5.66E-31 |
| LINC01354 | -2.46587 | 4.231325 | 2.33E-32 | 7.26E-31 |
| AC011365.3 | -2.07056 | 3.421901 | 4.01E-32 | 1.24E-30 |
| AC124067.2 | 3.408985 | 8.52888 | 4.47E-32 | 1.38E-30 |
| DTNB-AS1 | -2.47345 | 5.048901 | 8.45E-32 | 2.60E-30 |
| AC009522.1 | -3.14782 | 3.206016 | 1.13E-31 | 3.41E-30 |
| SFTA1P | -2.57271 | 4.774462 | 1.61E-31 | 4.79E-30 |
| AL049836.1 | 3.242703 | 7.124332 | 2.50E-31 | 7.40E-30 |
| AP003071.4 | -2.23922 | 6.11636 | 3.08E-31 | 9.08E-30 |
| DLGAP1-AS2 | 2.110919 | 10.24971 | 4.06E-31 | 1.19E-29 |
| AC024337.2 | -2.21238 | 3.752056 | 4.88E-31 | 1.41E-29 |
| LINC01979 | 2.931228 | 7.97722 | 1.15E-30 | 3.29E-29 |
| AC010336.2 | -2.28585 | 3.395932 | 1.75E-30 | 4.91E-29 |
| LINC01080 | -2.8331 | 3.58413 | 2.45E-30 | 6.78E-29 |
| AP000697.1 | 5.055874 | 4.256196 | 2.88E-30 | 7.93E-29 |
| LINC02595 | 3.400587 | 5.049605 | 7.08E-30 | 1.92E-28 |
| LINC01687 | -3.4513 | 4.356869 | 8.97E-30 | 2.42E-28 |
| AL513542.1 | -2.29313 | 3.593678 | 1.24E-29 | 3.34E-28 |
| AL161431.1 | 5.873846 | 10.1502 | 1.54E-29 | 4.11E-28 |
| AC134312.1 | -2.44938 | 4.262291 | 2.42E-29 | 6.45E-28 |
| AL355312.4 | 4.346362 | 7.560724 | 2.51E-29 | 6.66E-28 |
| AC009097.4 | -2.27401 | 3.766463 | 2.55E-29 | 6.76E-28 |
| MIR17HG | 2.488287 | 8.573799 | 2.66E-29 | 7.00E-28 |
| AC007556.1 | -3.0468 | 3.231676 | 3.12E-29 | 8.18E-28 |
| NPSR1-AS1 | 6.209299 | 6.73652 | 5.43E-29 | 1.41E-27 |
| AC027601.1 | 2.550444 | 7.939819 | 5.75E-29 | 1.49E-27 |
| LINC02716 | -2.2049 | 3.817352 | 6.86E-29 | 1.77E-27 |
| AL592429.2 | -2.77778 | 3.099234 | 7.03E-29 | 1.81E-27 |
| AC005392.2 | -2.80321 | 7.039761 | 7.24E-29 | 1.85E-27 |
| TRPC7-AS1 | -2.17401 | 5.149304 | 1.26E-28 | 3.22E-27 |
| LINC01978 | 3.524029 | 8.478027 | 1.74E-28 | 4.42E-27 |
| AC026369.2 | 2.547432 | 5.9444 | 1.92E-28 | 4.87E-27 |
| AC022034.4 | -2.56598 | 3.251199 | 2.14E-28 | 5.38E-27 |
| AC105219.3 | 2.510301 | 6.357718 | 2.63E-28 | 6.55E-27 |
| LINC01798 | -2.31366 | 3.766628 | 2.67E-28 | 6.64E-27 |
| AC004637.1 | -2.35073 | 3.594155 | 3.40E-28 | 8.39E-27 |
| LINC02253 | 5.412145 | 8.084333 | 4.25E-28 | 1.05E-26 |
| GAS6-AS1 | 3.207812 | 10.76426 | 5.20E-28 | 1.27E-26 |
| AL163953.1 | 3.432593 | 6.443587 | 7.68E-28 | 1.88E-26 |
| LINC00940 | -2.56864 | 5.769068 | 8.15E-28 | 1.98E-26 |
| AP003548.1 | -2.07348 | 3.402605 | 1.07E-27 | 2.56E-26 |
| AL121987.1 | -2.16343 | 3.375696 | 1.27E-27 | 3.03E-26 |
| AC078923.1 | 4.754686 | 5.379932 | 2.50E-27 | 5.94E-26 |
| LINC01781 | -2.46439 | 4.28109 | 2.74E-27 | 6.48E-26 |
| AC126773.4 | 2.093839 | 5.537954 | 5.00E-27 | 1.17E-25 |
| AC104958.2 | 2.729365 | 9.162872 | 6.42E-27 | 1.49E-25 |
| LINC01234 | 7.560809 | 8.99283 | 6.94E-27 | 1.60E-25 |
| FAM30A | -2.368 | 8.003932 | 8.31E-27 | 1.91E-25 |
| LINC01356 | 2.46668 | 6.889334 | 9.09E-27 | 2.09E-25 |
| AL109910.2 | -2.42598 | 3.316575 | 1.18E-26 | 2.70E-25 |
| AC025154.2 | 5.472087 | 6.159792 | 1.21E-26 | 2.77E-25 |
| AC027807.2 | -2.34774 | 3.131094 | 1.22E-26 | 2.77E-25 |
| AC105219.1 | 4.145893 | 4.960079 | 1.34E-26 | 3.03E-25 |
| AL512306.3 | -2.19222 | 3.680495 | 1.61E-26 | 3.64E-25 |
| LINC02320 | 2.884894 | 6.093131 | 2.27E-26 | 5.08E-25 |
| LINC02798 | -2.17951 | 4.241316 | 3.69E-26 | 8.18E-25 |
| LINC02408 | -2.14897 | 4.138497 | 4.15E-26 | 9.17E-25 |
| LINC01016 | -2.1554 | 3.872326 | 1.16E-25 | 2.50E-24 |
| AC107959.3 | 2.98055 | 6.145303 | 1.38E-25 | 2.97E-24 |
| AL590483.1 | 3.302924 | 5.059719 | 1.47E-25 | 3.14E-24 |
| AC005256.1 | 6.032841 | 5.671103 | 1.57E-25 | 3.35E-24 |
| AL121832.1 | 3.861588 | 7.212527 | 1.63E-25 | 3.46E-24 |
| AC104024.2 | 4.163303 | 5.456243 | 1.64E-25 | 3.47E-24 |
| LINC02723 | -2.00241 | 3.24271 | 2.74E-25 | 5.74E-24 |
| EMSLR | 2.812661 | 9.337041 | 3.38E-25 | 7.04E-24 |
| LINC02427 | -2.01535 | 3.539555 | 3.76E-25 | 7.81E-24 |
| FABP6-AS1 | -2.70441 | 3.733265 | 4.33E-25 | 8.97E-24 |
| AC036108.2 | -2.34551 | 3.639633 | 4.57E-25 | 9.44E-24 |
| LINC02257 | 5.15262 | 5.023807 | 5.03E-25 | 1.03E-23 |
| AC026336.3 | 8.41688 | 7.780842 | 5.09E-25 | 1.04E-23 |
| CADM3-AS1 | -2.40408 | 5.167897 | 5.29E-25 | 1.08E-23 |
| AC117386.2 | 5.720179 | 5.506615 | 5.53E-25 | 1.13E-23 |
| AC087482.1 | -2.41339 | 4.753077 | 5.87E-25 | 1.19E-23 |
| AC100791.2 | 3.930473 | 4.96564 | 6.12E-25 | 1.24E-23 |
| LINC01748 | 3.710615 | 7.905855 | 6.21E-25 | 1.25E-23 |
| AL161457.1 | -2.81513 | 3.109663 | 6.59E-25 | 1.33E-23 |
| SLCO4A1-AS1 | 3.491017 | 10.26054 | 9.33E-25 | 1.87E-23 |
| GCSIR | -2.05041 | 3.292948 | 1.11E-24 | 2.21E-23 |
| LINC02245 | -2.1639 | 3.249565 | 1.30E-24 | 2.59E-23 |
| AC124319.1 | 3.305928 | 6.663018 | 1.31E-24 | 2.61E-23 |
| AC016831.1 | 2.252434 | 8.577814 | 1.46E-24 | 2.88E-23 |
| CYP4A22-AS1 | 2.08025 | 5.232232 | 1.54E-24 | 3.01E-23 |
| AC106900.1 | 2.347816 | 6.058722 | 2.37E-24 | 4.60E-23 |
| AC104971.3 | -2.43432 | 3.168136 | 2.39E-24 | 4.62E-23 |
| AL162582.1 | 5.954879 | 6.750297 | 3.01E-24 | 5.76E-23 |
| LINC01829 | -2.5723 | 4.107796 | 3.35E-24 | 6.36E-23 |
| LINC02132 | -2.29 | 3.219947 | 3.41E-24 | 6.45E-23 |
| AP005233.2 | 3.646245 | 6.785697 | 4.09E-24 | 7.66E-23 |
| AC007406.3 | 2.470571 | 5.45576 | 4.61E-24 | 8.55E-23 |
| LINC00402 | -2.38152 | 4.280561 | 5.12E-24 | 9.47E-23 |
| AL391056.1 | 3.029447 | 8.368598 | 5.91E-24 | 1.09E-22 |
| GRIK1-AS1 | -2.01332 | 3.300405 | 6.28E-24 | 1.16E-22 |
| LINC01614 | 5.294209 | 6.140562 | 6.60E-24 | 1.21E-22 |
| MIR503HG | 2.908249 | 7.386124 | 7.80E-24 | 1.42E-22 |
| BX470102.1 | 2.240392 | 8.255646 | 8.75E-24 | 1.59E-22 |
| C2-AS1 | 2.780311 | 4.697973 | 1.32E-23 | 2.37E-22 |
| SCAT1 | 3.313008 | 6.03556 | 1.33E-23 | 2.39E-22 |
| AF064860.2 | -2.38435 | 4.15701 | 1.46E-23 | 2.61E-22 |
| AC104823.1 | 7.798815 | 9.170395 | 1.90E-23 | 3.37E-22 |
| AC021218.1 | 2.255732 | 11.23179 | 2.02E-23 | 3.57E-22 |
| LINC01836 | 2.599413 | 6.229678 | 2.21E-23 | 3.91E-22 |
| AC091182.2 | 3.035257 | 5.604597 | 3.48E-23 | 6.10E-22 |
| AC022733.1 | -2.26321 | 3.229896 | 3.61E-23 | 6.30E-22 |
| LINC01357 | 2.579446 | 6.368045 | 4.90E-23 | 8.46E-22 |
| PLAC4 | 4.485977 | 8.346556 | 4.98E-23 | 8.58E-22 |
| AC026336.2 | 5.323334 | 4.280998 | 5.44E-23 | 9.35E-22 |
| AL136366.1 | -2.02358 | 3.29192 | 5.55E-23 | 9.51E-22 |
| LINC02397 | -2.29884 | 4.585046 | 6.26E-23 | 1.06E-21 |
| AC105429.1 | 2.075809 | 6.41134 | 6.42E-23 | 1.09E-21 |
| AC110813.1 | -3.20378 | 4.991702 | 6.67E-23 | 1.12E-21 |
| LINC01797 | -2.6302 | 3.078557 | 7.21E-23 | 1.21E-21 |
| AC010719.1 | 2.041548 | 8.026409 | 7.62E-23 | 1.28E-21 |
| LINC01594 | 3.886059 | 5.752475 | 8.10E-23 | 1.36E-21 |
| PCAT18 | -2.88518 | 4.164852 | 8.47E-23 | 1.41E-21 |
| AC020656.2 | 3.786697 | 8.293649 | 9.10E-23 | 1.51E-21 |
| GPRACR | -2.48299 | 3.544885 | 9.60E-23 | 1.59E-21 |
| AC013287.1 | 3.661824 | 4.509683 | 1.05E-22 | 1.74E-21 |
| LINC01411 | 6.630078 | 7.92498 | 1.43E-22 | 2.35E-21 |
| LEF1-AS1 | 2.03738 | 5.319052 | 1.45E-22 | 2.37E-21 |
| FOXD3-AS1 | -2.61666 | 4.465638 | 1.50E-22 | 2.46E-21 |
| AP003071.3 | -2.30421 | 3.190384 | 1.65E-22 | 2.69E-21 |
| AC026704.1 | 2.000057 | 5.817611 | 1.98E-22 | 3.21E-21 |
| AC073283.1 | 3.023657 | 5.715542 | 2.65E-22 | 4.28E-21 |
| LINC01615 | 3.148866 | 5.312435 | 5.00E-22 | 7.90E-21 |
| LINC00488 | -2.60738 | 3.853904 | 5.01E-22 | 7.90E-21 |
| AC099792.1 | 3.538051 | 6.473282 | 5.06E-22 | 7.95E-21 |
| AC009336.1 | -2.02732 | 3.933605 | 6.88E-22 | 1.08E-20 |
| AL590004.3 | 4.080116 | 6.599372 | 7.59E-22 | 1.18E-20 |
| AL353150.1 | 2.41653 | 6.880877 | 8.90E-22 | 1.38E-20 |
| LINC01730 | 2.718956 | 5.590786 | 1.30E-21 | 2.02E-20 |
| LINC01783 | -2.29831 | 3.650187 | 1.56E-21 | 2.39E-20 |
| AC106772.1 | 3.488972 | 4.293611 | 1.56E-21 | 2.39E-20 |
| LINC02554 | -2.59622 | 3.559203 | 1.69E-21 | 2.58E-20 |
| SH3PXD2A-AS1 | 2.763191 | 9.114843 | 1.80E-21 | 2.74E-20 |
| AL359881.1 | 3.872557 | 4.822736 | 1.88E-21 | 2.86E-20 |
| AP005230.1 | 3.653673 | 5.03299 | 2.27E-21 | 3.44E-20 |
| AC135388.1 | 5.997853 | 4.789029 | 2.88E-21 | 4.33E-20 |
| AC021683.1 | -2.42413 | 5.553636 | 2.93E-21 | 4.38E-20 |
| LMO7-AS1 | 2.548443 | 5.454815 | 3.13E-21 | 4.67E-20 |
| TRPM2-AS | 3.071462 | 9.051262 | 3.64E-21 | 5.38E-20 |
| SNHG25 | 4.526982 | 9.6318 | 3.85E-21 | 5.66E-20 |
| AC003965.2 | 2.993855 | 5.414791 | 4.76E-21 | 6.96E-20 |
| LINC01433 | 2.5725 | 3.949985 | 5.07E-21 | 7.38E-20 |
| AC022893.2 | -2.31115 | 3.687065 | 5.28E-21 | 7.68E-20 |
| AL157931.1 | -2.86907 | 3.377626 | 5.66E-21 | 8.17E-20 |
| AP004609.3 | -2.36856 | 3.390518 | 5.91E-21 | 8.52E-20 |
| AC020659.1 | 3.937461 | 8.066402 | 6.53E-21 | 9.40E-20 |
| LINC01571 | -2.6062 | 3.369466 | 6.89E-21 | 9.87E-20 |
| LINC01266 | -2.10051 | 3.64507 | 8.22E-21 | 1.17E-19 |
| LINC01996 | 4.743661 | 5.706795 | 1.05E-20 | 1.49E-19 |
| AC093732.1 | 3.525993 | 4.942284 | 1.05E-20 | 1.49E-19 |
| AL031710.2 | 2.032482 | 4.920937 | 1.18E-20 | 1.66E-19 |
| AC048344.4 | 2.251764 | 4.928453 | 1.28E-20 | 1.80E-19 |
| GDNF-AS1 | -2.05291 | 4.257783 | 1.33E-20 | 1.86E-19 |
| AC011676.1 | 2.324797 | 4.885306 | 1.55E-20 | 2.17E-19 |
| GATA2-AS1 | 2.709137 | 8.952493 | 2.11E-20 | 2.92E-19 |
| AC136475.3 | 3.5087 | 9.446515 | 2.61E-20 | 3.59E-19 |
| AC104794.4 | 4.625963 | 4.318942 | 3.24E-20 | 4.44E-19 |
| LINC01711 | 3.180468 | 4.604578 | 3.47E-20 | 4.74E-19 |
| LINC00941 | 4.367629 | 7.174695 | 3.51E-20 | 4.78E-19 |
| AC239584.1 | 4.840651 | 5.138211 | 4.52E-20 | 6.07E-19 |
| LINC01050 | 4.459487 | 4.529576 | 4.65E-20 | 6.21E-19 |
| AC073365.1 | 7.586813 | 6.392537 | 4.70E-20 | 6.27E-19 |
| LINC02616 | -2.77491 | 3.833797 | 5.22E-20 | 6.92E-19 |
| LINC01593 | 4.813308 | 4.206874 | 6.33E-20 | 8.33E-19 |
| AC100803.2 | 3.311071 | 4.232743 | 6.92E-20 | 9.09E-19 |
| AC026368.1 | 2.423492 | 5.348433 | 7.78E-20 | 1.02E-18 |
| AC012363.2 | 3.870468 | 4.813138 | 7.87E-20 | 1.03E-18 |
| TFAP2A-AS1 | 3.439723 | 6.724777 | 8.16E-20 | 1.06E-18 |
| AL513123.1 | 3.060515 | 4.738066 | 9.00E-20 | 1.16E-18 |
| SPATA3-AS1 | 2.107655 | 6.741497 | 1.04E-19 | 1.35E-18 |
| CHN2-AS1 | 3.688749 | 5.682756 | 1.21E-19 | 1.56E-18 |
| AL135924.2 | -2.23195 | 3.981427 | 1.25E-19 | 1.61E-18 |
| AP000851.1 | 5.273972 | 5.93084 | 1.42E-19 | 1.83E-18 |
| PCAT1 | 2.020277 | 5.17624 | 1.43E-19 | 1.83E-18 |
| AL162413.1 | 6.658925 | 5.691769 | 1.54E-19 | 1.96E-18 |
| AC007277.1 | 5.07694 | 7.32864 | 1.79E-19 | 2.27E-18 |
| DNAH17-AS1 | 3.414735 | 4.919031 | 1.97E-19 | 2.48E-18 |
| DUXAP8 | 3.558416 | 7.70572 | 2.44E-19 | 3.07E-18 |
| IGFL2-AS1 | 5.693495 | 7.015394 | 2.71E-19 | 3.40E-18 |
| LINC02866 | -2.26442 | 3.264312 | 2.97E-19 | 3.71E-18 |
| LINC01602 | 6.682734 | 6.03333 | 3.24E-19 | 4.04E-18 |
| LINC00698 | 3.385919 | 4.232489 | 4.27E-19 | 5.29E-18 |
| AC010913.1 | 2.008602 | 5.31322 | 5.51E-19 | 6.77E-18 |
| KRT7-AS | 3.262254 | 5.01812 | 5.69E-19 | 6.98E-18 |
| AC069222.1 | 2.202797 | 5.536632 | 6.24E-19 | 7.64E-18 |
| AC005264.1 | 2.590665 | 3.916315 | 7.28E-19 | 8.85E-18 |
| AC108681.1 | 3.373404 | 3.735736 | 1.02E-18 | 1.22E-17 |
| LINC02223 | 4.542291 | 4.548794 | 1.12E-18 | 1.34E-17 |
| UCA1 | 4.009254 | 11.88762 | 1.25E-18 | 1.50E-17 |
| CLDN10-AS1 | 5.157195 | 5.271623 | 1.40E-18 | 1.68E-17 |
| AL139020.1 | -2.96328 | 3.741212 | 1.86E-18 | 2.21E-17 |
| AC010967.1 | 4.297122 | 3.761793 | 2.98E-18 | 3.51E-17 |
| AC064807.2 | 3.648344 | 5.908293 | 3.59E-18 | 4.20E-17 |
| AL391987.4 | -2.01267 | 3.27292 | 4.73E-18 | 5.50E-17 |
| AP000695.2 | 2.452164 | 4.978336 | 5.65E-18 | 6.51E-17 |
| LINC01124 | 2.42589 | 8.532606 | 6.02E-18 | 6.92E-17 |
| AC244205.1 | -2.01311 | 4.277276 | 6.18E-18 | 7.10E-17 |
| AC078860.2 | 2.788275 | 5.421124 | 7.46E-18 | 8.53E-17 |
| AL391335.1 | -2.31648 | 3.640882 | 7.83E-18 | 8.91E-17 |
| AL137026.1 | -2.05501 | 3.233104 | 9.86E-18 | 1.11E-16 |
| AL662890.1 | 2.776811 | 4.543243 | 1.00E-17 | 1.13E-16 |
| ARHGEF26-AS1 | -2.13193 | 4.817092 | 1.15E-17 | 1.29E-16 |
| LINC02474 | 7.420927 | 6.739564 | 1.24E-17 | 1.39E-16 |
| AC022784.1 | 4.14999 | 5.446088 | 1.50E-17 | 1.66E-16 |
| AC005534.1 | 2.133568 | 4.713776 | 1.79E-17 | 1.97E-16 |
| LINC01351 | -2.98069 | 4.038472 | 1.99E-17 | 2.18E-16 |
| AC090116.1 | 3.765564 | 5.516532 | 2.11E-17 | 2.31E-16 |
| AC011611.3 | 2.942565 | 4.287651 | 2.13E-17 | 2.33E-16 |
| AC012501.2 | 5.755431 | 4.796775 | 2.18E-17 | 2.37E-16 |
| LINC01555 | 2.714156 | 6.752835 | 2.21E-17 | 2.41E-16 |
| LINC01655 | 4.314943 | 3.783027 | 2.40E-17 | 2.61E-16 |
| AC020891.2 | 2.510749 | 4.779608 | 3.00E-17 | 3.24E-16 |
| AL121899.1 | 2.467148 | 6.012749 | 3.72E-17 | 4.00E-16 |
| KCNMB2-AS1 | 3.827044 | 6.655346 | 4.85E-17 | 5.17E-16 |
| AC068580.2 | 5.296938 | 4.575884 | 5.03E-17 | 5.34E-16 |
| AC004988.1 | 3.252128 | 4.935889 | 7.14E-17 | 7.53E-16 |
| AC004080.1 | 5.062101 | 5.9746 | 7.87E-17 | 8.27E-16 |
| AC021242.2 | -2.52656 | 3.345687 | 8.24E-17 | 8.64E-16 |
| AC003958.2 | 5.831737 | 4.833772 | 8.65E-17 | 9.05E-16 |
| AC246787.2 | -2.11389 | 3.596408 | 8.84E-17 | 9.23E-16 |
| MAP3K20-AS1 | 3.828051 | 8.351509 | 9.72E-17 | 1.01E-15 |
| SCAT8 | 2.864059 | 5.960194 | 1.25E-16 | 1.29E-15 |
| AL049539.1 | 2.54635 | 4.634523 | 1.36E-16 | 1.40E-15 |
| LINC01511 | 4.701286 | 5.219569 | 1.37E-16 | 1.41E-15 |
| LINC02345 | 3.251172 | 5.765641 | 1.56E-16 | 1.60E-15 |
| LINC01169 | 5.492048 | 5.76324 | 2.10E-16 | 2.13E-15 |
| EVX1-AS | 4.41895 | 6.194868 | 2.30E-16 | 2.32E-15 |
| AP000553.2 | 2.434203 | 4.25897 | 2.64E-16 | 2.65E-15 |
| AL035458.2 | 2.005426 | 5.459822 | 3.32E-16 | 3.30E-15 |
| AC145207.1 | 2.961801 | 4.104679 | 3.39E-16 | 3.37E-15 |
| LINC00618 | 2.244745 | 4.39801 | 4.19E-16 | 4.13E-15 |
| AC009093.1 | 2.587403 | 6.106048 | 8.66E-16 | 8.41E-15 |
| AC133785.1 | 4.16165 | 4.616759 | 9.02E-16 | 8.75E-15 |
| AP003355.2 | -2.22924 | 3.25522 | 9.44E-16 | 9.14E-15 |
| CNIH3-AS2 | 3.077274 | 4.120193 | 9.49E-16 | 9.17E-15 |
| AC073323.1 | 3.460024 | 3.502612 | 1.07E-15 | 1.03E-14 |
| AC079612.1 | 3.177712 | 3.498084 | 1.10E-15 | 1.06E-14 |
| AL078587.1 | 2.224961 | 5.280836 | 1.33E-15 | 1.27E-14 |
| CCAT2 | 3.65991 | 4.636619 | 1.37E-15 | 1.31E-14 |
| LINC02331 | 4.239539 | 4.213819 | 1.44E-15 | 1.37E-14 |
| SH3TC2-DT | 2.620499 | 4.743976 | 1.46E-15 | 1.38E-14 |
| AC103706.1 | 2.038111 | 5.947061 | 1.81E-15 | 1.71E-14 |
| AL109615.2 | 3.276535 | 4.021933 | 2.05E-15 | 1.93E-14 |
| KCNQ1OT1 | 2.134684 | 10.1032 | 2.49E-15 | 2.33E-14 |
| AL356652.1 | 2.375404 | 4.140766 | 2.97E-15 | 2.77E-14 |
| AC104035.1 | 4.825981 | 5.870372 | 3.87E-15 | 3.58E-14 |
| LINC02266 | 4.622862 | 4.079622 | 4.25E-15 | 3.92E-14 |
| AC007750.1 | 2.703272 | 4.904341 | 4.78E-15 | 4.41E-14 |
| AC078820.2 | 3.471848 | 4.261022 | 5.17E-15 | 4.74E-14 |
| LINC01704 | 2.903021 | 3.311105 | 5.29E-15 | 4.84E-14 |
| AFAP1-AS1 | 5.727068 | 10.49529 | 5.81E-15 | 5.29E-14 |
| HMGA2-AS1 | 2.677576 | 6.505652 | 6.24E-15 | 5.68E-14 |
| AP005271.1 | 3.993899 | 5.129857 | 6.28E-15 | 5.70E-14 |
| AC090709.1 | 3.756894 | 5.733489 | 7.15E-15 | 6.48E-14 |
| H19 | 3.870191 | 12.68796 | 7.22E-15 | 6.53E-14 |
| LINC01929 | 3.108191 | 5.278628 | 9.64E-15 | 8.67E-14 |
| AC104564.4 | 2.951915 | 3.663886 | 9.73E-15 | 8.73E-14 |
| AC079466.1 | 6.829944 | 6.791161 | 1.07E-14 | 9.56E-14 |
| LINC01697 | -2.35625 | 3.504776 | 1.16E-14 | 1.03E-13 |
| LEMD1-AS1 | 3.220341 | 4.34477 | 1.36E-14 | 1.21E-13 |
| AC079145.1 | 2.194059 | 4.522014 | 1.41E-14 | 1.25E-13 |
| AC125616.1 | 3.1161 | 4.880313 | 2.43E-14 | 2.13E-13 |
| AP002478.1 | 3.55976 | 5.089103 | 2.59E-14 | 2.27E-13 |
| LUCAT1 | 2.694836 | 6.107333 | 2.69E-14 | 2.34E-13 |
| TSPEAR-AS2 | 2.665096 | 6.815241 | 3.39E-14 | 2.93E-13 |
| AC093866.1 | 5.996319 | 8.031177 | 3.39E-14 | 2.93E-13 |
| AC078993.1 | 4.428326 | 8.798872 | 3.78E-14 | 3.24E-13 |
| AC011511.3 | 3.270094 | 4.454734 | 5.54E-14 | 4.72E-13 |
| AP003031.3 | 2.499894 | 3.790203 | 8.85E-14 | 7.40E-13 |
| LINC01833 | 4.174007 | 7.306176 | 1.29E-13 | 1.06E-12 |
| AC002546.1 | -2.04022 | 3.273589 | 1.29E-13 | 1.06E-12 |
| AC083809.1 | 2.89376 | 6.453411 | 1.43E-13 | 1.17E-12 |
| AC092484.1 | 4.394609 | 3.685504 | 1.45E-13 | 1.19E-12 |
| MIR378D2HG | 2.198933 | 3.722712 | 1.49E-13 | 1.22E-12 |
| AC104365.2 | 4.516227 | 4.866778 | 1.52E-13 | 1.24E-12 |
| AC011352.3 | 4.251135 | 3.874881 | 1.53E-13 | 1.24E-12 |
| LINC02195 | 2.703081 | 4.521736 | 1.74E-13 | 1.41E-12 |
| AC138904.1 | 2.085658 | 6.711542 | 1.83E-13 | 1.49E-12 |
| AC067930.4 | 2.03451 | 6.054431 | 2.15E-13 | 1.73E-12 |
| SCARNA9 | 2.530015 | 6.96586 | 2.18E-13 | 1.75E-12 |
| AC108134.3 | 2.28562 | 8.358965 | 2.27E-13 | 1.82E-12 |
| AC023421.1 | -2.61691 | 6.291125 | 2.55E-13 | 2.04E-12 |
| AC010789.1 | 4.603246 | 4.375352 | 2.58E-13 | 2.06E-12 |
| AC129926.1 | 4.087116 | 3.750001 | 2.96E-13 | 2.35E-12 |
| AC022784.6 | 2.469179 | 4.035169 | 3.55E-13 | 2.81E-12 |
| LINC00592 | 3.216234 | 3.388097 | 3.80E-13 | 3.00E-12 |
| LINC01269 | 2.745867 | 4.886312 | 4.45E-13 | 3.49E-12 |
| PWRN1 | -2.10426 | 3.375757 | 4.75E-13 | 3.72E-12 |
| AL359881.2 | 2.624862 | 4.247233 | 4.90E-13 | 3.83E-12 |
| AC093895.2 | 4.41286 | 4.467074 | 5.16E-13 | 4.02E-12 |
| AC113346.1 | 4.220742 | 3.813179 | 5.44E-13 | 4.22E-12 |
| LINC01942 | 2.546727 | 4.114407 | 5.57E-13 | 4.31E-12 |
| LINC02563 | 3.939222 | 5.11321 | 5.60E-13 | 4.33E-12 |
| AC136475.7 | 2.374233 | 4.771376 | 5.69E-13 | 4.39E-12 |
| LINC02254 | 3.447458 | 4.51479 | 5.85E-13 | 4.51E-12 |
| LINC01485 | 3.210226 | 6.120858 | 6.45E-13 | 4.95E-12 |
| LINC02544 | 3.56131 | 4.657667 | 7.08E-13 | 5.41E-12 |
| AC003101.2 | 2.218227 | 3.887857 | 7.36E-13 | 5.62E-12 |
| AC007608.2 | 5.433327 | 4.869528 | 7.85E-13 | 5.98E-12 |
| AC144450.1 | 3.424683 | 5.007363 | 9.91E-13 | 7.48E-12 |
| DLX6-AS1 | 4.051207 | 7.577918 | 1.01E-12 | 7.60E-12 |
| AC108751.4 | 2.165557 | 4.224038 | 1.06E-12 | 7.99E-12 |
| ABCA9-AS1 | 3.743358 | 4.899361 | 1.08E-12 | 8.09E-12 |
| AC010547.2 | 5.05845 | 5.179842 | 1.18E-12 | 8.85E-12 |
| AC093904.2 | 2.461274 | 4.593756 | 1.23E-12 | 9.17E-12 |
| AC009163.6 | -2.00533 | 4.588095 | 1.55E-12 | 1.15E-11 |
| LINC02657 | 2.543078 | 5.011569 | 1.62E-12 | 1.19E-11 |
| AC111149.2 | 4.755176 | 4.05582 | 1.67E-12 | 1.23E-11 |
| LINC00922 | 2.377445 | 4.282829 | 1.68E-12 | 1.24E-11 |
| Z97200.1 | 3.187089 | 4.485435 | 1.69E-12 | 1.24E-11 |
| AF127577.3 | 4.110761 | 5.565894 | 1.80E-12 | 1.32E-11 |
| AC092691.1 | -2.62862 | 3.164634 | 2.05E-12 | 1.50E-11 |
| WASIR2 | 2.541876 | 4.760978 | 2.40E-12 | 1.74E-11 |
| AP000696.1 | 3.856455 | 3.514128 | 2.41E-12 | 1.74E-11 |
| FRGCA | 2.620511 | 4.685225 | 2.60E-12 | 1.88E-11 |
| PCAT14 | 6.168034 | 8.681033 | 2.79E-12 | 2.01E-11 |
| AC018685.2 | 4.142462 | 3.551548 | 2.86E-12 | 2.06E-11 |
| LINC02428 | 4.758495 | 4.53557 | 3.00E-12 | 2.15E-11 |
| AC007405.3 | 3.487172 | 3.342823 | 3.02E-12 | 2.16E-11 |
| AL355112.1 | 2.847646 | 3.7956 | 3.31E-12 | 2.37E-11 |
| AC011498.3 | 2.463869 | 3.716897 | 3.33E-12 | 2.37E-11 |
| AL023803.3 | 2.150646 | 3.873157 | 3.34E-12 | 2.38E-11 |
| EIPR1-IT1 | 2.412099 | 3.964507 | 3.36E-12 | 2.39E-11 |
| AC093904.4 | 2.375987 | 6.288095 | 3.58E-12 | 2.55E-11 |
| INHBA-AS1 | 2.248567 | 4.519668 | 4.96E-12 | 3.47E-11 |
| MALAT1 | 2.396164 | 15.46939 | 5.56E-12 | 3.88E-11 |
| FGF13-AS1 | -2.08616 | 3.340243 | 9.81E-12 | 6.74E-11 |
| AC112721.2 | 2.954596 | 4.101864 | 1.02E-11 | 6.97E-11 |
| AC105460.1 | 8.054146 | 8.636204 | 1.07E-11 | 7.25E-11 |
| LINC02244 | 2.552229 | 4.105017 | 1.07E-11 | 7.25E-11 |
| MYO16-AS1 | 3.951805 | 4.111525 | 1.10E-11 | 7.50E-11 |
| AC090015.1 | 4.2486 | 4.067758 | 1.32E-11 | 8.92E-11 |
| AC008083.2 | 2.520602 | 4.193457 | 1.36E-11 | 9.18E-11 |
| CLMAT3 | 3.109459 | 5.14928 | 1.44E-11 | 9.67E-11 |
| STEAP2-AS1 | 2.321567 | 3.540731 | 1.84E-11 | 1.21E-10 |
| LINC01694 | 2.70219 | 7.491274 | 1.96E-11 | 1.29E-10 |
| LINC02263 | 3.937554 | 3.68546 | 2.05E-11 | 1.35E-10 |
| AC083967.1 | 3.672155 | 3.774343 | 2.54E-11 | 1.66E-10 |
| LINC02188 | 4.012888 | 5.127259 | 2.76E-11 | 1.79E-10 |
| AC118754.1 | 2.302518 | 4.094906 | 2.97E-11 | 1.92E-10 |
| TRHDE-AS1 | -2.1772 | 4.272417 | 2.98E-11 | 1.92E-10 |
| AP001434.1 | 3.793459 | 3.677971 | 3.19E-11 | 2.05E-10 |
| AL033543.1 | 2.694004 | 5.187594 | 3.21E-11 | 2.06E-10 |
| LINC01913 | 4.96738 | 4.749512 | 3.26E-11 | 2.09E-10 |
| PURPL | 4.490238 | 6.98902 | 3.44E-11 | 2.20E-10 |
| LINC02432 | 4.42634 | 7.99321 | 3.88E-11 | 2.47E-10 |
| AL022316.1 | 2.470185 | 5.933984 | 3.90E-11 | 2.48E-10 |
| AC110285.3 | 3.543303 | 4.788364 | 4.08E-11 | 2.59E-10 |
| AL807752.4 | 2.196716 | 3.754828 | 4.18E-11 | 2.64E-10 |
| AC011611.2 | 3.308633 | 4.041595 | 4.20E-11 | 2.65E-10 |
| LINC02043 | 2.201416 | 4.026271 | 4.34E-11 | 2.73E-10 |
| LINC01549 | 3.212505 | 4.581999 | 4.35E-11 | 2.73E-10 |
| AL121885.2 | 4.244615 | 3.974938 | 4.41E-11 | 2.77E-10 |
| AC007608.1 | 2.651544 | 6.382953 | 4.41E-11 | 2.77E-10 |
| ERVMER61-1 | 6.286447 | 5.719837 | 4.65E-11 | 2.91E-10 |
| LINC02029 | 2.329128 | 3.404158 | 4.93E-11 | 3.08E-10 |
| ARNTL2-AS1 | 3.135792 | 3.415858 | 5.29E-11 | 3.30E-10 |
| TMEM132D-AS1 | 7.980828 | 7.058909 | 5.42E-11 | 3.37E-10 |
| ST7-OT4 | 2.505964 | 4.813819 | 5.91E-11 | 3.66E-10 |
| AL121827.2 | 2.660785 | 6.621253 | 6.68E-11 | 4.11E-10 |
| AC007159.1 | 4.109051 | 3.652861 | 6.99E-11 | 4.29E-10 |
| SLC7A11-AS1 | 2.581743 | 5.497952 | 7.02E-11 | 4.31E-10 |
| LNCOG | 2.100448 | 4.018369 | 7.51E-11 | 4.61E-10 |
| LINC02178 | 5.568817 | 4.924272 | 7.68E-11 | 4.70E-10 |
| AC120498.4 | 2.797951 | 4.593748 | 8.22E-11 | 5.01E-10 |
| AC108112.1 | 3.651972 | 3.312571 | 8.36E-11 | 5.09E-10 |
| C17orf77 | 3.549332 | 8.200364 | 8.77E-11 | 5.33E-10 |
| AC133540.1 | 2.698073 | 4.818224 | 8.95E-11 | 5.43E-10 |
| AC134312.5 | 2.006459 | 5.782712 | 9.00E-11 | 5.46E-10 |
| LINC02560 | 2.656303 | 4.20541 | 9.61E-11 | 5.81E-10 |
| AC093520.1 | 2.329435 | 4.150157 | 9.77E-11 | 5.90E-10 |
| AL355312.3 | 2.514205 | 6.520274 | 1.16E-10 | 6.96E-10 |
| POU6F2-AS1 | 5.072844 | 4.670928 | 1.21E-10 | 7.20E-10 |
| AC011352.1 | 3.487261 | 3.353937 | 1.23E-10 | 7.32E-10 |
| AC125603.2 | 3.053614 | 6.258907 | 1.29E-10 | 7.67E-10 |
| AL591222.1 | 3.520557 | 3.856242 | 1.30E-10 | 7.73E-10 |
| AL162727.1 | 2.002001 | 3.809693 | 1.36E-10 | 8.08E-10 |
| AL365226.1 | 3.51695 | 10.54289 | 1.48E-10 | 8.74E-10 |
| DPP10-AS1 | -2.38796 | 6.978442 | 1.49E-10 | 8.78E-10 |
| AC036176.3 | 3.208934 | 3.756621 | 1.50E-10 | 8.83E-10 |
| AC139720.2 | 3.842773 | 3.980044 | 1.55E-10 | 9.10E-10 |
| AL136307.1 | 2.320582 | 4.321273 | 1.74E-10 | 1.02E-09 |
| AL136418.3 | 2.260008 | 6.184912 | 1.88E-10 | 1.09E-09 |
| LINC00658 | 3.135398 | 4.414867 | 1.90E-10 | 1.10E-09 |
| C7orf69 | 2.662658 | 3.20385 | 1.98E-10 | 1.15E-09 |
| AC005363.2 | 2.113593 | 3.322442 | 2.01E-10 | 1.16E-09 |
| LINC01210 | 3.892435 | 3.436907 | 2.31E-10 | 1.32E-09 |
| DSG1-AS1 | 2.381313 | 4.075196 | 2.61E-10 | 1.49E-09 |
| AC104809.2 | 3.382767 | 6.329171 | 2.64E-10 | 1.50E-09 |
| LINC00524 | 3.432018 | 4.128747 | 2.65E-10 | 1.50E-09 |
| EGOT | 2.125559 | 4.508142 | 2.74E-10 | 1.55E-09 |
| AC116345.1 | 3.386962 | 6.943003 | 2.74E-10 | 1.55E-09 |
| LINC02156 | 2.134577 | 3.611802 | 2.82E-10 | 1.59E-09 |
| LINC01419 | 5.963435 | 4.795716 | 2.99E-10 | 1.69E-09 |
| LINC02119 | 4.844608 | 4.814875 | 3.14E-10 | 1.76E-09 |
| AC008456.1 | 2.569918 | 4.324132 | 3.14E-10 | 1.76E-09 |
| FSIP2-AS2 | 2.10502 | 5.191992 | 3.30E-10 | 1.85E-09 |
| AP000525.1 | 2.893361 | 4.696958 | 3.50E-10 | 1.95E-09 |
| AC011483.1 | 3.549408 | 3.37099 | 3.55E-10 | 1.98E-09 |
| C15orf54 | 2.710833 | 3.941818 | 3.70E-10 | 2.06E-09 |
| AC092198.1 | 2.471224 | 4.952942 | 3.75E-10 | 2.08E-09 |
| AC127024.2 | 2.04563 | 4.478643 | 4.24E-10 | 2.34E-09 |
| AC010998.3 | -2.09003 | 5.283258 | 4.32E-10 | 2.38E-09 |
| LINC01146 | 2.253556 | 5.80132 | 4.52E-10 | 2.49E-09 |
| KIF26B-AS1 | 3.863027 | 3.770216 | 5.32E-10 | 2.90E-09 |
| AC112721.1 | 2.499365 | 3.721101 | 5.75E-10 | 3.12E-09 |
| AC092118.1 | 2.797796 | 3.392925 | 6.17E-10 | 3.32E-09 |
| AC114321.1 | 3.354753 | 3.191147 | 6.74E-10 | 3.61E-09 |
| AC016745.1 | 2.714062 | 3.334462 | 7.18E-10 | 3.84E-09 |
| LINC01807 | 3.465887 | 5.674286 | 7.19E-10 | 3.84E-09 |
| LINC01429 | 2.898365 | 3.729818 | 7.21E-10 | 3.85E-09 |
| BX322234.2 | 7.834889 | 6.431353 | 7.76E-10 | 4.13E-09 |
| BX640514.2 | 2.150136 | 4.761877 | 7.79E-10 | 4.14E-09 |
| AC004231.1 | 2.6552 | 5.354875 | 7.86E-10 | 4.18E-09 |
| SLC5A4-AS1 | 2.261059 | 4.247021 | 8.42E-10 | 4.46E-09 |
| IGF2-AS | 3.659175 | 5.556899 | 8.91E-10 | 4.71E-09 |
| AC009630.2 | 2.248969 | 3.182039 | 9.12E-10 | 4.82E-09 |
| AC104964.1 | 2.19953 | 6.287025 | 9.26E-10 | 4.88E-09 |
| CLLU1 | 3.450747 | 4.357837 | 9.68E-10 | 5.10E-09 |
| LINC02027 | 3.917853 | 3.669947 | 1.00E-09 | 5.26E-09 |
| AC090809.1 | 5.029819 | 4.237103 | 1.31E-09 | 6.79E-09 |
| SMILR | 3.155243 | 3.612604 | 1.46E-09 | 7.52E-09 |
| MRPL23-AS1 | 3.34682 | 5.248584 | 1.66E-09 | 8.55E-09 |
| LINC02582 | 6.670851 | 6.272153 | 1.71E-09 | 8.77E-09 |
| DIAPH2-AS1 | 2.269719 | 5.349715 | 1.88E-09 | 9.58E-09 |
| BMP7-AS1 | 3.079965 | 3.081406 | 1.90E-09 | 9.67E-09 |
| AC012494.1 | 3.339376 | 3.349934 | 2.10E-09 | 1.06E-08 |
| AL136114.1 | 3.052901 | 3.310989 | 2.26E-09 | 1.14E-08 |
| AC010595.1 | 4.99701 | 4.244272 | 2.27E-09 | 1.14E-08 |
| AC083906.3 | 2.430252 | 3.365844 | 2.31E-09 | 1.16E-08 |
| LINC01819 | 3.595797 | 10.05826 | 2.32E-09 | 1.16E-08 |
| AC108136.1 | 3.126203 | 3.774165 | 2.32E-09 | 1.16E-08 |
| NEUROG2-AS1 | 4.027064 | 4.53742 | 2.54E-09 | 1.27E-08 |
| AC009262.1 | 2.6064 | 3.289808 | 2.62E-09 | 1.31E-08 |
| AL008723.2 | 2.454686 | 3.950995 | 2.64E-09 | 1.31E-08 |
| CAMTA1-IT1 | 2.56383 | 3.329184 | 2.65E-09 | 1.32E-08 |
| CASC11 | 2.130947 | 5.467349 | 2.80E-09 | 1.39E-08 |
| AL033381.2 | 2.769822 | 3.708032 | 2.96E-09 | 1.46E-08 |
| AC020907.1 | 3.187606 | 5.100589 | 2.97E-09 | 1.46E-08 |
| TESC-AS1 | 2.798833 | 3.402983 | 3.15E-09 | 1.55E-08 |
| AL645608.2 | 2.515826 | 4.833311 | 3.19E-09 | 1.57E-08 |
| AP001429.1 | 2.40232 | 4.774241 | 3.34E-09 | 1.64E-08 |
| LINC02154 | 2.197129 | 4.767395 | 3.58E-09 | 1.75E-08 |
| LINC01060 | 2.802232 | 3.648738 | 3.60E-09 | 1.76E-08 |
| AC073257.2 | 2.256208 | 3.903735 | 3.61E-09 | 1.76E-08 |
| AC092112.1 | 3.626619 | 5.430207 | 3.67E-09 | 1.79E-08 |
| LINC01267 | 2.577132 | 4.205408 | 3.94E-09 | 1.91E-08 |
| LINC00355 | 5.253056 | 4.798627 | 4.24E-09 | 2.05E-08 |
| AC137894.1 | 2.213299 | 4.386259 | 4.47E-09 | 2.15E-08 |
| AL033397.1 | 3.431026 | 5.983126 | 4.83E-09 | 2.32E-08 |
| AL031275.1 | 2.324058 | 4.933441 | 4.89E-09 | 2.35E-08 |
| AC211433.1 | 2.077935 | 5.332073 | 4.97E-09 | 2.38E-08 |
| AC008551.1 | 2.926153 | 4.271231 | 5.57E-09 | 2.66E-08 |
| AL513324.1 | 2.704249 | 3.17433 | 5.60E-09 | 2.67E-08 |
| AL355075.4 | 6.659088 | 7.583482 | 5.73E-09 | 2.73E-08 |
| AC016831.4 | 2.398959 | 6.195779 | 5.75E-09 | 2.74E-08 |
| AC092168.2 | 2.433427 | 4.778041 | 5.80E-09 | 2.76E-08 |
| NKX2-1-AS1 | 5.394169 | 4.342752 | 5.81E-09 | 2.76E-08 |
| LINC02832 | 2.454269 | 3.378669 | 6.18E-09 | 2.93E-08 |
| AC006262.2 | 3.258259 | 3.324906 | 7.15E-09 | 3.37E-08 |
| DLGAP1-AS5 | 5.282548 | 6.8932 | 7.78E-09 | 3.66E-08 |
| AC068491.3 | 2.784335 | 3.261026 | 8.47E-09 | 3.97E-08 |
| LINC02783 | 3.739995 | 3.65385 | 8.85E-09 | 4.15E-08 |
| LINC01971 | 2.73657 | 3.512634 | 9.66E-09 | 4.50E-08 |
| AC018553.1 | 3.100059 | 5.193292 | 1.02E-08 | 4.73E-08 |
| MYRF-AS1 | 2.003627 | 4.940329 | 1.11E-08 | 5.12E-08 |
| AC005096.1 | 2.249351 | 3.858386 | 1.13E-08 | 5.23E-08 |
| LINC02404 | 4.478113 | 3.991795 | 1.14E-08 | 5.28E-08 |
| LINC00545 | 2.878613 | 3.907554 | 1.31E-08 | 6.00E-08 |
| AC136475.9 | 2.286119 | 4.804866 | 1.43E-08 | 6.51E-08 |
| AC148477.4 | 2.765134 | 5.097335 | 1.50E-08 | 6.82E-08 |
| AC020891.3 | 2.157255 | 3.174748 | 1.64E-08 | 7.45E-08 |
| Z99289.2 | 2.44777 | 3.857806 | 1.65E-08 | 7.47E-08 |
| IGFBP7-AS1 | 2.63106 | 5.727776 | 1.91E-08 | 8.56E-08 |
| AL117329.1 | 4.598532 | 3.981845 | 1.94E-08 | 8.70E-08 |
| AC107308.1 | 2.197914 | 3.542931 | 2.04E-08 | 9.15E-08 |
| AL138760.1 | 2.186313 | 3.733764 | 2.20E-08 | 9.80E-08 |
| LINC01630 | 3.857333 | 4.572262 | 2.27E-08 | 1.01E-07 |
| AL138789.1 | 2.25773 | 5.256471 | 2.48E-08 | 1.10E-07 |
| MAGEA4-AS1 | 5.134936 | 4.155028 | 2.63E-08 | 1.16E-07 |
| AL591806.1 | 2.174966 | 3.488233 | 2.69E-08 | 1.18E-07 |
| AP000785.1 | 2.484018 | 5.120757 | 2.73E-08 | 1.20E-07 |
| AC007009.1 | 4.00998 | 3.516598 | 2.85E-08 | 1.25E-07 |
| AL592043.1 | 3.570177 | 3.671482 | 3.22E-08 | 1.41E-07 |
| AL499627.1 | 3.531306 | 3.37161 | 3.32E-08 | 1.45E-07 |
| AC105118.1 | 2.71519 | 3.243746 | 3.79E-08 | 1.65E-07 |
| AC010894.3 | 3.300086 | 5.967359 | 3.83E-08 | 1.67E-07 |
| AC105460.2 | 4.47911 | 4.133582 | 3.88E-08 | 1.69E-07 |
| AC091868.2 | 2.670079 | 3.150231 | 4.10E-08 | 1.78E-07 |
| AL352984.1 | 3.112026 | 3.355015 | 4.11E-08 | 1.78E-07 |
| AL391421.1 | 2.521818 | 4.125204 | 4.14E-08 | 1.80E-07 |
| AC125603.1 | 3.388783 | 4.483632 | 4.32E-08 | 1.87E-07 |
| MAGEA10-MAGEA5 | 2.135869 | 3.291778 | 4.35E-08 | 1.88E-07 |
| AL355483.2 | 3.296825 | 3.357159 | 4.41E-08 | 1.91E-07 |
| TBL1XR1-AS1 | 2.842343 | 4.022588 | 4.42E-08 | 1.91E-07 |
| AL445183.2 | 2.017723 | 4.612115 | 4.52E-08 | 1.95E-07 |
| MIR2052HG | 3.242839 | 3.941665 | 4.53E-08 | 1.95E-07 |
| AC021534.1 | 4.095272 | 3.615838 | 6.02E-08 | 2.56E-07 |
| LINC00958 | 2.432507 | 5.464284 | 6.36E-08 | 2.69E-07 |
| AC012494.2 | 3.334325 | 3.302926 | 6.59E-08 | 2.79E-07 |
| AC009055.2 | 4.274869 | 3.646603 | 6.70E-08 | 2.82E-07 |
| RBAKDN | 2.472739 | 3.602371 | 6.81E-08 | 2.87E-07 |
| Z82217.1 | 2.323463 | 5.314222 | 7.05E-08 | 2.96E-07 |
| FAM230G | 2.870041 | 3.172582 | 7.24E-08 | 3.03E-07 |
| AC108676.1 | 2.181175 | 6.405704 | 7.56E-08 | 3.15E-07 |
| AC093425.1 | 2.727725 | 5.622546 | 7.69E-08 | 3.21E-07 |
| AC024581.1 | 2.130206 | 3.282004 | 7.94E-08 | 3.30E-07 |
| AL645608.8 | 2.199168 | 3.628319 | 9.07E-08 | 3.74E-07 |
| AC036222.3 | 2.726403 | 3.238216 | 9.23E-08 | 3.80E-07 |
| AC011754.1 | 5.136897 | 5.054774 | 9.25E-08 | 3.80E-07 |
| AC087857.1 | 2.936893 | 3.489171 | 9.34E-08 | 3.84E-07 |
| LINC00973 | 4.077022 | 4.305563 | 9.38E-08 | 3.85E-07 |
| AL160408.1 | 2.553741 | 3.470298 | 9.83E-08 | 4.02E-07 |
| LINC02700 | 3.429906 | 3.512915 | 1.00E-07 | 4.09E-07 |
| RMRP | 6.557317 | 6.593075 | 1.11E-07 | 4.52E-07 |
| AL161729.2 | 2.10486 | 4.086572 | 1.32E-07 | 5.30E-07 |
| MIR31HG | 3.128491 | 5.297344 | 1.38E-07 | 5.52E-07 |
| AC116049.1 | 2.309594 | 4.122258 | 1.42E-07 | 5.68E-07 |
| AC106875.1 | 3.575217 | 3.568267 | 1.42E-07 | 5.68E-07 |
| LINC01633 | 2.70974 | 3.167615 | 1.49E-07 | 5.92E-07 |
| AL136115.2 | 2.042778 | 4.566016 | 1.59E-07 | 6.31E-07 |
| LINC01992 | 4.039917 | 3.879749 | 1.69E-07 | 6.69E-07 |
| AL080312.2 | 2.045338 | 3.537853 | 1.70E-07 | 6.74E-07 |
| AL121761.1 | 2.073647 | 7.47591 | 1.71E-07 | 6.76E-07 |
| LINC01749 | 2.555894 | 4.112627 | 1.80E-07 | 7.09E-07 |
| AL445223.1 | 4.053913 | 3.777782 | 1.80E-07 | 7.10E-07 |
| AL008721.1 | 2.098628 | 3.73261 | 1.84E-07 | 7.25E-07 |
| DDC-AS1 | 2.097024 | 3.935255 | 1.95E-07 | 7.66E-07 |
| LINC02533 | 2.352531 | 3.254513 | 2.01E-07 | 7.87E-07 |
| AC141930.1 | 2.226355 | 4.788496 | 2.01E-07 | 7.87E-07 |
| ST8SIA6-AS1 | 3.345642 | 6.456089 | 2.12E-07 | 8.29E-07 |
| Z97192.1 | 2.285749 | 4.371378 | 2.16E-07 | 8.41E-07 |
| AC008514.1 | 2.852435 | 5.206377 | 2.21E-07 | 8.62E-07 |
| AL353747.3 | 2.230843 | 4.768787 | 2.50E-07 | 9.67E-07 |
| AC084262.1 | 2.270648 | 3.751488 | 2.52E-07 | 9.75E-07 |
| AL138962.1 | 2.626779 | 3.268752 | 2.63E-07 | 1.01E-06 |
| AL122058.1 | 2.134591 | 4.36517 | 2.73E-07 | 1.05E-06 |
| LINC00626 | 3.268725 | 3.434619 | 2.80E-07 | 1.08E-06 |
| AC109439.1 | 4.625123 | 3.840454 | 2.83E-07 | 1.09E-06 |
| AL606537.1 | 2.13374 | 3.934729 | 2.87E-07 | 1.10E-06 |
| BOK-AS1 | 4.086751 | 6.426364 | 2.89E-07 | 1.11E-06 |
| AC108451.2 | 3.448428 | 3.48928 | 2.90E-07 | 1.11E-06 |
| AC074124.1 | 3.073736 | 4.11119 | 2.93E-07 | 1.12E-06 |
| FOXC2-AS1 | 3.346606 | 3.499996 | 3.00E-07 | 1.14E-06 |
| AC127024.3 | 2.240499 | 3.939164 | 3.24E-07 | 1.23E-06 |
| LINC02525 | 3.094052 | 4.581019 | 3.25E-07 | 1.23E-06 |
| AL138733.1 | 2.074356 | 3.23644 | 3.54E-07 | 1.34E-06 |
| AL031665.1 | 2.342566 | 4.142028 | 3.61E-07 | 1.36E-06 |
| AL035252.3 | 2.939906 | 4.383597 | 3.64E-07 | 1.37E-06 |
| LINC02395 | 2.338059 | 3.325783 | 3.70E-07 | 1.39E-06 |
| AC096746.1 | 2.804492 | 3.231048 | 3.84E-07 | 1.44E-06 |
| LINC02475 | 3.257534 | 4.629283 | 3.84E-07 | 1.44E-06 |
| AL807761.3 | 3.259316 | 3.982883 | 3.97E-07 | 1.49E-06 |
| ESRG | 3.680705 | 4.106438 | 4.01E-07 | 1.50E-06 |
| LINC02419 | 2.508761 | 3.318398 | 4.17E-07 | 1.56E-06 |
| AC005307.1 | 2.832262 | 6.166411 | 4.34E-07 | 1.62E-06 |
| AC091153.2 | 2.335235 | 3.140215 | 4.39E-07 | 1.64E-06 |
| DBET | 2.541559 | 4.918974 | 4.45E-07 | 1.66E-06 |
| FP325330.3 | 3.272461 | 3.34216 | 4.51E-07 | 1.68E-06 |
| LHX1-DT | 3.177741 | 3.198696 | 4.72E-07 | 1.75E-06 |
| WT1-AS | 2.879356 | 4.652614 | 5.29E-07 | 1.95E-06 |
| AC073651.1 | 3.575732 | 3.951677 | 5.30E-07 | 1.95E-06 |
| AC011389.2 | 3.26408 | 3.618976 | 5.77E-07 | 2.12E-06 |
| AC247036.1 | 5.297295 | 4.865168 | 5.86E-07 | 2.15E-06 |
| FP671120.7 | 5.608666 | 5.73513 | 5.92E-07 | 2.17E-06 |
| LINC01219 | 2.641834 | 3.200721 | 5.93E-07 | 2.17E-06 |
| AC009646.2 | 3.873595 | 3.674829 | 5.97E-07 | 2.18E-06 |
| LINC01980 | 4.547046 | 4.950116 | 6.88E-07 | 2.50E-06 |
| AC026310.2 | 2.540192 | 3.472466 | 7.13E-07 | 2.59E-06 |
| COL4A2-AS2 | 2.123047 | 3.612407 | 7.33E-07 | 2.65E-06 |
| AC011287.1 | 2.883462 | 3.531647 | 7.52E-07 | 2.72E-06 |
| AC100823.1 | 2.461574 | 4.198376 | 7.57E-07 | 2.73E-06 |
| AC073326.1 | 2.000601 | 3.582036 | 7.84E-07 | 2.82E-06 |
| AL611929.1 | 2.956894 | 3.485026 | 7.90E-07 | 2.84E-06 |
| AL096677.1 | 2.330137 | 3.415759 | 8.11E-07 | 2.91E-06 |
| AC096759.2 | 3.487576 | 3.411327 | 8.37E-07 | 3.00E-06 |
| SND1-IT1 | 3.244805 | 4.694465 | 8.48E-07 | 3.04E-06 |
| AC005291.1 | 2.056434 | 3.324856 | 8.87E-07 | 3.17E-06 |
| LINC02864 | 4.555182 | 6.670835 | 9.03E-07 | 3.22E-06 |
| LINC01414 | 3.340144 | 3.546251 | 9.55E-07 | 3.39E-06 |
| AC092718.6 | 2.074259 | 3.723451 | 9.84E-07 | 3.49E-06 |
| ASTN2-AS1 | 3.250974 | 3.276458 | 9.96E-07 | 3.53E-06 |
| MACC1-AS1 | 2.936592 | 4.114015 | 1.00E-06 | 3.54E-06 |
| AC097478.1 | 3.087928 | 3.528163 | 1.01E-06 | 3.56E-06 |
| AC018552.3 | 2.244134 | 3.599435 | 1.02E-06 | 3.61E-06 |
| AC092127.1 | 2.50373 | 4.436925 | 1.03E-06 | 3.63E-06 |
| AL139147.1 | 2.492818 | 3.920584 | 1.03E-06 | 3.64E-06 |
| DSCR8 | 5.054984 | 4.694601 | 1.06E-06 | 3.74E-06 |
| LINC01840 | 2.000051 | 4.809944 | 1.09E-06 | 3.83E-06 |
| LINC02042 | 4.00404 | 4.115671 | 1.10E-06 | 3.86E-06 |
| ATP11A-AS1 | 3.101696 | 3.966742 | 1.11E-06 | 3.88E-06 |
| LINC02466 | 3.230919 | 3.15053 | 1.12E-06 | 3.92E-06 |
| LINC00885 | 2.121258 | 3.962954 | 1.12E-06 | 3.94E-06 |
| AC013457.1 | 3.271723 | 3.486182 | 1.13E-06 | 3.96E-06 |
| LINC01967 | 2.724351 | 3.912097 | 1.39E-06 | 4.82E-06 |
| HOTAIR | 3.223608 | 6.307793 | 1.50E-06 | 5.17E-06 |
| AC004704.1 | 2.502035 | 3.211652 | 1.57E-06 | 5.40E-06 |
| AC084375.1 | 2.4632 | 4.309534 | 1.73E-06 | 5.91E-06 |
| AC011676.2 | 2.411376 | 3.510916 | 1.76E-06 | 6.00E-06 |
| AC104461.1 | 2.064017 | 3.209453 | 1.77E-06 | 6.04E-06 |
| LINC01170 | 2.637756 | 3.699399 | 1.79E-06 | 6.10E-06 |
| AL139023.1 | 3.82084 | 3.519232 | 1.87E-06 | 6.35E-06 |
| ERVH48-1 | 2.274791 | 5.859175 | 1.92E-06 | 6.48E-06 |
| AIRN | 3.115477 | 3.822579 | 2.03E-06 | 6.83E-06 |
| AL356488.2 | 2.718183 | 3.585667 | 2.04E-06 | 6.87E-06 |
| DSCAM-AS1 | 4.336198 | 3.76632 | 2.05E-06 | 6.88E-06 |
| AC099811.1 | 2.032466 | 4.332932 | 2.14E-06 | 7.19E-06 |
| AL021328.1 | 2.551229 | 3.983107 | 2.21E-06 | 7.38E-06 |
| AC007785.1 | 2.330826 | 3.794002 | 2.33E-06 | 7.75E-06 |
| LINC02433 | 3.103935 | 3.126702 | 2.44E-06 | 8.08E-06 |
| AL035078.1 | 2.353679 | 3.34622 | 2.59E-06 | 8.55E-06 |
| LNCAROD | 4.203848 | 4.782526 | 2.60E-06 | 8.59E-06 |
| LINC02476 | 4.284212 | 3.652226 | 2.66E-06 | 8.76E-06 |
| AL589743.5 | 2.70951 | 3.323155 | 2.74E-06 | 9.02E-06 |
| GRM8-AS1 | 3.635028 | 3.576275 | 2.79E-06 | 9.15E-06 |
| AC132807.2 | 2.913114 | 4.722351 | 2.94E-06 | 9.61E-06 |
| AC011933.2 | 2.869595 | 4.593485 | 3.26E-06 | 1.06E-05 |
| LINC02672 | 3.640891 | 4.518944 | 3.37E-06 | 1.09E-05 |
| TERC | 2.373764 | 3.872981 | 3.38E-06 | 1.09E-05 |
| MIR205HG | 5.205688 | 5.691552 | 3.53E-06 | 1.14E-05 |
| AC145146.1 | 2.426597 | 3.127294 | 3.53E-06 | 1.14E-05 |
| MIR181A1HG | 2.779056 | 4.015929 | 3.83E-06 | 1.23E-05 |
| Z93403.1 | 2.437634 | 3.656559 | 4.14E-06 | 1.32E-05 |
| AL354863.1 | 3.275424 | 3.800123 | 4.20E-06 | 1.34E-05 |
| LINC02182 | 2.087289 | 3.599569 | 4.36E-06 | 1.39E-05 |
| AC073957.1 | 2.146687 | 3.248047 | 4.61E-06 | 1.46E-05 |
| AC008649.2 | 2.182573 | 3.960225 | 4.65E-06 | 1.47E-05 |
| AC006967.3 | 2.683713 | 4.467581 | 4.66E-06 | 1.48E-05 |
| LINC01641 | 2.204536 | 3.850554 | 4.70E-06 | 1.49E-05 |
| MRTFA-AS1 | 2.129184 | 4.738595 | 4.92E-06 | 1.55E-05 |
| AC004223.2 | 2.095385 | 3.802304 | 4.92E-06 | 1.55E-05 |
| LINC01611 | 2.847195 | 3.169723 | 5.05E-06 | 1.59E-05 |
| AC107419.1 | 2.983663 | 3.663921 | 5.08E-06 | 1.59E-05 |
| LINC02575 | 2.43131 | 5.926971 | 5.14E-06 | 1.61E-05 |
| AP002993.1 | 2.314982 | 3.075531 | 5.17E-06 | 1.62E-05 |
| LIF-AS1 | 2.007194 | 3.202849 | 5.24E-06 | 1.64E-05 |
| AP005262.1 | 3.707818 | 3.673973 | 5.58E-06 | 1.74E-05 |
| EIF1AX-AS1 | 2.208922 | 3.826232 | 5.73E-06 | 1.79E-05 |
| ST7-AS2 | 3.200597 | 4.222189 | 5.82E-06 | 1.81E-05 |
| AC069120.1 | 2.332607 | 4.196383 | 6.19E-06 | 1.92E-05 |
| OSBPL10-AS1 | 2.332647 | 3.840085 | 6.26E-06 | 1.94E-05 |
| AC010983.1 | 2.963808 | 3.157718 | 6.53E-06 | 2.01E-05 |
| AC016831.5 | 2.812803 | 3.395371 | 6.63E-06 | 2.04E-05 |
| G2E3-AS1 | 3.565218 | 4.066801 | 6.72E-06 | 2.07E-05 |
| C8orf49 | 3.256601 | 3.807161 | 6.79E-06 | 2.09E-05 |
| LINC00393 | 3.041957 | 4.898118 | 7.74E-06 | 2.36E-05 |
| AL442224.1 | 3.114135 | 3.516479 | 8.14E-06 | 2.47E-05 |
| PHACTR2-AS1 | 2.557873 | 4.420003 | 8.30E-06 | 2.52E-05 |
| LPP-AS1 | 3.709984 | 3.420207 | 8.40E-06 | 2.55E-05 |
| AC011595.1 | 3.031134 | 3.268776 | 8.82E-06 | 2.66E-05 |
| MPRIP-AS1 | 2.0828 | 3.758027 | 9.18E-06 | 2.76E-05 |
| AL133284.1 | 2.416051 | 3.842901 | 9.25E-06 | 2.78E-05 |
| AC004009.1 | 2.805045 | 4.500268 | 9.31E-06 | 2.79E-05 |
| AL110505.1 | 3.174076 | 3.39441 | 9.31E-06 | 2.79E-05 |
| AL390961.1 | 3.455195 | 3.355891 | 9.81E-06 | 2.93E-05 |
| PINCR | 2.982015 | 3.265582 | 1.02E-05 | 3.05E-05 |
| LINC00616 | 3.108385 | 3.803885 | 1.11E-05 | 3.31E-05 |
| AC090220.1 | 3.741391 | 3.986234 | 1.12E-05 | 3.34E-05 |
| AC040904.1 | 2.108267 | 3.248356 | 1.13E-05 | 3.36E-05 |
| AC110772.2 | 2.733327 | 5.929247 | 1.24E-05 | 3.66E-05 |
| FAM41C | 2.625175 | 3.928464 | 1.27E-05 | 3.75E-05 |
| BARX1-DT | 2.813413 | 3.531019 | 1.39E-05 | 4.09E-05 |
| LINC02617 | 2.76843 | 3.426436 | 1.44E-05 | 4.23E-05 |
| AC005072.1 | 2.155515 | 4.147445 | 1.46E-05 | 4.28E-05 |
| AC079915.1 | 2.535756 | 4.368829 | 1.56E-05 | 4.52E-05 |
| LINC02735 | 2.563025 | 3.518775 | 1.56E-05 | 4.54E-05 |
| AL020995.1 | 2.286207 | 3.717831 | 1.63E-05 | 4.71E-05 |
| CFTR-AS1 | 2.366913 | 3.556261 | 1.76E-05 | 5.09E-05 |
| CNOT10-AS1 | 2.360232 | 3.100289 | 2.07E-05 | 5.91E-05 |
| AL008636.1 | 2.357123 | 3.615896 | 2.10E-05 | 5.96E-05 |
| Z93022.1 | 2.760815 | 3.478027 | 2.14E-05 | 6.08E-05 |
| LINP1 | 3.31526 | 3.330604 | 2.22E-05 | 6.28E-05 |
| FP236383.5 | 3.878608 | 3.842829 | 2.23E-05 | 6.30E-05 |
| HOXC-AS2 | 2.172221 | 4.673029 | 2.31E-05 | 6.53E-05 |
| C8orf34-AS1 | 2.988373 | 4.450083 | 2.43E-05 | 6.83E-05 |
| AC091151.1 | 2.583 | 3.701369 | 2.47E-05 | 6.94E-05 |
| AL133260.2 | 2.385872 | 3.116691 | 2.57E-05 | 7.21E-05 |
| NFIA-AS1 | 2.946101 | 3.73754 | 2.65E-05 | 7.42E-05 |
| AC008517.1 | 3.582446 | 3.266233 | 2.70E-05 | 7.54E-05 |
| LINC00400 | 3.975479 | 3.412043 | 2.82E-05 | 7.84E-05 |
| LINC01346 | 2.142902 | 3.480543 | 2.88E-05 | 7.99E-05 |
| AL365356.1 | 2.131972 | 4.279694 | 3.21E-05 | 8.85E-05 |
| CYP4F26P | 2.300268 | 4.086865 | 3.25E-05 | 8.94E-05 |
| AC016705.1 | 2.171174 | 3.233187 | 3.29E-05 | 9.03E-05 |
| AC022537.1 | 3.283857 | 3.320421 | 3.65E-05 | 9.94E-05 |
| LINC01709 | 2.737192 | 3.386879 | 3.66E-05 | 9.96E-05 |
| AC026333.3 | 2.758528 | 3.465152 | 3.71E-05 | 0.0001009 |
| C5orf66-AS2 | 2.485239 | 3.102574 | 3.80E-05 | 0.0001031 |
| AC092801.1 | 2.967909 | 3.846343 | 3.88E-05 | 0.000105 |
| RASA2-IT1 | 2.914065 | 3.432338 | 3.92E-05 | 0.000106 |
| AC020661.4 | 2.551125 | 3.409049 | 4.13E-05 | 0.0001113 |
| AC093817.1 | 2.617704 | 3.030813 | 4.13E-05 | 0.0001113 |
| AC012355.1 | 3.34362 | 3.176725 | 4.16E-05 | 0.0001121 |
| CACNA1C-AS4 | 3.172415 | 3.509087 | 4.17E-05 | 0.0001122 |
| AC005725.1 | 2.281128 | 6.176372 | 4.27E-05 | 0.0001148 |
| SMCR5 | 2.058261 | 3.972978 | 4.74E-05 | 0.0001263 |
| CELF2-AS2 | 3.424092 | 3.590922 | 4.91E-05 | 0.0001304 |
| AC021237.1 | 2.842899 | 3.412082 | 5.27E-05 | 0.0001391 |
| AC100774.1 | 2.04415 | 3.821996 | 5.33E-05 | 0.0001405 |
| LINC01940 | 2.955438 | 4.249801 | 5.40E-05 | 0.0001421 |
| HECW1-IT1 | 3.110402 | 3.162905 | 5.58E-05 | 0.0001465 |
| AC007431.2 | 2.899768 | 3.158603 | 5.73E-05 | 0.0001501 |
| AP001442.1 | 2.109761 | 4.730855 | 6.14E-05 | 0.0001604 |
| AC110772.1 | 2.398036 | 4.637086 | 6.29E-05 | 0.0001639 |
| LINC01896 | 3.080691 | 3.186254 | 6.36E-05 | 0.0001655 |
| AL034349.1 | 3.063758 | 3.747647 | 6.41E-05 | 0.0001669 |
| SOX21-AS1 | 2.724138 | 4.266274 | 6.50E-05 | 0.0001688 |
| AL390774.2 | 2.636098 | 3.570199 | 6.89E-05 | 0.0001786 |
| AC106799.3 | 2.954015 | 3.496794 | 7.04E-05 | 0.0001818 |
| AC106873.1 | 2.646096 | 3.373515 | 7.11E-05 | 0.0001833 |
| AC087893.3 | 2.352636 | 3.679407 | 7.30E-05 | 0.0001879 |
| AC100782.1 | 2.201118 | 2.99537 | 7.35E-05 | 0.0001891 |
| AL589863.1 | 2.875135 | 3.147118 | 7.66E-05 | 0.0001966 |
| LINC02675 | 2.012408 | 3.818758 | 7.69E-05 | 0.0001973 |
| AC112236.1 | 2.082536 | 3.195336 | 7.98E-05 | 0.0002043 |
| BTBD9-AS1 | 2.590517 | 3.526939 | 8.02E-05 | 0.0002052 |
| AL157402.2 | 3.308169 | 3.493096 | 8.33E-05 | 0.0002128 |
| AC100763.1 | 3.279733 | 3.505526 | 8.34E-05 | 0.0002129 |
| MIR548XHG | 3.182102 | 3.340387 | 8.41E-05 | 0.0002146 |
| AL359851.1 | 2.781087 | 3.376773 | 8.91E-05 | 0.000226 |
| AP006259.1 | 2.737639 | 3.223732 | 8.92E-05 | 0.0002261 |
| SPATA13-AS1 | 2.682701 | 3.569008 | 9.05E-05 | 0.0002291 |
| FAM230C | 3.071083 | 3.105683 | 9.27E-05 | 0.0002341 |
| ITCH-IT1 | 2.275993 | 3.415991 | 9.28E-05 | 0.0002343 |
| PRKCA-AS1 | 2.381008 | 4.232243 | 9.43E-05 | 0.000238 |
| AC012531.1 | 2.21576 | 4.457759 | 9.45E-05 | 0.0002383 |
| NHS-AS1 | 2.606892 | 2.970125 | 9.51E-05 | 0.0002396 |
| AC097381.2 | 2.094862 | 3.065381 | 9.84E-05 | 0.0002471 |
| AL390778.2 | 2.332677 | 4.528647 | 9.86E-05 | 0.0002476 |
| AC092969.1 | 2.311341 | 3.932909 | 0.0001027 | 0.000257 |
| AC144521.1 | 2.664834 | 3.078367 | 0.0001072 | 0.000267 |
| LINC01087 | 2.326318 | 3.237956 | 0.0001099 | 0.000273 |
| LINC01320 | 2.052926 | 3.518978 | 0.0001117 | 0.0002771 |
| AC009054.1 | 2.184026 | 3.149843 | 0.0001118 | 0.0002773 |
| AC008277.1 | 2.531884 | 4.077717 | 0.0001199 | 0.0002959 |
| AL731577.1 | 2.484574 | 3.568521 | 0.0001231 | 0.0003035 |
| LINC02303 | 2.671536 | 3.41136 | 0.0001272 | 0.0003131 |
| MIR663AHG | 2.042108 | 3.909071 | 0.0001319 | 0.0003236 |
| FAM155A-IT1 | 2.702237 | 3.009884 | 0.0001351 | 0.0003306 |
| AC026401.1 | 2.004782 | 3.112664 | 0.0001381 | 0.0003372 |
| AC016866.2 | 2.394891 | 2.980438 | 0.0001546 | 0.0003754 |
| AC007431.1 | 2.419342 | 3.669689 | 0.0001628 | 0.0003939 |
| AC084816.1 | 2.119734 | 3.146353 | 0.0001668 | 0.0004032 |
| AL391863.1 | 2.289443 | 3.924966 | 0.0001733 | 0.0004172 |
| AC002558.2 | 2.017915 | 3.276124 | 0.0001739 | 0.0004184 |
| LINC02241 | 3.382963 | 3.421699 | 0.0001749 | 0.0004207 |
| AL583808.1 | 2.592044 | 3.520846 | 0.0001819 | 0.0004363 |
| SMYD3-IT1 | 2.127778 | 3.274918 | 0.0001822 | 0.000437 |
| DOCK4-AS1 | 2.838739 | 3.788483 | 0.000184 | 0.0004401 |
| AC018607.1 | 2.960026 | 3.295755 | 0.0001864 | 0.0004456 |
| AC011840.1 | 3.93324 | 4.292426 | 0.000187 | 0.0004468 |
| AP003696.1 | 2.655378 | 3.785047 | 0.0001889 | 0.0004513 |
| AP000919.2 | 2.701249 | 3.620361 | 0.0001921 | 0.0004583 |
| E2F3-IT1 | 2.730415 | 3.476013 | 0.000196 | 0.0004669 |
| AC110792.2 | 2.86438 | 3.715611 | 0.0001995 | 0.0004741 |
| AC037487.2 | 2.629437 | 3.651287 | 0.0002016 | 0.0004788 |
| AC006141.1 | 2.08922 | 3.787464 | 0.0002029 | 0.0004815 |
| VCAN-AS1 | 2.496959 | 3.773754 | 0.0002039 | 0.0004833 |
| ARHGEF3-AS1 | 2.396034 | 2.976647 | 0.000211 | 0.0004988 |
| AC007849.1 | 2.301626 | 4.102955 | 0.0002163 | 0.0005101 |
| AC068389.3 | 2.669766 | 3.394229 | 0.0002188 | 0.0005156 |
| ARHGAP26-AS1 | 2.161165 | 3.764538 | 0.0002285 | 0.0005369 |
| AC018359.1 | 3.063165 | 3.248503 | 0.0002396 | 0.0005613 |
| DIAPH1-AS1 | 2.738811 | 4.620913 | 0.0002517 | 0.0005865 |
| AC112493.1 | 2.35685 | 3.243578 | 0.0002532 | 0.0005896 |
| PCA3 | 2.009522 | 5.005466 | 0.000254 | 0.000591 |
| AC097478.2 | 2.293685 | 3.231254 | 0.0002808 | 0.000647 |
| NCOA7-AS1 | 2.540731 | 3.098397 | 0.0002855 | 0.0006565 |
| AC108865.2 | 2.371941 | 6.85621 | 0.0002969 | 0.0006801 |
| LINC01606 | 2.326034 | 3.516451 | 0.0003049 | 0.0006977 |
| AL136320.1 | 2.749324 | 3.761054 | 0.0003053 | 0.0006981 |
| AL079303.1 | 2.498478 | 3.910936 | 0.0003068 | 0.0007014 |
| LINC01790 | 2.989421 | 3.13091 | 0.0003073 | 0.0007024 |
| AC046158.1 | 2.67944 | 3.496963 | 0.00033 | 0.0007502 |
| AL031428.1 | 2.247322 | 3.077108 | 0.0003313 | 0.000753 |
| LINC01115 | 2.603741 | 4.004219 | 0.0003318 | 0.0007537 |
| PLCB1-IT1 | 2.786908 | 3.055388 | 0.0003323 | 0.0007546 |
| DSCR4 | 2.718877 | 3.119594 | 0.0003526 | 0.0007963 |
| AF131215.2 | 2.283022 | 4.004608 | 0.0003527 | 0.0007963 |
| AC118555.1 | 2.358084 | 3.168484 | 0.0003945 | 0.0008831 |
| ATG10-AS1 | 2.428228 | 2.997837 | 0.0004175 | 0.000931 |
| CREB3L2-AS1 | 2.429335 | 3.501724 | 0.0004199 | 0.0009358 |
| POU6F2-AS2 | 2.294606 | 4.789483 | 0.0004206 | 0.000937 |
| AC108865.1 | 2.180838 | 8.476728 | 0.0004286 | 0.0009531 |
| LINC01194 | 2.953712 | 3.384389 | 0.0004346 | 0.0009644 |
| AC007533.2 | 2.317444 | 3.50777 | 0.0004411 | 0.0009776 |
| AL133480.1 | 2.25213 | 3.175824 | 0.0004507 | 0.0009973 |
| BCAR4 | 2.05931 | 3.31428 | 0.0004583 | 0.0010134 |
| AC020704.1 | 2.385351 | 2.914413 | 0.0004628 | 0.0010225 |
| AC093001.1 | 2.274719 | 3.488915 | 0.0004743 | 0.0010446 |
| AP000897.1 | 2.586401 | 2.981893 | 0.0004745 | 0.0010447 |
| AL136984.1 | 2.119667 | 4.121668 | 0.0004847 | 0.0010653 |
| AC073569.1 | 2.142276 | 3.799023 | 0.0004906 | 0.0010768 |
| AC107953.2 | 2.454624 | 3.357833 | 0.000493 | 0.0010818 |
| AC108102.1 | 2.431349 | 3.337947 | 0.000539 | 0.0011703 |
| AC131182.1 | 2.332134 | 3.921642 | 0.0005504 | 0.001193 |
| AC107958.3 | 2.336881 | 2.901324 | 0.0006078 | 0.0013053 |
| LINC01413 | 2.195155 | 3.665821 | 0.0006123 | 0.0013134 |
| AC011294.1 | 2.136864 | 3.269126 | 0.0006197 | 0.0013279 |
| LINC01206 | 2.068633 | 3.136235 | 0.0006246 | 0.0013375 |
| Z99127.1 | 2.022178 | 3.653909 | 0.0006257 | 0.0013394 |
| USP12-AS1 | 2.602466 | 3.502274 | 0.0006314 | 0.0013507 |
| AL139120.1 | 3.007835 | 3.631696 | 0.0006478 | 0.0013838 |
| Z99289.3 | 2.210419 | 3.022375 | 0.0006575 | 0.0014024 |
| AL391863.2 | 2.807436 | 3.979578 | 0.0006598 | 0.0014066 |
| AC107958.2 | 2.31363 | 2.956036 | 0.0006887 | 0.001463 |
| LINC00491 | 2.73026 | 4.024551 | 0.0006887 | 0.001463 |
| DTD1-AS1 | 2.526802 | 3.335251 | 0.0006893 | 0.0014634 |
| AL445123.1 | 2.554565 | 3.210062 | 0.0006915 | 0.0014676 |
| AC106799.2 | 2.620319 | 3.114726 | 0.0007 | 0.0014847 |
| LINC02050 | 2.119899 | 3.231031 | 0.0007014 | 0.0014874 |
| AL031658.2 | 2.142458 | 3.190134 | 0.0007252 | 0.0015311 |
| AC002558.3 | 2.186449 | 3.384236 | 0.0007345 | 0.0015497 |
| AL162408.1 | 2.047109 | 3.398336 | 0.0007414 | 0.001564 |
| AC013549.3 | 2.188545 | 3.065884 | 0.0007448 | 0.0015703 |
| AC109454.3 | 2.071734 | 3.899225 | 0.0007605 | 0.0016027 |
| LINC02820 | 2.040021 | 4.45243 | 0.0007777 | 0.0016381 |
| AC087257.1 | 2.338644 | 3.742445 | 0.0007882 | 0.0016593 |
| AC011939.2 | 2.521185 | 3.587883 | 0.0008163 | 0.0017123 |
| AC112178.1 | 2.178856 | 3.073195 | 0.0008411 | 0.001757 |
| AC064874.1 | 2.44415 | 3.513063 | 0.0008583 | 0.0017897 |
| AC005920.2 | 2.408771 | 3.487166 | 0.000863 | 0.001798 |
| AC020661.2 | 2.144466 | 3.111322 | 0.0009004 | 0.0018673 |
| CD200R1L-AS1 | 2.495967 | 3.552313 | 0.0009367 | 0.001936 |
| AL356805.1 | 2.17652 | 3.170594 | 0.0009506 | 0.0019613 |
| AC100800.1 | 2.770738 | 3.773927 | 0.0009643 | 0.0019848 |
| AL158801.3 | 2.14772 | 3.60542 | 0.001094 | 0.002233 |
| AC130650.1 | 2.235438 | 3.105839 | 0.0011238 | 0.0022912 |
| AC007496.1 | 2.310997 | 3.538743 | 0.0011399 | 0.0023207 |
| DIAPH3-AS2 | 2.245884 | 2.999411 | 0.0011678 | 0.0023723 |
| CR392039.4 | 2.212375 | 3.089091 | 0.0011981 | 0.0024263 |
| AC130895.1 | 2.609944 | 3.728885 | 0.0013139 | 0.0026468 |
| AC074035.1 | 2.206786 | 2.929721 | 0.0013555 | 0.0027192 |
| AL391097.1 | 2.551747 | 3.122072 | 0.0013895 | 0.0027797 |
| AL359095.1 | 2.3996 | 3.069088 | 0.0013985 | 0.002794 |
| AC109597.1 | 2.157541 | 3.24372 | 0.001415 | 0.0028237 |
| AC087286.1 | 2.539019 | 3.650031 | 0.0014175 | 0.0028279 |
| AC055733.2 | 2.111545 | 2.96508 | 0.0014294 | 0.0028496 |
| AL049835.1 | 2.115122 | 3.201994 | 0.0014309 | 0.0028511 |
| AL162511.1 | 2.052231 | 3.45955 | 0.0014891 | 0.002953 |
| ZBTB20-AS3 | 2.492677 | 3.132695 | 0.0015472 | 0.0030548 |
| AC078950.1 | 2.124322 | 3.120137 | 0.0015609 | 0.0030794 |
| AC092435.3 | 2.290312 | 2.899517 | 0.0015824 | 0.0031166 |
| AC009884.2 | 2.244811 | 3.284374 | 0.0015883 | 0.0031274 |
| AL807757.1 | 2.242082 | 3.258009 | 0.0015945 | 0.0031388 |
| AP001011.2 | 2.011155 | 3.275159 | 0.0016357 | 0.0032113 |
| BACH1-IT3 | 2.347762 | 2.976473 | 0.0017766 | 0.0034615 |
| AL360091.2 | 2.072961 | 3.190662 | 0.0017905 | 0.0034854 |
| AC012555.2 | 2.404476 | 3.300208 | 0.001799 | 0.0035005 |
| LINC02470 | 2.086166 | 3.393659 | 0.0018778 | 0.0036381 |
| AC096741.1 | 2.55018 | 3.80291 | 0.0019215 | 0.0037091 |
| LINC02347 | 2.103638 | 3.215303 | 0.0019288 | 0.003722 |
| AL359382.1 | 2.126973 | 2.956589 | 0.0019403 | 0.0037413 |
| AC069079.1 | 2.485468 | 3.21994 | 0.002016 | 0.0038758 |
| AC113398.2 | 2.385194 | 2.987051 | 0.0020629 | 0.0039608 |
| MACROD2-AS1 | 2.039737 | 3.116062 | 0.0020751 | 0.0039831 |
| AL035634.1 | 2.216225 | 3.386961 | 0.0021232 | 0.0040701 |
| AC012568.1 | 2.279947 | 3.284409 | 0.0022001 | 0.0042086 |
| AF178030.1 | 2.345301 | 3.053339 | 0.0022701 | 0.0043299 |
| LINC02830 | 2.132435 | 3.941592 | 0.0022937 | 0.0043704 |
| AL049820.1 | 2.006947 | 3.166552 | 0.0023968 | 0.0045536 |
| AC092435.1 | 2.329198 | 3.12652 | 0.0025305 | 0.0047864 |
| CACNA1C-IT3 | 2.123632 | 3.031015 | 0.0026438 | 0.0049747 |
| MAGI1-AS1 | 2.185977 | 3.661598 | 0.0026629 | 0.0050057 |
| AC133106.1 | 2.280418 | 3.264001 | 0.0027362 | 0.005126 |
| AC097512.1 | 2.336605 | 3.284878 | 0.0027552 | 0.0051535 |
| AC009248.2 | 2.022352 | 2.97394 | 0.0030526 | 0.0056689 |
| BIRC6-AS1 | 2.284827 | 3.416518 | 0.0035555 | 0.0064931 |
| ZBTB20-AS5 | 2.22376 | 3.887015 | 0.0037953 | 0.0068738 |
| AC009303.3 | 2.067911 | 3.221934 | 0.0043182 | 0.0077359 |
| AC026124.1 | 2.028594 | 3.750751 | 0.0044399 | 0.0079323 |
| LATS2-AS1 | 2.126296 | 3.061802 | 0.0048729 | 0.0086293 |
| AL354794.1 | 2.03544 | 3.253598 | 0.0050453 | 0.0089168 |
| AC012404.1 | 2.09281 | 3.003067 | 0.0052436 | 0.0092295 |
| AC005344.1 | 2.06757 | 3.051213 | 0.0056214 | 0.0098443 |
| AL357793.2 | 2.018113 | 3.051521 | 0.0103733 | 0.0171469 |

| **Table S3: The summary table of DEmiRNA** | | | | |
| --- | --- | --- | --- | --- |
| **miRNA** | **logFC** | **logCPM** | **PValue** | **FDR** |
| hsa-mir-328 | -5.19858 | 4.74621 | 0 | 0 |
| hsa-mir-197 | -4.12201 | 8.385394 | 1.23E-225 | 3.50E-223 |
| hsa-let-7d | -3.0451 | 9.410093 | 1.52E-164 | 2.88E-162 |
| hsa-mir-139 | -4.5258 | 5.421907 | 6.49E-132 | 9.22E-130 |
| hsa-mir-766 | -3.81334 | 3.044337 | 2.36E-109 | 2.69E-107 |
| hsa-mir-1306 | -3.56078 | 3.175322 | 1.05E-97 | 9.95E-96 |
| hsa-mir-504 | -5.64559 | 0.633364 | 2.86E-91 | 2.32E-89 |
| hsa-mir-486-2 | -4.76878 | 5.842304 | 7.64E-90 | 5.42E-88 |
| hsa-mir-486-1 | -4.72857 | 5.838351 | 1.64E-85 | 1.04E-83 |
| hsa-mir-125a | -3.20095 | 8.510296 | 8.47E-84 | 4.81E-82 |
| hsa-mir-21 | 6.707773 | 17.71653 | 6.60E-80 | 3.41E-78 |
| hsa-mir-6511b-2 | -4.66254 | 0.683125 | 5.67E-79 | 2.68E-77 |
| hsa-mir-6511b-1 | -4.59166 | 0.638175 | 4.95E-73 | 2.16E-71 |
| hsa-mir-1976 | -3.32234 | 3.331847 | 5.33E-71 | 2.16E-69 |
| hsa-mir-574 | -3.20897 | 6.08936 | 3.60E-65 | 1.36E-63 |
| hsa-let-7b | -2.60915 | 13.36953 | 9.32E-62 | 3.31E-60 |
| hsa-mir-101-1 | 5.85983 | 11.67253 | 4.79E-54 | 1.60E-52 |
| hsa-mir-101-2 | 5.844372 | 11.68299 | 7.52E-54 | 2.37E-52 |
| hsa-mir-149 | -3.63924 | 3.998682 | 7.19E-52 | 2.15E-50 |
| hsa-mir-141 | 5.626973 | 10.90641 | 7.50E-48 | 2.13E-46 |
| hsa-mir-423 | -2.14413 | 7.243559 | 7.94E-45 | 2.15E-43 |
| hsa-mir-374a | 8.10619 | 9.998443 | 1.22E-43 | 3.15E-42 |
| hsa-mir-1296 | -3.01245 | 2.619689 | 1.06E-41 | 2.62E-40 |
| hsa-mir-99b | -2.37423 | 13.92688 | 4.11E-40 | 9.72E-39 |
| hsa-mir-542 | 6.260095 | 7.358217 | 2.26E-39 | 5.14E-38 |
| hsa-mir-126 | 4.578766 | 11.14862 | 4.54E-39 | 9.91E-38 |
| hsa-mir-193a | -2.84547 | 7.375443 | 4.97E-39 | 1.05E-37 |
| hsa-mir-374b | 4.800423 | 6.267666 | 1.03E-38 | 2.09E-37 |
| hsa-mir-19b-1 | 7.010148 | 7.12435 | 5.48E-38 | 1.07E-36 |
| hsa-mir-98 | 4.913563 | 5.796146 | 1.02E-37 | 1.93E-36 |
| hsa-mir-24-2 | 2.892157 | 10.02583 | 1.36E-37 | 2.49E-36 |
| hsa-mir-29b-1 | 5.717211 | 8.811259 | 6.78E-37 | 1.20E-35 |
| hsa-mir-335 | 5.532972 | 7.887252 | 1.06E-36 | 1.82E-35 |
| hsa-mir-19b-2 | 7.417232 | 6.941338 | 1.11E-35 | 1.86E-34 |
| hsa-mir-24-1 | 2.787338 | 10.0159 | 1.81E-35 | 2.94E-34 |
| hsa-mir-2355 | 4.882017 | 5.20428 | 2.51E-35 | 3.96E-34 |
| hsa-mir-15a | 4.237166 | 7.059676 | 1.28E-34 | 1.96E-33 |
| hsa-mir-1180 | -3.14328 | 3.74883 | 3.48E-34 | 5.21E-33 |
| hsa-mir-16-2 | 4.946431 | 8.50801 | 4.68E-33 | 6.82E-32 |
| hsa-mir-145 | -3.04966 | 11.64236 | 5.32E-33 | 7.55E-32 |
| hsa-mir-129-2 | -4.23169 | 1.722683 | 3.00E-32 | 4.16E-31 |
| hsa-mir-30e | 2.586014 | 13.17738 | 1.24E-31 | 1.68E-30 |
| hsa-mir-16-1 | 4.832719 | 8.493511 | 1.53E-31 | 2.02E-30 |
| hsa-mir-642a | -3.54365 | 2.416057 | 2.66E-31 | 3.43E-30 |
| hsa-mir-152 | 4.189857 | 7.697593 | 3.84E-31 | 4.85E-30 |
| hsa-mir-129-1 | -4.21592 | 1.620865 | 1.04E-30 | 1.28E-29 |
| hsa-mir-150 | -2.96891 | 8.95717 | 1.77E-30 | 2.10E-29 |
| hsa-mir-590 | 7.91661 | 4.781876 | 1.77E-30 | 2.10E-29 |
| hsa-mir-379 | 4.944921 | 9.661728 | 1.97E-30 | 2.29E-29 |
| hsa-mir-20a | 5.806941 | 9.23138 | 3.29E-30 | 3.74E-29 |
| hsa-mir-92b | -2.50742 | 6.233991 | 2.67E-29 | 2.97E-28 |
| hsa-mir-142 | 7.418583 | 11.45363 | 8.34E-29 | 9.11E-28 |
| hsa-mir-582 | 5.647924 | 8.65113 | 1.32E-28 | 1.41E-27 |
| hsa-mir-182 | 5.516171 | 14.06298 | 2.52E-28 | 2.65E-27 |
| hsa-mir-889 | 6.445574 | 4.072114 | 2.75E-28 | 2.84E-27 |
| hsa-mir-181a-1 | -2.23549 | 9.058175 | 2.96E-28 | 3.00E-27 |
| hsa-mir-424 | 5.956617 | 7.246834 | 5.76E-28 | 5.74E-27 |
| hsa-mir-1249 | -2.99737 | 1.144687 | 9.02E-28 | 8.83E-27 |
| hsa-mir-148a | 4.423645 | 16.10263 | 1.26E-27 | 1.21E-26 |
| hsa-mir-193b | -2.36545 | 5.318698 | 1.31E-27 | 1.24E-26 |
| hsa-mir-454 | 6.296498 | 3.212716 | 1.91E-27 | 1.78E-26 |
| hsa-mir-708 | 5.756197 | 5.914685 | 2.82E-27 | 2.58E-26 |
| hsa-mir-452 | 5.478205 | 6.985548 | 4.02E-27 | 3.62E-26 |
| hsa-mir-19a | 10.85009 | 6.090823 | 5.10E-27 | 4.53E-26 |
| hsa-mir-17 | 3.842667 | 10.56623 | 1.24E-26 | 1.07E-25 |
| hsa-mir-429 | 4.391449 | 8.740581 | 1.79E-26 | 1.52E-25 |
| hsa-mir-3615 | -2.52946 | 2.587354 | 5.33E-26 | 4.45E-25 |
| hsa-mir-326 | -2.76994 | 2.411899 | 6.24E-26 | 5.14E-25 |
| hsa-mir-1226 | -3.09301 | 0.936495 | 1.89E-24 | 1.49E-23 |
| hsa-mir-2110 | -2.50366 | 1.753535 | 2.62E-24 | 2.04E-23 |
| hsa-mir-660 | 4.678683 | 5.732725 | 2.84E-24 | 2.18E-23 |
| hsa-mir-32 | 3.856631 | 5.218139 | 1.25E-23 | 9.46E-23 |
| hsa-mir-485 | -2.57742 | 2.035833 | 1.85E-23 | 1.38E-22 |
| hsa-mir-96 | 5.698973 | 4.41622 | 2.02E-23 | 1.49E-22 |
| hsa-mir-605 | -3.21935 | 0.294083 | 7.93E-23 | 5.77E-22 |
| hsa-mir-135b | 9.917775 | 6.742001 | 9.66E-23 | 6.94E-22 |
| hsa-mir-6720 | -3.42862 | 0.242335 | 9.84E-23 | 6.99E-22 |
| hsa-mir-7-1 | 3.694688 | 5.870456 | 9.98E-23 | 7.00E-22 |
| hsa-mir-26b | 2.84644 | 9.367646 | 1.06E-22 | 7.37E-22 |
| hsa-mir-3173 | -3.08533 | 0.313153 | 1.19E-22 | 8.13E-22 |
| hsa-mir-30b | 3.737942 | 8.468379 | 2.76E-22 | 1.83E-21 |
| hsa-let-7g | 2.281331 | 9.394746 | 3.04E-22 | 1.98E-21 |
| hsa-mir-133a-2 | -3.43635 | 3.977328 | 8.83E-22 | 5.70E-21 |
| hsa-mir-203a | 5.225155 | 14.46621 | 6.59E-21 | 4.19E-20 |
| hsa-mir-450b | 8.043325 | 3.351248 | 6.64E-21 | 4.19E-20 |
| hsa-mir-433 | -2.63119 | 1.060277 | 1.22E-20 | 7.60E-20 |
| hsa-mir-1343 | -3.1142 | 0.28227 | 1.29E-20 | 7.96E-20 |
| hsa-mir-186 | 2.076199 | 8.209456 | 2.97E-20 | 1.79E-19 |
| hsa-mir-203b | 6.724575 | 7.769081 | 3.50E-20 | 2.09E-19 |
| hsa-mir-3917 | -2.80181 | 1.241339 | 4.33E-20 | 2.56E-19 |
| hsa-mir-3127 | -2.34909 | 3.102004 | 5.29E-20 | 3.10E-19 |
| hsa-mir-22 | 2.468519 | 15.57926 | 7.60E-20 | 4.41E-19 |
| hsa-mir-369 | 4.229124 | 3.774814 | 1.18E-19 | 6.79E-19 |
| hsa-mir-27a | 2.870185 | 10.70537 | 2.38E-19 | 1.35E-18 |
| hsa-mir-144 | 6.316968 | 6.407134 | 4.64E-19 | 2.60E-18 |
| hsa-mir-26a-1 | 2.417619 | 9.765729 | 4.66E-19 | 2.60E-18 |
| hsa-mir-136 | 4.746183 | 5.179218 | 5.82E-19 | 3.21E-18 |
| hsa-mir-3613 | 4.530195 | 4.049674 | 6.92E-19 | 3.78E-18 |
| hsa-mir-4677 | 4.403879 | 2.945646 | 7.41E-19 | 4.01E-18 |
| hsa-mir-133a-1 | -3.2727 | 4.145472 | 8.87E-19 | 4.75E-18 |
| hsa-mir-26a-2 | 2.433067 | 9.768341 | 9.04E-19 | 4.80E-18 |
| hsa-mir-29b-2 | 3.258054 | 8.821088 | 9.52E-19 | 5.01E-18 |
| hsa-mir-337 | 3.256503 | 4.743098 | 2.22E-18 | 1.16E-17 |
| hsa-mir-151a | 2.549277 | 11.37608 | 7.17E-18 | 3.70E-17 |
| hsa-mir-7706 | -2.2389 | 1.588887 | 1.25E-17 | 6.38E-17 |
| hsa-mir-185 | 2.183846 | 5.886267 | 3.12E-17 | 1.57E-16 |
| hsa-mir-7702 | -3.36838 | 0.91615 | 1.08E-16 | 5.40E-16 |
| hsa-mir-340 | 3.356327 | 4.329589 | 1.15E-16 | 5.66E-16 |
| hsa-mir-3940 | -2.68654 | 1.398128 | 2.06E-16 | 1.01E-15 |
| hsa-mir-183 | 3.538166 | 13.10171 | 4.61E-16 | 2.22E-15 |
| hsa-mir-6793 | -2.9542 | -0.01756 | 5.12E-16 | 2.44E-15 |
| hsa-mir-495 | 4.039697 | 2.879716 | 5.42E-16 | 2.56E-15 |
| hsa-mir-34a | 2.710946 | 6.895963 | 6.30E-16 | 2.96E-15 |
| hsa-mir-199b | 3.625625 | 11.49535 | 6.54E-16 | 3.05E-15 |
| hsa-mir-153-2 | 6.344118 | 5.515734 | 8.66E-16 | 3.97E-15 |
| hsa-mir-192 | 3.692649 | 16.21884 | 1.04E-15 | 4.74E-15 |
| hsa-mir-1295a | -3.11971 | -0.17024 | 1.71E-15 | 7.71E-15 |
| hsa-mir-376c | 5.806358 | 2.741594 | 1.99E-15 | 8.90E-15 |
| hsa-mir-411 | 5.151433 | 3.244177 | 3.54E-15 | 1.57E-14 |
| hsa-mir-301a | 5.527194 | 3.963733 | 3.93E-15 | 1.73E-14 |
| hsa-mir-194-1 | 2.58315 | 13.15225 | 6.20E-15 | 2.71E-14 |
| hsa-mir-577 | 8.240307 | 6.853522 | 7.03E-15 | 3.05E-14 |
| hsa-mir-10a | 3.832711 | 16.27861 | 1.15E-14 | 4.96E-14 |
| hsa-mir-6892 | -2.13617 | 1.40423 | 2.40E-14 | 1.03E-13 |
| hsa-mir-552 | 5.786051 | 7.727426 | 3.95E-14 | 1.67E-13 |
| hsa-mir-592 | 10.04379 | 5.293931 | 9.45E-14 | 3.97E-13 |
| hsa-mir-5187 | -2.62031 | -0.02757 | 1.48E-13 | 6.19E-13 |
| hsa-mir-199a-2 | 2.957724 | 11.04821 | 1.82E-13 | 7.53E-13 |
| hsa-mir-628 | 5.993335 | 4.454074 | 3.62E-13 | 1.49E-12 |
| hsa-mir-3605 | -2.24646 | 1.838794 | 4.67E-13 | 1.91E-12 |
| hsa-mir-199a-1 | 2.636992 | 10.21597 | 1.36E-12 | 5.45E-12 |
| hsa-mir-6808 | -2.77915 | -0.09644 | 2.04E-12 | 8.05E-12 |
| hsa-let-7f-2 | 4.647137 | 12.18525 | 2.54E-12 | 9.94E-12 |
| hsa-let-7f-1 | 4.610169 | 12.16457 | 3.04E-12 | 1.18E-11 |
| hsa-mir-29c | 3.30716 | 10.04879 | 3.59E-12 | 1.39E-11 |
| hsa-mir-125b-1 | -2.01877 | 7.166518 | 3.72E-12 | 1.43E-11 |
| hsa-mir-10b | 2.842525 | 15.22047 | 4.14E-12 | 1.58E-11 |
| hsa-mir-190a | 3.477488 | 2.79467 | 4.34E-12 | 1.63E-11 |
| hsa-mir-450a-2 | 6.460719 | 1.907977 | 4.40E-12 | 1.65E-11 |
| hsa-mir-421 | 4.418369 | 2.5743 | 5.16E-12 | 1.92E-11 |
| hsa-mir-196a-2 | 3.595147 | 7.263626 | 5.21E-12 | 1.92E-11 |
| hsa-mir-125b-2 | -2.00924 | 7.207166 | 6.71E-12 | 2.46E-11 |
| hsa-mir-375 | -2.14973 | 14.90547 | 7.29E-12 | 2.65E-11 |
| hsa-mir-1224 | -3.76954 | 1.445507 | 1.22E-11 | 4.42E-11 |
| hsa-mir-1228 | -2.23169 | 0.626533 | 1.26E-11 | 4.53E-11 |
| hsa-mir-450a-1 | 6.466424 | 1.912031 | 1.27E-11 | 4.53E-11 |
| hsa-mir-95 | 3.492387 | 4.115231 | 2.14E-11 | 7.58E-11 |
| hsa-mir-6802 | -2.71789 | -0.13097 | 3.18E-11 | 1.12E-10 |
| hsa-mir-760 | -2.50054 | 0.80332 | 4.05E-11 | 1.42E-10 |
| hsa-mir-217 | 4.461894 | 5.373001 | 6.15E-11 | 2.14E-10 |
| hsa-mir-187 | -3.38591 | 1.374517 | 6.93E-11 | 2.40E-10 |
| hsa-mir-490 | -3.89491 | 2.76213 | 7.06E-11 | 2.43E-10 |
| hsa-mir-130a | 2.197525 | 5.290266 | 7.49E-11 | 2.56E-10 |
| hsa-mir-4676 | -2.19283 | 0.625176 | 8.21E-11 | 2.79E-10 |
| hsa-mir-188 | 3.787019 | 3.065584 | 8.96E-11 | 3.03E-10 |
| hsa-mir-598 | 3.771098 | 4.003088 | 1.04E-10 | 3.50E-10 |
| hsa-mir-215 | 5.294307 | 10.00158 | 1.32E-10 | 4.38E-10 |
| hsa-mir-224 | 2.933815 | 6.535535 | 1.74E-10 | 5.73E-10 |
| hsa-mir-3677 | 3.32716 | 3.385685 | 1.77E-10 | 5.82E-10 |
| hsa-mir-3928 | -2.05872 | 1.136485 | 1.94E-10 | 6.33E-10 |
| hsa-mir-493 | 2.135556 | 3.901506 | 2.06E-10 | 6.70E-10 |
| hsa-mir-651 | 4.258806 | 2.017033 | 3.46E-10 | 1.11E-09 |
| hsa-mir-33a | 3.469778 | 5.97245 | 5.33E-10 | 1.69E-09 |
| hsa-mir-1-1 | 6.374519 | 4.857387 | 6.59E-10 | 2.08E-09 |
| hsa-mir-2116 | -2.07183 | 0.474369 | 7.36E-10 | 2.31E-09 |
| hsa-mir-18a | 2.775753 | 5.185987 | 8.39E-10 | 2.62E-09 |
| hsa-mir-6125 | -2.58505 | 0.040088 | 9.09E-10 | 2.82E-09 |
| hsa-mir-7-2 | 5.465817 | 2.480553 | 1.15E-09 | 3.55E-09 |
| hsa-mir-451a | 3.591446 | 8.361746 | 1.59E-09 | 4.89E-09 |
| hsa-mir-143 | 3.771714 | 17.39164 | 2.00E-09 | 6.11E-09 |
| hsa-mir-653 | 7.884402 | 3.197115 | 2.06E-09 | 6.25E-09 |
| hsa-mir-223 | 3.386195 | 8.550779 | 2.32E-09 | 6.96E-09 |
| hsa-mir-5010 | -2.00206 | 0.483877 | 2.73E-09 | 8.18E-09 |
| hsa-mir-218-2 | 3.587148 | 3.312956 | 4.34E-09 | 1.29E-08 |
| hsa-mir-196a-1 | 2.813367 | 7.231548 | 4.91E-09 | 1.45E-08 |
| hsa-mir-7-3 | 4.727816 | 2.493306 | 8.62E-09 | 2.50E-08 |
| hsa-mir-3944 | -2.52185 | -0.08425 | 8.63E-09 | 2.50E-08 |
| hsa-mir-4668 | 6.135661 | 1.663676 | 1.11E-08 | 3.21E-08 |
| hsa-mir-1-2 | 5.376742 | 4.929457 | 1.21E-08 | 3.47E-08 |
| hsa-mir-5000 | 4.125906 | 1.326512 | 1.69E-08 | 4.80E-08 |
| hsa-mir-576 | 2.24444 | 3.702195 | 1.78E-08 | 5.02E-08 |
| hsa-mir-146a | 2.571918 | 7.965912 | 2.38E-08 | 6.66E-08 |
| hsa-mir-584 | 2.641875 | 8.430771 | 2.87E-08 | 8.00E-08 |
| hsa-mir-496 | 3.859629 | 1.709147 | 3.38E-08 | 9.35E-08 |
| hsa-mir-338 | 3.166808 | 9.076295 | 3.63E-08 | 1.00E-07 |
| hsa-mir-1245a | 5.598609 | 1.219067 | 5.21E-08 | 1.42E-07 |
| hsa-mir-3150b | -2.28593 | 2.153726 | 5.21E-08 | 1.42E-07 |
| hsa-mir-4728 | -2.53945 | 1.18045 | 5.30E-08 | 1.44E-07 |
| hsa-mir-4662a | 4.178244 | 3.593918 | 6.75E-08 | 1.83E-07 |
| hsa-mir-376a-1 | 5.582899 | 1.207913 | 7.78E-08 | 2.09E-07 |
| hsa-mir-301b | 4.733681 | 1.834868 | 1.13E-07 | 3.01E-07 |
| hsa-mir-655 | 4.240318 | 1.389224 | 1.15E-07 | 3.04E-07 |
| hsa-mir-1277 | 5.478267 | 1.13775 | 1.56E-07 | 4.11E-07 |
| hsa-mir-376b | 4.151041 | 1.32221 | 1.58E-07 | 4.12E-07 |
| hsa-mir-5586 | 5.365671 | 1.042446 | 1.67E-07 | 4.35E-07 |
| hsa-mir-7641-1 | -2.90769 | 0.540392 | 1.83E-07 | 4.74E-07 |
| hsa-mir-33b | 3.72962 | 2.950251 | 2.14E-07 | 5.51E-07 |
| hsa-mir-627 | 3.298656 | 1.641692 | 2.20E-07 | 5.63E-07 |
| hsa-mir-106a | 4.03782 | 5.581843 | 2.63E-07 | 6.66E-07 |
| hsa-mir-181d | 2.829364 | 4.449722 | 2.86E-07 | 7.19E-07 |
| hsa-mir-196b | 3.179821 | 11.84248 | 3.37E-07 | 8.44E-07 |
| hsa-mir-615 | -2.82648 | 1.275067 | 1.07E-06 | 2.57E-06 |
| hsa-mir-549a | 5.486787 | 1.141131 | 1.18E-06 | 2.82E-06 |
| hsa-mir-153-1 | 5.735258 | 1.328813 | 1.93E-06 | 4.56E-06 |
| hsa-mir-556 | 5.286052 | 0.999 | 2.08E-06 | 4.91E-06 |
| hsa-mir-6798 | -2.22307 | -0.11027 | 2.21E-06 | 5.16E-06 |
| hsa-mir-1229 | -2.0596 | 0.313615 | 2.50E-06 | 5.83E-06 |
| hsa-mir-6820 | -2.13809 | -0.02469 | 3.83E-06 | 8.77E-06 |
| hsa-mir-3682 | 4.85631 | 0.680142 | 6.28E-06 | 1.43E-05 |
| hsa-mir-494 | 3.283302 | 1.237582 | 6.40E-06 | 1.45E-05 |
| hsa-mir-4787 | -2.2842 | -0.12379 | 8.72E-06 | 1.96E-05 |
| hsa-mir-7705 | 4.990651 | 0.780367 | 1.45E-05 | 3.19E-05 |
| hsa-mir-4444-2 | 4.798707 | 0.688993 | 1.54E-05 | 3.39E-05 |
| hsa-mir-218-1 | 2.192583 | 3.380294 | 1.64E-05 | 3.57E-05 |
| hsa-mir-4791 | 5.193531 | 0.935237 | 2.78E-05 | 5.97E-05 |
| hsa-mir-643 | 4.545449 | 0.511504 | 3.41E-05 | 7.30E-05 |
| hsa-mir-147b | 2.796625 | 2.593321 | 3.63E-05 | 7.76E-05 |
| hsa-mir-570 | 4.794398 | 0.649475 | 6.02E-05 | 0.000127 |
| hsa-mir-3662 | 4.887507 | 0.717746 | 6.53E-05 | 0.000137 |
| hsa-mir-559 | 4.477778 | 0.458523 | 0.000115 | 0.000238 |
| hsa-mir-3913-1 | 3.246796 | 0.670003 | 0.000122 | 0.000249 |
| hsa-mir-380 | 4.680162 | 0.562043 | 0.000134 | 0.000274 |
| hsa-mir-4444-1 | 4.207354 | 0.341307 | 0.000146 | 0.000296 |
| hsa-mir-376a-2 | 4.556438 | 0.493251 | 0.000153 | 0.00031 |
| hsa-mir-580 | 4.315152 | 0.359292 | 0.00018 | 0.000363 |
| hsa-mir-3065 | 2.170507 | 3.911471 | 0.000277 | 0.000549 |
| hsa-mir-508 | 4.985721 | 5.315162 | 0.000314 | 0.000619 |
| hsa-mir-499a | 5.71204 | 1.313273 | 0.000318 | 0.000625 |
| hsa-mir-216a | 3.032458 | 1.017147 | 0.00033 | 0.000643 |
| hsa-mir-545 | 4.378364 | 0.424276 | 0.000337 | 0.000656 |
| hsa-mir-548f-1 | 5.187602 | 0.932764 | 0.000429 | 0.000821 |
| hsa-mir-656 | 4.342298 | 0.378815 | 0.000447 | 0.000853 |
| hsa-mir-6516 | 4.003182 | 0.204999 | 0.000526 | 0.000997 |
| hsa-mir-5683 | 6.361302 | 1.870987 | 0.000543 | 0.001025 |
| hsa-mir-3912 | 3.948868 | 0.143438 | 0.000598 | 0.001125 |
| hsa-mir-9-1 | 2.358898 | 7.072004 | 0.000644 | 0.001208 |
| hsa-mir-412 | 2.337882 | 2.67758 | 0.000682 | 0.001269 |
| hsa-mir-9-2 | 2.29491 | 7.072022 | 0.000853 | 0.001578 |
| hsa-mir-9-3 | 2.28591 | 7.069091 | 0.000885 | 0.001622 |
| hsa-mir-514a-3 | 6.914984 | 2.255753 | 0.000936 | 0.00171 |
| hsa-mir-3117 | 4.192339 | 0.285701 | 0.00101 | 0.001833 |
| hsa-mir-514a-1 | 6.832237 | 2.186726 | 0.001014 | 0.001835 |
| hsa-mir-6854 | 2.420439 | 0.618849 | 0.001039 | 0.001874 |
| hsa-mir-514a-2 | 6.923421 | 2.263548 | 0.001118 | 0.002009 |
| hsa-mir-4766 | 3.984503 | 0.205603 | 0.001234 | 0.002198 |
| hsa-mir-3913-2 | 2.044636 | 0.689116 | 0.001326 | 0.002347 |
| hsa-mir-1255a | 3.873939 | 0.141023 | 0.001403 | 0.002474 |
| hsa-mir-4999 | 2.959426 | 0.534493 | 0.001523 | 0.002678 |
| hsa-mir-3189 | 4.015813 | 0.207492 | 0.001755 | 0.003052 |
| hsa-mir-3942 | 3.862043 | 0.114875 | 0.001757 | 0.003052 |
| hsa-mir-5706 | 3.753111 | 0.081509 | 0.001885 | 0.003254 |
| hsa-mir-3664 | 2.792473 | 0.435909 | 0.002076 | 0.003573 |
| hsa-mir-548o | 3.635881 | 0.014433 | 0.002568 | 0.00438 |
| hsa-mir-4709 | 3.7633 | 0.068771 | 0.003151 | 0.005343 |
| hsa-mir-374c | 5.597548 | 1.142247 | 0.004269 | 0.007098 |
| hsa-mir-7974 | 2.823118 | 0.895669 | 0.004274 | 0.007098 |
| hsa-mir-561 | 3.988286 | 0.192403 | 0.004681 | 0.007728 |
| hsa-mir-34b | 2.938896 | 0.504003 | 0.005394 | 0.008855 |
| hsa-mir-6733 | 3.514162 | -0.0287 | 0.005811 | 0.009513 |
| hsa-mir-548au | 3.5888 | 0.029028 | 0.006321 | 0.010288 |
| hsa-mir-31 | 2.744196 | 4.552708 | 0.006841 | 0.011038 |
| hsa-mir-3684 | 3.456076 | -0.04419 | 0.006894 | 0.011092 |
| hsa-mir-206 | 4.574834 | 0.517782 | 0.007916 | 0.012629 |
| hsa-mir-5696 | 3.383956 | -0.09004 | 0.010062 | 0.015919 |
| hsa-mir-3941 | 2.292837 | 0.161605 | 0.010268 | 0.016201 |
| hsa-mir-5684 | 3.382468 | -0.08017 | 0.010738 | 0.01686 |
| hsa-mir-3136 | 2.00239 | 0.409815 | 0.010745 | 0.01686 |
| hsa-mir-665 | 3.342426 | -0.10536 | 0.012699 | 0.019762 |
| hsa-mir-372 | 8.21615 | 3.552775 | 0.01274 | 0.019772 |
| hsa-mir-1185-1 | 3.391703 | -0.09115 | 0.012873 | 0.019923 |
| hsa-mir-466 | 4.274997 | 0.346422 | 0.013274 | 0.020488 |
| hsa-mir-526b | 3.989541 | 0.210618 | 0.013879 | 0.021363 |
| hsa-mir-3654 | 3.799262 | 0.097047 | 0.015278 | 0.023327 |
| hsa-mir-509-2 | 2.495523 | 2.441489 | 0.024965 | 0.037016 |
| hsa-mir-573 | 2.317635 | 0.148028 | 0.025857 | 0.038048 |
| hsa-mir-7156 | 3.33181 | -0.12315 | 0.026292 | 0.038589 |
| hsa-mir-509-1 | 2.420463 | 2.373468 | 0.026393 | 0.038637 |
| hsa-mir-641 | 3.185271 | -0.15829 | 0.027275 | 0.039724 |

**Table S4: Grouping statistics for training group, test group and validation group**

| Characteristics | Variables | TCGA  Training(n=248) | TCGA  Test(n=248) | GEO Validation(n=294) |
| --- | --- | --- | --- | --- |
| Age | <=65 | 105 (42.34%) | 116(46.77%) | 118(40.14%) |
|  | >65 | 143 (57.66%) | 132(53.23%) | 176(59.86%) |
| Gender | FEMALE | 96 (38.71%) | 130(52.42%) | 136(46.26%) |
|  | MALE | 152 (61.29%) | 118(47.58%) | 158(53.74%) |
| AJCC-Stage | StageⅠ | 39 (15.73%) | 46(18.55%) | 24(8.16%) |
|  | StageⅡ | 94 (37.90%) | 91(36.69%) | 135(45.92%) |
|  | StageⅢ | 70 (28.23%) | 70(28.23%) | 125(42.52%) |
|  | StageⅣ | 39 (15.72%) | 32(12.90%) | 10(3.40%) |
|  | unknow | 6 (2.42%) | 9(3.63%) | 0(0.00%) |
